# ABSTRACTS OF THE WORKS PRESENTED AT THE 22^nd^ Academic Conference of the Bauru School of Dentistry “Dr. Waldyr Antonio Janson”

**DOI:** 10.1590/S1678-77572009000700022

**Published:** 2009

**Authors:** Carlos Eduardo Francischone, Rafael Massunari Maenososno

## 001 Local trauma: An important triggering factor for gingival recession

BORGES FILHO, F.F. (Fausto Frizzera Borges Filho faustofrizzera@yahoo.com.br); FINOTTI, L.; ORRICO, S.R.P.

Smile harmony could be influenced by the smile line, gingival contour, lip position and correct tooth arrangement and shape. Alterations in gingival tissue, such as hyperplasia, bone crest loss compromising the gingival papillae, uneven contour and exposure of root surfaces, are factors that affect negatively this harmony. Gingival recession (GR) is the apical migration of gingival margin exposing the cementoenamel junction. It may cause unsatisfactory esthetics, dentinal hypersensitivity, root cavities, dental abrasion and other alterations. GR have a multifactorial origin, and could affect a significant part of the population with good oral hygiene. The aim of this study was to evaluate the relationship between GR and local trauma due to deleterious habits and inadequate oral hygiene comparing different research studies and case reports. Oral hygiene factors, such as toothbrushing duration, brushing force, daily frequency and toothbrushing technique, were associated with GR as well as toothbrush characteristics (bristle stiffness) and frequency of replacement. Deleterious habits like thumb- or pacifier-sucking, tongue interposition during swallowing, self-inflicted gingival mutilation cause gingival injury and may also be an important factor related with GR. Therefore it may be concluded that local trauma is one of the main factors that might start and/or lead to GR progression.

## 002 Esthetic parameters in the transformation of maxillary anterior teeth – case report

FAVARÃO, I.N. (Isabella Negro Favarão isinha_fav@hotmail.com); KASUYA, A.V.B.; FABRE, H.S.C.; QUAGLIATTO, O.S.; FONSECA, R.B.

Several principles should be managed during restorative treatment planning and execution in order to achieve harmonic esthetic results. The presence of deficient restorations or unaesthetic dental format contributes to a disharmonic smile, but can be corrected by a direct bonding technique. The aim of this study is to report the sequence of analysis of esthetic parameters and clinical steps, from treatment planning to final polishing, in order to achieve success in esthetic transformation of maxillary anterior teeth. A 45 year-old male patient presented with a disharmonic smile according to the esthetic standards, with grooves and stains in the anterior teeth, ample interdental cervical spaces between teeth 12 and 13, and 11 and 12, and almost no cervical spaces between teeth 11 and 21, 21 and 22 and 22 and 23. The anterior teeth were not in esthetic proportion (width/height ratio). After color selection (VITA shade guide), the maxillary anterior teeth were reshaped with direct bonding technique and then adjusted according to the principles of esthetic harmony and masticatory function. The maxillary anterior teeth showed better esthetic in terms of shape, texture and color, with excellent final outcomes. During dental transformation, professionals must examine all tooth structures, bearing in mind the anatomic and esthetic knowledge, and looking for an ideal model that will assist them during the esthetic and functional dental reconstruction.

## 003 Saliva as a vehicle of *Helicobacter pylori* transmission: influence of age, gender, periodontal and socioeconomic conditions

AZEVEDO, F.A. (Fernanda Almeida de Azevedo fernanda_ada@hotmail.com); MECA, L.B.; LINS, S.A.; GAETTI-JARDIM JR, E.

FAPESP 2007/51016-3.

*Helicobacter pylori* infection is deeply related to the development of gastroduodenal ulcers and oral cavity may act as a reservoir for its transmission, especially in the most populated cities of developing countries. In this study, the occurrence of *H. pylori* in saliva from native and non-native Brazilians was investigated and the influence of age, gender, socioeconomic status and periodontal health conditions on the presence of this microorganism in saliva was also determined. Samples of resting saliva from 150 native Brazilians of 8 different ethnic groups from Mato Grosso and Maranhão States, as well as resting saliva from 500 non-Indians living in urban areas of São Paulo State, aged 6 months to 96 years were collected and stored at −196oC. After extraction of the microbial DNA, the presence of *H. pylori* DNA was evaluated by nested PCR using primers designed to amplify *H. pylori-*specific segments within the bacterial 16S rDNA. The external primers used in first-round PCR were EHC-U and EHC-L, while in the second round of DNA amplification, it was used primers ET-5U and ET-5L producing a 230-bp fragment within the amplicon of the first-round PCR. The occurrence of this bacterium was associated with socioeconomic conditions, regardless of gender and ethnicities, being more frequent in low income people, and subjects aged 30 years or older, as well as individuals with history of use of antimicrobial drugs. In general, *H. pylori* was detected in 38.67% Indians' saliva and 47.5% non-Indians' saliva, regardless of their periodontal health conditions. In edentulous children, *H. pylori* was detected in 7.5% of the samples, but its presence in oral cavity seems to be transient in these children.

## 004 Gram-positive strict anaerobes in periodontitis: evaluation in different Brazilian populations

SALINEIRO, F.C.S. (Fernanda Cristina Sales Salineiro fernanda_css@hotmail.com); GALLO, A.J.; SOARES, A.S.; VIEIRA, E.M.M.; GAETTIJARDIM, E.C.

The role of Gram-negative anaerobes in the etiology of periodontitis has been evaluated but recently some Gram-positive strict anaerobes, such as genera Eubacterium, Mogibacterium and Slackia, are in evidence. The present investigation aimed to evaluate the occurrence of these microorganisms in oral cavity of two groups of adult patients in Brazil: 100 native Brazilians and 350 non-Indian Brazilians living in urban areas of São Paulo State and Maranhão State, of both genders and aged 18-96 years. The detection of *Eubacterium sp., E. saphenum, S. exigua, and M. timidum* was carried out by DNA amplification by conventional PCR using specific primers. The comparisons of plaque index and other clinical parameters were performed by Kruskal-Wallis statistical test. Pairwise comparisons were performed by Bonferroni adjusted nonparametric Mann-Whitney test, while significant differences in the occurrence of selected microorganisms were assessed by weighted least squares method. *Genii Eubacterium* and S. exigua were largely distributed in the population, regardless of the periodontal status and geographical area and ethnicity, while *M. timidum* and *E. saphenum* were rarely isolated from native Brazilians. *E. saphenum* was detected from 31.58% Indians with periodontitis and 14.19% periodontally healthy natives, while its frequency was 46.86% non-Indians with periodontitis and 38.58% periodontally healthy non-Indians. *M. timidum* and *S. exigua* were observed in a higher prevalence in non-Indians, regardless of their periodontal conditions. The presence of *M. timidum* was evidenced from 44% non-Indians with periodontitis and from 23.68% natives with gingivitis, while for *S. exigua* these values were 58.63% and 44.74%, respectively. In these human groups, the data did not support a major role of these Gram-positive anaerobic rods in the development of periodontal infection.

## 005 Treatment of panfacial fractures: case report

FLORIAN, F. (Fernanda Florian fer_florian@hotmail.com); NOGUEIRA, L.M.; TRINDADE, P.A.K.; PEREIRA FILHO, V.A.; GABRIELLI, M.F.R.

The treatment of panfacial fractures requires extreme care and experience due to the complexity of structures and sense bodies related to the face. The proper reestablishment of the facial projection, function and esthetics must be considered together in the treatment of panfacial fractures. This report describes the treatment of a victim of car accident, which was rescued by the Emergency Mobile Healthcare Service (SAMU) and forwarded to the Urgency and Emergency Service of the Santa Casa de Misericórdia Hospital of Araraquara. This work describes the approach of the patient by the Oral and Maxillofacial Surgery and Traumatology Team of Araraquara Dental School and the sequence of the treatment accomplished, as well as the techniques used for the reduction and fixation of the complex facial fractures. The patient presented favorable evolution without postoperative functional changes after years of treatment, demonstrating the importance of knowledge of the sequence of treatment in this type complex facial fracture.

## 006 Criteria for replacement of restorations in dental practice

FARIA, F.G. (Fernanda Gabriela de Faria nandagf18@gmail.com); SILVA, G.R.; CABRAL, F.C.; NOVAIS, V.R.; MARTINS, L.R.M.; SOARES, C.J.

The replacement of restorations is a common procedure in clinical dentistry. Therefore, it is important that the professional knows how to evaluate carefully the clinical conditions of the restorations to define the better treatment. To avoid that the criteria for replacement of restorations are purely subjective, some specific criteria have been raised in the scientific literature and mostly are related to mechanical, functional, biological and esthetic failures. In this work, data related to the longevity of restorations and the main reasons for replacement are shown and treatment options are addressed based on clinical characteristics. The scientific evidence was obtained with papers indexed in PubMed database in the last 10 years. Additionally, repair, recontouring and repolishing of direct amalgam and composite resin restorations are discussed. The main criteria employed in the literature are usually modifications of the United States Public Health Service (USPH). The main reasons for replacing restorations are: secondary caries, deficiencies on marginal adaptation or occlusal and proximal contact, staining and color change, roughness, loss of anatomical shape and brightness, and postoperative sensitivity. It may be concluded that only the appearance of the restorations should not be used as indicative of the need for replacement. The recontouring and repair are alternatives to the replacement of restorations, as they present advantages such as increasing the longevity of the restoration with low cost and preservation of tooth structures.

## 007 Clinical evaluation in patients with temporomandibular disorders subjected to the therapy with occlusal splints

MARGON, F.O. (Fernanda de Oliveira Margon nandamargon@hotmail.com); STRINI, P.J.S.A.; MACHADO, N.A.G.; FERNANDES-NETO, A.J.; SOUSA, G.C.; BERNADINO, R.

The purpose of this study was to evaluate clinical changes in individuals with temporomandibular disorders (TMD) subjected to therapy with occlusal splints. Twenty patients with signs and symptoms of TMD who met the inclusion and exclusion criteria participated in the study. The patients were assessed by clinical examination during which it was observed the side of mastication, occlusal interference and joint pain before and after 30 days of therapy with stabilizing occlusal splint. An acetate interocclusal device with maxillary full-coverage was made for each participant. These devices were relined with colorless autopolymerized acrylic resin and adjusted in centric relation with the disocclusion pattern by anterior guidance. The initial data were compared to results obtained after 30 days of use of the splint. It was observed that the installation of the device changed the side of mastication in 35% of patients; in 25% of these patients there was a shift from unilateral to bilateral mastication. Regarding the painful symptomatology, pain remission was observed in 50% of patients and the other 50% still had pain (in 25% of them the pain shifted from bilateral to unilateral, in 10% the unilateral pain changed to bilateral pain, and in 15% the side of pain remained unchanged). It may be concluded that the therapy with occlusal splints is an important method in the remission of painful symptoms, capable of influencing the masticatory function and change the dynamic relations of occlusion.

## 008 Clinical evaluation of avulsed teeth – patient profile, etiological factors and presence of resorption

RODRIGUES, F.C.C. (Flávia Cássia Cabral Rodrigues flaviacassiacabral@hotmail.com); ROSCOE, M.G.; VALDIVIA, A.D.C.M.; SILVA, G.R.; NOVAIS, V.R.; SOARES, C.J.

The study of the etiology and consequences of dental avulsion is important in the development of preventive programs and treatment protocols. This study evaluated cases of dental avulsion treated in the Trauma Clinic of the Federal University of Uberlândia (FOUFU) according to the factors: age, gender, relation of the etiology with other traumas, avulsed tooth, number of involved teeth, and development of tooth resorption. The results indicated that the most prevalent age range was between 6 and 12 years old (66.7%), being 66.7% of male individuals. In this age group, the predominant etiologies were fall (41.7%) and bicycle accident (41.7%). In older age groups, bicycle accident presented the highest frequency (44.4%), followed by fall (27.8%), motorcycle accident (11.1%) and fights (16.6%). The most commonly involved teeth were the upper central incisors (77.8%), 88.9% of the cases involving only 1 tooth and 11.1% involving 2 teeth. Tooth resorption (33.3%) is associated with the delay between the moment of accident and the first-care treatment, as well as to patient withdrawal after the first-care treatment and the beginning of the endodontic therapy with calcium hydroxide. The prevalence of dental avulsion in male patients aged 6-12 years due to falls and bicycles accidents shows the possibility of reducing this event with use of preventive measures, such as the use of mouthguards. The education of the population in looking for immediate care and the knowledge of the professional in starting as soon as possible the endodontic therapy are decisive factors for treatment success.

## 009 Influence of cola drink type and dental biofilm on enamel erosion

ZAIDAN, F.C. (Flavia Zaidan Cardoso dos Santos favivinha_zcs@hotmail.com); HONÓRIO, H.M.; MAGALHÃES, A.C.; MACHADO, M.A.A.M.; BUZALAF, M.A.R.; RIOS, D.

This *in situ*/ex vivo study evaluated the influence of light and regular cola drink on human enamel wear and superficial demineralization in the presence and absence of dental biofilm. During 3 experimental 14-day crossover phases, 10 volunteers wore intraoral appliances containing human enamel blocks that were allocated to six groups: 1st phase- (RC) regular cola drink and (RCDP) regular cola drink with dental biofilm accumulation, 2nd phase- (LC) light cola drink and (LCDP) light cola drink with dental biofilm accumulation, 3rd phase- (SS) 8% sucrose solution (as contained in the regular cola drink) and (AS) 0.024% aspartame solution (as contained in the light cola drink), both with dental biofilm accumulation. Thus, in RC and LC, the specimens were fixed at the intraoral appliance surface level. In RCDP, LCDP, SS and AS, they were fixed 1.0 mm into the appliance level and covered by plastic meshes. The samples were demineralized extraorally 3 times daily for 5 minutes in the respective solutions. Enamel alterations were measured using profilometry and surface microhardness change (%SMHC). The data were tested by ANOVA and Tukey's test (p<0.05). Regarding enamel loss, RC (3.03 μm) showed significantly greater wear than the other groups, which did not differ significantly from each other (RCDP/0.02; LC/0.29; LCDP/0.03; SS/0.02; AS/0.04 μm). Regarding surface softening, the highest demineralization was observed for RC (-79.75) and LC (-64.10), which differed significantly between them and also from all the other groups, which, in turn, did not differ from each other (RCDP/-9.84; LCDP/-5.52; SS/-13.69; AS/-3.85). The light cola drink was less erosive than the regular one and the presence of dental biofilm reduced the erosive effect of both types of cola drink. Financial support: FAPESP (Proc. 2006/03874-8).

## 010 Dental caries and fluorosis in 13- and 14-year-old adolescents

IONTA, F.Q. (Franciny Querobim Ionta francinyionta@hotmail.com); GOYA, S.; SALES-PERES, A.C.; MOURA, P.G.; SALES-PERES, A.; SALES-PERES, S.H.C.

The purposes of this study were to determine the prevalence of dental caries and fluorosis in 721 adolescents aged 13 and 14 years old in Maringá, PR. Clinical examinations were done by a previously calibrated dentist according to the World Health Organization methodologies, using caries (DMFT index) and fluorosis (Dean index) indexes. Descriptive statistics was used and it was presented by means of relative and absolute frequencies. In the 13-year-old adolescents, the DMFT was 1.75 (m=1.54; f=2.01), 49.44% were free of caries and 18.89% presented some fluorosis level. In the 14-year-old adolescents, the DMFT index was 2.36 (m=2.09; f=2.55), 35.46% were caries free and 15.51% present some fluorosis level. The results showed that dental caries has declined and that fluorosis did not represent a public health problem in the studied city. Additionally there is a need to implement strategies that may guide actions and procedures towards individuals of major caries risk.

## 011 Direct composite veneer in an anterior tooth: a case report

FRANCISCONI, L.F. (Luciana Fávaro Francisconi luff@usp.br); SCAFFA, P.M.C.; SILVA, L.M. NAHSAN, F.P.S.; SAMPAIO, P.C.P.; FRANCISCONI, P.A.S.

Direct veneers are effective in replicating natural dentition when knowledge of tooth characteristics and principles of nature, art, and materials science are understood and applied. The fabrication of direct veneers requires skills to correctly reproduce the main features of tooth structures, since they are especially indicated for previous shape and/or color alterations. Overcoming these problems is not always easy, especially when only one of the anterior teeth is involved in the treatment plan. This case report describes a technique for fabrication of a direct composite resin veneer, addressing its advantages, limitations, and clinical procedures. A 30 year-old patient complained of dissatisfaction with the color and shape of the maxillary right central incisor, previously bleached. Among the treatment options, a direct composite veneer was considered. The preoperative visual color analysis was completed, and, silicon guides were obtained from the anterior teeth in order to provide a better visualization of tooth wear in the mesiodistal and cervico-incisal dimensions. Tooth #11 was prepared according to the shaper technique. After rubber dam isolation, acid etching and adhesive application, a layer of white opaque resin was used to mask the dark gray color of the dentin, and the different tooth structures and optical characteristics were reproduced using various resin shades. In a second section, finishing and polishing were accomplished and a cervical repair was done. Although indirect restorations have demonstrated greater longevity, composite materials can be predictably built up to provide realistic esthetics with acceptable durability and immediate patient satisfaction. The advantages of placing direct veneers are increasing and include immediate single-visit treatments, reduced cost, and conservative preparations. When properly indicated, direct composite restorations, including veneers, can be a viable treatment modality, as demonstrated in this case report.

## 012 Ranula: case report

CUNHA, F.J.P. (Frederico José Pessoa Cunha fred_522@hotmail.com); RODRIGUES, M.T.V.; GALVÃO, N.S.; PINTO, J.M.V.; ALEIXO, R.Q.; COSTA, M.R.S.N.

The salivary glands are susceptible to the development of several pathological processes. One of the most frequent salivary gland lesions is mucous or saliva retention inside the excretory duct or adjacent tissues, due to extravasation of mucous after trauma or obstruction of the salivary glands ducts. This lesion is termed mucocele, but when occurring in the oral floor it is called ranula. A 10-year-old Caucasian female, without relevant medical or dental history sought treatment complaining of “bullous lesion under the tongue” with nearly six months of evolution. A bluish hue oval swelling was observed in left sublingual gland region after clinical examination. These findings led to a presumptive diagnosis of ranula. The treatment consisted in lesion enucleation followed by removal of the affected sublingual gland and histopathological examination. The final diagnosis was mucous extravasation cyst (clinically compatible with ranula). Even considering other treatment options, the present case confirms better prognosis after lesion enucleation followed by surgical removal of the affected sublingual gland for complete resolution of this condition, minimizing lesion recurrence.

## 013 Success of endodontic treatment in immature teeth: case report

BARBÉRIO, G.S. (Gabriel Salles Barbério gasalles@gmail.com); MARQUES, V.R.; OROSCO, F.A.; GARCIA, R.B.; BRAMANTE, C.M.

Permanent teeth are considered as young teeth when the apex does not present apical dentin covered by cementum histologically and does not reaches Nolla's stage 10 radiographically, which means open apex. These young teeth exhibit characteristics such as thin and fragile root walls and apical opening larger than the root canal and therefore the endodontic treatment is not indicated for these cases. Some years ago, the endodontic treatment of immature teeth consisted in the adaptation of the filling material by means of apical surgeries with root-end amalgam restoration. These surgeries, however, have drawbacks such as being carried out in young people, reducing the crown-root ratio, possible failure in the amalgam adaptation to the canal walls, and high failure rate. Therefore, the current practice indicate apical seal with calcium hydroxide, which has a high success rate in endodontic treatment until proper anatomic conditions are obtained to perform an adequate and definitive obturation. This work reports a case of apexification obtained with calcium hydroxide and the whole operative technical sequence adopted in FOB-USP, in a 12-year-old patient who presented with pulp necrosis three teeth secondary to dental trauma with four years of follow up. In conclusion, the repair of endodontically treated immature teeth is characterized radiographically by the appearance of a radiopaque tissue, closing the apical opening or inducing the completion of root formation, and that calcium hydroxide remains the most commonly used and accepted material in the treatment of immature, although some researchers believe that apical closure can occur when the apical infection is controlled without the need for placing a material that induces mineralized tissue deposition.

## 014 Moebius syndrome: case report

OLIVEIRA, G.C. (Gabriela Cristina de Oliveira oliveira_gabi@yahoo.com.br); ROBERTO, A.F.B.; MARTA, S.N.

The Moebius Syndrome is a disease characterized by a total or partial facial paralysis. The eyes and the face movements to express emotions are compromised. Its etiology is not fully known and it is attributed to environmental factors such as the use of chemical inducers of uterine contractility (misoprostol), maternal hypotension with effect on fetal hemodynamics, trauma during pregnancy and hyperthermia on the pregnant woman. The nerves affected by the disease are the abducent and the facial, but anomalies in other pairs of cranial nerves are also often observed, particularly the glossopharyngeal and the hypoglossal. The main clinical feature of the syndrome is the “mask-like facial expression”, that is, the lack of facial expression in situations of sadness or joy. The most frequent limbic malformations are finger hypoplasia in several degrees, sometimes reaching adactilia, and the club foot (talipes equinovarus). Furthermore there can be alterations in the tongue, microstomia, micrognathia, convergent strabismus, high and narrow palate, and hearing problems. The treatment for the carrier of Moebius Syndrome should be conducted by a multiprofessional team comprising the different physical, cognitive, social and emotional needs of the patient. In this sense, this study describes the case of a patient with Moebius Syndrome highlighting the clinical and dental aspects as well as the dental treatment performed.

## 015 TNF-alpha and IL-10 modulate differentially coupled bone formation in experimental periodontitis in mice

GENNARO, G. (Gabriela Gennaro gabigennaro@hotmail.com); CLAUDINO, M.; SILVEIRA, E.M.; ASSIS, G.F.; TAGA, R.; GARLET, G.P.

In physiological conditions, bone resorption is followed by osteoblast-mediated bone formation in a process termed “coupling”. However, the mechanisms involved in the regulation of coupled bone formation under inflammatory bone loss conditions (such as periodontitis) remain unknown. The aim of this study was to compare the role of TNF-alpha and IL-10 in the control of coupled bone formation in experimental periodontitis induced by A. actinomycetemcomitans in IL-10 (IL-10KO), TNFp55 (TNFp55KO) knockout and control (WT) C57Bl/6 mice. It was evaluated expression of bone formation markers OCN (Osteocalcin), ALP (Alkaline phosphatase) and CBFA1 (Core binding factor alpha1) by RealTimePCR. When compared to WT mice, IL-10KO mice presented a significant decrease in CBFA-1, OCN, ALP levels, which occurred concomitantly with an increased bone resorption activity (higher levels of RANKL, cathepsin K and MMP-13 expression), resulting in a higher level of bone loss. Conversely, TNFp55KO strain presented a lower bone loss rate, possibly due by decreased expression of bone resorption markers and increased levels of CBFA-1, OCN and ALP. Then, the results demonstrate that IL-10 and TNF-β presented opposite roles in the modulation of coupled bone formation in inflammatory environments, being stimulate by IL-10 and inhibited by TNF-β.

## 016 Epidemiological evaluation of the periodontal status of patients with cleft lip and palate

NATALICIO, G.L. (Gabriela Leticia Natalicio gabinatalicio@gmail.com); CUNHA, M.J.S.; OLIVEIRA, P.G.F.P.; FIAMENGUI FILHO, J.F.; ALMEIDA, A.L.P.F.

In Dentistry, few studies have been conducted in Periodontics on individuals with cleft lip and palate, either addressing the prevalence, incidence, extension and severity of periodontal lesions or the treatment of these disorders in adults. This study analyzed the prevalence and severity of periodontal disease on 400 individuals with unilateral or bilateral complete cleft lip and palate, aged 15 to 50 years, attending the Hospital for Rehabilitation of Craniofacial Anomalies of the University of São Paulo, without any previous periodontal treatment. Clinical examination comprised measurements of probing depth, clinical attachment level, gingival index, plaque index and gingival recession. A total of 86.75% of patients presented probing depth < 3 mm. No sextant exhibited probing depth > 6 mm. There was statistically significant difference in probing depth according to age, types of cleft and sextant (p<0.001); 95.87% of teeth presented mean attachment level = 3 mm. The sextant with cleft did not present higher means of probing depth, clinical attachment level, plaque index and gingival index. There was no statistically significant difference between gender and the other variables. There was gingival bleeding and presence of biofilm in 99.69 and 97.40% of the sample, respectively, in both maxillary and mandibular teeth. The frequency and severity of gingival recessions were increased with age, affecting mainly the premolars and molars. The frequency of recession in incisors and canines was 10 times higher compared to the general population. The type of cleft was not an important factor influencing the prevalence of periodontal disease. Gender did not influence any of the clinical parameters in this sample. Age seems to be an important factor influencing the prevalence and severity of periodontal disease, for all aspects investigated. Periodontal disease in individuals with clefts, in the present study, occurred in a similar manner as observed in other populations. The presence of a cleft does not seem to increase the prevalence of the disease.

## 017 Black triangle elimination with periodontal plastic surgery and esthetic restorative procedures

OLIVEIRA, G.U. (Gabriela Ulian de Oliveira gabiulian@usp.br); PINTO, R.C.N.C.; ISHIKIRIAMA, S.K.; ISHIKIRIAMA, B.L.C.

Miller class IV recessions cause considerable esthetic, phonetic and functional complications. In such cases, the combination of surgical and restorative procedures may be required. This case report describes a variation of a surgical technique in which a coronally advanced flap (CAF) procedure was associated to a connective tissue pedicle graft rotated (CTPGR) from the palatal region to reconstruct the interdental papilla in adjacent Miller class IV recessions. In addition, restorative procedures were performed on both affected teeth (#6 and #7) to improve the esthetic appearance. A healthy 39-year-old woman presented with compromised esthetics between teeth #6 and #7. Clinical and radiographic examinations revealed two Miller class IV gingival recessions involving these teeth with recession depths of 3 mm and 4 mm, respectively. IN addition, a Nordland & Tarnow class III interdental tissue loss of 7 mm was observed. After 1-year follow-up, the results demonstrated physiological probing depth and clinical attachment level gain with no clinically evident signs of inflammation in the gingival margin. There was complete coverage of recession (tooth #6) with an increase in papillary volume, an improvement in the gingival zenith position, and considerable reduction of the black triangle. Within the limits of this case report, the combination of the coronally advanced flap surgery and a connective tissue pedicle graft, complemented by restorative procedures led to a satisfactory esthetic outcome and may be a viable approach for the treatment of Miller class III and IV recession-type defects.

## 018 Determination of rotational freedom between abutment and implant for analytical model

FERREIRA, G.T. (Gabriella Tavares Ferreira gabitfer@hotmail.com); SILVA-NETO, J.P.; CORÓ, V.; ARAÚJO, C.A.; DAVI, L.R.; NEVES, F.D.

The aim of this study was to validate analytical models comparing the theoretical with the experimental rotational freedom, between the implant and the abutment hexagons. The following external hexagon implants were used: Internal torque (IT), Branemark System MK III (NO) and conventional external hexagon (EH). The theoretical freedom was determined by means of an analytical model developed in the MATLAB software program, from mathematical expressions. The experimental rotational freedom was determined cording to manufacturers' testing. The results were subjected to the Student's t-test (p<0.05). The averages of the experimental and theoretical results were, respectively: IT 3.30 +- 0.17o and 3,34 +- 0.18o, NO 2.58 +- 0.35 and 2,81 +- 0.39o, EH 3,31 +- 0.41 and 3,62 +- 0.48o. No statistically significant difference could be found between the experimental and theoretical results for each type of implant evaluated. The relative error between the averages of theoretical and experimental rotational freedom was lesser than 10% for all implants analyzed. Therefore, the analytical model used in the MATLAB software is valid to determine the theoretical rotational freedom angle of each sample, with no need of making the measurements of rotational freedom angles in the experimental device.

## 019 Esthetic solution for darkened teeth with laminate composite veneers

GAMBARINI, C.T.A.G. (Cristiana Toledo de Arruda Gambarini cris_gambarini@yahoo.com.br); VERONEZI, M.C.; LEGRAMANDI, D.B.; BONELLI, A.C.C.; GOMES, C.C.; CARBALLEDA, G.S.

Over the past decades, the Restorative Dentistry specialty has been in evidence due to the increased search of patients for a \”perfect smile\” because current society consider it a synonymous of status, increasing people's self-esteem and confidence. On the other hand, it reinforces the responsibility of the professionals acting in this field due to the constant and fast appearance of new materials and techniques. Laminate veneers produce full or partial coverage of the buccal surface of esthetically compromised teeth with conservative materials and techniques. By the direct technique, laminate veneers can be performed by handpicked free, using composite resins with similar optical characteristics to those of the dental structures. This case report shows the fabrication of laminate composite veneers on severely darkened teeth 11 and 21 due to endodontic treatment and old and yellowish fillings. Excellent esthetic and functional outcomes were obtained, reestablishing the patient self-esteem.

## 020 Temporomandibular joint arthrocentesis

SANTOS, G.S. (George Soares Santos georgesoaress@gmail.com); PROVENZANO, N.; MARQUEZ, I.M.; ROCHA, F.S.; FURTADO, L.M.; ZANETTA-BARBOSA, D.

Temporomandibular dysfunction (TMD) is a generic term that refers to pathologies of the temporomandibular joint (TMJ). For the precise diagnosis of this disease, it is necessary a comprehensive anamnesis as well as physical and complementary exams. Therapeutic procedures, such as medication and interoclusal splints can also be of great importance in the differential diagnosis and treatment. . Each type of joint disorder needs a different approach that may range from medication to open surgery. Arthrocentesis is the washing of the upper compartment of the TMJ aiming at removing debris and inflammatory mediators. This low invasive procedure can be used either for diagnosis or as definitive treatment of several affections of the TMJs, such as: temporomandibular disk displacement with and without reduction, synovitis and arthrosis. The objectives of this work are to make a brief review of the literature about arthrocentesis and to describe a clinical case of an 18 year-old woman with complaint of open mouth reduction with 15 days of evolution. The patient reported having heard a click in the left TMJ joint during yawning and could not open her mouth since then. The initial maxillofacial clinical examination showed open mouth limitation (22 mm), absence of right lateral movements and mandibular deviation to the left in maximum opening. After the anamnesis, clinical exam and complementary exams, the hypothesis of anterior disk displacement without reduction was established. Interoclusal splint therapy was used with little success. TMJ arthrocentesis was then used and the patient improved maximum mouth opening to 44mm and recovery mandibular movements.

## 021 Isolated fracture of the zygomatic arch: Case report and discussion of the most important surgical managements

GIL, A.P.S. (Ariane Paredes de Sousa Gil aps.gil@hotmail.com); CARVALHO, A.C.G.S.; QUEIROZ, T.P.; MAGRO-FILHO, O.

The zygomatic complex fractures have a high incidence among the different kinds of facial trauma, and its occurrence is only lower than nasal fractures. However, its etiology and epidemiology depends on the social, economic and cultural factors of each evaluated region. The zygomatic arch projects itself laterally in the face, giving its transversal dimension. Its fracture may be associated to low intensity impacts because of the fragility of this structure. Clinically, the zygomatic arch fractures lead to reasonable facial asymmetries, limitation of mouth opening due to impaction of the mandibular coronoid process, as well as edema and pain and crackling on palpation. Radiographically, these fractures are observed in three points, modifying the convex morphology of this structure to a “V” shape, and are easily noted at the submentovertex radiograph. The aims of this study are report an isolated fracture of the zygomatic arch and discuss the various therapeutic managements that can be used in the surgical treatment of this type of fracture. A 26-year-old male had a sporting accident and was referred to the Araçatuba School of Dentistry complaining of “a hole in the face”. After clinical and radiographic examination, an isolated fracture of the right zygomatic arch was diagnosed. A surgical treatment was performed under local anesthesia, and a Barros hook was used for the reduction of the fracture. This technique permitted an anatomical reduction of the fracture and the patient was rehabilitated on his esthetic and function.

## 022 Increase of the keratinized gingival tissue width by the free gingival graft technique

ANOVAZZI, G. (Giovana Anovazzi giovanaanovazzi@hotmail.com); SOUTO, B.H.M.; OLIVEIRA, G.J.P.L.; SOUZA, J.A.C.; FONTANARI, L.A.; SAMPAIO, J.E.C.

It is known that the presence of keratinized tissue is not necessary for the maintenance of the stability of periodontal tissues. However, the presence of some local conditions, such as, thin gingival tissue, prominent root and gingival recession, combined with a reduced or missing amount of attached gingiva, may indicate a gingival augmentation procedure. Among the techniques proposed to achieve this increase, a free gingival graft procedure is one of the most predicable approaches for keratinized tissue augmentation, and the results remain stable for long periods of observation. The aim of this case report is to demonstrate the effectiveness of the free gingival graft technique in increasing the width of the keratinized gingival tissue. The patient had recessions and no attached gingiva associated with the lower incisors, which hindered her oral hygiene. Considering the limitations of this work, it may be concluded that the free gingival graft technique leads to an increase in the width of keratinized tissue.

## 023 Autogenous transplantation - mediate technique

GRACIO, A.C.M.M. (Ana Clara Maria Malta Gracio acgracio@hotmail.com); BARBIERI, A.A.; ALVES, M.G.O.; ALMEIDA, J.D.

The dental transplantation involves an autologous tooth implantation in a new alveolus. The objective of this study is to demonstrate the viability of the auto- transplantation of the 3rd molar tooth germ to the 2nd molar tooth place, by the mediate technique. The first session is used to prepare the alveolus and the transplantation is done in the second session. A 16-year-old Caucasian female patient with indication for extraction of tooth #37 was selected for this study. During the clinical and radiographic examinations, an extensive carious lesion involving the furcal region and periapical lesion were detected. After anesthetic blockage, incision and flap elevation, tooth #37 was sectioned and extracted, followed by curettage of the periapical lesion and preparation of the receptor alveolus. In the following session, the tooth #38 to be implanted was put in infraocclusion and stabilized with an X-suture; the region was cleaned and the patient medicated. After removing the suture, the following clinical features were observed: good bone insertion, lack of sensitivity and health periodontium. Although other treatment options are available, dental transplantation, when well indicated, can bring good clinical results, which makes it an excellent therapeutic option for oral rehabilitation in young patients.

## 024 Long-term durability of composite resin oral rehabilitation in a multidisciplinary treatment

PASQUINELLI, G.B.A. (Guilherme Bordini do Amaral Pasquinelli guibacana@hotmail.com); SOARES, C.J.; BRANCO, C.A.; QUAGLIATTO, P.S.; FONSECA, R.B.

The use of techniques for composite resin combined with esthetic and functional parameters provide greater chances for long-term effective treatments that deal with different areas in dentistry. The aim of this work is to present in a clinical case the principles and techniques of dental transformations with composite resin, relationships with periodontics and occlusion, and the results of 8 years of follow-up. A 46-year-old female patient complained of a diastema between teeth #11 and #12, pigmented restoration in tooth #12 and dissatisfaction in the position of maxillary teeth. The clinical examination showed generalized periodontal disease and posterior premature contact generating mandible deviation and excessive contact at palatal surface of tooth #12. Occlusal adjustment and periodontal treatment were done followed by restorative treatment with closing of the diastema and dental transformations. The multidisciplinary intervention resulted in periodontal health, good occlusal pattern with tooth alignment in addition to esthetic improvements. The treatment outcomes were followed up during 8 years, showing slight wear and satisfactory general conditions. The adhesive techniques with direct composite resin in dentistry allow expanding treatment opportunities and availability with excellent durability.

## 025 Effect of post length on the fracture resistance of endodontically treated teeth restored with custom cast post and core or prefabricated post and composite resin core

COSTA, G.C. (Guilherme Corbetta Costa guicorbetta@gmail.com); PEREIRA, J.R.; GHIZONI, J.S. VALLE, A.L.; OLIVEIRA, J.A.; ZOGHEIBE, L.V.

This study compared the fracture resistance of endodontically treated teeth restored with posts and cores with different post lengths covered by cast restorations. Sixty extracted intact canines were endodontically treated and randomly divided into 6 groups of 10 teeth each. Groups 1, 2 and 3 were restored with custom cast posts and cores with different combinations of post length (5.0 mm, 7.5 mm and 10 mm, respectively) and groups 4, 5 and 6 were restored with prefabricated post and composite resin core with different combinations of post length (5.0 mm, 7.5 mm and 10 mm, respectively). A compressive loading was applied at a 45-degree angle to the long axis of the tooth until failure. The two-way analysis of variance (á=.05) showed a statistically significant difference between the types of post (P<.001), among the different post lengths (P<.001) and showed a highly significant interaction between type of post and post length (P<.001). However, when the mean fracture forces for the groups were compared (Group 1 – 254.4 N; 2, 3, 4, 5, 6 groups– 331.7 N, 434.7 N, 405.4 N, 395.6 N and 393.8 N, respectively), no significant differences could be detected among the three groups restored with prefabricated posts and composite resin. This study showed that an increased post length in teeth restored with prefabricated posts does not significantly increase the fracture resistance of endodontically treated teeth. On the other hand, endodontically treated teeth restored with custom cast posts and cores showed a significant increase in fracture resistance when the post length was increased.

## 026 Histomorphometric analysis of the periodontium of rat molars after furcal perforations filled with different materials

SILVA, G.F. (Guilherme Ferreira da Silva gferreiras@hotmail.com); TANOMARU-FILHO, M.; GUERREIRO-TANOMARU, J.M.; SASSO-CERRI, E.; CERRI, P.S.

The success in the treatment of root perforations depends on the material used to fill them. Thus, the aim of the present study was to evaluate the biological response of three sealing materials at furcal perforation in rat molars: White MTA-Angelus (MTA), Endo-C.P.M.-Sealer (CPM) and zinc oxide and eugenol cement (ZOE). After 7, 15, 30 and 60 days, the animals were killed and the specimens were prepared to be stained with hematoxylin and eosin, Masson's trichrome and subjected to TRAP (Tartrate Resistant Acid Phosphatase) reaction. The morphometric analysis was performed considering three parameters: periodontal ligament thickness (PL), number of inflammatory cells (IC) and number of TRAP-positive osteoclasts in the alveolar bone surface. The experimental groups showed, in all periods, significant increase (p=0.05) in the PL thickness in comparison to the control group; this thickness being more accentuated in the ZOE group. The periodontal space in the CPM group was thinner in all experimental periods in comparison to MTA and ZOE groups, except for the period of 60 days; in this period, statistically significant differences in the periodontal space were not detected between CPM and MTA groups. At 7 days, a large number of IC was verified in all groups, especially in ZOE; from 7 to 60 days, the number of IC decreased gradually. Quantitative analysis revealed a significant increase in the number of TRAP-positive osteoclasts in the ZOE group, in all studied periods, in comparison to the other groups. In CPM and MTA groups, the number of osteoclasts was not significantly different in the periods of 15, 30 and 60 days. Moreover, in the 60-day period, no significant differences were observed in the number of osteoclasts among CPM, MTA and control group. Therefore, the present results indicate that MTA-based materials seem to be more biocompatible than ZOE.

## 027 Viral infection in an immunosuppressed patient under chemotherapy

SPAGNOL, G. (Guilherme Spagnol guilherme.spagnol@uol.com.br); FREITAS, R.R.; SANTOS, P.S.S.; NAPOLES, B.B.

Patients subjected to chemotherapy become immunosuppressed and are therefore susceptible to infection. Bacterial, fungal and viral infections, such as cytomegalovirus, herpes simplex, varicella-zoster and Epstein-Barr, are associated with high morbidity among such patients. The viruses have similar clinical manifestations in the oral mucosa. The aim of this case report is to show the importance of the diagnosis and treatment of oral herpetic infection in immunosuppressed patients. A 20-month-old female patient with neuroblastoma, who had been receiving chemotherapy treatment for six months, was hospitalized one day after a course of chemotherapy. She had febrile peaks (39 degrees) and leucopenia (leukocytes 0.200/mm^3^) on the day of admission. There were painful lesions on her tongue, gums and lips, with a vesicular-bullous center of approximately 0.5 cm and erythematosus edges and she mentioned having dysphagia. During her hospital stay, the patient received antibiotic therapy with the following drugs: ceftazidim, vancomycin, oxacillin, crystalline penicillin G and metronidazole but the febrile peaks did not diminish. Scarification of the lesion was carried out and the Tzanck method showed the presence of cells with viral inclusion on the tenth day of hospitalization, which led to the diagnosis of recurrent herpetic stomatitis. The patient was treated with a daily intravenous dose of 450 mg of aciclovir and a 3 times a day topical aqueous 0.12% chlorhexidine to prevent secondary infection of the lesions. She had just one febrile peak 24 hours after starting treatment with acyclovir and the fever ended after 48 hours. Within 3 days, the patient started to feed normally, with general clinical recovery. It may be concluded that the diagnosis, treatment and prevention of infections in immunocompromised patients are of great importance because they may have atypical presentations, making them difficult to diagnose.

## 028 Evaluation of root canal fillings using five types of sealers

ANDRADE, G.H. (Gustavo Henrique Barbosa de Andrade ghb_andrade@hotmail.com); BRAMANTE, C.M.; BERNARDES, R.A.; MORAES, I.G.; BERNARDINELI, N.; GARCIA, R.B.

The aim of this study was to evaluate the sealing ability of root canal fillings performed with five root canal sealers evaluated by the fluid filtration model. Sixty-four human single-rooted premolars were used. After preparing the coronal access to the pulp chamber, the diameter of the apical foramen was standardized with a #25 K-file. The cleaning and shaping of the root canals was performed using the nickel-titanium Profile .04 and .06 instruments until the 40.06 ProFile instrument reached the working length. The root canals were irrigated using 1% sodium hypochlorite. The teeth were randomly divided into 5 experimental groups (n = 12): AH Plus (G1), MBP (G2), Acroseal (G3), Sealapex (4), MTA sealer(G5) and two controls (positive and negative; n = 2). The sealing ability was measured by using the fluid filtration model. Four measurements, one every 2 minutes for 8 minutes were analyzed after 15, 30 and 60 days. The fluid filtration model showed that Sealapex, Acroseal and MTA sealer showed an increment in the leakage in the period from 15 to 60 days. AH Plus and MTA sealer showed a decrease in the leakage pattern after 30 days. There was no significant difference in the sealing ability among the sealers except for Acroseal in the 30-and 60-day periods and Sealapex in the 60-day period. AH plus and MBP sealers had the highest sealing ability.

## 029 Bite marks: a case report

MAROTE, I.A.A. (Isa Azevedo de Almeida Marote i.marote@hotmail.com); NARESSI, S.C.M.; BARBIERI, A.A.; QUELUZ, D.P.; SCHMIDT, C.M.; PREZA, A.O.

The work of forensic dentistry experts at the Forensic Medicine Institutes has been increasingly recognized by the authorities due to their role in human identification in live individuals, bones and corpses in an advanced stage of decomposition; aid at anthropology departments; and aid in the identification of suspects and body injury examinations by means of the study and analysis of teeth, dental events, bite marks and facial bones. This paper reports the case of a family that had been victim of drowning due to a flood occurred in the city of Cuiabá-MT in the beginning of 2001. The victims were brought to the Forensic Medicine Institutes of Cuiabá/MT to be subjected to necroscopic examinations, including a dental examination. During the examination of the bodies, it was found that one child had a bite mark on the neck. In order to identify the cause of the injury, the study cast models were obtained from the children's parents. The forensic dentistry expert used a caliper to make the following measurements: canine to canine, mesiodistal distance of anterior teeth and size of dental arches in the victims, in the models and also in the injury found in the child. The models were further superpositioned directly on the injury site. Since this injury did not cause the child's death, more detailed examinations were not performed because the purpose of the analysis was only to find who had produced it. After the analysis, it was found out that the injury had been produced in life, had characteristics of having been produced recently (the same day) and the comparative analysis of injury and models of the dental arches of the parents led to the conclusion that the bite mark had been produced by the child's father in an attempt to save her, according to reports from witnesses.

## 030 Ccr5+ cells in experimental periodontal disease: modulation of pro- and antiinflammatory cytokine expression

NUNES, I.S. (Isabela Sousa Nunes isanunes74@yahoo.com.br); CLAUDINO, M.; RAIUMUNDO, F.M.; COLAVITE, P.; REPEKE, C.E.; GARLET, G.P.

Inflammatory and immune response to periodontopathogens result in inflammatory alveolar bone resorption, in an environment characterized by high levels of chemokines and their receptors, such as CCR5 receptor. However, mechanisms linking CCR5+ cells to the immunoregulation of periodontal disease remain unknown. The aims of this study were to evaluate the modulation of proinflammatory (TNF-alpha and IL-1beta) and antinflammatory (IL-10) cytokines expression in C57Bl/6 (WT) and CCR5KO mice after A. actinomycetemcomitans oral inoculation, and to correlate it with periodontal disease severity. After 0, 7, 15, 30 and 60 days of infection, alveolar bone loss and levels of TNF-alpha, IL-1beta and IL-10 expression (by RealTimePCR) were evaluated. CCR5KO mice presented a significant reduction (p<0.001) of bone loss after 30 days of inoculation. It was also verified that CCR5KO mice showed a significant reduction in TNF-alpha and IL-1beta expression (p<0.001), while only a trend of increased IL-10 expression (p>0.05) was observed. RANKL levels were reduced (p<0.01) in CCR5KO mice, possibly due by reduced levels of TNF-alpha and IL-1beta; while OPG levels were discreetly increased (p>0.05). Our results suggest that CCR5+ cells present a proinflammatory role in experimental periodontitis, regulating TNF-alpha, IL-1beta and RANKL levels.

## 031 Role of mast cells in the release of IFN-gama, IL-4, CCL5/RANTES, CXCL1/KC and XCL1/Lymphotactin in diabetic mice with periodontal disease

FREIRE, I.R. (Isabelle Rodrigues Freire isabellefreire@ig.com.br); SALZEDAS, L.M.P.; OLIVEIRA, S.H.P.

Previously, we observed that mice with diabetes mellitus (DM) subjected to periodontal disease (PD), demonstrated an increase in bone resorption when compared to normoglycemic mice. Thus, the purpose of the present study was to evaluate the role of mast cells (MAST) in the production of cytokines and chemokines induced by PD in mice with DM. Mice were pretreated with a single dose of streptozotocin (STZ) for induction of DM. To evaluate the role of MAST in PD, the mice were depleted of MAST by pretreatment with compound 48/80 (48/80). Subsequently, PD was induced using a ligature around the homologous first molars. Neutrophils (NE) recruited into the gingival tissue were evaluated by the production of myeloperoxidase enzyme (MPO), and the levels of IFN-gama, IL-4, RANTES/CCL5, KC/CXCL1 and Lymphotactin/XCL1 produced in tissue were evaluated by Enzyme-Linked Immunosorbent Assay (ELISA). It was observed the MPO levels were higher in diabetic and normoglycemic mice 14 days after the induction of PD. It was observed a partial reduction of MPO levels in diabetic mice with PD treated with 48/80. The level of IFNgama, IL-4, RANTES/CCL5, KC/CXCL1 and Lymphotactin/XCL1 was observed in diabetic mice independently of induction of PD after 7 or 14 days. In conclusion, DM increased the neutrophil recruitment in the PD mice. The MAST depletion decreased the recruitment of NE and it induced the production of high levels of IFN-gama, IL-4, RANTES/CCL5, KC/CXCL1 and Lymphotactin/XCL1, independently of induction of PD. Taken together, our results suggest that MAST may have a dual role on the PD under diabetic conditions.

## *In situ* evaluation of the effectiveness of low concentration fluoride dentifrices supplemented with calcium and phosphate

AMARAL, J.G. (Jackeline Gallo do Amaral jackelineamaral@gmail.com); DELBEM, A.C.B.; SASSAKI, K.T.; MARTINHON, C.C.R.

The aim of this study was to evaluate the effectiveness of low concentration fluoride dentifrices supplemented with calcium and phosphate on the demineralization of enamel and the dental biofilm formed *in situ*. The study was conducted in four experimental phases of 7 days, with 7-day intervals. Ten volunteers wore palatal appliances containing 4 bovine enamel blocks for each experimental phase. Cariogenic challenge was produced with 30% sucrose solution, 6 times per day. The groups were defined as follows: negative control (Placebo); 500 μg F/g dentifrice; 500 μg F/g with Ca dentifrice; positive control: 1100 μg F/g dentifrice (Crest®). Enamel alterations were evaluated by the percentage of surface microhardness change (%SMHC). Fluoride measurement was performed using an ion-specific electrode. The amount of calcium and phosphate was analyzed by testing the colorimetric spectrophotometer. The statistical analysis showed that the placebo had higher percentage of mineral loss than the 500 μg F/g dentifrice. The 500 μg F/g with Ca and 1100 μg F/g dentifrices showed similar minerals losses and lower the other groups (Analysis of Variance and Tukey test, p<0.05). The 1100 μg F/g dentifrice had higher concentration of fluoride, calcium and phosphate on the biofilm compared to others experimentals dentifrices, while the placebo dentifrice observed the lowest concentration of these ions (Kruskal-Wallis, p <0.05). Based on this study, the low concentration fluoride dentifrices supplemented with calcium and phosphate showed similar results to the standard, being able to reduce the demineralization of bovine enamel under high cariogenic challenge.

## 033 Hypernasality after orthognathic surgery: effect of pharyngeal flap surgery on correction of velopharyngeal insufficiency

ZANOLLA, J. (Jaine Zanolla jaine.zanolla@usp.br); SAMPAIO-TEIXEIRA, A.C.M.; YAMASHITA, R.P.; FUKUSHIRO, A.P.; TRINDADE, I.E.K.

Pharyngeal flap surgery (PFS) is routinely used for the correction of velopharyngeal insufficiency (VPI) in patients with cleft palate, based on the mechanical obstruction caused by the flap, which links the soft palate to the posterior pharyngeal wall, while two lateral holes are left for nose breathing. On the other hand, orthognathic surgery with maxillary advancement in this population has the potential to impair velopharyngeal closure, and cause hypernasality as a consequence. The purpose of this report is to present a clinical case of a patient subjected to orthognatic surgery followed by PFS, whose effects on speech resonance were evaluated by means of nasometry, a technique that allows the estimation of nasality. The patient had a surgically repaired unilateral cleft lip and palate. He was subjected to orthognatic surgery with maxillary advancement at 16 years of age and to PFS 9 months later. Nasometry was performed at the pre-and postoperative periods of both procedures during the reading of a set of oral sentences (OS) and nasal sentences (NS). A questionnaire was applied to assess respiratory symptoms. Nasalance scores obtained before orthognatic surgery corresponded to 37% (NS) and 17% (OS), indicating the presence of hyponasality without hypernasality The patient reported mouth breathing and left nasal obstruction. After orthognatic surgery (7 months), nasalance scores changed to 61% (NS) and 46% (OS), suggesting hyponasality elimination, with improvement of nasal breathing, and the development of iatrogenic hypernasality. After PFS (1 year and 3 months), scores corresponded to 49% (NS) and 15% (OS) demonstrating adequate oronasal resonance without respiratory complaints. The findings show that pharyngeal flap surgery is an effective option for the surgical treatment of VPI induced by orthognathic surgery with maxillary advancement.

## 034 Parameters for esthetic success in periodontics-restorative dentistry relationship

MIOTTO, J.C. (Janaína Castilho Miotto janaina_miotto@hotmail.com); OLIANI, F.L.; BRANCO, C.A.; BUSO, A.M.; QUAGLIATTO, P.S.; FONSECA, R.B.

Erosive lesions with dentin exposure when associated with color and shape alterations in anterior teeth result in unfavorable esthetics. The correct association of surgical-esthetic periodontital treataments and dental transformation with composite resin is an effective alternative for the resolution of these cases. The aim of this work is to show by a clinical case, all stages of an anterior esthetic rehabilitation by means of the interaction between restorative procedures and periodontal surgery. Male patient, JMA, 46 years old, presented subgingival erosive lesions mainly at buccal surfaces of maxillary teeth, discolored teeth (yellow) and esthetic disharmony (diastemata and uncohesive dental). The lesions were exposed by periodontal surgery, increasing clinical crown and then provisionally restored with glass ionomer cement. The teeth were subjected to 2 sessions of bleaching with 35% carbamide peroxide and, after 21 days, closing the diastemata and dental transformations were performed with adhesive techniques and direct composite resin. After 24 hours, the restorations were polished with diamond pastes (medium and fine granulations) on rubber cups, and the final gloss was done with polishing brushes. The use of the above-mentioned techniques resulted in periodontal health and esthetic-functional improvement of the case. The Periodontics-Restorative Dentistry association by means of correct planning and rigorous esthetic perfection allows excellent outcomes, making it possible to maintain the health of dental and periodontal tissues.

## 035 Clinical approach of ankyloglossia in babies: report of two cases

SILVA, J.Z.(Janaína Zavitoski Silva zavitoski_foa@yahoo.com.br); FARIA, M.D.; CUNHA, R.F.

Ankyloglossia is a developmental anomaly of the tongue characterized by a short lingual frenum, resulting in restricted movement of the tongue. Its etiology is undefined and there is no gender preference. Few studies are available in the literature and the diagnosis and management of ankyloglossia in infants remain controversial. We report two cases of infants subjected to lingual frenectomy, emphasizing the management of ankyloglossia and its implications in breast-feeding.

## 036 Characterization of patients with oral cancer in the northwestern region of Paraná

UCHIMURA, J.Y.T. (Joana Yumi Teruya Uchimura nuchimura@pop.com.br); PAVAN, A.J.; FERREIRA, J.O.H.; VENTRINI, V.C.

The objective of this study was to identify the epidemiological characteristics of patients with oral cancer in the northwestern region of Paraná, and the environmental and behavioral risk factors. This was a cross-sectional, descriptive, analytical study with secondary data obtained between January and December 2008 from the charts of all patients with oral cancer at the Center of Oncology and Radiotherapy Sant'Ana, in the city of Maringá-PR. From the total of 480 records, 49 patients had oral cancer. Data were collected in a questionnaire containing identification, place of residence, classification of cancer, presence of nodules, signs and symptoms before and after treatment. After analysis, it was found that exposure to sun was related to the type of jobs and sites of occurrence in the oral region. Data were entered to Excel and analyzed with Statistica 7.1 software. From the total population, 89.8% were Caucasian, 69.4% male, and the most prevalent age range was from 50 to 60 years (40.8%). The profession of farmer made a percentage of 28.6%, followed by housekeepers with 16.3%. The TNM (tumor, node, metastasis) staging system, classification of malignant tumors, had 6.1% T0; 8.1% T1, 26.5% T2, 12.4% T3, 34.7% T4, 36.7% N0, 26.5% N1, 18.4% N2, 6.1% N3, 85.7% M0, and no case of M1. The pathological findings showed 93.9% of mucoepidermoid carcinoma, being 10.2% in the tonsils, 26.5% on palate, 26.5% in the tongue, 10.2% in the floor of the mouth, and 10,0% in the retromolar region. It was observed that 69.4% of patients smoked, and 32.7% of them for 30 to 50 years. It was observed that 28.6% smoked 4 to 6 packs/day and 8.2% of patients over 6 packs/day. It was observed that 38.8% of the studied population were alcoholics. This study allowed concluding that there is a urgent need of effective measures, capable of promoting, the reduction of the risk of becoming sick and the prioritization of actions to control the disease.

## 037 The use of newly forming bone graft in the coverage of denuded root surface: a new surgical technique of periodontal plastic surgery

BARROS, J.P.C. (João Paulo Corrêa Barros joaof11@usp.br); FERRAZ, B.F.R.; SANT'ANA, A.C.P.; PASSANEZI, E.; REZENDE, M.L.R.; GREGHI, S.L.A.

The coverage of denuded root surfaces is a challenge in periodontal therapy, especially when attachment loss is > 5mm. The currently used therapies show variable rates of coverage, with poorer results in large recession areas. The aim of this study was to evaluate the effects of a newly forming bone graft as an alternative therapy in large recession areas, usually treated by connective sub-epithelial graft. A surgical alveolus with dimensions corresponding of those of tooth extraction alveolus was set and a collagen membrane was placed above it. After 25 days, the area was reopened and the newly forming bone was collected and grafted in a Miller's Class II dehiscence defect with 5 mm of extension from the cementoenamel junction to the marginal gingiva in a mandibular left second premolar. In the surgery, the root surface was treated by scaling and acid conditioning, and the graft was associated with a coronal sliding flap. The clinical parameters evaluated pre- and postoperatively were probing depth, clinical attachment level, marginal gingiva recession, bleeding on probing, plaque index and amount of keratinized tissue. The follow-up periods of 30 days, 3, 6 months, suggest reduction of clinical probing depth and marginal gingiva recession, and increase of clinical attachment level.

## 038 Unconventional rehabilitation to single tooth loss – 10 years of follow-up

SILVA, J.P.L. (João Paulo Lyra e Silva joaodf22@hotmail.com); SILVA-NETO, J.P.; DANTAS, L.C.M.; CARNEIRO, T.A.P.N.; NEVES, F.D.

The aim of this case report is to present an unconventional way of rehabilitation to single tooth loss, with 10 years of follow-up. A. 18-year-old patient had the central incisor (#11) fractured at middle root third with avulsion of coronal portion after trauma to the anterior facial area. The treatment of choice was the extraction of the root remnant and implant installation in the same session (3i Self-Tap® - 18 x 3.75mm). GingiSCULPT - 3i® abutment, supplied in two pieces for single-session surgeries, was used to maintain the gingival contour; after abutment installation a provisory prosthesis was fabricated joined to the adjacent teeth with a fiberglass bar. After the osseointegration period, the AurAdapt abutment (Nobel Biocare®) was selected for further fabrication of the metallic framework, which received ceramic application for complete metal covering. At the same time, the natural tooth crown had its radicular portion worn out and the internal contents removed, leaving only enamel. Before the ceramic burning, the crown was positioned over the abutment, molding the internal portion on ceramic, still in the dough stage. After abutment personalization, the internal portion of the crown was relined with photoactivated composite resin to increase the adaptation to the abutment. Finally, the abutment was screwed to the implant and the crown was cemented over the abutment with dual resin luting cement. The use of this technique showed esthetic advantages with the preservation of the natural characteristics and disadvantages related to complex procedures and the future caries possibility. Over 10 years follow-up, the treatment was proven effective, preserving functional characteristics; however slight esthetic alterations were observed over time, though without compromising the patient's satisfaction.

## 039 Effect of propolis gel on the reduction of dentin permeability, *in vitro*

REINATO, J.V.D. (João Victor Donazan Reinato jvreinato@yahoo.com.br); CARVALHO, F.L.N.; MARSICANO, J.A.; MATTOS, M.C.; PEREIRA, J.C.; SALES-PERES, S.H.C.

The aim of this study was to evaluate the effect of two propolis gels on reducing the hydraulic conductance of dentin, *in vitro*. The methodology used for the measurement of hydraulic conductance of dentin in the present study was based on the model proposed in literature. Thirty-six 1-mm-thick dentin discs obtained from human extracted third molars, were divided into 4 groups of nine specimens each. The groups corresponded to the following experimental materials: GI-10% propolis gel, pH 4.1; GII-30% propolis gel; GIII- 3% potassium oxalate gel, pH 4,1; and GIV-1.23% fluoride gel, pH 4.1, applied to the dentin under the following surface conditions: after 37% phosphoric acid etching and before 6% citric acid etching. Data were analyzed statistically by two-way ANOVA and the Tukey's test at 5% significance level. The results showed no significant differences among GI, GII, GIII and GIV in any tested conditions for reducing dentin permeability. However, the active agents produced the smallest reduction in hydraulic conductance when compared to the presence of smear layer (p<0.05). The results suggest that the effect of both 10% and 30% propolis gels was similar to that of Oxa-gel in reducing dentin permeability. Further ex-vivo and *in vivo* studies must be conducted to clarify the real action of propolis in dentin permeability.

## 040 Comparison of nickel-titanium and stainless steel files in the preparation of root canals

BAGATELI, J.C.E. (Jocilene Cristina Evangelista Bagateli jobagateli@hotmail.com); GABANA, M.L.; HIDALGO, M.M.

This study analyzed, comparatively by a literature review, the characteristics of the conventional stainless steel and nickel-titanium files, since these materials have been described during the last years as having good properties in the preparation of root canals. A specific questionnaire applied to endodontists was used to evaluate the practicability of using these instruments, and their importance in dental practice. The results showed that nickel-titanium files: cause less transportation of the canal, resulting in a higher percentage of round final shape of the transversal cross-sectional canal preparation; present less tendency to clockwise direction failure, which the direction most coomonly used in instrumentation techniques; permit similar time of instrumentation compared to stainless steel files, since the period used to pre-curve proceedure would not be counted; and remove the smallest amounts of dentin when compared to the stainless steel files. In conclusion, an association between both files to the instrumentation of the curved root canals would be the best choice, using the potential of each instrument in order to reduce its disadvantages.

## 041 Photoelastic analysis of stress distribution on platform switching implants

SANTIAGO-JR, J.F. (Joel Ferreira Santiago Junior - joeljr@usp.br); PELLIZZER, E.P.; FÁLCON-ANTENUCCI, R.M.; CARVALHO, P.S.P.; VERRI, F.R.; ALMEIDA, D.A.F.

The aim of this study was to evaluate the stress distribution on latform switching implants by means of a photoelastic analysis. Three models were made of photoelastic resin PL-2 (Vishay Measurements Group, Inc Raleigh, NC, USA), with one implant (Conexão, São Paulo, SP, Brazil) with different diameters (3.75 and 5.00 mm), and a screwed crown, simulating a mandibular molar with abutments of different diameters (4.1 and 5.00 mm). Model A - Platform Ö5.00 mm/abutment 4.1 mm (Platform Switching), Model B - Platform Ö3.75 mm/abutment 4.1 mm (regular diameter/conventional) and Model C - Platform Ö5.00 mm/abutment 5.00 mm (wide diameter/conventional). The prostheses were standardized and made of NiCr alloy. In order to visualize the stress fringes, a circular polariscope was used, and axial loads of 100 N were applied by means of a Universal Test Machine (EMIC-DL 3000). The results were photographed and analyzed qualitatively in the image-editing program (Adobe Photoshop CS3). A greater intensity of stresses was observed in Model B (Platform Ö3.75 mm/abutment 4.1 mm), with stresses concentrated around the body of the implant and at its apex. In models A (Platform Ö5.00 mm/abutment 4.1 mm - Platform Switching) and C (Platform Ö5.00 mm/abutment 5.00 mm), the fringe distribution patterns were similar, as the stress was concentrated at the level of the implant apex. In Model A, the stress concentration was more centralized along the axis and was less intense around the implant body. It was concluded that: 1) Model B presented the highest concentration of stress. 2) Model A and Model C presented similar stress distribution.

## 042 Occurrence of opportunistic bacteria in the oral cavity of female patients attending a detoxification program for drug dependents

SANGALLI, J. (Jorgiana Sangalli jorgianasangalli@hotmail.com); CIESIELSKI, F. I. N.; LINS, S.A.; GAETTI-JARDIM JR, E.

FAPESP 2007/54851-3

The consumption of drugs has become a worldwide public health problem. The consequences of the abuse of licit or illicit drugs promote profound social, economic and, oral and systemic health changes. In these circumstances, the oral cavity may become a reservoir of opportunistic microorganisms acquired due to the environment in which drug consumption occurs and by the nature of human contacts under these conditions. Therefore, this study assessed the occurrence of opportunistic microorganisms in the oral microbiota of 50 female patients (compared to a control group) aged 18 to 55 years (mean age = 29.88 ± 9.3 years) attending a detoxification program for drug dependents in the city of Santa Fé do Sul, and in a control group of non-dependent females, with similar age range and periodontal conditions. In the group of drug addicter, 19 had generalized gingivitis, 12 had chronic periodontitis, 13 were periodontally healthy, and 6 wore complete dentures. Samples of saliva and sub and supragengival biofilm were collected and the presence of microorganisms of Enterobacteriaceae and Pseudomonadaceae families, as well as *Enterococcus sp., E faecalis, E. faecium, Helicobacter sp. and H. pylori*, was assessed by amplification of DNA by PCR or nested PCR, using specific primers for each infectious agent. Comparisons between clinical parameters and microbiological data were performed by the Kruskal-Wallis, and dichotomous variables were analyzed by the Chi-Square and Mann-Whitney non-parametric tests. Results showed significant differences between groups with respect to microorganisms mainly in relation to the occurrence of *Enterobacteriaceae, Helicobacter sp*. and *H. pylori*, which were much more frequent in drug dependent patients, irrespective of their periodontal conditions, evidencing prevalence from two to four times higher than in the control group.

## 043 Bariatric patients and their oral conditions

MARSICANO, J.A. (Juliane Avansini Marsicano juavansini@yahoo.com.br); SALES-PERES, A.; LAURIS, J.R.P.; CENEVIVA, R.; SALES-PERES, S.H.C.

The prevalence and incidence of obesity have become alarming in adults and children. In many cases, conventional and less invasive treatments for obesity do not produce good results and the bariatric surgery is indicated. The aim of this study was to identify the prevalence of periodontal diseases, dental caries and dental wear and saliva flow in 52 patients who had undergone bariatric surgery. The Community Periodontal Index (CPI), Dental Caries index (DMFT), the Dental Wear Index (TWI) were used to evaluate oral conditions. The oral exam was performed 16.9±20.7 months after the bariatric surgery. The mean age of the group was 39.6±9.6 and 75.00% of them were female. The patients had CPI of 3.05±0.84, and 88.46% of them had at least one reference tooth with periodontal pocket depth between 4-6 mm. The DMFT was 16.11±5.19; all patients presented history of dental caries and 71.20% presented the component “decay” (D). All patients presented at least one tooth with dental wear, and 78.80% presented at least one tooth with dentin wear. The saliva flow was 0.64±0.47 mL/min and 75.00% of the patients had hyposalivation. It may be concluded that the bariatric patients need attention in oral health and new research must be carried out for sedimentation of the scientific evidence in relation to the oral conditions of the patients undergoing bariatric surgery.

## 044 Increase of vestibule depth and keratinized tissue width with acellular dermal matrix graft in an area of gingival recession

SOLDATI, K.R. (Kahena Rodrigues Soldati ksoldati@hotmail.com); REINO, D.M.; NOVAES JR, A.B.; PALIOTO, D.B.; MAIA, L.P.

Many techniques have been used in an attempt to achieve root coverage. The acellular dermal matrix (ADM) has come as an alternative to the autogenous grafts and has been proven advantageous in increasing the keratinized gingival tissue width. One of the agents that can influence the treatment success of gingival retraction in mandibular anterior teeth is the vestibule depth, which hinders the hygiene and exerts a tensile action on the gingival margin. A case of gingival recession on the region of tooth #31 demonstrates the ADM technique application to increase the vestibule depth and the keratinized tissue width. A 20-year-old female patient came to the Periodontal Clinic of FORP – USP, complaining of the esthetic exposure due to a 7-mm gingival recession in the buccal face of tooth#31, in which root coverage was tried without success. After oral hygiene instructions, supra-gingival scaling and prophylaxis, a sliding flap was elevated. This procedure was chosen because the tooth had buccal tipping tending to the mesial, which resulted in an adequate width of gingival tissue in the distal area. However, flap necrosis occurred, probably due to the amplitude of the area to be covered, poor tooth position, and shallow vestibule. After 1 month of weekly biofilm control, a new surgical procedure was chosen to increase vestibule depth and keratinized tissue width. For this, ADM graft was done from teeth #33 to #43. Four months later, it was observed good healing, and increase of the keratinized tissue height and vestibule depth. In a later moment, a new surgical procedure for root coverage will be necessary. ADM may be useful to increase the keratinized tissue width and the vestibule depth, producing good esthetic and functional results.

## 045 Influence of mechanical cycling on the impact strength of denture base acrylic and direct reline resins

ALTIERI, K.T. (Karen Tereza Altieri karenaltr@gmail.com); ZAMPERINI, C.A.; PEREZ, L.E.C.; MACHADO, A.L.; VERGANI, C.E.; PAVARINA, A.C.

Autopolymerizing reline resins used to improve the fit of dentures to adjacent tissue directly in oral cavity must ideally ensure impact strength to relined denture. The purpose of this study was to evaluate comparatively, using the Charpy impact test (0.5 J), the impact strength of a denture base acrylic resin (Lucitone-L), two direct reline resins (New Truliner-NT and Kooliner-K) and combinations of them. Twenty samples (60X6X4 mm) of each material (L; NT; K) were made and tested intact. Samples of L (60X6X2 mm) were also made and relined with L (L/L; n=20), with NT (L/NT; n=20) and with K (L/K, n=20). Before testing, half of the samples obtained were subjected to mechanical cycling (5 Hz; 10.000 cycles). V-notches were machined at the midpoint of the length of all specimens. The analysis of the results (kJ/m2) by the Kruskal-Wallis test (á=0.01) showed that the mechanically cycled samples (L: 1.57, NT: 0.62, K: 1.03) presented similar means compared to non-cycled samples (L: 1.55, NT: 0.72, K: 1.16), with higher values for the resin L, followed by resins K and NT. Similar results were observed for the relined samples. No statistically significant differences were found among the mechanically cycled (L/L: 1.56, L/NT: 0.72; L/K: 3.91) and the non-cycled (L/L: 1.52, L/NT: 0.74; L/K: 5.15) samples, with higher values for L/K, followed by L/L and NT. The mean value of the impact strength of L/L was similar to that of L, while L/K had higher value and L/NT had lower. In conclusion, mechanical cycling not have a significant effect on the impact strength of the resins evaluated. Under the two tested conditions, the material used in the relining influenced the impact strength, in the following decreasing order: L/K, L/L and L/N.

## 046 Evaluation of the harmful effects of tobacco in osseointegration

RODRIGUES, K. (Keila Rodrigues odonto_keila@hotmail.com); FERRARI, W.; BONOTTO, A.P.; SUKEKAVA, F.; ARAUJO, M.G.; ARRUDA, T.

Since the creation of dental implants by Ingvar Branemark, in Sweden, the demand for treatment with osseointegrated implants has only grown, and it is not different in Brazil. Osseointegrated implants have constituted one of the greatest advances in dentistry in the last decades, offering the patient an esthetic and functional solution. The aim of this study was to evaluate the effect of smoking on the failure of dental implants and to propose measures to reduce the deleterious effects caused by tobacco use. The implant is a direct, structural and functional connection between vital bone and the surface of a titanium implant, covered by an artificial tooth. Several aspects may influence the success rates of implants, and the habit of smoking represents a greater risk failure of implant osseointegration but only if it considered together with other factors, such as poor bone quantity and quality, systemic diseases, anatomic limitations, initial stability degree, oral hygiene, occlusion, among others. The review of the literature shows that tobacco use has harmful effects on osseointegration and that even a temporary interruption of smoking before and after the process of osseointegration may lead to a more satisfactory prognosis. However, there is still need for more research to clarify what is the exact mechanism though which smoking interferes in this mechanism. Therefore, smoking alone cannot be considered responsible for failure in osseointegration in smokers, but is the dentist's responsibility to advise these patients that smoking can have a detrimental effect on this type of treatment.

## 047 Coronal flap sliding and Zucchelli and DeSanctis' papilla rotation with therapeutic purpose for coverage of multiple recessions

BARBOSA, K.T. (Keize Távora Barbosa kztavora@hotmail.com); BRAGA, A.C.O.P.; MARQUES, I.S.V.; OLIVEIRA, P.G.F.P.; VALLE, A.C.

Gingival recession is the apical migration of the marginal gingiva in relation to the cementoenamel junction. Several etiological factors are related, such as by brushing trauma, occlusal trauma, periodontal disease, tooth position in the arch, muscular attachment, orthodontic movement, restorative treatments, among others. These factors need to be removed before the surgical treatment to obtain the success. The root exposure brings as a consequence the appearance of dentin hypersensitivity, root caries, esthetic impairment and gingival disharmony. Due to the large current esthetic requirement, periodontics has developed several surgical techniques for handling the gingival tissue, which refer to the coverage of multiple recessions using the Zucchelli and DeSanctis' coronal slide technique, where oblique incisions are made beginning cementoenamel junction of the central tooth up to the end of the recession of the adjacent tissue. The outcome of this case was very favorable because of the correct indication of the proposed technique.

## 048 Chronic sinusitis and asymptomatic cervical mass: an odd association

TJIOE, K.C. (Kellen Cristine Tjioe kellen.tjioe@usp.br); ARAUJO, A.C.; SOARES, C.T.; RUBIRA-BULLEN, I.R.F.; CERVANTES, O.; DAMANTE, J.H.

A 57-year-old man was referred to our service complaining about painless neck lump with 6 months of evolution. He was smoker and used to consume alcohol occasionally. Physical examination revealed a firm and fixed cervical mass in the right side. Panoramic radiograph showed opacification of the right maxillary sinus – confirmed by the Water's projection - and an ill-defined sinus floor. There were dental implants in the region of upper right molars with severe periimplantitis. A curettage biopsy of sinus maxillary was performed through one implant socket and a fine-needle aspiration (FNA) biopsy was done in the cervical mass. The result of the sinus biopsy was actinomycosis without malignance. However, the nodular mass revealed to be metastatic squamous cell carcinoma in lymph node. Computed tomography (CT) and Positron Emission Tomography (PET) was prescribed to investigate the primary tumor that justify the lymph node metastasis. The primary tumor was not found. Surgical debridement of the sinus and neck dissection were performed. The histopathologic exams confirmed the first diagnostics. The tumor was a carcinoma of unknown primary site. Carcinoma of unknown primary site is defined as the histological diagnosis of metastasis without the detection of a primary tumor. Neck lymph nodes are the preferential sites. PET allows detection of primary tumor in about 25% of cases. In relation to the actinomycosis, correct diagnosis is difficult because the clinical and radiological findings closely resemble metastatic tumors and other infectious processes. The patient received radio/ chemotherapy and has been healthy for the past 15 months.

## 049 Is there still time for orthodontics in the best age?

CHIECO, K.H.F. (Khamila Follador Chieco khamilafc@hotmail.com); OLTRAMARI-NAVARRO, P.V.P.; NAVARRO, R.L.; CONTI, A.C.C. F.; ALMEIDA, M.R.; ALMEIDA, R.R.

Life expectance rate has increased in the past decades and a crescent number of elderly patients are searching for dental treatment aiming at esthetic and functional rehabilitation. Oral Gerontology has been developed with an emphasis in oral health care of the elderly population, specially aiming at prevention and therapies of pathologies or conditions of systemic and chronic, associated with physiological, physical and psychological deficiencies. Other specialties in Dentistry, such as Orthodontics, have become important allies in this new challenge of the dental profession. This study aimed at analyzing characteristics involved in orthodontic treatment when associated with gerontologic needs, considering relevant factors to an adequate application based on a case report. The treatment was planned integrating Periodontics, Orthodontics, Prosthodontics and Operative Dentistry practice, allowing the reestablishment of function and esthetics harmonically. Orthodontic treatment represents a feasible procedure in Gerontology, as long as applying light forces and respecting both the characteristics and limitations of these areas of treatment.

## 050 Reestablishment of the occlusal vertical dimension in the oral rehabilitation of partially dentate patients

SANTANA, L.S. (Laila Silva Santana lailasilvasantana@yahoo.com.br); REIS, M.V.P.; GOMES, J.B.; LIMA, J.H.F.; ORSI, I.A.

The success of oral rehabilitations is directly related to the diagnosis of alterations in the occlusal vertical dimension (OVD), as well as to the correct determination, registration and maintenance of intermaxillary relations. By a literature review, it was verified that the etiologic factors that might be responsible for OVD alterations include dental anomalies, abrasion, erosion, parafunctional habits, loss of posterior support, restorative materials of different compositions and wear resistance, and unsatisfactory dentures. It was concluded that once the etiological factor is precisely identified, efforts must be focused on controlling the determinant factors as well as on disclosing the need for reestablishing the patient's OVD. Whenever the reestablishment of the OVD is required, the clinician should maintain it during all the phases of the rehabilitating treatment and further transfer the correct occlusal vertical dimension to the definitive denture.

## 051 The use of CVD burs: an alternative for ultraconservative cavities

AZEVEDO, L.M. (Larissa Marinho Azevedo larissamarinhoodonto@yahoo.com.br); CASAS-APAYCO, L.C.; MODENA, K.C.S.; FRANCO, E.B.; ATTA, M.T.

Preservation of sound dental tissue is an important factor to perform minimal intervention cavities. Advances in technology of low and high-speed handpieces, ultrasonic dental scaler, burs design, materials and early detection of primary lesions provide the possibility of conservative cavity preparations. The development of diamond burs with chemical vapor deposition (CVD) coating used with a ultrasonic scaler provide an excellent cutting performance because they reach areas of the tooth that are inaccessible to high-speed handpieces. The use of this device allows the preparation of conservative and well finished cavity preparations. This case report shows benefits of the use of CVD diamond burs attached to ultrasonic dental scaler in the treatment of proximal caries. Sloping and different types of CVD diamond burs provide the access to proximal carious lesion with less pain and minimal noise, and no damages to the gingival tissues.

## 052 Esthetic recovery of endodontically treated tooth: bases and clinical steps

VASCONCELOS, L.R.S.M. (Larissa Rachel Silva Munhoz de Vasconcelos lari_mini@hotmail.com); CALABRIA, M.P.; WANG, L.; HANNAS, A.R.; PEREIRA, J.C.; MONDELLI, R.F.L.

Fracture of endodontically treated anterior teeth consists is a frequent situation, which denotes the importance of a correct understanding of techniques that can offer resistance of remainder dental structure and restoration. Endodontically treated teeth require special considerations to be restored since they commonly involve a great structural dental loss due to initial access for endodontic treatment, cavity preparation and pulp devitalization, which cause modifications on their physical-mechanical properties. A 25-year-old female patient looked for treatment at Bauru School of Dentistry, complaining of darkening of tooth #21. Clinical and radiographic exams were performed, revealing color alteration and a satisfactory endodontic treatment. Thus, a clinical treatment plan was proposed in order to reestablish esthetics and function of the compromised tooth. First, internal bleaching of this tooth was performed associated with an external bleaching involving from the range #15 to #25 and from #35 to #45 teeth in order to produce uniform color shade perception. Next, a glass-fiber post was fixed using a conventional glass-ionomer cement luting followed by the restoration with a resin composite system. Treatment steps showed that a correct planning combined with knowledge of the available techniques and material properties are essential to obtain excellence in esthetic and mainly in function responses to endodontically treated anterior teeth.

## 053 Integrated treatment planning to achieve esthetics within functional periodontal limits

SARAIVA FILHO, L. (Leonardo Saraiva Filho leonardosaraivafilho@hotmail.com); OLTRAMARI-NAVARRO, P.V.P.;NAVARRO, R.L.; CONTI, A.C.C.F.; ALMEIDA, M.R.; ALMEIDA, R.R.

Vertical orthodontic movements are an important alternative of orthodontic treatment and allow the manipulation of teeth and their surrounding tissues. The indications of this approach are correction of gingival margins during alignment and leveling, diminishing gingival discrepancies and “gummy smile” and recovery of periodontal defects. Rapid tooth extrusion is indicated when the choice is for prosthetic rehabilitation and there is subgingival fractures, caries, root perforations or external tooth resorption. Three to four millimeters of health dental structure above the bone crest are needed to obtain esthetic and periodontal integrity at the end of the restorative treatment. Aiming at reestablishing the biological distances, 2 mm should be preserved and the remainder will serve for prosthetic reconstruction of root remnant. If these distances are not respected, the coronal margins will be invading the periodontal physiological limits characterizing an aggression and, therefore, resulting in esthetic and functional damage to the patient. This work aimed at describing a case report of a the treatment of a maxillary right central incisor with crown fracture within 1 mm above the interdental bone crest. It was decided to perform orthodontic rapid extrusion to reestablish the biological distances and allow prosthetic preparations. Fixed orthodontic appliance was partially installed on the maxillary arch to allow a three-millimeter extrusion. In the end of extrusion movement, a crown lengthening periodontal surgery was performed followed by the endodontic treatment. A fixed prosthesis was installed to conclude the treatment. Integrated planning was determinant to achieve reestablishment of esthetics within functional periodontal limits.

## 054 Use of single unit all-ceramic prostheses in anterior teeth: case report

PEREIRA, L.V. (Leonardo Viana Pereira leovianapereira@hotmail.com); DEKON, S.F.C.; GABAN, G.; ZAVANELLI, A.C.; SILVA, C.R.

Due to the increasing esthetic demand, metal-free fixed partial denture systems have improved significantly in the last years, to the point to be possible the fabrication of these types of restorations even in bridge prostheses. Some of these systems use, among others, lithium dissilicate and aluminum as main compound in their composition, and the laboratorial processing can be based on injection, infiltration or use CAD-CAM systems. Due to the technique sensitivity of the several clinical and laboratorial steps involved, it is not possible to choose the most efficient, and each one should individually in order to obtain the maximum of their properties. The aim of this study was to present the case of a 42-year-old patient, F.A.S., treated at the clinic of Araçatuba School of Dentistry, with complaint of the esthetics of the maxillary anterior teeth and the mandibular left lateral incisor. After clinical and radiographic exams and analysis of studies models, the fabrication of complete crowns using the system All-Glass (EDG) was planned, with ceramic system All-Ceram (Degussa). The cementation was done with resin self-etch cement. It may be concluded that the system used in this study has favorable esthetics when properly processed.

## 055 Effect of commercial desensitizing dentifrices on the prevention of enamel erosion associated with abrasion

MONTAGNOLLI, L.G. (Lia Guimarães Montagnolli liamontagnolli@usp.br); BARBOSA, C.S.; KATO, M.T.; BUZALAF, M.A.R.

The aim of this study was to evaluate, *in vitro*, the effect of dentifrices that are claimed to have a desensitizing action on the prevention of enamel erosion associated with brushing abrasion. Ninety bovine enamel specimens (4X4X3 mm) were randomly allocated to 5 groups (n=18/group), according to the treatments: Sensodyne Pro-Esmalte® (SPro, 1,425 ppm NaF, 5% KNO3), Sensodyne original® (SOri, no fluoride, 10% SrCl2), Colgate Sensitive® (CSen, 1,450 ppm MFP, 5% K citrate), Crest® (Cr, positive control, 1,100 ppm NaF) and water (Wt, negative control). The samples were subjected to 30 cycles, alternating re- and demineralization (5 days). Demineralization was performed with Coca-Cola® (1min, 30mL/block) and remineralization with artificial saliva (59 min, 30 mL/block), at 37°C under agitation. Every 2 de-remineralization cycles, abrasion was conducted after demineralization, using an automatic toothbrush (10 s/1 mL of slurry of the dentifrices, 3g dentifrice/10mL water) or water (negative control. The wear was analyzed by profilometry. Data were analyzed by ANOVA and Tukey's test (p<0.05). The mean wear (±se, μm) was significantly lesser for CSena (0.55±0.03), SOrib (0.71±0.02) and Crab (0.63±0.13) when compared to SProc (2.38±0.14) or Wtd (3.15±0.17). These results suggest that the presence in dentifrices of fluoride or desensitizing substances alone or in combination can reduce the wear of enamel subjected to erosion associated with brushing abrasion, but this is not valid for all formulations.

## 056 All-ceramic system failures: case report

SANTOS, L.A. (Livia Aguiar Santos livia-aguiarsantos@hotmail.com); MARTINS, L.M.; OLIVEIRA, P.C.G.; LORENZONI, F.C.; BONFANTE, G.; LOPES, L.D.S.

The continuous search for esthetic and biocompatible materials, capable of withstanding masticatory efforts led to the development of sufficiently resistant ceramics to be used as frameworks on fixed prosthodontics (lithium dsilicate, alumina and zirconia). Thus, all-ceramic prosthesis became alternative materials to traditional metal-ceramic prostheses. However, even with improvements related to fracture resistance, all-ceramic crowns still present higher failure numbers compared to metal-ceramic prostheses. While metal-ceramic systems present biological problems (carious lesions and periodontal disease) as the main cause of failures, all-ceramic systems present mechanical problems (fracture of coverage or framework ceramics). The pattern of failure of all-ceramic systems depends on the ceramic material, its composition, shape and size of crystalline phase granules. Therefore, in terms of mechanical reliability, rates of metal-ceramic system survival are still the goal to be achieved by all-ceramic crowns. In the metal-ceramic systems, there is no difference between regions; on the other hand, the clinical reliability of all-ceramic systems on the anterior region is higher than on the posterior region. The aim of this study was to describe and discuss main failure modes present in the various ceramic systems by means of a case report.

## 057 Effect of TiF_4_ and AmF treatment combined with CO_2_ laser-irradiation on enamel and dentin erosion

COMAR, L.P. (Lívia Picchi Comar liviacomar@usp.br); WIEGAND, A.; MAGALHÃES, A.C.; NAVARRO, R.S.; RIOS, D.; BUZALAF, M.A.R.

This *in vitro* study aimed to analyze the influence of CO_2_ laser irradiation on the efficacy of fluoride solutions (TiF_4_ and AmF) to prevent enamel and dentin erosion. Bovine enamel and dentin samples (n=10 each/group) were pretreated with CO2 laser irradiation (group I); TiF_4_ (1% F, group II); CO2 laser irradiation prior to (group III) or through (group IV) TiF_4_ application; AmF (1% F, group V) or CO_2_ laser irradiation prior (group VI) or through (group VII) AmF application. Controls remained untreated. The samples were then subjected to erosive demineralization (Sprite Zero, 4 x 90s/day) and remineralisation (artificial saliva, between the erosive cycles) cycling for 5 days. Enamel and dentin loss was measured profilometrically after pretreatment, 1st day (4 cycles) and 5th day (20 cycles) of pH cycling. The data were analyzed statistically by ANOVA and Scheffe's post-hoc tests (p<0.05). SEM analysis was performed in pretreated but not cycled samples (n=2/group). After 5th day, enamel loss was significantly decreased in group V and IV, while dentin loss was significantly reduced in group V only. All other groups were not significantly different from the controls. Lased surfaces (group I) appeared unchanged in the SEM images. However, microscopic images of enamel, but not of dentin, showed that the formation of fluoride precipitates was affected by CO_2_ laser irradiation. Thus, AmF decreased enamel and dentin erosion but its efficacy was not improved by CO_2_ laser irradiation. TiF_4_ showed only a limited capacity to prevent erosion, but CO_2_ laser irradiation enhanced its ability to reduce enamel erosion to a significant level.

## 058 Labial frenectomy prior to orthodontic treatment: case report

FINOTI, L.S. (Livia Sertori Finoti lifinoti@msn.com); CHIARELLI, F.M.; BORGES FILHO, F.F.; ANOVAZZI, G.

An upper labial frenum (ULF) inserted in the palatal papilla will prevent natural diastema closure between maxillary central incisors after permanent lateral incisors and canines eruption. Pathological ULF, also known as persistent tectolabial frenum, is present in few adult patients, their usual complaint is about unsatisfactory esthetics because of the median diastema that may persist even after orthodontic closure when this frenum is not removed. Frenectomy is the complete excision of the frenum including its attachment to the underlying bone. One technique to remove it is the conventional approach using periodontal incisions with scalpel. The aim of this case report is to demonstrate with details a frenectomy of a pathological ULF where a different approach was done to reduce bleeding and surgery time. The patient presented a pathological ULF and a 7 mm diastema between upper central incisors, and was willing to receive orthodontic treatment in order to close this space. Surgical removal was then indicated prior to median diastema closure. After the healing period the ULF was normal and present in a more apical position, there was an increase in attached gingival height and vestibular depth.

## 059 External bleaching and direct composite resin veneer in a stained maxillary central incisor: esthetic procedures in vital teeth

SOUZA, L.V. (Lorraine Vilela de Souza lorraine_vs@hotmail.com); RAPOSO, L.H.A.; SOARES, P.V.; SOARES, C.J.

With the advent and improvement of the adhesive techniques in the contemporary dentistry, there has been a continuous search for esthetics. Even more, patients unsatisfied with their teeth for several reasons look for conservative and affordable treatment solutions. In some cases, simple procedures can be performed to help those patients, recovering their satisfaction and esthetics. A 35-year-old female patient attending the Integrated Dental Clinic program complained of her right maxillary central incisor color and her smile. Clinical and radiographic evaluation revealed tooth vitality and no previous endodontic therapy. None specific congenital tooth staining was noted. The treatment of choice was established as a single tooth external bleaching with a 35% hydrogen peroxide in-office bleaching system. After the bleaching procedure, tooth shade appeared adequate for the dentist and the patient. However, in a second visit, 15 days after bleaching, the patient was still unsatisfied with her teeth and another treatment modality was chosen. At this time, a direct composite resin veneer was suggested. All steps for a direct veneer correct preparation with knife edge design in a vital tooth were carried out as means to produce sufficient restorative material for the esthetic restoration. After conditioning of the dental structures with 37% phosphoric acid, a single-step adhesive system was applied in two layers and photo-activated for 20 seconds. Nanofilled composite resins from different shades were inserted incrementally and photoactivated for 20 seconds to built the restoration. Subsequently, finishing of the restoration was performed with extra-fine grit diamond burs and abrasive silicone polishing tips, and an occlusal evaluation was performed. External bleaching and the direct composite resin veneering are conservative procedures that can be used routinely in the dental clinical practice, reestablishing esthetics in an inexpensive and easy way.

## 060 Biocompatibility evaluation of Epiphany/Resilon root-canal filling system in subcutaneous tissues of rats

ROSELINO, L.M.R. (Lourenço de Moraes Rego Roselino lourencoroselino@yahoo.com.br); GARCIA, L.F.R.; MARQUES, A.A.F.; PIRES-DE-SOUZA, F.P.C.; CONSANI, S.

Several studies have been conducted to assess the biocompatibility of root canal sealers, which is essential for ensuring their good performance and successful endodontic treatment. The purpose of this study was to evaluate the biocompatibility of the new root canal filling system Epiphany/Resilon in the subcutaneous tissues of rats. Fifteen rats weighing 250 g on average were separated into three groups (n=5), according to period of sacrifice (7, 21, 42 days). As part of the surgical procedure, four incisions were made on the dorsal region of each rat, where four dentin tubes filled with the tested materials were implanted in the subcutaneous tissue, as follows: GI - Epiphany/Resilon without primer; GII - Epiphany/Resilon associated with self-etch primer; GIII - Endofill/gutta-percha points (positive control) and GIV - empty tube (negative control). After 7, 21 and 42 days, animals were killed, obtaining 5 samples per group, at each analyzed time. From the morphologic and morphometric analyses, using a score system (0 - 3) (100x), results showed that Epiphany/Resilon system (GI and GII) induced a mild (1) inflammatory reaction after 42 days. However, in GI, in which the primer was not applied, extensive necrosis and a severe (3) to moderate (2) inflammatory reaction were observed, between 7 and 21 days. When compared to the control groups, it was observed that these groups presented tissue reaction ranging from mild (1 - 7 and 21 days) to absent (0 - 42 days). In conclusion, Epiphany/Resilon root-canal filling system presented satisfactory tissue reaction, being biocompatible when tested in subcutaneous tissues of rats.

## 061 Inferior alveolar nerve transposition for setting of osseointegrated implants

HOMSE, L.C. (Lucas Correa Homse lucas_homse@yahoo.com.br); CAVALIERE-PEREIRA, L.; PASTORI, C.M.

In the rehabilitation of the edentulous posterior mandible with the technique of osseointegrated implants some items that should be evaluated such as the quantity and bone quality and anatomical location of the remaining mandibular canal. These are extremely important for the stabililization and subsequent success of the implant. When teeth are lost, the aveolar bone loses its function and is physiologically atrophied. This is important, particularly in the posterior region of the mandible by the presence of a noble structure, the vasculo-plexus inferior alveolar nerve. The anatomical location of this plexus in atrophic mandibles sometimes makes impossible the rehabilitation with implants. There are some techniques for reconstruction of the posterior mandible to these situations, such as bone grafts for gain in height, set of short implants and inferior alveolar nerve transposition. This is to change the way of the inferior alveolar nerve for installation of dental implants. There are two different surgical techniques: one in which the nerve is transposed without including the foramen mentoniano and another in which the foramen is included. The choice for the technique is done by the extension of the area to be rehabilitated. Some accidents and complications are inherent to the surgical procedure, and the most common is transient paresthesia. The objective of this work is to present a clinical case of surgical rehabilitation of posterior mandibular osseointegrated implants associated with inferior alveolar nerve transposition as well to discuss the indications and the contraindications, advantages and disadvantages of this technique.

## 062 Dose–response of estrogen in bones of ovariectomized rats

LAHR, L.P. (Lucas Perez Lahr lucas_lahr18@hotmail.com); DUARTE, N.T.; PRATES, L.F.; NAKAMUNE, A.C.M.S.; SALZEDAS, L.M.P.; DORNELLES, R.C.M.

Estrogen plays an important role in both reproductive and nonreproductive tissues. It influences pubertal growth, regulates bone maturation, and maintains bone mass in women and in men after puberty. In menopausal women, it is associated with various diseases, including bone resorption. Currently, medicine has attempted to make the hormone replacement without causing further damage to the body. The goal of the present study was to systematically define the dose–response effect of estrogen on the bones of ovariectomized rats. Wistar rats (6 m) were selected after analysis of estrous cycle, and ovariectomized (OVX). After 15 days, the animals received pellets containing corn oil or estrogen with 100 or 200 ìg of 17â–estrogen, during 60 days. After this period and under anesthesia (xylazine-4mg/kg; ketamine-40mg/Kg), blood was collected from the jugular vein and the animals were sacrificed by anesthetic overdose for removal of the femurs. The blood was centrifuged and plasma stored in a freezer for subsequent biochemical determination of calcium and phosphorus. The femurs were tested on the three-point bending, axial compression was applied to the condyle and bone densities were analyzed using Digora digital system. The results showed no statistically significant difference in the analysis of the middle third of the three experimental groups. However, the optical density was lower in the condyle of the femur in the group of animals that received 200 ìg of estrogen for 60 days. The plasma concentration of phosphorus in the group of OVX animals treated with 100 ìg/estrogen was significantly lower and calcium was similar between groups. These results suggest there are important differences between the axial and appendicular skeleton in terms of the response to different doses of estrogen.

## 063 Orthopedic treatment with IHG headgear appliance followed by orthodontic correction – a case report

PIERI, L.V. (Lucelma Vilela Pieri lucelmapieri@usp.br); HENRIQUES, J.F.C.; FREITAS, M.R.; JANSON, G.; PINZAN, A.; PUPULIM, D.C.

The Interlandi Head Gear appliance (IHG) with occipital anchorage or mean traction is indicated in Class II Division 1 treatment with maxillary and/or dentoalveolar protrusion, maxilla with normal transverse dimensions and mandible well positioned. The IHG headgear influences significantly bone and soft tissue profile providing a harmonious profile, mainly in growing patients with vertical pattern or increased LAFH. The upper right and left first molar tubes are placed on a cervical position. The external and internal arches are parallels to the occlusal plane with the external arch 2 cm longer than internal one. Variations in the angulations of elastic ½ inch over the occlusal plane with individual ideal forces from 350 to 600 g bring about effects and action mode according to the facial types and desired effects when inserted in the extremities of external arms and attached to occipital traction of Interlandi type. The upper molar crowns can move more than their roots with angulation below 15° with extrusion in the horizontal and normal pattern. From 15° to 20° of angulation, the force pass through the molar's center of resistance (trifurcation area) causing a bodily movement of the upper molars to distal with extrusion restriction in the normal to vertical pattern. The roots and crowns move to distal practically in the same proportion. An angulation more than 20°, the roots start to move distally more than crowns with slight “intrusion” in severe vertical pattern and/or increased LAFH. More this angulation is increased more the roots move distally and the upper molar extrusion is restricted. A 9-year old girl with convex profile and severe horizontal trespass, without any space for the eruption of the permanent upper lateral incisors treated with an IHG appliance (20° angulation and 350g each side) worn 24 hours/day for seven months will be presented. The upper and lower fixed appliances were placed to align and level the teeth. The IHG was efficient in moving upper molars distally with a body movement once the forces passed through the center of resistance bringing about sufficient space for eruption of the permanent teeth and a harmonic facial profile.

## 064 Balance between proliferation and apoptosis of parenchymal cell during atrophy of submandibular glands of rats with Alloxan-induced diabetes

KUROKAWA, L.A. (Luciana Ayumi Kurokawa luciana_ayumi@hotmail.com); ASSIS, G.F.; CESTARI, T.M.; CEOLIN, D.S.; TAGA, R.

The non-compensated diabetes leads to several progressive changes in the body that in many cases become irreversible. In the mouth, these changes occur mainly by the decrease in salivary flow and damage the quality of saliva. The major salivary glands are the parotid and submandibular because they have more influence by producing most of the saliva. The secretory portion of the submandibular gland suffers greater influence in conditions of diabetes. Therefore, histological sections were obtained and stained with hematoxylin and eosin to obtain the total number of acinar cells and granular ducts and also subjected to immunohistochemical procedures for cell proliferation (Method of PCNA) and apoptosis (TUNEL method) in periods of 1, 3 and 6 months of induction of diabetes in rats. The figures were compared among groups by analysis of variance (ANOVA) at the level of 5%. Over the 6 months of experimental diabetes: a) the mass of the submandibular gland decreased according to the reduction of acini and granular ducts b) the absolute volume of acini decreased mainly because the volume of acinar cells and granular ducts on the basis of cell volume and number of cells c) the number of acinar cells and granular ducts that are produced and die has a low rate close to the physiological condition, the apoptosis being slightly larger than the proliferation, thus leading to reduction of the mass of submandibular gland. It may be concluded that diabetes induced by Alloxan over 6 months in rats caused a gradual decrease in submandibular gland as a function of the cell volume and the greater occurrence of death of cells of the secretory system, particularly the granular ducts.

## 065 i-CAT: advantages and applications of cone beam computed tomography

FERNANDES, L.M.P.S.R. (Luciana Maria Paes da Silva Ramos Fernandes lucianamfernandes@usp.br); RUBIRA-BULLEN, I.R.F.; CAPELOZZA, A.L.A.

Cone beam computed tomography is a diagnosis resource frequently used in Odontology. It is a digital exam, which allows accurate and three dimensional (axial, sagittal and coronal) evaluation of the region to be studied. The absence of superimposition of the structures in tomographic images and the obtaining of illustrative 3-D reconstructions are advantages of this new technology. The exam is easy and quick to perform and offers convenience to the patient. The purpose of this presentation is to introduce the main features, advantages and applications of i-CAT (Imaging Sciences, USA), illustrated with cases in which this technology has been applied.

## 066 Orthodontic treatment in adults associated with implant rehabilitation – a case report

BERTATO, L.P.1 (Ludmila da Paz Bertato ludmilabertato@yahoo.com.br); PIERI, L.V.; HENRIQUES, J.F.C.; JANSON, G.; FREITAS, M.R.; PINZAN, A.

Orthodontic treatment in adults is complex once we cannot count on growth anymore to correct malocclusion. In addition, dental losses can be involved as well as periodontal diseases. A case report of a dolichofacial 28-year-old woman with Class II malocclusion, maxillary protrusion and mandibular retrusion, severe overjet, deep bite, increased LAFH, severe convex profile, absence of seal lip and dental losses (46 and 47) will be presented. This patient was treated with dental compensations, extraction of teeth #14 and #24, mesialization of tooth #48 and implantation of tooth #46. The orthodontic treatment in adults should have a multidisciplinary approach for success to be reached and maintained after the retention orthodontic phase.

## 067 Esthetic and functional correction of upper central incisor with porcelain veneer

OLIVEIRA-NETO, L.A. (Luiz Alves de Oliveira Neto luizneto@usp.br); LORENZONI, F.C.; COSTA, M.D.; MARTINS, L.M.; BONFANTE, G.

Current dentistry recommends that restorations must be esthetic, functional and with minimal biologic damage to dental tissues. Porcelain veneers restore esthetics, function and presents minimal biological damage compared to tooth preparations to full crowns, which involve larger reduction of enamel and dentin, procedure that can irreversibly affect the pulp. Porcelain veneers are the restorative choice for esthetics in various clinical situations, such as diastema restorations, misalignment correction, and discolored or teeth with shape defects. Ceramic is the material used in veneers due to their superior characteristics such as esthetics and biocompatibility, even when it is possible to do composite restorations. Chemical stability, lower cytotoxicity, reduced risk of tissue irritation and reduced accumulation of plaque are some advantages related to ceramics. They have been used as a choice material in reconstructive dentistry by imitating the characteristics of natural teeth. The development of ceramic systems allowed many restorative possibilities. However, in order to mimic the intrinsic and extrinsic characteristics of color, shape and surface texture is still a challenge. Most laminates can be done with feldspathic porcelain of low melting or pressable ceramics. The aim of this study is to present and discuss the challenges of planning and constructing a single-unit porcelain veneer in a esthetically compromised central incisor without possibilities of alignment with orthodontics due to root resorption.

## 068 Morfo-functional comparison between dental and periodontal tissues of deciduous and permanent teeth

GRACINDO, L.F. (Luiz Fernando Gracindo fernandogracindo@hotmail.com); AYUB, B.; NASCIMENTO, B.M.; BELEZE, P.; ALCALDE, M.P.; SILVEIRA, E.M.V.

This study is a review of the difference between the human dentitions regarding the shape, size and histological structures with the aim of assisting in prevention, diagnosis and treatment of these structures. Books and papers of authors with worldwide reputation in the subject were used, serving as base to support the study. The target of the research was the critical analysis of all dental structures as well as the supporting tissues aiming to demonstrate the main discrepancies between these organs in the different phases of their development. The main approach was given to the following tissues: enamel, dentin, pulp and periodontium and, the physical and anatomical characteristics and the mineralization degree of the tissues were analyzed in a general context. It is expected that this information could help in the daily clinical routine of dental professionals, as well as in the research activities for development of new technologies that can improve the quality of life of the population.

## 069 Influence of different tightening forces before laser welding to the implant/framework fit

CAVALCANTE, L.A.L. (Luisa de Andrade Lima Cavalcante luisa. cavalcante@gmail.com); SILVEIRA-JÚNIOR, C.D.; NEVES, F.D.; FERREIRA, F.M.; SIMAMOTO-JÚNIOR, P.C.

The aim of the present study was to evaluate the influence of abutment screw tightening force before laser welding procedures on the vertical fit of metal frameworks over four implants. To construct the frameworks, prefabricated titanium abutments and cylindrical titanium bars were joined by laser welding to compose three groups: group of manual torque (GMT), GT10 and GT20. Before welding, manual torque simulating routine laboratory procedure was applied to GTM. In GT10 and GT20, the abutment screws received 10 and 20 Ncm torque, respectively. After welding, the implant/framework interfaces were assessed by optical comparator microscope using two methods. First, the single screw test (SST) was used, in which the interfaces of the screwed and non-screwed abutments were assessed, considering only the abutments at the framework extremities. Second, the interfaces of all the abutments were evaluated when they were screwed. In the SST, intergroup analysis (Kruskal Wallis) showed no significant difference among the three conditions of tightening force; that is, the different tightening force before welding did not guarantee smaller distortions. Intragroup analysis (Wilcoxon) showed that for all groups, the interfaces of the non-screwed abutments were statistically greater than the interfaces of the screwed abutments, evidencing distortions in all the frameworks. ANOVA was applied for the comparison of interfaces when all the abutments were screwed and showed no significant difference among the groups. Under the conditions of this study, pre-welding tightness on abutment screws did not influence the vertical fit of implant-supported metal frameworks.

## 070 Esthetic and functional correction of upper central incisor with porcelain veneer

OLIVEIRA-NETO, L.A. (Luis Alves de Oliveira Neto luizneto@usp.br); LORENZONI, F.C.; COSTA, M.D.; MARTINS, L.M.; BONFANTE, G.

Current dentistry recommends that restorations must be esthetic, functional and with minimal biologic damage to dental tissues. Porcelain veneers restore esthetics, function and presents minimal biological damage compared to tooth preparations to full crowns, which involve larger reduction of enamel and dentin, procedure that can irreversibly affect the pulp. Porcelain veneers are the restorative choice for esthetics in various clinical situations, such as diastema restorations, misalignment correction, and discolored or teeth with shape defects. Ceramic is the material used in veneers due to their superior characteristics such as esthetics and biocompatibility, even when it is possible to do composite restorations. Chemical stability, lower cytotoxicity, reduced risk of tissue irritation and reduced accumulation of plaque are some advantages related to ceramics. They have been used as a choice material in reconstructive dentistry by imitating the characteristics of natural teeth. The development of ceramic systems allowed many restorative possibilities. However, in order to mimic the intrinsic and extrinsic characteristics of color, shape and surface texture is still a challenge. Most laminates can be done with feldspathic porcelain of low melting or pressable ceramics. The aim of this study is to present and discuss the challenges of planning and constructing a single-unit porcelain veneer in a esthetically compromised central incisor without possibilities of alignment with orthodontics due to root resorption.

## 071 Evaluation of the erosive potential of regular and light acidic drinks on dentin

CASSIANO, L.P.S. (Luiza de Paula Silva Cassiano luiza.cassiano@usp.br); RIOS, D.; MOINO, A.L.; MAGALHÃES, A.C.; HONÓRIO, H.M.; BUZALAF, M.A.R.

This *in vitro* study aimed to compare the erosive potential of different regular and light beverages on dentin. Bovine root dentin samples were divided into 9 groups (each n=12) and immersed in the regular or light/sugar zero version of the following drinks: Coca-cola, Guaraná, Sprite and passion fruit juice (Del Valle). Half of the surface of the specimens was coated with nail varnish for reference. The samples were subjected 3 times daily to erosive challenges (30 mL beverage/sample) for 5 minutes at room temperature, under agitation. Between the erosive challenges, the samples were immersed in artificial saliva (30 mL/sample) for 5 h, at room temperature. In each day, after the pH-cycles the samples were stored in artificial saliva. At the 5th day, the dentin surface alterations were measured by profilometry (μm). The data were analyzed by ANOVA followed by Tukey's test (for cola drinks) and unpaired t test (for other beverages) (p<0.05). There were significant differences between the regular and light beverages, except for the regular and light passion fruit juices (regular: 2.43±0.66 and light: 2.36±0.61). Regular coke (2.53±0.39) provoked a similar dentin wear compared to zero coke (2.46±0.49), but both caused higher dentin loss compared to light version (1.88±0.35). On the other hand regular Guaraná (2.35±0.51) provoked lower dentin loss compared to light one (2.88±0.69). Regular sprite (2.49±0.59) also provoked lower dentin erosion compared to light version (3.09±0.72). Thus, this *in vitro* study showed that regular and light drinks might have different performances with regard to dentin erosion, with exception of the fruit juice.

## 072 Enamel microhardness change after erosive challenge with regular and light drinks

JORDAO, M.A. (Maisa Camillo Jordão maisacjordao_usp@hotmail.com); REBELATO, R.; MAGALHÃES, A.C.; HONÓRIO, H.M.; BUZALAF, M.A.R.; RIOS, D.

A previous *in situ* study showed differences on the erosive potential of a regular and a light cola drink on enamel, after 14 days of erosive challenge. However, no information is available about the erosive potential of regular and light drinks in short challenges, which promotes initial erosive lesions with demineralization and no wear. Thus, this *in vitro* study aimed to evaluate the enamel demineralization after short erosive challenge using different regular and light beverages. Bovine enamel samples were divided into 7 groups (each n=6) and immersed in the regular or light/sugar zero versions of the following drinks: Coca-cola, Guaraná and Sprite. The samples were subjected 4 times per one day to erosive challenges (30 mL beverage/sample) for 2 minutes at room temperature. Between the erosive challenges, the samples were immersed in artificial saliva (30 mL/sample) for 2 hours. After each immersion (total of 4), the enamel surface alterations were measured by superficial microhardness test (KHN). The data were analyzed by ANOVA followed by Tukey's test (for cola drink), unpaired t test (for guaraná) and Mann Whitney test (for sprite) (p<0.05). On all measurements, light cola provoked a similar enamel demineralization compared to zero cola, and both caused less demineralization compared to the regular version. The enamel microhardness change of regular and zero Guaraná did not differ from each other, on all of the 4th immersions. However, zero sprite resulted in higher demineralization when compared to regular sprite, only in the 3rd and 4th erosive challenge. This *in vitro* study showed that the light and zero cola drinks promoted less enamel demineralization, but this was not observed for the zero versions of guaraná and sprite. Financial support: FAPESP (Proc. 2006/07260-4).

## 073 Adhesive cementation: materials and technique – a literature review

REIS, M.V.P. (Manuella Verdinelli de Paula Reis manu_verdinelli@hotmail.com); SANTANA, L.S.; GOMES, J.B.; ORSI, I.A.; ONO, R.; GERVÁSIO, A.M.

Since the cementation is the final phase of the indirect restorative treatment, it is necessary that the operator understand its importance, the specific technique and the appropriate luting materials, because these factors will determine a satisfactory completion of the indirect restoration. A review of the literature addressed the factors related to the cementation of the esthetic restorations such as the treatment of the inner surface of the prosthetic piece and the treatment of the tooth, in addition to the correct selection of the resin luting agent for each indirect restoration involved. It is concluded that in all the adhesive procedures, the success of the cementation of the indirect pieces depends on the quality of the piece/tooth union provided by the intermediary adhesive agents. Considering that indirect restorations are exposed to an extremely adverse environment and that they can fail due to the action of chemical, physical and biological factors, or by the combination of them, the knowledge of main features of the luting agents and their mechanisms of action is essential to reduce the risk of failure of restorative procedures and obtain a successful rehabilitating outcome.

## 074 Analysis of the tensions in implant-supported frameworks with alteration of the number of regular implants in the Brånemark protocol

PRUDENTE, M.S. (Marcel Santana Prudente marcel_prudente@hotmail.com); SILVA-NETO, J.P.; SIMAMOTO-JÚNIOR, P.C.; ARAÚJO, C.A.; NEVES, F.D.; NÓBILO, M.A.A.

This goal of this study was to evaluate the mechanical behavior of Brånemark protocols with alteration of the number and distribution of the implants through the plane photoelasticity technique, distributed in two groups, G5 with five implants (n=3) and G3 with three implants (n=3). The photoelastic models were built starting from six metal frameworks. Force of 1.33 kgf was applied in one distal extension of the prosthesis, evaluating the stress gradient in 16 points distributed along the implants. The shear bond stress was determined using the equation of the optical law of the tensions. From the analysis of the obtained values, normalized by G5, it was observed that G3 presented values of tension 12% greater than G5 in the cantilever area, while the other implants also presented higher values, but the difference was very small. Based on the obtained results it was observed greater values of shear bond stress in all implants from G3, when compared to their counterparts in G5, with greater concentration in those adjacent to the cantilever area. In spite of this, given to the practical and economical advantages of the protocol with three regular implants, further studies are needed before it can be established as a routine clinical protocol, benefitting a larger number of individuals. It is suggested, from the exposed, a similar work increasing the diameter of the implants.

## 075 Patients with chronic gingivitis as periodontitis-genetic resistant subjects: an alternative approach to unravel the genetic basis of periodontitis susceptibility/resistance based on classic functional SNPs data

CLAUDINO, M. (Marcela Claudino da Silva garletgp@usp.br); CARDOSO, C.R.; ASSIS, G.F.; MARTINS JR, W.; TROMBONE, A.P.F.; GARLET, G.P.

Many studies have investigated the genetic basis of periodontal disease (PD), but usually inconclusive or negative results have been found, possibly due an inappropriate case-control experimental design. Indeed, the inclusion of periodontally healthy subjects (with a proper microbial control) as a control population does not assure that this individuals are genetic susceptible to PD. The objective of study was to evaluate the potential of chronic gingivitis subjects, exposed to a chronic bacterial and inflammatory challenge but without evidences of bone loss, to be included as a genetically-resistant population in PD genetic association studies. Therefore, we investigated the frequency of different functional SNPs (single nucleotide polymorphisms) IL10-592, TNFA-308 and IL1B-3954 in healthy (H/n=190), chronic periodontitis (CP/n=178) and chronic gingivitis (CG/n=153) subjects. Using the traditional H vs CP approach, our results demonstrated a lack of association regarding TNFA-308 ((p=0.1882/OR=0.7265)), and weak to moderate association to IL10-592 (p=0.0001, OR=2.309) and IL1B-3954 (p=0,0169/OR=0.5886). However, when the alternative CG vs CP evaluation is analyzed, stronger statistical values were found to TNFA-308 (p=0.0051/OR=2,208), IL10-592 (p<0.0001, OR=0.2811) and IL1B-3954 (p=0.0075/OR=0,5348), suggesting that a comparison between susceptible (CP) and resistant (CG) subjects is a more suitable approach to unravel the association of SNP with PD outcome. Indeed, based on PD natural history literature data, H group virtually include (approximately 70%) susceptible and (30%) resistant subjects, but the proper microbial control does not allow the distinction between these opposing phenotypes within H groups. Surprisingly, when H and CG groups were compared, no significant variations in the frequency of the SNPs investigated were found. Therefore, our results demonstrated that CG/CP analysis provide a more appropriate and powerful tool to investigate the genetic basis of PD susceptibility/resistance than the traditional H/CP approach; and also reinforce the hypothesis that CG patients may present a potentially genetically-resistant background regarding PD development.

## 076 Ameloblastic carcinoma: clinical case report

MIYASAKI, M.L. (Marcela Lumi Miyasaki marcela_mlm@hotmail.com); FRANÇA, D.C.C.; AGUIAR, S.M.H.C.A.

The ameloblastic carcinoma is a rare lesion that can occur in patients of both sexes and in many stages of life. This lesion, although malignant, may have a slow development. It is reported the case of a 59-year-old man that sought a dentist in the city of Tangará da Serra, MT, complaining of “pain in the bone.” The dentist identified the lesion area and made a curettage. The patient felt pain relief. However, two years later, he returned feeling the same symptoms and the lesion grew rapidly. It was decided to refer the patient to a specialized center. In the clinical examination, he reported pain and presented facial asymmetry, volume increase of the right side, alar nose elevation and disappearance of the nasolabial sulcus. In the intraoral examination, there was volume increase of the hard palate, with appearance of the lesion without ulceration. The upper left central incisor presented mobility. A computed tomography scan revealed expansion and destruction of the alveolar cortical plate, involving the nasal cavity and the eyeball. Incisional biopsy was done. The material was sent to the oral pathology laboratory. Histologically there was observed ectodermal cancer consisted of cells interpreted as ameloblasts with intense pleomorphism, hypercromatic and atypical mitoses cellular. Along the clinical data, image reviews and histopathological aspects it was concluded the diagnosis of ameloblastic carcinoma, whose treatment is very aggressive. However, when detected and treated early it get better prognosis of the case.

## 077 *In situ* evaluation of a dentifrice with low fluoride content and supplemented with phosphate in caries progress

DANELON, M. (Marcelle Danelon marcelledanelon@hotmail.com); TAKESHITA, E.M.; CASTRO, L.P.; SASSAKI, K.T.; DELBEM, A.C.B. FAPESP (proc #07/03254-2, 07/03660-0).

The aim of this *in situ* study was to evaluate whether the supplementation with sodium trimetaphosphate (TMP) of a dentifrice with low fluoride (F) content (500 μg/g) would provide a similar effect to that of a standard dentifrice (1100 μg F/g). In this crossover double blind study, 9 volunteers, wearing acrylic palatal appliance containing 4 enamel bovine teeth, were subjected to 4 treatment groups: placebo (negative control), dentifrice with low-F, dentifrice with low F and 1% TMP and a dentifrice with 1100 ìg F/g (positive control). During the experimental period (14 days each), test dentifrices were applied 2x/day, and a 20% sucrose solution was applied 6x/day by being dripped on the blocks. After each phase, surface microhardness was assessed to calculate the lesion progress (△KHN), and F, calcium (Ca) and phosphorus (P) present in enamel were also measured. The results showed that the dentifrice supplemented with F and 1% TMP showed the lowest △KHN (p<0.05). The order of effect in reducing △KHN was dentifrice with low F and 1% TMP > positive control > dentifrice with low-F > placebo. Regarding F and Ca in enamel there were no differences between dentifrice with low-F and TMP and positive control (p>0.05), but they were different when compared to dentifrice with low F (p<0.05). It is concluded that the addition of 1% of TMP to the 500 μg F/g dentifrice allowed a similar effect as compared with a standard dentifrice in this *in situ* model.

## 078 Membranes and bone graft material: study in dogs

MORETTO, M.J. (Marcelo Juliano Moretto mjmoretto@terra.com.br); BERNABÉ, P.F.E.; GOMES FILHO, J.E.; DEZAN JR, E.; NERY, M.J.; OTOBONI FILHO, J.A.

The literature is scarce regarding the use of membranes and bone graft materials isolated or associated, justifying to the need of experiments in biological models to determine the actual indication of these materials in surgical procedures. Therefore, 48 roots with associated periapical lesion of 6 dogs underwent apical surgical interventions. Trephine drills (5 mm diameter) were used for standardization of the surgical site, after root-end resection, lesion curettage, preparation of the root-end cavities with ultrasound and retrofilling with mineral trioxide aggregate. The 48 sites were divided as follows: Group 1 - filled with blood clot; Group 2 - filled with blood clot and covered with membrane; Group 3 - filled with bovine bone; Group 4 - filled with bone bovine and covered with membrane. The results showed that the inflammatory infiltrate and the periapical healing process were similar in all groups. It was concluded that the use of membranes and bone graft materials isolated or associated in apical surgery did not alter the periapical healing process.

## 079 Odontogenic keratocyst tumor in mandibular anterior area: a case report

STEFANELI, M.A.B. (Márcio de Ávila Baena Stefaneli marciobaena_5@hotmail.com); RODRIGUES, K.; BAGATELI, J.C.E.; KAMEI, N.C.

The odontogenic keratocyst tumor, named by Reichart in 2002, was first described by Phillpsen in 1956, being initially termed as odontogenic keratocyst. Due to its destructive potential and high recurrence rate, this lesion is now classified as a benign tumor, derived from odontogenic epithelial cells and fibrous stroma, without ectomesenquimal component. The aim of this study is to report a clinical case of patient A.M.C., male, aged 49, Caucasian, treated at the LEBU (Diagnóstico, Tratamento e Epidemiologia das Doenças da Cavidade Bucal) Project of the State University of Maringá. Bulging of the mandibular cortical was clinically observed. Radiographically there was an unilocular radiolucent lesion with sclerotic well defined margins. Taking into account these features, the first diagnosis hypothesis was residual radicular cyst, followed by odontogenic keratocyst tumor. The definitive diagnosis was established after histological analysis, which is essential if considering that the exact determination of the microscopic variations of this entity is closely related with its different recurrence rates and aggression. Considering the pathologist's judgment and the clinical and radiographic findings, the diagnosis of odontogenic keratocyst tumor was established.

## 080 Enterococcus faecalis infected dentin: *in vitro* vs *in vivo* findings

VELOSO, M.V.N. (Marcio Vinicius Nunes Veloso marcio_vnv@hotmail.com); BRAMANTE, C.M.; BERNARDINELI, N.; GARCIA, R.B.; MORAES, I.G.; ZAPATA, R.O.

The aim of this study was to compare the infection pattern of Enterococcus faecalis infected dentin under laboratory conditions and in a canine model. Five cylindrical dentin specimens were infected with Enterococcus faecalis in BHI for 21 days. For the *in vivo* infection model, five maxillary incisors in one dog were inoculated using 50 μL of a Enterococcus faecalis HI suspension. After the experimental period, the specimens were sectioned using an Isomet saw, stained with acridine orange and analyzed by confocal laser scanning microscopy (CLSM). CLSM analysis of laboratory infected dentin showed no or sparse colonization of the root canal walls. In vivo infected dentin was characterized by severe colonization of root canal walls by bacterial biofilms. In all cases, the presence of bacteria in the dentinal tubules, *in vitro* or *in vivo*, was a common finding. It was concluded that *in vitro* infected dentin differed from *in vivo* infected dentin.

## 081 Effects of different posts and restorative techniques on fracture resistance and failure mode of structurally compromised incisor roots

QUEIROZ, M.V.M. (Marco Vinicios Martins Queiroz marcoviniciosqueiroz@hotmail.com); SILVA, G.R.; SANTOS-FILHO, P.C.F.; CABRAL, F.C.; MARTINS, L.R.M.; SOARES, C.J.

The enlargement of root canal increases the risk of dental fracture and the influence of techniques and materials employed to restore these teeth is not yet clear. So, this study investigated the effects of different post systems and restorative techniques on the fracture resistance and failure mode of structurally compromised bovine incisor roots. Ninety bovine roots were endodontically treated and divided into 6 groups (n=15). The guttapercha was removed resulting in a root canal with 10 mm width and 0.5 mm in diameter and a ferule with 0.5 mm thickness and 2 mm height was prepared. The roots of two reference groups were restored with cast post and core – CPC(G1) and fiberglass post – FGP(G2). In the other groups, root canals were overflared 9 mm deep, resulting in a canal with 3.5 mm diameter, and were restored with: CPC(G3), FGP(G4), FGP and accessory FGP(G5) and FGP rebased with composite resin(G6). The resin cement was used for luting procedures and the core was made with composite resin. All teeth were restored with complete metal crowns. Mechanical fatigue was performed with 3x105 cycles of 50 N. The fracture resistance (N) was measured at 135o. The data were analyzed with a 2-way ANOVA (2X2) comparing post systems (CPC or FGP) with the characteristics of root followed by the Tukey's test. After, one-way ANOVA and Tukey's test were employed to compare the types of restorative techniques. Failure mode was classified as favorable or catastrophic. The results were (N): G6:949.8±210.6A; G1:859.9±199.3A; G5:842.6±174A; G2:627.1±119.9B; G3:625.3±164.3B; G4:620.2±164.2B. Statistical analysis showed that CPC decreases fracture resistance and increase catastrophic failures in weakened roots. Fiberglass post associated with composite resin or with accessory fiberglass posts seems to be more indicated as an alternative to cast post and core in weakened roots because of the lower risk of catastrophic failures.

## 082 Temporomandibular muscle dysfunction: diagnosis, etiology and treatment

PALONE, M.R.T. (Marcos Roberto Tovani Palone marcos_palone@hotmail.com); DANELON, L.B.F.; HADDAD, M.F.; PESQUEIRA, A.A.; GOIATO, M.C.

This study conducted a literature review to indicate the means for diagnosis, main etiologies, types of temporomandibular muscle dysfunction and their respective treatment options. The literature review comprised papers in English, Spanish and Portuguese languages, available on the Pubmed and Scielo databases. The temporomandibular dysfunctions (TMDs) may be of articular or muscle origin. The TMDs of muscle origin may be diagnosed by a detailed clinical examination, comprising physical examination with palpation of articulations and muscles of the head and neck. These tmds have a multifactorial etiology, involving predisposing, precipitating and perpetuating factors. They may be classified as protective co-contraction; local muscle sensitivity; myospasm; myofascial pain; centrally mediated chronic myalgia; and fibromyalgia. The therapeutic options include the placement of occlusal plates, massage, physical therapy, ultrasound, laser, electric currents, and application of botulin toxin.

## 083 Relationship between subgingival microbiota and microbial contamination in saliva

ARRUDA, M.C.V. (Maria Cristina Viana Arruda arruda_cristina@yahoo.com.br); SANGALLI, J.; GAETTI-JARDIM JR, E.

FAPESP 2007/51016-3 and 2007/54851-0

The evaluation of subgingival microbiota associated to periodontal diseases constitutes important strategy in the determination of risk of attachment loss. However, in epidemiological studies, the collection of saliva is faster and safer than sampling of subgingival biofilm. Thus, the present study evaluated the reliability of resting saliva on the determination of microbiota associated with subgingival biofilm. Occurrence of 14 periodontopathogens in resting saliva and subgingival biofilm from subjects with different periodontal conditions was evaluated through PCR and real-time PCR by using specific primers and probes for each microorganism, verifying similarities and discrepancies in the prevalence of these microorganisms in the two environments. Samples of resting saliva and subgingival biofilm were collected from 510 subjects, irrespective of their periodontal conditions, aged from 6 to 96 years old. In this group, 159 were gingivitis patients, 88 were chronic periodontitis patients, and 83 children or adolescents were periodontally healthy or gingivitis patients. The comparisons in the prevalence of selected pathogens and clinical periodontal parameters between groups were performed by mean of Kruskal-Wallis test. Pairwise comparisons were performed by mean of Bonferroni adjusted nonparametric Mann-Whitney test, while differences in the occurrence of selected microorganisms were evaluated by weighted least square method. The results evidenced saliva was not reliable on determination of prevalence of P. gingivalis, A. actinomycetemcomitans and T. forsythia from periodontally healthy young people and children, but its reliability is significant in the determination of subgingival microbiota of periodontitis patients and adults. For the other commonly detected periodontal microorganisms, such as P. intermedia, F. nucleatum, C. rectus and T. denticola, microbial levels were deeply related to those observed in specimens from subgingival biofilm, irrespective the age and periodontal conditions.

## 084 Digital certification of electronic documents in Dentistry: what patients and professionals should know

MADEIRA, M.F.C. (Maria Fernanda Conceição Madeira maria.madeira@usp.br); OLIVEIRA, C.; CAPELOZZA, A.L.A.

The use of electronic files and digital images has increased noticeably in Dentistry. Professionals from different specialties have experienced the benefits of the virtual archiving of documents in contrast to films and paper, such as: faster acquisition and manipulation of the images, improvement in the management of data and better use of the office space. Additionally, the wide access of the population to computer-assisted technology has driven dentists to apply digital technology to their offices, in order to meet the expectations of their clients. However, many professionals and clients are not aware about legal validity, possibility of fraudulent manipulations, and warranties of authenticity of digital documentation. Thus, this work aims to clarify and divulgate a chain of digital certification, which aims to warrant the authenticity and legal integrity of electronic documents, explaining how it works and highlighting relevant aspects to patients and professionals.

## 085 Psychological and dental aspects of non-nutritive sucking habits

VIANNA, M.F.O. (Maria Fernanda de Oliveira Vianna mariafernanda_1101@hotmail.com); MARTINS, C.M.; SALLES, C.L.F.

Habit can be understood as the disposition acquired by frequent repetition face to an act, use or customs. The objectives of this paper were to investigate the physiological and pathological aspects of non-nutritive sucking habits, evaluating the possible psychological factors and dental consequences of these habits. A literature review was done about the habit of the pacifier use, bottle-feeding and thumb-sucking regarding the etiology of the habit, its consequences and the best way and time for removing it. Suction is a primitive reflex and essential for the survival of children, involving emotional, psychological and organic elements. Therefore, nutritive sucking is extremely important for the child's ood orofacial and affective development, but non-nutritive sucking habits lead the child to some changes in his/her behavior and have negative effects to the stomatognathic system. It is important to satisfy the child's “hunger of suction”, but we should avoid that harmful habits are acquired because their consequences can be very serious and the future treatment is complicated.

## 086 Masticatory ability of complete denture wearers

DELLA VECCHIA, M.P. (Maria Paula Della Vecchia paulavecchia@yahoo.com.br); ROMANELLA, D.C.; REGIS, R.R.; SOUZA, R.F.

The masticatory ability (MH) of complete denture wearers is an important aspect of life quality related to oral and systemic health, and can be influenced by factors like ridge height. The aim of this study was perform a preliminary survey on masticatory ability of conventional complete denture wearers through a subjective approach and analyze the association degree among this variant and the ridge height. Through a questionnaire translated from an English version, the masticatory ability was evaluated in 15 patients treated with bimaxilary mucous complete dentures. The mandibular bone height was measured (mm) using the patient's panoramic radiograph obtained before the prostheses installation, in the following regions: 10 mm from symphysis, on the mental foramen and between the mental foramen and mandibular ramus. All the measurements were taken by the same examiner and the medium of both sides was used for the final analysis. An index of masticatory problems was obtained by counting of the negative meaning answers attributed to questions 1 to 3, 5 to 8, 10 and 11. From 0 (any problems) until 11 (extremely impaired MH), the median and mode were 5.5 and 5, respectively. The mean ridge height was 31±8mm. The Spearman correlation coefficient between MH and the mean ridge height was weak and without statistical significance (r=-0.189, P=0.500). It was concluded that there is no relation between the subjective perception of MH in conventional complete denture wearers and the ridge height. A great variance was found for MH, although the clinical similarity among the studied cases. It points to an important influence of the subjective factors, in contrast with the commonly observed normative parameters.

## 087 Occurrence of *Helicobacter pylori* in the oral cavity of native Brazilians

RIBEIRO, C.P. (Clícia Pereira Ribeiro cliciaribeiro@foa.unesp.br); HILDEBRAND, M.C.; CIESIELSKI, F.I.N.; VIEIRA, E.M.M.; GAETTIJARDIM JR, E.

FAPESP 2007/51016-3

The periodontium has been suspected as an extra-gastroduodenal reservoir for *H. pylori* infection and transmission, but conflicting evidence exists about the occurrence of this microorganism in pre-Colombian societies that maintain strong relationship with the ancestors' traditions. Thus, the aim of this study was to determine the occurrence of *H. pylori* in the subgingival biofilm of 100 native Brazilians from Umutina Indian Reservation, Mato Grosso State, with different conditions of periodontal health, in comparison to a group of non-Indians presenting similar periodontal and socioeconomic status. Relevant dental and periodontal parameters and general health parameters, were recorded. Samples of subgingival biofilm were collected and the presence of H. pylori DNA was evaluated through nested PCR. The second PCR was performed using 5 ìL of the first PCR reaction. Primers for both rounds of PCR were designed to amplify *H. pylori*-specific segments within the bacterial 16S rDNA. The primers used in first-round PCR were EHC-U and EHC-L, while in the second round of amplification, primers ET-5U and ET-5L were used, producing a 230-bp fragment within the amplicon of the first-round PCR. In Indians, this bacterium was detected in subgingival biofilm from 21.43% healthy subjects, 33.33% gingivitis patients and 34.21% periodontitis patients. In periodontal specimens from non-Indians, the presence of *H. pylori* was observed from 32.65% healthy subjects, 26.67% gingivitis patients and 38.46% periodontitis patients. This confirms the role of oral cavity as a reservoir of the microorganism and it was not found any significant difference between the occurrences of this rod from Indians and non-Indians, despite of deep differences observed in the diet and personal habits of these communities, and this microorganism did not evidence any association with periodontal status of the patients.

## 088 Treatment of infrabone defects associated with multiple recessions

CRIVELENTI, M.D. (Mariana Dassie Crivelenti mdcrivelenti@yahoo.com.br); REINO, D.M.; GRISI, M.F.M.; SOUZA, S.L.S.; MAIA, L.P.

The application of enamel matrix proteins (EMD) (Emdogain) promotes clinical and histological benefits on regenerative treatment of infrabone defects. However, in large defects, its semi-fluid consistency prevents the maintenance of space for bone deposition. The combination of EMD with a graft material can solve this problem. The mixture of â-tricalcium phosphate and hydroxyapatite (â FTH) (bone ceramic) has been indicated for clinical use, due to good stability and porosity. The present clinical case has the purpose to show the use of EMD with â FTH in the treatment of infrabone defects associated with multiple recessions. A male patient, 71 years old, attended the clinic of the Periodontics of FORP - USP for periodontal treatment and the presence of infrabone defects and multiple gingival recessions in upper posterior teeth was detected. After basic periodontal treatment, a regenerative treatment was planned. In the upper right sextant, a full-thickness flap was elevated through the mucogingival line to provide access for debridement of the defects and root scraping and smoothing. Upward mucogingival line, a split-thickness flap was made for later coronal slice. Root conditioning with 24% EDTA, neutral pH, was performed according to the manufacturer's instructions. Emdogain was applied on the roots and Emdogaim associated with bone ceramic was used to fill the defects. The flap was pulled coronally and sutured. Clinical and radiographic 6-months controls showed a reduction on probing depth and height of recessions. In conclusion, periodontal defects with favorable characteristics can be successfully regenerated using EMD associated with â FTH, in addition to the capacity of the material to support the root coverage.

## 089 Implementation of measures and actions to promote oral health during pregnancy

NAGATA, M.E. (Mariana Emi Nagata marieminagata@hotmail.com); HIDALGO, M.M.; SALLES, C.L.F.; MACIEL, S.M.; SERAFIM, D.; FRACASSO, M.L.C.

The study evaluated the behavior and habits of pregnant women in relation to the oral health, as well their knowledge about the most important oral problems. Pregnant women (n=79) assisted by the extension project “Treatment of low risk pregnant women” of the Specialties Outpatient Service of the Regional University Hospital of Maringá” participated of the study. The data were collected with a semi-structured questionnaire, focusing on the main aspects of oral health (caries, periodontal diseases and extension of the interference of myths related dental treatment). The obtained results, presented as percentages, showed that 65.82% of the pregnant women ate between the meals, of which 48% ate fruits and the others ate cariogenic foods. There was no correlation between the ingestion frequency and the daily brushing frequency. It was observed that 78% of the participants had already used fluoride, but 53% did not known its function. Considering the whole sample, 39% declared that caries is not transmissible and, when were asked about its etiology, 60% mentioned poor oral hygiene and 2.53% associated it with pregnancy. About the etiology of gingivitis, 37% related it with poor oral hygiene, 4% with pregnancy and the majority of them (59%) did not know the answer. These data demonstrate the importance of dentistry in the prenatal multidisciplinary assistance offered to pregnancy women, including educational, prevention and restorative measures, in the benefit of general health.

## 090 Occurrence of yeasts of the genus Candida in the oral cavity of female drug users

AZUMA, M.M. (Mariane Maffei Azuma mariane_azuma@hotmail.com); LINS, S.M.; GAETTI-JARDIM JR, E.

Although the involvement of yeasts of the genus Candida in the oral microbiota of immunocompromised patients has been continuously studied, the literature on the effects of licit or illicit drugs on this important group of microorganisms is scarce, being restricted to the effects of tobacco and alcoholism. In individuals with immunological impairment these fungi may proliferate and produce severe infections, particularly on mucosal surfaces. This study aimed to evaluate the presence of Candida sp., Candida albicans, C. tropicalis, C. parapsilosis and C. glabrata in samples obtained from oral mucosa, saliva, biofilm sub and supragengival of 50 female patients attended in a detoxification program for drug users in the city of Santa Fe do Sul, in comparison with a group of never-user females presenting similar socioeconomic conditions and age. Presence of yeasts of the genus Candida was evaluated by amplification of DNA by PCR and “semi-nested” PCR, using primers and conditions specific to each infectious agent. Fungi of the genus Candida was present more homogeneously distributed in the mucosal and supragengival biofilm and when the data are analyzed in relation to periodontal condition of patients examined, irrespective of the existence of chemical dependence, it appears that, in samples of saliva, C. albicans was more frequent among patients with gingival bleeding and gingivitis. The presence of C. albicans in saliva samples, regardless of chemical dependency or not, was linked to severe gingivitis (Mann-Whitney test, P = 0037). Among the factors that further exacerbate the oral colonization by these yeasts are highlight smoking, alcoholism and the presence of complete or partial dentures. Thus, it is possible that the high frequency of detection of these fungi, particularly in patients with chemical dependency, will reflect the direct effects of drugs on the mucosa and, quite possibly, on the cellular immune response associated with mucosal surfaces.

## 091 Subepithelial connective graft associated with lateral flap for covering and increase of the keratinized gingiva: report of a case

LONGO, M. (Mariéllen Longo mary.lonngo@hotmail.com); FERNANDES, L.A.; MURAKAWA, A.C. MARTINS, T.M.; GARCIA, V.G.; BOSCO, A.F.

Gingival recession cause root exposure and consequent symptoms such as hypersensitivity, caries, abrasions and changes in the esthetic pattern. For its treatment, coverage techniques have been employed among which is the subepithelial connective tissue graft associated with laterally positioned flap. The purpose of this study is the presentation of a clinical case of patient M.A., 37 years old, who sought the Postgraduate Periodontics Clinic of Araçatuba Dental School reporting discomfort caused by lowering of the gingival margin and hypersensitivity in teeth #46 and #45. The intraoral examination revealed Miller's Class III gingival recessions in teeth #46 and #45 probably because of occlusal trauma. The technique of choice for treatment of the patient was subepithelial connective tissue graft associated with laterally positioned flap. Before the periodontal surgery, root canal treatment was done in tooth #46. Within a45-day postoperative period, it was observed partial coverage of root exposure and increase of the keratinized gingiva width that solved the patient complaint.

## 092 Evaluation of root canal filling performance of four different techniques

SILVA, M.A.M. (Marina Angélica Marciano da Silva marinangelica@usp.br); DELGADO, R.J.R.; BRAMANTE, C.M.; MORAES, I.G.; GARCIA, R.B.; BERNARDINELI, N.

The aim of the study was to evaluate the percentage of voids, guttapercha and root canal sealer present after the filling procedures using four different techniques. Fifty-two extracted maxillary lateral incisors teeth were cleaned and shaped using the Oregon's technique. The teeth were randomly divided in four groups, according to the filling technique used (n=13). Lateral condensation (LC); Tagger's hybrid (TH); MicroSeal (MS) and GuttaFlow (GF). Horizontal cross-sections of each tooth were made at the 2, 10 and 15 mm level from the apex. Digital images of the root canal areas were acquired using a stereomicroscope and evaluated using the Image Tool 3.0 software. Nonparametric statistical analysis was performed using the Kruskal-Wallis test (p< 0.05). In general, the gutta-percha filled area decrease significantly at the apical level in all the evaluated techniques (p<0.05). Also, all the filling techniques showed an increment in the sealer area at the apical level (p<0.05). Regarding the presence of voids, there was not statistical difference (p>0.05) among the techniques at the apical level. There was not a relationship between voids and the apical level of the filling except for the GuttaFlow technique that showed an increment of voids at the coronal third (p<0.05). MicroSeal and Tagger's Hybrid showed higher percentage of gutta-percha at the coronal and middle thirds. On the other hand, lateral condensation presented the highest percentage of gutta-percha at the apical third. However, a statistical difference (p>0.05) was not found. From the results of the present study it can be concluded that the quality of the filling decrease at the apical level independently of the filling technique used. Microseal and Tagger's hybrid performed higher gutta-percha filled area than the lateral condensation and Guttaflow technique at the coronal and middle third level (p<0.05). The presence of voids was similar among the evaluated techniques.

## 093 Anterior open bite treatment with Nogueira® lingual bonded spur and high-pull chincup: a clinical case report

HEGG, M.P. (Marina de Paula Hegg marinahegg@yahoo.com.br); CASSIS, M.A.; ALMEIDA, R.R.

Anterior open bite is defined as a negative overbite between the incisal edges of the maxillary and mandibular teeth with the posterior teeth in occlusion. In the mixed dentition, the prevalence of open bite can reach 17% and it is four times higher in people with facial hyperdivergency and sucking habits than in the normal subjects. The treatment has a better prognosis and greater stability in the mixed dentition. The Nogueira® lingual bonded spur appears as a viable alternative for the treatment of this malocclusion and for a better vertical control, it can be used in association with high-pull chincup therapy. In view of the mentioned aspects, the aim of this study was to review some concepts regarding anterior open bite, with the presentation of a clinical case.

## 094 Fluoride levels in water supply in Mozambique municipalities

MAPENGO, M.A.A. (Marta Artemisa Abel Mapengo martamapengo@hotmail.com); MARSICANO, J.A.; MOURA, P.G.; SALES-PERES, A.C.; SALES-PERES, A.; SALES-PERES, S.H.C.

This study aimed to analyze the fluoride concentration in water supply of each province of Mozambique (2008) and relate to the latest data available (1978), considering that Mozambique has a natural system of fluoridation in the water supply. Eleven samples were collected in duplicate, directly from taps in homes of their municipalities. The concentration of fluoride from water samples was determined in duplicate, using the ion sensitive electrode (Orion 9609), coupled to a potentiometer (Procyon, model 720). Data analysis was the descriptive analysis, performed using Student's t-test, adopting a significance level of 5%. The fluoride concentrations found in 1978 and 2008 were: Cabo Delgado (0.62 and 0.28 ppm), Niassa (0.18 and 0.98 ppm), Nampula (0.18 and 0,095ppm), Zambezia / Quelimane (0, 81 and 0.37 ppm), Tete / city (1.00 and 0.97 ppm), Sofala / Beira (0.00 and 0,01ppm), Manica (0.04 and 0,01ppm), Inhambane / city (0.00 and 0.05ppm), Gaza (0.01 and 0.06 ppm) and Maputo city (0.23 and 0.36ppm), respectively. The mean of fluoride concentration found in this study (2008) was 0.33ppmF, while the mean of the previous data (1978) was 0.31ppmF. There was no significant reduction (p = 0.83) between the concentrations of fluoride in water from 2008 and 1978 (p> 0.05). Data obtained from 1978 in the Tete province river detected 5.50 ppmF. In this province there was found high prevalence of dental fluorosis, although it was been made the defluoretation in recent years. It is concluded that there was no change in the level of fluoride in the water supply of the main municipalities of Mozambique. Fluoride concentration in water supply must be standardized in order to reduce dental caries prevalence without increase dental fluorosis prevalence in the region.

## 095 Esthetic clinical crown lengthening: review of literature and case report

MANTOVANI, M.B. (Matheus Bortoluzzi Mantovani m_mantovani1@hotmail.com); TEIXEIRA, B.M.; NISHITA, P.; SILVA, C.O.

Some studies discuss the manipulation of the gingival exposure to promote a esthetically pleasant smile. Recently, more attention has been given to the problem of severe gingival exposure and to the potential of the periodontal plastic surgeries in improving the smile line. In this work, a literature review about the subject “esthetic clinical crown lengthening” is done and two clinical cases using different treatment techniques are present. Although the majority of the people present a smile line within the average standards, there are those who present a “gummy smile”, which can become a problem for the person. If the excess of displayed gingival is related with insufficient clinical crown length, the best procedure is gingivectomy/gingivoplasty associated with an osteotomy. Some factors must be considered for these crown lengthening procedures, such as localization of the gingival margin in relation to the cementoenamel junction, the bone crest and the crown-root-alveolar bone relationship. In the first case, patient G.A.S., 20 years old, female, Caucasian, dental student, complained of having short teeth. After clinical and radiographic examination, the patient was subjected to a single-procedure of gingivectomy. In the second case, patient F.L.P., 21 years old, female, student, who also complained to present short anterior teeth, needed gingivectomy associated with osteotomy. Neither of the patients presented recurrence after the treatment. Esthetic clinical crown lengthening brings innumerable benefits to the patients and the indication of the best technique for the solution of each case allows the long-term success of the treatment.

## 096 Esthetic reestablishment of an anterior fractured tooth with composite resin

SPIN, M.D. (Mauricio Donalonso Spin mauriciospin@hotmail.com); MODENA, K.C.S.; NAHSAN, F.P.S.; REINATO, J.V.D.; SILVA, L.M.; FRANCISCONI, P.A.S.

The oral cavity is an extremely important and dominant segment on the dentofacial esthetic composition of a person in where anterior teeth play a fundamental role. From the cosmetic point of view, teeth appearance value is of great importance on the society. Reestablishment of oral health associated to esthetics aims to provide a beautiful and esthetic smile with shape, color and harmonious proportion. Loss of this harmony, due to anterior dental trauma, may cause an altered psychological profile such as difficulties to establish relationships and excessive shyness. Adequate restoration of a fractured anterior tooth is a constant concern of clinicians. The development of various composites, with enhanced optical and mechanical properties, associated to a better understanding of dental tissues performance to light incidence, offered an artistic approach on the execution of direct resin restorations. This case report aims to describe the reconstruction of an anterior fractured tooth (#21) with composite resin. A clinical sequence of composite insertion will be presented where both enamel and dentin will be reconstructed in their original thickness. Fractures of anterior teeth are clinical situations that demand scientific knowledge, technical ability and artistic sense for a successful treatment, which has the adhesive technology as a rapid and conservative alternative with excellent esthetic results.

## 097 Odontogenic dental features of cleidocranial dysplasia: case report

LANCIA, M.(Melissa Lancia melissalancia@gmail.com); GARIB, D.G.; LARA, T.S.; SILVA FILHO, O.G.; BERTOZ, F.A.

Cleidocranial dysplasia consists in a rare congenital bone malformation with dominant autosomal genetic etiology. This malformation is characterized by various degrees of clavicle hypoplasia, the patients tend to present low stature, a pronounced frontal and parietal procumbence and a delay in the cranial bone ossification. The dental features include a delay in primary tooth exfoliation, eruption delay or retention of permanent teeth, and the presence of multiple supernumerary teeth. This study aimed, by means of a clinical report, to describe the features of cleidoclanial dysplasia of interest to dentistry. In the present patient, maxillary anterior primary teeth were extracted for allowing traction of the permanent incisor. It is highlighted the importance of the dentist recognizing the clinical and radiographic dental features of cleidocranial dysplasia for an adequate treatment plan and familial counseling.

## 098 Occurrence of *Porphyromonas gingivalis* and *Prevotella intermedia* in the oral microbiota of patients subjected to radiotherapy for treatment of head and neck malignant tumors

MELO, M.E. (Moriel Evangelista Melo ieielmelo@hotmail.com); GERALDES, A.M.; GAETTI-JARDIM, E.J.

Radiotherapy is employed for the treatment of head and neck cancer with favorable results. However, the side effects of radiation on salivary glands and other oral tissues may create favorable conditions for the establishment and proliferation of oral microorganisms. This investigation evaluated the occurrence of *Porphyromonas gingivalis* and **Prevotella intermedia** in the oral microbiota of patients undergoing radiotherapy for treatment of head and neck cancer. After clinical examinations, samples of saliva, dental biofilm and surfaces of oral mucosa were collected, and this procedure was performed at different moments during treatment, as well as 6 months after completion of radiotherapy. The presence of these anaerobes was carried out through culture using Brucella agar supplemented with hemin, menadione, and horse blood, incubated under anaerobiosis, at 37°C for 14 days, and by PCR. It was verified that populations of these anaerobes increased significantly after and during radiotherapy and the occurrence of *P. gingivalis* varied from 35.71% gingivitis patients to 69.23% periodontitis patients. The subjects harboring *P. gingivalis* evidenced 2.08. 105 CFU/DNA-copies in the samples before radiotherapy and increased to 6.3 105 CFU/DNA-copies after radiotherapy. In regard to *P. intermedia*, it was cultivated from 21.43% of gingivitis patients and from 40 % periodontitis patients, before radiotherapy, and its detection by PCR varied from 28.57% to 69.23% in these same samples. Six months after conclusion of the treatment, this anaerobe was observed from 44.44% gingivitis patients and from 80.0% periodontitis patients. The results suggest that xerostomia and other changes in the oral cavity associated with radiotherapy may collaborate with the proliferation of these strict anaerobes.

## *In situ* evaluation of the remineralizing efficacy of a fluoridated foam with neutral pH

MANARELLI, M. M. (Michele Mauricio Manarelli mi_manarelli@hotmail.com); DANELON, M.; DELBEM, A.C.B.; VIEIRA, A.E.M.; BRIGHENTI, F.L.

Fluoride foam offers a lower risk of fluoride (F) ingestion due to its consistency and because lesser amounts of the product is necessary to the application. The F kinetics of this product can interfere on enamel reactivity. However, there are no studies evaluating the ability of fluoride foam on remineralizing carious lesions. The aim of this study was to evaluate the ability of a neutral fluoride foam on remineralize incipient carious lesions. Enamel bovine blocks were selected by analysis of surface microhardness (SMH) and randomized in three groups: 1) control group (no treatment); 2) treatment with neutral fluoride gel (2% NaF, DFL) and 3) treatment with neutral fluoride foam (1.23% F, FGM). The products were applied at the beginning of each phase. Ten volunteers used a palatal appliance containing four enamel blocks during three days. A wash-out period of four days was allowed between each phase. Two blocks were removed 30 min after the treatment to analyze calcium fluoride (CaF2) formed. The two blocks remaining were used to analyze SMH and CaF2 retained. The amount of CaF2 formed and retained were higher for the fluoride foam groups than for the fluoride gel group. It can be concluded that there are no differences in remineralizing incipient carious lesions after the use of fluoride foam when compared to fluoride gels.

## 100 Dental trauma: tooth fragment reattachment and prevention of recurrences with use of mouthguard

MUNDIM, M.P.C. (Michelle Pereira Costa Mundim michellemundim@hotmail.com); MARTINS, C.; SILVA, G.R.; MARTINS, L.R.M.; CABRAL, F.C.; SOARES, C.J.

Dental trauma is an injury promoted by extrinsic factors being one of the most frequent causes of tooth loss or esthetic and functional damages. This work presents a case of dental trauma, addressing the tooth fragment reattachment technique and the importance of mouthguard use in the prevention of recurrences. A 12-year-old male patient came to the Dentoalveolar Trauma Clinic of the Dental School of the Federal University of Uberlândia, with a crown fracture in tooth #1 occurred during sporting activities. After indirect pulp capping with calcium hydroxide cement and glass ionomer cement, the coronal fragment was bonded to the tooth remnant with adhesive system and resin cement. The restoration was completed with composite resin and functional adjustments were done. In order to prevent future dental traumas, a 2-mm-thick silicone mouth guard was fabricated. The mouthguard prevents injuries to the soft tissues and can reduce and distribute the stresses resulting from direct and frontal impacts. When the fractured fragment is available, the tooth fragment reattachment technique is an efficient, conservative and low-cost treatment alternative that can recover, the tooth shape, esthetics and function. The use of mouthguards during engagement in sporting activities is of paramount importance because they reduce the risk of fractures and recurrences.

## 101 Glass-ionomer cement: update in clinical application

MICHIELIN, M.B. (Martha Beteghelli Michielin ma_angevil@hotmail.com); NAHSAN, F.P.S.; GOMES, O.S.; MONDELLI, R.F.L.; ISHIKIRIAMA, S.K.; WANG, L.

Since its introduction to Dentistry, glass ionomer cements (GICs) have shown promising perspectives. This category of dental material comprehends advantageous properties such as fluoride release, bonding to dental substrates, biocompatibility and similar coefficient of thermal expansion to that of the tooth structure. All these properties make GIC a versatile material that is clinically indicated for restorative, luting or lining procedures. The purpose of the present study was to explore the abilities of GICs and present new perspectives based on the modifications proposed since its introduction.

## 102 Influence of nicotine and ovariectomy on healing process of autogenous bone block grafts: a histomorphometric study in aged female rats

SANTOS, M.R. (Murillo Rezende Santos murillo_rs@hotmail.com); BOSCO, A.F.; MORAES, R.O.; BONFANTE, S.; LUIZE, D.S.; MURAKAWA, A.C.

Estrogen deficiency and cigarette smoking are among the factors that may affect the repair of bone grafts. The aim of this study was to perform qualitative and quantitative analyses of the effect of ovariectomy and nicotine on autogenous bone block grafts in aged rats and to describe events of the initial healing. Thirty-six 12-month-old female Wistar rats were randomly divided into 2 groups according to treatment. The experimental group underwent ovariectomy surgery and daily applications with nicotine hemisulfate, while the control group underwent sham ovariectomy surgery without ovary removal and daily saline solution applications. After 30 days, both groups received an autogenous bone block graft harvested from the calvaria on the angle of the mandible. The animals continued receiving the solutions until the moment of sacrifice at, 7, 14 or 28 days postoperatively. The histological analysis in the experimental group showed a delay in the osteogenic activity at the graft-recipient bed interface as well as the decrease in granulation tissue organization. The specimens of the experimental group exhibited less new bone formation, which was poorly cellularized and vascularized. The statistical analysis revealed significantly less bone formation in the experimental group at 14 days (40.92% versus 57.41%) and 28 days (53.09% versus 68.35%). In conclusion, estrogen depletion and the systemic influence of nicotine, although did not prevent, delayed the healing process of autogenous bone block grafts in aged rats.

## 103 Root canal treatment: a new perspective

LEONARDO, M.F.P. (Mário F. P. Leonardo marioctba@hotmail.com); LEONARDO, R.T.L.; PALO, R.M.; IGLECIAS, E.F.

The fast technological evolution of endodontic looks for faster and more efficient instrumentation. Besides this, the other objective is to diminish stress for both professionals and patients. It is necessary to know new options of instruments, as well as the combination between them in order to prepare, clean and sanitize the internal anatomy, which does represent a uniform cone, but rather a complex root canal system, with flattening and curvatures. It requires a proper protocol of instrumentation. The objective of cervical third preparation is to promote widening and tapering, and thus rotary systems appear as an interesting option. In the middle third, due to its anatomy, the indication is to work with oscillatory systems by using stainless steel files with small tip sizes and varied tapers. Finally, in the apical portion, we recommend manual Ni/Ti files, respecting the root anatomy and creating a great apical stop. By associating these systems, routine endodontic treatment becomes easier and efficient in addition to saving the instruments for a longer time. This protocol also decreases patient stress and improves the professional quality of life.

## 104 Effectiveness of the low level laser therapy on the postoperative edema and pain following secondary bone graft

CUNHA, M.J.S. (Mércia Jussara da Silva Cunha merciacunha@hotmail.com); NATALICIO, G.L.; OLIVEIRA, P.G.F.P.; FIAMENGUI FILHO, J.F.; ALMEIDA, A.L.P.F.

The present study aimed at evaluating the effectiveness of low level laser therapy on the postoperative pain and edema of humans subjected to secondary bone graft. Sixty-one consecutive subjects (34 males and 27 females), with age ranging from 9 to 15 years old, presenting unilateral cleft lip and palate and subjected to secondary osseous graft were selected and divided into 2 groups. The experimental group was comprised by 31 patients irradiated with low level laser, dose of 4J/cm2, power 15mW and wave length 780nm, 10 seconds per site. On the placebo group (30 patients) LED was applied during 60 seconds. Both groups received 10 applications immediately, 24 and 48 hours after the surgery. Pain and edema were recorded in each application session and on the fifth day after the surgery. Statistically significant differences were not found between groups regarding pain and edema. Gender was also found non significant. In conclusion, the laser therapy, at least with the settings adopted, did not bring significant benefits in terms of pain and edema to the unilateral cleft lip and palate patients subjected to secondary bone graft. Further investigations are encouraged using different methods in order to verify the real role of this tool on the postoperative pain and edema of secondary bone graft interventions.

## 105 Use of antimicrobial agents into reline materials as a therapeutic modality for denture stomatitis

BUENO, M.G. (Mírian Galvão Bueno miroca@usp.br); URBAN, V.M.; CAMPANHA, N.H.; JORGE, J.H.; ALMILHATTI, H.J.; NEPPELENBROREK, K.H.

Denture stomatitis is the most common form of oral candidal infection with overall incidence of about 65% of otherwise denture wears. Tradition management of this type of infection consists of topical application of an oral antifungal agent and correction of the existing denture adaptation with temporary reline material. Nevertheless, the success of topical application of drugs in the oral cavity may be compromised by some factors, such as the absence of the discomfort caused by the infection, the medication expenses for an unperceived need, the continuing use of the dentures during the medication, the unpleasant taste, and the frequency of dosage. Moreover, it is impossible to maintain the effective concentration in the infected location due to saliva and deglutition, which quickly dissolve and eliminate any drug from the oral cavity. It has been reported that combining antimicrobial agents into tissue conditioner can be used in the treatment of denture stomatitis. This treatment can reduce the application frequency, the limited compliance of patients and the cost of the treatment since only a fraction of the antifungal agent is used compared to conventional therapy. Furthermore, the action of the drug becomes prolonged, enabling tissue recovery and providing amore predictable therapeutic outcome. Tissue conditioners combined with antifungal agents also allow simultaneous treatment of injured denture bearing tissue and candidal infection. The incorporation of antifungal agents such as miconazole, ketoconazole, chlorhexidine, ?uconazole, amphotericin B and nystatin into reline materials has been shown to be effective and viable in some *in vitro* and *in vivo* studies. It has been demonstrated that this treatment can be useful in inhibiting the growth of C. albicans growth. The purpose of this study was to overview the possibility of the use of antimicrobial agents into reline materials as a therapeutic modality for denture stomatitis.

## 106 Acellular extrinsic fiber cementum: some characteristics in human and dogs

MISAWA, M.Y.O. (Mônica Yuri Orita Misawa monica_yuri@hotmail.com); ARAÚJO, M.; SUKEKAVA, F.

The periodontal disease in dogs is a disorder that can occur naturally and has many human periodontal disease characteristics. Therefore the dog has been used as an experimental model to study the factors involved in the etiology and pathogenesis of periodontitis, and also to study the regenerative potential of the periodontal tissues after different surgical techniques application. Although there are a great number of studies using dogs, so far there is no work comparing the human cementum with the canine cementum. In fact, little is known about the dog's radicular cement characteristics. However, it is anticipated that an animal experimental model must have a biological behavior similar to that of humans, so there is a direct clinical explanation. The aim of the study was to evaluate the characteristics of the acellular extrinsic fibers of cementum in humans and dogs. 52 premolars, 22 of human about 14 years old and 30 premolars of dogs divided in 3 groups (1 year old, 2 years old and 8 years old) were used. The premolars were carefully extracted and prepared for histological analysis. The acellular extrinsic fiber cementum at the most coronal portion of the root was described and histological measurements were performed. The results demonstrated that the acellular extrinsic fiber cementum in humans and dogs was morphologically similar. It was also showed that the number of attaching collagen fibers per 100 μm of root surface and the cementum growth rate (thickness growth) were similar between both specimens. The present study suggests that the dog may represent an adequate experimental model for the analysis of cementum formation following regenerative therapy.

## 107 Dental age as an adolescence indicator

PARTEIRA, N.J.S. (: Naiara Jordão Souza Parteira na_jordao@hotmail.com); LARA, T.S.; SILVA FILHO, O.G.; BERTOZ, F.A.

The purpose of the current study was to analyze the possible relationship between root formation of the first premolars and some skeletal maturational stages identified in hand-wrist radiographs. This transverse study was defined with panoramic and hand-wrist radiographs of 123 males and 109 females aged between 4 years and 5 months and 17 years and 12 months. The root formation stages of the first premolars were related to the ossification stages of the sesamoid bone, the epiphyseal stages of the thumb phalanx and the epiphyseal stages of the radium. The results showed statistically significant correlation. The roots of the lower first premolars do not reach 2/3 of their length before adolescence.

## 108 Comparative study by the masticatory performance between patients with an without temporomandibular dysfunction

STRINI, P.J.S.A. (paulinne@netsite.com.br); GORRERI, M.C.; FERNANDES-NETO, A.J.; NEVES, F.D.; MENDES, F.A.; BORGES, T.F.

Temporomandibular disorders (DTM) are a set of signs and symptoms that affect the stomatognathic system and adjacent structures, generating mainly a pain able to radiate to inter-related structures. The purpose of this study was to evaluate the severity of TMD using the Helkimo Clinical Index (HCI) and Masticatory Performance (MP) in patients with TMD (n = 9) and compare it to the control group (CG) (n = 15), both with natural dentition and bilateral posterior occlusion. The MP tests were performed with the food simulator “Optocal” in portions of 17 cubes with 5.6 mm side and the subjects instructed to chew it for 20 (C1) and 40 (C2) masticatory strokes, monitored by a single examiner. The material collected was placed in a set of eight coupled granulometric sieves in a decresing order of opening. Then it was performed, sieving, material collection, drying and weighing of the contents of each sieve. It was calculated the geometric mean diameter of the chewed particle (MGD), obtaining a percentage of the MP index. The obtained data were subjected to the parametric Test t Student for independent samples (p <0.05). According to ICH, n = 8 presented severe DTM and n=1 showed light DTM. The results indicated statistically significant differences (p = 0000) both for GC (47.76 ± 8.75 in C1, C2 at 70.27 ± 3.37) and for the TMD group (24.06 ± 12.93 in C1, 49.32 ± 10.77 in C2). We conclude that patients with painful symptoms tend to have its chewing function impaired, requiring a treatment able to restore their physiological functions, improving their chewing ability and promoting their life quality.

## 109 Leiomyoma in mounth: case report

MACEDO, N.S. (Nara Sarmento Macêdo ns.macedo@yahoo.com.br); PAULO, L.F.B.; ROSA, R.R.; DURIGHETTO JÚNIOR, A.F.; CARDOSO, S.V.

Fibroma and lipoma, benign neoplasms of mesechuimal origin, are common in the oral cavity. Leiomyoma, a smooth muscle benign neoplasm, has little expressivity in the mouth, occurring in most cases in the tongue, inferior lips, cheeks and palate. It is characterized as a slowly growing nodule, sessile, smooth surface, with no color alteration, painless, no gender predilection and on the 5th and 6th decade of life. It is uncommon in the retromolar region. Case: Patient CLCB, Caucasian, female, 56 years old. Main complaint: nodule in the retro-molar region with fast growing. Clinical characteristics: sessile nodule, smooth surface, no color alteration, elastic, with 2 cm of diameter about a month and compression painful. Initial diagnosis: mucoepidermoid carcinoma. Incisional biopsy. Immunohistochemistry: smooth muscle actin, vimentin and desmin. Definitive diagnosis: Leyomioma. Treatment: surgical removal. Follow up: 6 months without recurrence. Discussion: The initial diagnosis was based on the fast expansive growing and the retromolar localization. The immunohistochemistry examination was positive for smooth muscle actin and desmin suggesting leiomyoma. Considering the biological behavior, fast growing nodule, the mitoses was counted in 10 microscopic high magnifying fields. If more than 4 mitoses were detected, it should be considered a malignant behavior. There were only 2.6 mitoses in all ten fields.

## 110 Immediate loading of implants in a case of multiple agenesis treated by iliac bone grafting and implant-anchored orthodontics

OLIVEIRA, N.C.M. (Natássia Cristina Martins Oliveira natassiacmo@yahoo.com.br); SARDINHA, M.C.; MARQUEZ, I.M.; FURTADO, L.M.; BATISTA, J.D.; ZANETTA-BARBOSA, D.

Multiple agenesis is an anomaly of dental development not very common on human being. Early diagnosis and proper treatment of this development alteration are essential in order to achieve occlusal, functional and esthetic harmony. The purpose of this study is to describe prosthetic rehabilitation with implants in a case of a male young patient with multiple agenesis. Due to the severe lack of bone, the treatment comprised a bilateral sinus lift and autologous bone grafting of the maxilla and mandible using corticocancellous bone blocks and particulated bone, both from the iliac crest. After eight months, two implants were installed in each side of maxilla, in the previously augmented area, left to osseointegrated for six months and then used as anchorage for orthodontic movements. The occlusion was temporally restored with an adaptation of a removable partial prosthesis, which had been used by the patient since childhood. After orthodontics, one additional implant was inserted in each side of the maxilla as well as four implants were installed in the mandible and an immediate provisional prosthesis was constructed, reestablishing function and esthetics. The association of bone reconstruction, implant-based orthodontic movements and immediate loading of implants appears to be a suitable treatment option for young adult patients with multiple agenesis.

## 111 Effect of demineralizing agents on dental roots surfaces: a scanning electron microscopy study

AMARAL, N.G. (Nathália Godoy do Amaral nathalia.amaral@usp.br); HIRATA, F.; REZENDE, M.L.R.; SANT'ANA, A.C.P.; GREGHI, S.L.A.; PASSANEZI, E.

Dental roots that have been exposed to periodontal disease present superficial and structural changes which inhibit celular insertion. Scaling is not sufficient to make those roots again biocompatible because the resultant smear layer retains bacterial cytotoxic products that justify the use of conditioning agents. Four chemical agents were evaluated according to their capacity for removing smear layer, exposing and widening dentinal tubules. Fifty human teeth extracted due to periodontal disease were scaled and divided in 5 groups: CA Group: demineralization wit citric acid pH1 for 3'; AT Group: demineralization with acid tetracycline for 3'; EDTA Group: demineralization with EDTA at 24% for 3'; PA Group: demineralization with phosphoric acid at 50% for 3'; Control group: burnishing of saline solution for 3'. Fragments retrieved from the treated areas were observed at scanning electron microscopy and the images were analyzed with the aid of a computerized program. The data were compared by Kruskal–Wallis, Dunn and ANOVA tests (for p<0,05). Smear layer was present in 100% of the specimens of Control and PA groups; in 80% of those of the EDTA group; 33.3% of AT group and 0% of CA group. The mean percentage of area occupied by exposed tubules was: AC Group=0.12±0.17%; AT=0.08±0.06%; PA=0.03±0.05%; EDTA=0.01±0.01% and Control=0±0%. Only CA group was statistically different from the other groups, with exception of AT group. The mean amount of exposed tubules within a standardized area was: AT=43.8±25,2; CA=39.3±37; PA=12.1±16.3; EDTA=4.4±7.5 and Control=2.3±5.7. statistically significant difference was present when comparing the AT and Control groups, AT and EDTA, CA and Controls and CA and EDTA. It was concluded that citric acid and tetracycline seam to present greater potential for exposure and widening of dentinal tubules than the other agents. Clinical evaluations relating these properties to potential for collagen fiber reattachment would be focused on further studies.

## 112 A new methodology to quantify the expression of osteocalcin and osteopontin: a study in rat calvaria

CAMPOS, N.(Natália de Campos natidecampos@gmail.com); NAGATA, M.J.H.; MESSORA, M.R.; POLA, N.M.; FURLANETO, F.A.C.; BOSCO, A.F.

A great problem in performing immunohistochemical studies is the lack of objective methods to quantify the data obtained. The use of scores or other subjective methods to evaluate the data may weaken the reliability of the results. The purpose of this study was to evaluate a new method to quantify the expression of osteopontin (OP) and osteocalcin (OC) on bone healing in surgically created critical-size defects (CSD) in rat calvaria. 15 rats were divided into 3 groups: C (control), AB and AB/PRP. A 5 mm diameter CSD was created in the calvarium of each animal. In Group C, defect was filled with blood clot only. In Group AB, defect was filled with autogenous bone graft. In Group AB/PRP, defect was filled with autogenous bone graft combined with 100 ìL of platelet-rich plasma (PRP). One L-shaped mark was made 2 mm anterior and one 2 mm posterior to the margins of the surgical defect. The marks were filled with amalgam. Their purpose was to allow identification of the center line of the original defect during laboratory processing and also to be used as references to locate the original bone margins of the surgical defect during immunohistochemical analysis. Animals were euthanized 30 days postoperatively. OP and OC immunohistochemical staining were performed. OP-positive and OC-positive cells (bone lining cells and osteocytes) were quantified within the confines of the total area of the original defect. Data were statistically analyzed (ANOVA, Tukey, p < 0.05). Group AB/PRP presented a significantly higher number of OP-positive cells than Group C (p < 0.05) and also a significantly larger number of OC-positive cells than Groups C and AB (p < 0.05). Within the limits of this study, it can be concluded that the methodology used led to a more objective and reliable quantitative analysis of the immunohistochemical data.

## 113 Influence of platelet-rich plasma on osteogenesis and resorption of autogenous bone grafts: a histologic and histometric study in rat calvaria

POLA, N.M. (Natália Marcumini Pola nat_pola@hotmail.com); NAGATA, M.J.H.; MESSORA, M.R.; CAMPOS, N.; FURLANETO, F.A.C.; GARCIA, V.G.

It has been suggested that autogenous bone (AB) grafts incorporate faster when combined with Platelet-Rich Plasma (PRP). This incorporation seems to occur as a result of the enhancement of osteogenesis process and resorption of non viable particles of bone grafts. This study histologically analyzed the effect of autogenous PRP on incorporation of AB grafts placed in surgically created critical-size defects (CSD) in rat calvaria. 30 rats were divided into 3 groups: Group C (control), Group AB and Group AB/PRP. A 5 mm diameter CSD was created in the calvarium of each animal. In Group C, the defect was filled by blood clot only. In Group AB, the defect was filled with 0.01 mL of AB graft. In Group AB/PRP, the defect was filled with 0.01 mL of AB graft combined with 50 ìL of PRP. All animals were euthanized at 30 days post-operative. Histometric, using image analysis software, and histologic analyses were performed. bone area (BA) and the remaining bone graft particles area (RPA) were calculated as a percentage of the total area of the original defect. Percentage data were transformed into arccosine for statistical analysis (analysis of variance, Tukey, p < 0.05). No defect completely regenerated with bone. Group AB/PRP (60.27% ± 8.04%) had a statistically greater BA than Groups C (19.29% ± 5.11%) and AB (49.93% ± 7.01%) (p < 0.001). No statistically significant differences were observed between Groups AB and AB/PRP with regard to RPA (p > 0.05). Within the limits of this study, it can be concluded that PRP improved the incorporation of AB grafts, increasing the amount of new bone formed. PRP has not influenced the resorption of non viable particles of the AB grafts.

## 114 Interest and satisfaction of professionals acting in family health program teams

SA, N.V. (Natália Vieira de Sá nativdesa@hotmail.com); CARVALHO, M.L.; SALIBA, N.A.; ARCIERI, R.M.

The aim of this study was to identify questions related to the interest, satisfaction and motivation of professionals who act in Family Health Program teams, pointing out the importance of these factors for the management's exercise in Brazilian public health. This qualitative and quantitative research, which was realizes through an interview, makes use of a half-structuralized script. The data show to one high degree of satisfaction in the work in categories of auxiliary level (nurse aid and communitarian agents of health, with 75% and 68.4% respectively), despite the low wages. Already, amongst the professionals of superior level, the dentist presented high degree of dissatisfaction (833%) and the medical classroom, in its great majority, showed (71.4%). It was concluded that most of the professionals that work in the Health Family Program have demonstrated interest and likeness with this program, in spite of the exposed dissatisfaction degree by some groups of professionals. It was also evident the importance of acknowledging subjective factors for the optimization of the dynamics of the work in the health field.

## 115 Benign cementoblastoma: case report

GALVÃO, N.S. (Neiandro dos Santos Galvão neiandrogalvao@gmail.com); RODRIGUES, M.T.V.; ALEIXO, R.Q.; CUNHA, F.J.P.; COSTA, G.V.C.; COSTA, M.R.S.N.

Benign cementoblastoma or cementoma is a benign odontogenic tumor. It is believed that this lesion presents the unique true cementum neoplasm. It is a rare lesion comprising 1 to 6,2% of odontogenic tumors. A healthy 16-year-old white female, without relevant medical or dental history, complained of pain in right posterior region of mandible. Clinical examination revealed an intraoral hard swelling by means of palpation in that region. Radiographic examination demonstrated a 10 mm well-defined radiopaque image attached to the root of tooth 46, surrounded by a radiolucent area. These clinical and radiographic findings led to presumptive diagnosis of benign cementoblastoma. The treatment consisted to surgical enucleation, tooth extraction and histopathological examination. The definitive diagnosis was benign cementoblastoma. Based on these findings, enucleation of the lesion followed by dental extraction and peripheral osteotomy consisted in treatment, beyond periodical clinical and radiographic follow-up. The findings related in this case corroborate with literature findings. This case reinforces the peculiarities of the cementoblastoma, which, due to its low incidence, is often not included in the differential diagnosis involving tooth root lesions.

## 116 Trilok® system, a new generation of fixation materials for mandible fractures

NOGUEIRA, L.M. (Lamis Meorin Nogueira lamismnogueira@yahoo.com.br); FLORIAN, F.; TRINDADE, P.A.K.; HOCHULI-VIERIA, H.

Motor vehicle accidents and assaults are the primary causes of mandible fractures. Fracture treatment can be classified by the method of reduction, as either open or closed. If open reduction is used, it can be further subclassified into nonrigid and rigid fixation (RIF). Management of mandibular fractures with transosseous wires has largely been replaced with the techniques of rigid fixation. Before rigid fixation became popularized, transosseous wires were used to maintain fracture fragment reduction in combination with MMF (maxillomandibular fixation). Rigid fixation techniques in the dentate patient begin with fixation of the occlusion. MODUS® TriLock® is a new generation multidirectional and angular stable fixation system for maxillofacial surgery. The purpose of this study is to demonstrate the advances of this new system that allows locking screws to be fixed in the plate within a freely selectable range (± 15°) in mandible fractures. This study used materials found in the literature. The spherical three-point wedge-locking creates a connection between the head of the screw and the plate hole which is stable in angle and axial plane. It can be concluded that this system provide a satisfactory stability of the fracture reduction.

## 117 Orthodontic and restorative dentistry interdisciplinary management for treatment of missing maxillary lateral incisors: case report

PINI, N.I.P.; UBALDINI, A.L.M.; MARCHI, L.M.; GIRARDI, A.R.; PASCOTTO, R.C.

Congenitally missing maxillary lateral incisors are commonly associated with an asymmetric smile and disharmony dental facial. The orthodontic space closure with posterior recountouring of the canines and premolars in lateral incisors and canines, respectively, is one of the alternatives of treatment for these cases. The purpose of the present assignment was to show the interdisciplinary approach between Orthodontics and Restorative Dentistry in the esthetic and functional rehabilitation in cases of maxillary bilateral lateral incisors agenesis. A female patient JH, 30 years old, looked for dental treatment, complaining of the absence of her maxillary lateral incisors. Based on the suitable indications, the treatment option was the movement of the canines and premolars to mesial and subsequent recountouring of these teeth with esthetic materials. Right before the bracket attachment, the cusp tips, and buccal and proximal faces of the canines were reduced. Flattening of the buccal face of the canines permitted attaching specific brackets to afford the movement of these teeth with adequate clinical crown torque. In this phase, besides the movement, the gingival zenith was aligned, with canine extrusions and premolar intrusions, and the torques and angulations of “new” lateral incisors and canines were adjusted. For a better distribution of space and adequacy of dental ratios in the restorative stage, a diastema between the central and lateral incisors was maintained for posterior closure with composite resin. After conclusion of the orthodontic treatment, an esthetic treatment of the anterior teeth was initiated, with bleaching for color adjustment and use of composite resins for cosmetic recountouring. The results of combination techniques were highly satisfactory concerning the esthetic principles and the patient's expectation. The integrated and multidisciplinary planning of the present case, involving Orthodontics and Restorative Dentistry, is of great value for esthetic and functional rehabilitation of the patients with lateral incisor agenesis.

## 118 Influence of the removal of dental structure in the biomechanical behavior of maxilary premolars

OLIVEIRA, L.K. (Larissa Kattiney de Oliveira lailaioliveira@hotmail.com); NORITOMI, P.Y.; KEMMOKU, D.T.; MARTINS, L.R.M.; SOARES, C.J.; SOARES, P.V.

It is known that the tooth structure loss influences on the stress concentration within the structure. However, little is known about which structure is important for reinforcement and dissipation of these stresses. This work evaluated the biomechanical behavior of maxillary human premolars by analysis of the distribution of stresses varying the type of cavity preparation. One maxillary premolar uniform coronal dimensions was selected with different types of root morphology Uni - uniradiculares. Seven types were generated for 3D finite element analysis: H-sound (control), O-occlusal, disto-occlusal-OD, MOD-mesio-occlusal-distal, MOD + A endodontic access, TE- endodontic treatment and CR - composite resin restoration. The biomechanical behavior of the teeth was analyzed in a qualitative and comparative by computer simulation to analyze the stress distribution (finite element method). It was observed that the removal of tooth structure favors higher accumulation of stresses within the structure, and that the pulpal wall and marginal ridges were essential for reinforcement of the preparation.

## 119 Gingival barrier in parendodontic surgery

MINOTTI, P.G. (Paloma Gagliardi Minotti paminotti@yahoo.com.br); BRAMANTE, C.M.; BRAMANTE, A.S.; GARCIA, R.B.; MORAES, I.G.; BERNARDINELI, N.

A surgical field free of blood is an indispensable condition for carrying out certain procedures during parendodontic surgery. Recently, it has sought means to make the isolation of the root apex to the achievement of the retrograde filling, which is not always possible according limited surgical access and the small remaining root. The use of gingival barrier, “Top-Dam” seems a viable option for this purpose. During the surgery, a curettage is performed and the hemostasis shall be obtained, the gingival barrier is then put in place covering the cavity walls, leaving the root apex uncovered. Then, the resin is cured for 30 seconds and, if necessary, additional layers of material may be added. Performed the surgery, retrograde filling, the barrier is removed in blocks with the use of curettes. This is facilitated by the contrasting color of the barrier, so avoiding the permanence of residue in the surgical cavity. There are no studies in the literature, so far, relating if the permanence of the material may affect healing process. The use of gingival barrier shows up as an alternative to surgical procedures that require an area free of blood and/or moisture.

## 120 Natural chromatism and texture in the treatment of anterior dental diastema using composite resins

RUSSO, P.D. (Patrícia Domene Russo patricia.russo@usp.br); NAHSAN, F.P.S.; SILVA, L.M.; MODENA, K.C.S.; FRANCISCONI, L.F.; MONDELLI, R.F.L.

Diastema is a dentofacial anomaly frequently found in Dentistry. Usually, it is located on the anterior segment between maxillary central incisors and is attributed to various etiologic factors, such as: abnormal growth and development, presence of labial frenum, intermaxillary suture failures, absent teeth, harmful addiction, muscular disorders, physical disabilities, iatrogenic origin, dentoalveolar discrepancies and pathological conditions, and others. Diastema restoration success is directly related to a precise diagnostic, treatment of the specific etiology and appropriate planning. There are various treatment options based on the size and etiology of diastemas. The most employed treatment is the direct adhesive restoration, due to its advantages. It is a practical, reversible, low-cost, less time-consuming clinical treatment and offers a variety of composite resin shades. The aim in this work is to report the clinical case of a 20-year-old patient, JPS, presenting a diastema between the maxillary central incisors remaining from an orthodontic treatment. A direct adhesive restoration was made with the nanofilled composite resin Four Seasons (Ivoclar-Vivadent), shade A1 enamel and A1 dentin. This case copied in details the anatomy, as well as chromatism and texture of natural teeth, providing results that guarantee satisfaction, harmony and perfection for the patient.

## 121 Relationship between periodontics and restorative procedures: surgical treatment of the restorative alveolar interface (R.A.I.) - case series

OLIVEIRA, P.G.F.P. (Paula Gabriela Faciola Pessôa de Oliveira paulagpessoa@yahoo.com.br); BARBOSA, K.T.; CUNHA, M.J.S.; FIAMENGUI FILHO, J.F.; NATALICIO, G.L.; ALMEIDA, A.L.P.F.

Iatrogenic restorative procedures may lead to gingival inflammation up to periodontal pockets formation with bone resorption and tooth mobility. Overcontoured restorations, inadequate proximal contacts, biological distances invasion and poor marginal adaptation may impair periodontal health, leading to a chronic inflammatory process and further restoration failure. The correct understanding of the periodontal tissues, dimensions and biology is extremely important for a successful restorative treatment. The objectives of this work were to show through case reports the rationale and techniques for the preparation of the restorative alveolar interface (R.A.I.), reestablishing periodontal health.

## 122 “Saddle-type” defects created in the inferior border of the mandible of dogs. A new surgical approach

JORGE, P.K. (Paula Karine Jorge paulak.odonto@gmail.com); MESSORA, M.R.; NAGATA, M.J.H.; FURLANETO, F.A.C.; GARCIA, V.G.; BOSCO, A.F.

Pre-clinical studies have been using “saddle-type” experimental bone defects surgically created on the alveolar ridge of animals to evaluate the effect of biomaterials and bone grafts on the corrections of alveolar ridge deformities. The occurrence of postoperative complications as bacterial infection, mucosal inflammation and early exposure of the barriers used is the major disadvantage of this experimental model. The purpose of this study was to evaluate the efficiency of a modified technique to create “saddle-type” defects in mandible of dogs. Bilateral bone defects, measuring 1.5 cm in width vs. 1 cm in height, were created in the inferior border of the mandible of 6 adult male dogs using an extraoral approach. The defects were filled with blood clot only. All animals were euthanized at 12 weeks postoperative. Histologic and histometric analyses were performed. No defect regenerated completely with bone tissue. Postoperative complications were not observed. The mean percentage of newly formed bone and the standard-deviation were 70.55 ± 8.01%. Within the limits of this study, it can be concluded that the experimental model used is efficient and predictable to evaluate the effect of biomaterials and bone grafts on the reconstruction of alveolar ridge deformities.

## 123 Revascularization study of autogenous bone block in ovariectomized aged and young rat model

FALEIROS, P.L. (Paula Lazilha Faleiros paulal.faleiros@hotmail.com); MURAKAWA, A.C.; FERNANDES, L.A.; MARTINS, T.M.; OKAMOTO, R.; BOSCO, A.F.

The aim of this study was to evaluate the influence of the ovariectomy (estrogen depletion), on healing process of autogenous bone block grafts in young and aged rats, through the immunolocalization of the PECAM-1. 96 female Wistar rats were used: 48 3-month-old and 48 12-month-old rats divided in two subgroups: Ovx, subjected the ovariectomy surgery and Sham, subjected to the same surgical procedure without the removal of the ovaries. After 30 days of Ovx or “sham” operation, all animals received autogenous bone block graft in the jaw, harvested from the calvaria. The animals were euthanized in 7, 14 and 28 days postoperatively. The specimens were subjected to histometric and immunohistochemistry analysis. This was accomplished in a semi-quantitative way, to analyze the immunolocalizations against PECAM-1, evaluating the interference of the estrogen in this process. The PECAM-1 immunolocalizations were more intense in the Sham subgroups, being more evident at 14 days when compared to Ovx subgroups, which presented fewer demarcations independently of the age. Comparing young and aged female rats, it was noticed more intense immunolocalizations in the first group. Within the limits of this study, it may be concluded that estrogen depletion affects the initial process of angiogenesis negatively.

## 124 Subjective analysis of esthetics of smile and the occurrence of golden proportion in natural dentition

NISHITA, P.M. (Paula Mayumi Nishita paulanishita@hotmail.com); BISPO, C.G.C.; JUNIOR, J.C.B.S.; MAGGIONI, B.T.; OZELAME, M.L.; SOBRINHO, E.M.

The aim of this study was determine the frequency that the golden proportion is exhibited in smiles considered as beautiful according to subjective aspects of selection. The smiles of sixty volunteers dental students were photographed. The smiles were randomly numbered and examined individually by three blinded examiners, who classified the smiles as beautiful and non-beautiful using subjective criteria. The beautiful and non-beautiful groups were composed of smiles rated this way by the three examiners in a consensual manner and the others were excluded. Smiles of both groups were printed and analyzed, verifying the golden proportion in the anterior teeth using a caliper. Among eleven smiles that composed the beautiful group, only three (27%) exhibited golden proportion, two female and one male smile. None of the seventeen smiles that composed the non-beautiful group exhibited golden proportion. The divine proportion, probably, is considered more frequently in smiles considered beautiful by subjective criteria and is more common in female smiles.

## 125 Correction of the anterior cross-bite with the progenic appliance: clinical case report

PRIMO, P.P. (Paula Patricia Primo paulapprimo@hotmail.com); SPADA, L.G.; SOUZA, R.S.

Due to the high frequency of anterior crossbite in patients that are in the deciduous dentition and beginning of the mixed dentition period, the performance of a differential diagnosis becomes indispensable to establish an effective orthodontic planning and consequently, a precise treatment, since malocclusion can be shown with dental, skeletal or functional characteristics. Therefore, the aim of this study was to demonstrate the treatment for anterior cross-bite malocclusion through the use of removable progenic appliances. The results demonstrated the normal occlusion acquired and also guided occlusal stability. In this meaning, this study pointed to the importance of dentists being alert to detect mild normality deviations, applying simple actions in order to prevent their negative influence over the following stages of development and, consequently, the future occlusion.

## 126 Treatment of caseous by tonsillectomy

SILVA, P.V.R. (Paula Verona Ragusa da Silva paulinharoxy@hotmail.com); HAYACIBARA, R.M.; ROCHA, A.L.; CANDIDO, A.G.; CANDIDO, G.C.; MAIO, R.C.

The halitosis is an oral alteration that causes a significant social impact, bringing on restriction of the patient and minimizing their quality of life. One of the etiological factors for the bad breath is the presence of tonsillar caseous. This is formed in invaginations of the palatine tonsils, known as crypts. It is composed of epithelial sloughed cells the oral mucosa, salivary proteins and protein food leftovers, which serve as a substrate to proteolytic anaerobic bacteria present. At the end of bacterial metabolism, are produced volatile sulfur compounds, responsible for halitosis. Clinically presents itself as a yellowish mass of extremely unpleasant odor. The proposed treatments are most often outpatients, through the use of anti-inflammatory in cases of chronic tonsillitis caseous, gargle with saline and antiseptic solutions, and laser or surgical criptolysis. The objective of this work is to show the tonsillectomy as a therapeutic option of surgical caseous tonsillar, reporting the case of a 20-year-old male patient with frequent occurrence of caseous who was subjected to this form of treatment. We concluded that although tonsillectomy is considered an invasive method, it can a viable alternative treatment because the conservative therapies are often mechanisms palliative. The laser criptolysis, despite being a safe and efficient, has a painful postoperative because several surgical interventions may be required. Currently there is not an economic and non-invasive treatment that produces satisfactory results as seen after the removal of palatine tonsils.

## 127 Treatment of congenitally missing lateral incisors

PEREIRA, P.F.J. (Peragibe Felix Pereira Junior peragibefelix@hotmail.com); PINELLI, F.V.; CURY, J.V.; CHIQUETO, K.

The congenitally missing maxillary lateral incisor shows a strong heritable aspect, with a prevalence of 2%, and more common among women. The orthodontic treatment of malocclusion with congenitally missing these teeth can be performed by closing or reopening the spaces. The aim of this work is to present the clinical proceeding in a case of congenitally missing lateral incisors of a twelve-year-old girl. The treatment involved space closure and extraction of the mandibular first premolars. There was no need for canine rebuilding, because the satisfaction of the patient was great at the end of treatment. The treatment length was one year and eight months, obtaining a straight profile, Class I molar relationship and satisfactory static and functional occlusion. These results ensure good treatment stability. The correct diagnosis and adequate treatment plan are essential for the orthodontic treatment success of congenitally missing teeth, restoring then the occlusion, function and esthetics.

## 128 Ectopic tooth in the mandibular incisure: case report

PEREIRA, A.L.B. (Anne Lis Barbosa Pereira annelis_bp@hotmail.com); ROCHA, F.S.; BATISTA, J.B.; SILVA, C.M.; GONDO, R.M.; MELO, P.E.C.

Ectopic tooth occurs when a tooth develops in the wrong position. This condition is a result of an abnormal tissue interaction in the tooth germ development stage. Ectopic teeth in distant places of the dental arc are uncommon; they have been reported in nasal septum, mandibular condyle, coronoid process, palate, maxillary sinus and others. Ectopic third molar cases are poorly reported, with etiology and treatment still questionable. We reported a case of an 18-year-old man with an inferior third molar localized in the mandibular incisure found in a routine panoramic radiograph. As the treatment approach, a surgical removal with intraoral approach was performed. The clinical, radiographic and histopathologic features of this entity will be presented and the important aspects of treatment and etiology of this lesion will be discussed.

## 129 The photodynamic therapy in dentistry

PERON, R.A.F. (Rodrigo Aparecido Flausino Peron odontoperon@yahoo.com.br); TERADA, R.S.S.; HAYACIBARA, M.F.; GRACIANO, A.X.; NAGATA, J.Y.

Photodynamic therapy (PDT) is a therapeutic modality that combines the use of a photosensitive compound and light to cause cell death. More than 4000 years ago the Egyptians began the therapy, through the ingestion of plants and exposure to sunlight to treat diseases such as vitiligo. The PDT as a technique and therapy, however, began to be used with systematic science only recently. It assumes that the interaction of light of wavelength appropriate compound with a non-toxic and result in oxygen reactive species such as singlet oxygen, capable of inducing the non viability cells. The purpose of this work is to present the current stage of photodynamic therapy in many fields of Dentistry, and their applications in cariology. For this, a literature review was done on national and international databases in the last 10 years. It was found that PDT is studied particularly in the fields like stomatology, endodontics, periodontics and dentistry. Especially for the last two, which depend on a biofilm, and through the elimination of that by PDT will result in the removal of the causative agents of oral diseases more prevalent and incident. There are different photosensitive being investigated, with different combinations of light sources. The photogem, the methylene blue, ortho-toluidine blue, Rose Bengal, erythrosine, chloride, phenothiazine, hematoporphyrin as photosensitive compounds in different concentrations; and laser, the LED, tungsten filament, and even the white light as light sources, at different times of application (from 5 seconds to 30 minutes) and power (11 to 450 mW) present themselves as possibilities for treatment. The PDT in Dentistry is a promising alternative of treatment and research in this field should expand in the short term.

## 130 Self-etching adhesive systems and conventional adhesive systems: a micromechanical analysis by 3-D FEM

PITA, D.S. (Diego Sucena Pita rodolfoanchieta2@hotmail.com); MARTIN JUNIOR, M.; ANCHIETA, R.B.; ARCHANGELO, C.M.; ROCHA, E.P.; FREITAS JUNIOR, A.C.

The aim of this study was to evaluate the stress distribution in the hybrid layer varying the adhesive system (total each and self-etching systems) using three-dimensional finite element analysis (FE). 4 FE models (M) were developed in Solidworks 2007: Mc - representation of a specimen of dentin (41x41x82 ìm) restored with composite resin (RC), showing the adhesive layer, hybrid layer (HL), TAGs, peri-tubular dentin, intertubular dentin in order to simulate simulating the HL according to the total-etch adhesive system; Mr - similar to Mc, with lateral branches of the adhesive; Ma – similar to Mc, without TAGs and showing the \”smear plug\” in order to simulate the environment for the self-etching adhesive system; Mat – similar to Ma, with TAGs. The models were considered isotropic, homogeneous and linearly elastic and numerical analysis was performed in ANSYS Workbench 10.0 to obtain the maximum principal stress (ómax) after application of a tension force of 0.03N perpendicular to the surface of the RC. The botton of all models was fixed in x, y and z axis. The highest ómax in HL was observed in the total etching system. The lateral branches increased the ómax in HL. The TAGs had a little influence on the stress distribution in self-etching system. The HL for the total-etch system showed ómax higher in comparison with the self-etch system. The presence of TAGs increased the ómax in the HL for the total-etch system.

## 131 Nasal morphology evaluation after rapid maxillary expansion in children

AYUB, P.V. (Priscila Vaz Ayub pri_ayub@yahoo.com.br); SILVA FILHO, O.G.; LARA, T.S.; OHASHI, A.S.C.; PICCOLI, V.D.

During the procedure of rapid maxillary expansion, the two maxillary halves are divided in a pendular way both horizontally and frontally with consequent skeletal alterations such as increase in nasal width, maxillary width and zygomatic width associated to anatomic alterations in nasal cavity. The possibility of changes in nasal morphology with rapid maxillary expansion performed on children could contraindicate the procedure in the deciduous and mixed dentition. The aim of this study was to determine, using facial analysis, the effects of rapid maxillary expansion in nasal morphology in children in the deciduous and mixed dentition. We have selected facial photographs in frontal and lateral norma of 60 patients during the pretreatment, immediate post-expansion and 1-year-post-expansion phases using a Haas expander. These facial photographs were evaluated twice — with a two-week interval between them — by three orthodontist examiners, separately. The examiners were asked to evaluate the nasal morphology and did not know about the content of the research. The agreement level was analyzed by kappa statistic test. There were no alterations in nasal morphology in any patient as far as nasal dorsum, alar base, nasal base and nasal width of midface were concerned. There are changes in nasolabial angle in 1.64% of the patients when the pretreatment and immediate post-expansion photographs are compared, in 4.92% of the patients when the immediate post-expansion and 1-year-post-expansion photographs are compared, and in 6.56% of patients when the pretreatment and 1-year-post-expansion photographs are compared. The facial analysis allow us to conclude that rapid maxillary expansion performed on children in the deciduous and mixed dentition did not show impact regarding their nasal morphology.

## 132 Use of subepithelial connective tissue graft in the correction of defects in maxillary ridges

ROMUALDO, P.C. (Priscilla Coutinho Romualdo priromualdo@hotmail.com); FERNANDES, P.G.; CRIVELENTI, M.D.; TABA JUNIOR, M.; REINO, D.M.

Alveolar ridges defects may occur after tooth extractions, congenitally, by trauma or iatrogeny. Their presence implies esthetic injury to the patient, especially when present in the anterior region of the oral cavity. This condition makes difficult to obtain esthetic in rehabilitation treatment and causes discomfort to patients. To resolve this problem, several techniques have been proposed, such as the use of bone grafts or connective tissue graft. The techniques of periodontal plastic surgery have high degree of predictability and esthetics, are less invasive, with good postoperative, allowing the start of prosthetic treatment more quickly when compared to the use of bone grafts. The use of subepithelial connective tissue graft has as one of their indications, the increase of the amount of tissue in deficient maxillary ridges. Therefore, the aim of this case report is to demonstrate the use of the subepithelial connective tissue graft for the treatment of deficient maxillary ridge, as a clinical option for improving the achievement of esthetics, when associated with the use of prostheses. A female patient with missing teeth #22 and #25, with deficiency in maxillary ridge, caused by tooth extractions, with necessity of rehabilitation treatment. The patient received supra-gingival scaling, plaque control and oral hygiene instructions. When the plaque index was less than 20%, surgical treatment was performed. The palate was used as a donor area for the connective tissue that was divided and inserted into the area corresponding to the teeth #22 and #25, after elevation of a partial thickness envelope flap. After evaluation with weekly, bi-weekly, monthly and quarterly, occurred a formation of large volume in the treated areas, with the presence of keratinized attached gingiva, allowing the achievement of esthetics in the provisional prostheses. Thus, the procedure was effective for the treatment of deficient maxillary ridge as a way of improving the esthetic and functional result obtained with prostheses.

## 133 Esthetic and functional rehabilitation of dental corrosion in integrated clinic

PUPIM, D. (Denise Pupim denipupim@hotmail.com); LUCAS, N.M.; PASCOTTO, R.C.; RIGOLON, C.J.; SILVA, R.S.; MELO, M.P.

The loss of tooth structure begins at the moment of teeth eruption. This loss of structure is currently called dental corrosion. The process may occur as a result of chemical agents (acids in the diet), physical agents (occlusion) and mechanical agents (toothbrush). Erosion is a non bacterial pathological loss of tooth structure, induced by chemical processes. The loss during the physiological masticatory movements is called dental attrition, which is exacerbated by parafunctional habits such as bruxism, accelerating the physiological process of tooth wear. The aim of this paper is to report the case of a patient who had severe anterosuperior tooth wear, with a multifactorial etiology. The acid diet and parafunctional habit caused the loss of tooth structure in the buccal and incisal surfaces, compromising the esthetics of the smile. After the bleaching procedure, performed using 35% hydrogen peroxide activated by a LED/laser light source, a cast model was obtained in which the esthetic and functional reconstruction was planned by waxing the worn surfaces. From the waxed model, we obtained a silicon guide that helped building the direct composite resin restorations. Finally, an occlusal splint was fabricated to protect the restorations.

## 134 Association of enamel microabrasion and dentinal bleaching in recovering an adolescent patient's smile: a case report

SOUZA, R.G. (Rafael Guimarães de Souza rafaeldesouza87@gmail.com); GUEDES, A.P.A.; OLIVEIRA, F.G.; MACHADO, L.S.; SUNDFELD NETO, D.; SUNDFELD, R.H.

This article presents the enamel microabrasion protocol for removing intrinsic white stains of hard texture from the enamel surface, using a 37% phosphoric acid/pumice mixture associated with a carbamide peroxide-based bleaching agent in custom-made mouth trays. We observed that these clinical procedures were safe and effective, and solved our patient's esthetic problem.

## 135 Impact of regular and light drinks on enamel erosion

REBELATO, R. (Rafael Rebelato rrebelato_1986@hotmail.com); MAGALHÃES, A.C.; HONÓRIO, H.M.; MACHADO, M.A.A.M.; BUZALAF, M.A.R.; RIOS, D.

FAPESP (Proc. 2006/07260-4)

A previous *in situ* study showed differences on the erosive potential of a regular and a light cola drink on enamel. However, no information is available about the erosive potential of regular and light drinks *in vitro*. Thus, this *in vitro* study aimed to compare the erosive potential of different regular and light beverages on enamel. Bovine enamel samples were divided into 7 groups (each n=10) and immersed in the regular or light/sugar zero version of the following drinks: Coca-cola, Guaraná and Sprite. Half of the surface of the specimens was coated with nail varnish for reference. The samples were subjected 4 times daily to erosive challenges (30 mL beverage/sample) for 2 minutes at room temperature. Between the erosive challenges, the samples were immersed in artificial saliva (30 mL/sample) for 2 h. In each day, after the pH-cycles the samples were stored in artificial saliva. At the 5th day, the enamel surface alterations were measured by profilometry (μm). The data were analyzed by ANOVA followed by Tukey's test (for cola drinks) and unpaired t test (for other beverages) (p<0.05). Light coke (0.52±0.19) provoked a similar enamel wear compared to zero coke (0.54±0.17), and both caused less enamel loss compared to the regular version (2.15±0.65). On the other hand, regular sprite (1.17±0.36) provoked lower enamel wear compared to zero version (2.25±0.75). Regular (1.06±0.36) and light Guaraná (1.02±0.26) provoked similar wear. This *in vitro* study showed that the light and zero cola drinks showed less erosive potential, however this was not observed for the zero versions of guaraná and sprite. Financial support: FAPESP (Proc. 2006/07260-4).

## 136 Is iron gel able to reduce erosion in bovine enamel?

BONATO, R.C.S. (Rafaela Carolina Soares Bonato rafaelacarolin@hotmail.com); BUENO, M.G.; MARSICANO, J.A.; SALES-PERES, A.C.; MOURA, P.G.; SALES-PERES, S.H.C.

FAPESP (proc. 2008/00240-3) e CNPQ (proc.109.160/2007-0)

The aim of this study was to evaluate, *in vitro*, the effect of an experimental gel containing iron and/or fluoride on the erosion of bovine enamel. For standardization of the blocks (n=80), a previous selection of specimens (4X4 mm) for the initial microhardness was made. The blocks were randomly allocated to four groups of 20 samples each, due the treatment: G1 (control, placebo gel); G2 (fluoride gel, 1.23%NaF); G3 (iron gel, 10 mmol/L FeSO4) and G4 (fluoride+iron gel, 1.23% and 10 mmol/L FeSO4). The gels were applied and removed after 5 min. Then, the blocks were subjected to six cycles, alternating re- and demineralization (only one day). Demineralisation was performed with the beverage Coke® (10 min, 30 mL) and remineralisation with artificial saliva (1 h). The effect of erosion was measurement by wear analysis (profilometry). Data were analyzed by ANOVA and Tukey test to individual comparison (p<0.05). The gel contained only iron showed that the better effect (G3: 0.47±0.11 μm), however there was no differences when compared to others gels (G2: 0.55±0.12 μm; G4: 0.53±0.13 μm). There was significant statistically reduction in enamel wear when it was used experimental gels and compared to control (p<0.001). It concluded that the gels containing iron with or without fluoride can work lin a preventive form against erosion, reducing the loss of tooth surface, *in vitro*.

## 137 Dental care for children with diabetes

SILVA, R.B.P. (Raquel B. Parra da Silva raqueparra@bol.com.br); DIOGO, F.S.F.; AGUIAR, S.M.H.C.A.

Diabetes mellitus (DM) is a metabolic disorder of multiple etiology characterized by chronic hyperglycemia with disturbances in the metabolism of carbohydrates, fats and proteins, resulting from defects of insulin secretion, the same action or both. The three types of diabetes are type 1, type 2 and gestational. The first type 1 diabetes results from destruction of beta cells of the pancreas and has a tendency to ketoacidosis. Includes cases arising from auto-immune diseases and those in which the cause of the destruction of beta cells is not known. Type 2 diabetes results, in general, of varying degrees of insulin resistance and relative deficiency of insulin secretion. Most patients have excess weight, and ketoacidosis occurs only in special situations, such as during serious infections. Oral complications may include: gingivitis, periodontal disease, the salivary gland dysfunction, xerostomia, oral susceptibility to infections and changes in taste. This presentation aims to emphasize the routine and emergency dental procedures for patients with DM, specially the child care. Well-controlled and uncomplicated diabetics can be treated in a similar way to non-diabetics, for most routine procedures. The dentist must be in communication with the physician to promote and maintain the welfare and quality of life of the diabetic patient.

## 138 Evaluation of pH and calcium ion release of five retrofilling materials

MIDENA, R.Z (Raquel Zanin Midena raquelmidena@yahoo.com.br); VIVAN, R.R.; DUARTE, M.A.H.; MORAES, I.G.; GARCIA, R.B.

The biological and antimicrobial action of the retrofilling materials is directly attributed to alkaline pH and calcium ion release. The aim of this study were to analyze the pH and the ion calcium release of five retrofilling materials (MTA white Angelus, MTA Bio, photopolymerized MTA, Sealepox RP and the clinker of Portland cement associated with bismuth oxide and calcium sulphate). Polyethylene pipes were used with 1,0 mm of internal diameter and 10.0 mm of length, with only one of the extremities open, they were filled with cements and immediately immersed in test tubes contend 10 mL of deionized water, where they remained during the experimental period. Assessments were taken at 3, 24, 72 and 168 hours, always renewing the deionized water to the end of each period. The reading of pH was done using the pHmeter and the calcium ion release by means of one spectorphotmeter atomic absortion. The values were statistically compared using the Kruskal-Wallis and Miller tests. In relation to pH, the results demonstrated that in the experimental periods of 3 and 24 hours, the highest values were reached by associated clinker to bismuth oxide and calcium sulphate. At the time interval of 72 and 168 hours, the highest pH values were reached by the MTA Bio. In relation the calcium ion release, at the interval time of 3 hours associated with clinker to bismuth oxide and calcium sulphate presented the highest value. At 24hours, MTA Bio and the Clinquer they were superior. In the other periods, MTA Bio presented the highest values. It was concluded that all cements presented alkaline pH and calcium release ion in all periods, with trend of reduction in the final periods.

## 139 Brown tumor of hyperparathyroidism: an unusual presentation

ZANATTA, R.F. (Rayssa Ferreira Zanatta zanatta.rayssa@gmail.com); DURIGHETTO JÚNIOR, A.F.; DE PAULO, L.F.B.; ROSA, R.R.; SANTOS, V.P.F.

The brown tumor of hyperparathyroidism is a bone alteration which arises as reflex of parathyroid hyperactivation. The parathyroid hormone (PTH) causes mobilization of calcium from bone and when a large quantity was captured can cause a generalized bone dystrophy with increased possibility of fractures at the minimum effort. Moreover, it could be responsible for the formation of uni or multiloculated areas completed by tissue richly vascularized and with several multinucleated giant cells characterizing the “brown tumor”. The clinical aspects of interest for dental clinics are the disappearance of the lamina dura around the teeth roots (bone dystrophy) and extensive osteolytic lesions in the jaws. The mandible is the predominantly affected site in the maxillofacial area. Maxillary involvement is rare. We present an 84-year-old female patient complaining of two painful bilateral nodular sessile elastic lesions in the alveolar ridge in maxilla. The patient's medical history was unremarkable. The initial clinical and radiological evaluation indicated a peripheral giant cell lesion or brown tumor of hyperparathyroidism. An incisional biopsy was performed and a cell population consisting of rounded or spindle-like mononucleate elements, mixed with a certain number of multinucleate giant cells, among which recent hemorrhagic infiltrates and hemosiderin deposits were evident. The biopsy analysis and the total serum PTH levels (153pg/mL) provided the diagnosis of hyperparathyroidism presenting as a brown tumor. An ultrasound of the region was unremarkable as well as the other biochemical studies (calcium and alkaline phosphatase) and, based on these findings, a diagnosis of secondary hyperparathyroidism was raised. The aging of the skin and no exposure to sunlight may decrease the synthesis of vitamin D and calcium absorption leading for a hyperparathyroidism. This case thus highlights the importance of another facet to the myriad of presentations associated with hyperparathyroidism.

## 140 Use of composite restorations associated with prosthesis on functional rehabilitation

REIS, G.R. (Giselle Rodrigues dos Reis paulovsoares@foufu.ufu.br); REIS, B.R.; PEREIRA, F.A.; SIMAMOTO, V.R.N.; SOARES, C.J.; SOARES, P.V.

The concern for esthetics is increasing in society, it gets worse as they also have an associated functional loss, so the esthetics and comfort of the stomatognathic system muscles become dominant factors in rehabilitation. With the aging of the population, noncarious lesions, such as abrasion and erosion, have been expressed most often in the office. It is in this context that the patient J.C.A., 70 years searched the Clinic of Specialization on Restorative Dentistry of the Faculty of Dentistry of the Federal University of Uberlandia (FOUFU) complaining of: muscle pain, difficulty in chewing and change in shape of teeth. During clinical examination, it was observed a reduction in the vertical dimension of occlusion due to loss of posterior teeth and accentuated tooth wear. To restore the function, a record was performed with facial arc for observation of relationship and construction of removable provisional prostheses allowing occlusal stability in a later moment. In this way, sufficient space was gained for further placement of composite resin restorations of improving the esthetics of the patient. Composite resin was the material of choice for restoring the shape of the anterior and posterior teeth because it provided a fast treatment with good esthetics and low cost. The interdisciplinary restorative technique employed reestablishing function and comfort of the stomatognathic system, resulting in a harmonious smile with better quality of life for the patient.

## 141 Esthetic and periodontal rehabilitation of a patient diagnosed with amelogenesis imperfecta

RODRIGUES, R.B. (Renata Borges Rodrigues renataborgesrodrigues@hotmail.com); ROSCOE, M.G.; SILVA, G.R.; SOARES, P.B.F.; MAGALHÃES, D.; SOARES, C.J.

Amelogenesis imperfecta is an alteration that disturbs the developing enamel structure, causing esthetic problems and dentin sensitivity, which can affect the patients' self-esteem. The success of the rehabilitation must be based on the answers of the complex dentin-pulp and on the health of periodontais tissues, aiming at the functional and esthetic rehabilitation and the harmony of the stomatognathic system. The integration of periodontal behaviors associates to the restorative procedures is of great importance from the planning phase up to case follow up. The propose of this clinical report aims at the integral and integrated treatment of 12-year–old patient with amelogenesis imperfecta with extreme sensitivity due to exposure of dentin and gingival excess covering part of the clinical crown of teeth, impeding the immediate restorative procedure. Periodontal crown lengthening surgery was done in all teeth. After the healing period, the rehabilitation of all teeth started by means of direct reconstruction with composite resin, since the patient was very young and still presented some erupting teeth. This type of treatment is as option with low cost and good functional, physicial and esthetic outcomes.

## 142 Esthetic challenges in pigmented roots by oxidation of copper-aluminum cast posts

HAAB, R.I. (Renata Introvini Haab renata_haab@hotmail.com); DA LUZ, N.; BRANCO, C.A.; BUSO, A.M.; FONSECA, R.B.

The use of copper-aluminum cast posts has the great disadvantage of the natural oxidation and consequent dental root pigmentation. Tooth bleaching and metal-free ceramic crowns may be an alternative to reduce these installed effects when crown replacement is needed. The aim of this work is to present a clinical case where different treatments were accomplished to minimize the esthetic discomfort of a patient with highly pigmented roots by oxidation of copper-aluminum cast posts. Female patient, 45 years old, came to UEL's Dental Service complaining about darkening of teeth 11 and 12 and the surrounding gingiva. There was a misalignment in the line of marginal free gingiva and a moderate thickness of attached gingiva, and the teeth had metal-ceramic crowns retained by copper aluminum cast posts. The correction of gingival position was performed by periodontal surgery; the crowns were removed and the preparations subjected to bleaching with 35% hydrogen peroxide (2 sessions). The cast posts were not removed due to root fracture risks. After that, metal-free ceramic crowns (Empress Esthetic) were cemented with Scotchbond and dual resin cement Rely-X. The treatment resulted in esthetic improvement of the region of darkened appearance and gingival contour, although the prognosis cannot be predicted due to gingival characteristics and maintenance of cast posts. In conclusion, the use of copper-aluminum cast posts should be avoided, but when their use or maintenance is necessary, metal-free ceramic crowns can be an alternative to avoid the unaesthetic staining generated by metal oxidation.

## 143 Cementoblastoma: case report and review of diagnostic features

MIRANDA, R.B.M. (Renato Barjona M de Miranda renatobarjona@yahoo.com.br); SANTOS, V.P.F.; BATISTA, J.D.; RAMOS, L.M.A.; DURIGHETTO, A.F.

Cementoblastoma is a benign lesion of odontogenic ectomesenchymal origin characterized by neoplastic cementum-like tissue around the root of a tooth. It is usually a solitary slow-growing lesion attached to the root of a vital erupted permanent tooth, most often the mandibular first molar. Radiographically it is a mixed to radiopaque mass fused with a root and limited peripherally by a radiolucent halo. We report the case of a 19-year-old man presenting with an asymptomatic swelling in the left side of the mandible with 8 months of growing. Intraoral examination showed enlargement of the buccal portion of the first and second molars. It was hard in palpation and covered by normal mucosa. Periapical radiograph showed a well-circumscribed mass of mixed aspect, surrounded by a radiolucent halo and attached to the first molar mesial root. Expansion of the cortical was seen in an occlusal radiography. Radiographic aspect was very suggestive of cementoblastoma, and therefore an excisional biopsy was done. At surgery, the mass was well circumscribed, easily detached from surrounding tissue and tightly attached to the molar root. A thin and easily detachable soft tissue capsule surrounded the hard tissue. Histopathologic features were also compatible with cementoblastoma. Follow-up of six months was uneventful. The present case showed typical radiographic features for a cementoblastoma, except for the only discrete radiopacity in the cortical expansion evidenced in occlusal radiography. Surgical aspects also aided in the diagnosis, since fusion with root, circumscription, easily detachment, presence of thin soft tissue capsule are usual features for cementoblastoma. Simple exeresis is usually curative, and recurrences are exceedingly rare.

## 144 Influence of low level laser therapy on the repair of autogenous bone block graft in the jaw: histologic and histometric study in rats

MORAES, R.O. (Ricardo Oliveira de Moraes rickfoa@hotmail.com); BOSCO, A.F.; MURAKAWA, A.C.; FERNANDES, L.A.; GARCIA, V.G.; MARTINS, T.M.

The purpose of this study was to evaluate through histological and histometrical analysis the influence of low level laser therapy (LLLT) in the repair of autogenous bone block grafts in the jaw of rats. They were used 48 mice that divided in two groups: Group C (Control Group n=24) and Group GL (Laser Group n=24). All animals received autogenous bone graft installed in the jaw, in the close area to the angle, tends as area donor the parietal bone of the calvaria. The animals of GL received treatment with the laser of low intensity in the surgical bed, before the fixation of the graft; while GC didn't receive any treatment. The animals were subjected to euthanasia in the periods of 7, 14 and 28 days after surgical procedure. In 7 days was observed presence of little organized connective tissue in both groups with larger angiogenesis in GL. To the 14 days it was found connective tissue developed in GC and GL, with larger bone formation and angiogenesis activity in GL. To the 28 days the osteogenesis was intense in both groups. In GL the recipient bone-graft interface was partially filled by newly formed matrix bone, establishing a union of the graft to the receiving bed. In GC, the interface was partially filled by newly formed matrix bone, with areas of connective tissue between the graft and the recipient bed. Histometrically the newly formed bone was significantly larger in GL (60.11% ± 8.57%) that in GC (44.03% ± 10.21%) at 14 days. At 28 days, there was was no statistically significant difference between groups GL (74.46% ± 9.11%) and GC (67.81% ± 8.02%). Within the limits of the present study, LLLT promoted a significant photobioestimulator effect on the process of repair of bone block grafts installed in the jaw.

## 145 Pemphigus vulgaris: case report

AZEVEDO, R.M.C. (Roberta Maués de Carvalho Azevedo robertamaues@yahoo.com.br); ALMEIDA, O.P.; ANDRADE, G.C.; VARGAS, P.A.; NAVARRO, C.M.

Pemphigus vulgaris (PV) is a rare immunomediated blistering mucocutaneous disease with early oral lesions potentially spreading to other mucous membranes and skin. Mucosal lesions may be the sole sign for an average of 5 months before skin injuries develop, or they may be the sole manifestation of the disease. Mean age of PV onset is 50 – 60 years. The oral lesions are characterized by erosions secondary to the rupture of flaccid blisters. The soft palate, buccal mucosa and lips are the most affected anatomical region of the mouth. We report a case of a 58-year-old man with a chief complaint of painful oral ulceration during previous 3 months. The patient medical history revealed malaria 4 years ago. The oral examination showed multiple vesicular and erosive lesions on lips, hard and soft palate, buccal mucosa, alveolar ridge and tongue. The Nikolski's sign was positive. Two incisional biopsies on upper lip (erosive and vesicular areas) were taken. The microscopical examination showed acantholisis, intraepithelial cleft with Tzank cells, and basal cell layer attached to subjacent connective tissue. This histopathological pattern is typical for PV. The patient was referred to the Ophthalmologist and presently is under control with Predinisone (Meticorten ® −15 mg/day). Since the oral lesions precede systemic involvement, we emphasize the importance of the dentist in the early diagnosis, improvement of prognosis and treatment of this disease.

## 146 Urbach-Wiethe syndrome

ROSA, R.R.; ROCHA, M.A.; DURIGHETTO, I.L.; SARGENTI, S.; BARBOSA DE PAULO, L.F.; ZANATTA, R.F.

Lipoid proteinosis (LP), also known Urbach-Wiethe syndrome, is an uncommon, recessively inherited genodermatosis characterized by deposition of amorphous hyaline material in different parts of the body, especially the skin, mucous membranes of the upper aerodigestive tract, and internal organs. Clinical manifestations of LP usually begin as a hoarseness and failure cry soon after birth or in the first year of life. However, other conditions may occasionally appear for few years later. Oral cavity is most extensively affected area by the disease and the main oral abnormalities include diffusive infiltration of white pea-size plaques and hardness of the tongue as well as inability to protrude it. In this report we describe the main aspects of a classical case of LP affecting a woman who presented with an unusual history of painful recurrent ulcerative lesions in her tongue since childhood, probable caused by a persistent and severe xerostomy, which had developed as a consequence of this genetic disorder.

## 147 Complication after installation of piercing in lower lip area: case report

RODRIGUES, V.L.O. (Vitor Hugo Leite de Oliveira Rodrigues granja31_chorito@yahoo.com); DUARTE, B.G.; DIAS-RIBEIRO, E.; ASTOLPHI, F.A.; SANT'ANA, E.; CAPELOZZA, A.L.A.

Body piercing is currently common in young people. Among the places chosen for the placement are: lip, tongue, uvula and frenum. The reasons that lead to this practice include expression of identity, esthetics, fashion and rebellion. The placement of the piercing is done through a perforation of the mucosa and the tissue at the desired location, which can cause complications in piercing users because the created communication path allow the penetration of microorganisms into the tissues, possibly causing severe infections. This presentation reports the case of complication after the installation of a piercing in the region of left lower lip. A 21-years-old Caucasian patient from the city of Bauru-SP was sent the Unimed Hospital in the same city, having put a piercing four days before. The main complaint was that it was \”buried\” in the lip and that it caused pain when was palpated. The patient reported that had sought the professional responsible for placing the jewel, and he said that it was not possible to remove because of the place was inflamed. On physical examination, the presence of the object was noted extraorally, surrounded blood, purulent drainage and mild swelling. The intra-oral examination revealed a swelling on site, with slightly erythematous mucosa, where the object was covered by connective tissue. The treatment of choice was the surgical removal of foreign body under local anesthesia in the outpatient facility, preceded by antibiotic prophylaxis with Amoxicillin 2g, 1 hour before surgery. Analgesics and antibiotic were for prescribed for seven days postoperatively, until the removal of suture, which was made without any signs of localized infection or complications.

## 148 Etiology of tooth darkening and its resolution by bleaching techniques

CARACANHA, R.B. (Romeu Baco Caracanha Junior romeujr_caracanha@hotmail.com); DANIELETTO, C.F.; LIMA, J.P.G.; PASCOTTO, R.C.; UBALDINI, A.L.M.; NUNES, M.C.P.

Faced with a culture that values the esthetics and considering the increasing technical advance of dentin, there is a greater demand on the perfect smile. In this way, the shape, alignment and especially the color of teeth are very valued. The causes of tooth color changes vary. Indication and efficiency of the bleaching process will depend on the cause of darkening, which may be due to intrinsic or extrinsic factors. There are two techniques for whitening darkened teeth: internal bleaching for teeth with endodontic treatment, and external bleaching, which can be used for both vital and nonvital teeth. The external bleaching techniques include: home or office, and in both the main bleaching agents are 10% carbamide peroxide and 35% hydrogen peroxide, respectively. Tooth bleaching is not a miraclous treatment; it depends of the patient's collaboration, habits and tooth colors. It is important to emphasize the possibility of some side effects such as dentin hypersensitivity, irritation to soft tissues, unpleasant taste, which disappear with the interruption of treatment. Considering that the success of bleaching treatment depends on a correct diagnosis of the change of color and the technique employed, the objective of this work is to present and discuss the etiology of the discoloration and the effectiveness of techniques currently employed for the bleaching of vital teeth, highlighting the main risks and precautions to be taken during the clinical procedure.

## 149 Experimental apical periodontitis induced by *Enterococcus faecalis*

ZAPATA, R.O. (Rafael Ordinola Zapata ronaldordinola@usp.br); BRAMANTE, C.M.; CAMPANELLI, A.P.; MORAES, I.G.; BERNARDINELI, N.; GARCIA, R.B.

The aim of this study was to evaluate the ability of *Enterococcus faecalis* to induce apical periodontitis in root canals of dog's teeth. Fifity premolar and 14 maxillary incisor root canals were used. After 60 days of inoculation, the root canals were evaluated by microbiological and radiographic methods. The root canals were divided in 2 experimental groups according to the *Enterococcus faecalis* ability to colonize the canals as the single strain or by the presence of contaminants from the oral microbiota (mixed infection). Periapical radiographs determined the presence of apical periodontitis and the dentin infection was verified using the acridine orange stain technique and confocal laser scanning microscopy (CLSM). Apical periodontitis was resent in all the root canals evaluated. CLSM analyses showed necroses of the dental pulp and dentin infection characterized by the presence of bacteria inside the dentinal tubules and the presence of bacterial biofilms in root canal walls.

## 150 Fluoride concentrations in recommended soy-based foods by Pediatricians and Nutritionists of Bauru, Brazil

GARCIA, R.P. (Rudan Paraiso Garcia paraizo@gmail.com); CARVALHO, C.A.P.; NICODEMO, C.A.Z.; CARVALHO, F.S.; BUZALAF, M.A.R.; SALES-PERES, S.H.C.

FAPESP- 07/03259-4 e 06/06888-0 e CAPES.

The aims of this study were to determine the fluoride concentrations in manufactured soy foods and to evaluate the possibility of development of dental fluorosis by the consumption of these foods. Twenty Pediatricians and 20 Nutritionists from Bauru-SP answered a questionnaire about the most recommended soy foods in cases of lactose intolerance or cow's milk allergy. Three lots of the 10 most cited foods were purchased, all manufactured foods. Fluoride concentrations were determined by HMDS-facilitated diffusion, using a fluoride ion-specific electrode (Orion 9409). Fluoride concentrations ranged between 0.03 and 0.50 μg F/mL. It was observed statistical difference in the fluoride concentrations among different lots of the same brand in 6 commercial analyzed. Manufactured soy foods analyzed in this study do not contribute separately to the risk for development of dental fluorosis, when reconstituted with non fluoridated water. However, the risk should be considered if these foods were reconstituted with fluoridated water, and the fact that the intake may occur several times a day, beyond other sources of fluoride consumed in this period. It was observed a wide variation in fluoride concentrations among different lots of the same brand, which reflects the necessity for standardization of the fluoride content in these foods.

## 151 The use of In Ceram Alumina crown associated with glass fiber intraradicular post in the esthetic rehabilitation of the smile

MEDEIROS, S.A.S.; REIS, B.R.; CASTRO, C.G.; SOARES, C.J.; SOARES, P.V.; SANTOS-FILHO, P.C.F.

The esthetic viability of restorative techniques involving ceramic associated to conservative aspect of adhesive procedures in different substrates, promotes an esthetic and functional rehabilitation of anterior teeth with extensive structural loss. This report presents a clinical case in which esthetic and functional rehabilitation of a patient with an unsatisfactory extensive restoration in an endodontically treated maxillary central incisor, mispositioned canine compromising the esthetics, and periodontally compromised tooth needing extraction. After extraction of the hopeless tooth and the mispositioned canine, the restorative procedures were initiated. The unsatisfactory restoration in tooth 21 was removed and the placement of a fiber glass post associated with core reconstruction with resin and In Ceram Alumina crown was the choice treatment. The space resulting from the extraction of tooth 24 was filled with an indirect adhesive denture associated with glass fiber reinforcement, reestablishing the function and esthetics. The diastema created after canine extraction was restored with resin. The esthetic of smile associated to functional rehabilitation, promoted great satisfaction to dentist and to patient.

## 152 Knowledge and practices of parents and preschool teachers about oral health of preschool children

HORTENSE, S.R. (Sandra Regina Hortense sandra.hortense@yahoo.com.br); CARVALHO, F.S.; CARVALHO, C.A.P.; BASTOS, R.S.; XAVIER, A.; BASTOS, J.R.M.

The health education has a fundamental role in obtaining good levels of oral health, since the knowledge awakens in individual the interest and the responsibility for health maintenance. The control of main diseases of the oral cavity depends on the knowledge and behavior of people in the habit of hygiene. The purpose of this study was to evaluate the knowledge and practices of parents and preschool teachers from three public preschools in Bauru-SP, related to oral health of preschool children. The sample was composed of 235 parents and 23 preschool teachers. The instrument of analysis was based on a questionnaire composed of objective-descriptive questions, concerning etiology, prevention, oral hygiene practices and source of the information about oral health. The questions were analyzed by descriptive statistics and both groups (parents and teachers) compared by the Mann-Whitney test. Regarding the cause of dental caries, 46.38% of parents and 56.52% of teachers related to poor oral hygiene. Dental caries was considered a disease for 58.72% of parents and 78.26% of teachers. The brushing was supervised by 25.96% of parents at home and 69.57% of teachers after school lunch. For prevention of oral diseases, 22.55% of parents would take their children to the dentist and 39.13% of teachers would indicate the dentist and the adoption of good hygiene habits. The results did not show a statistically significant difference between the groups concerning the questions evaluated (p>0.05). The teachers had good knowledge of oral health, but the parents showed more limitations on the concepts related to dental caries and the importance of attitudes and preventive practices. The interaction of health professionals, teachers and parents can guide new practices and allow the spread of knowledge in oral health for preschool children.

## 153 Study of the adjustment abutment/implant, using plastic Abutment “UCLA”

MARRA, S.T. (Sara Teodoro Marra sarateadoro@hotmail.com); PRUDENTE, M.S.; SILVA-NETO, J.P.; BARBOSA, G.A.S.; SIMAMOTO-JUNIOR, P.C.; NEVES, F.D.

An inadequate fit in the abutment/implant interface can lead to mechanical and biological problem. The laboratory stages could induce misfit in such interface when the castable “Ucla” abutment type is used. The purpose of this study was to comparatively evaluate three different prosthetic laboratories (Laboratories A, B and C) through a vertical and horizontal fit analysis of the castable “Ucla” abutment in the casting and soldering laboratorial stages, of the same clinical case. Four fixed prosthesis were built by each laboratory using abutments of the castable “Ucla” type. The evaluation was conducted based on the photos obtained with a scanning electron microscope at 500x magnification. The results were analyzed statistically (p>0.05). The values in relation to the vertical fit/misfit, after casting, were 95.8% between 0 and 10μm in “A”, and 70.8% and 87.5% in “B” and “C” respectively. The results obtained in the present study, both in relation to the vertical and horizontal fit/misfit were more satisfactory when compared to previous studies, using the same abutments. However more, though more satisfactory, such results are still considered clinically worrisome.

## 154 Clinical evaluation of the dental sensitivity in restorations of non-carious cervical lesions

SCATOLIN, R.S. (Renata Siqueira Scatolin re_scatolin@hotmail.com); OLIVEIRA, F.G.; PITA, D.S.; GUEDES, A.P.A.; MACHADO, L.S.; SUNDFELD, R.H.

The objective of this study was available the preoperative and postoperative sensitivity in restorations of non-carious cervical lesions. Three groups of 40 teeth each were formed, according the materials and techniques. Before application of the adhesive materials, the enamel and dentin of the teeth of groups I, II and III, were etched with 35% phosphoric acid. The specimens of group I received the conventional adhesive system Scotchbond Multi-Purpose (3M) followed by composite resin Z350; group II received resin-modified glass ionomer Fuji II L.C. only. Group III was restored with the same resin-modified glass ionomer, but two layers of ScotchBond Multi-Purpose primer was applied first. The teeth examined before and 1 week after placement of the restorations. Preoperative sensitivity was present in 83.33% of the cases, and absent in the other 16.67% of the cases. After placement of the restorations, sensitivity was absent in 80% of the teeth, but still present in 20% of them. The results obtained suggested that non-carious cervical lesions restoration is an effective clinical procedure for controlling of the dental sensitivity. (Apoio: FAPESP).

## 155 Central giant cell granuloma treated surgically

SCHUTZ, C.Y.K. (Cristiane Yuri Kohiyama Schutz crisyuri@msn.com); KAMEI, N.C.; TRENTO, C.L.

It is presented a case of central giant cell granuloma A Caucasian patient, female, twenty-five years old, presented with large lesion involving the mandibular anterior teeth. Radiographic exams including axial and coronal computed tomography scans showed a radiolucent area with multilocular aspect suggesting differential diagnosis of ameloblastoma, aneurismal bone cyst, fibrous dysplasia and giant cells lesion. Biopsy was made and the presence of multinucleated giant cells and groups of collagen fibers with areas of extravasation of erythrocytes and hemosiderin deposit was detected under light microscopy. For a definitive diagnosis, biochemical tests were made in the blood for determination of the levels of alkaline phosphatase, calcium and phosphorus, and dosage of parathormone, which were all within normal limits. The surgical treatment adopted was curettage of the lesion with extraction of teeth involved in the process. The 1-year follow up after surgery showed no sign of recurrence.

## 156 Unusual extraction protocol due to mini-implant root damage

BARROS, S.E.C. (Sérgio Estelita Cavalcante Barros sergioestelita@yahoo.com.br); JANSON, G.; CHIQUETO, K.

This case report describes a surgical accident due to the inadequate mini-implant insertion in the interradicular septum, causing significant molar root damage. As consequence, the two maxillary premolar extraction protocol initially planned to correct patient malocclusion was changed, and the drilled molar was extracted instead of premolar because its long term prognosis could not be safely predicted. Although the mini-implant anchorage was not replaced and dental anchorage units were reduced due to molar extraction, the dental anchorage reinforcement and patient compliance with Class II intermaxillary elastics allowed achieving excellent static and functional occlusal results. The patient was very satisfied with the dental and facial esthetic improvement. This accident report highlights the root damage risks associated to mini-implant insertion when this surgical procedure is performed in critical sites without any device to guide bone drilling.

## 157 Cosmetic adjustments and direct esthetic restorations used used in esthetic rehabilitation

SILAS, J.B.S. (Silas Junior Boaventura de Sousa paulovsoares@foufu.ufu.br); REIS, B.R.; PEREIRA, F.A.; SVERSUT, R.; SOARES, C.J.; SOARES, P.V.

The main advantage of dental transformation using direct composite resin is the preservation of healthy structure, being thus highly conservative and having its principles based on recent principles of adhesion. In addition, this type of restoration has a great esthetic adaptation, when the procedure of the technique is applied correctly. C.M.A., a female patient, 21 years old, presented to the integrated Clinic of the Dental School, Federal University of Uberlandia with main complaint of the esthetic appearance. The clinical examination showed lack of left canine guidance, altered shape and size of the upper anterior teeth, thus compromising the harmony of the smile. The treatment proposed was dental transformation with direct composite resin restorations. Impressions of both dental arches were maded, followed by waxing and fabrication of a silicone matrix. Next, the following procedures were done: reduction of the buccal, proximal and incisal faces of teeth 13.12, 22 and 23; selection of resin shade; rubber dam placement; checking of the silicone matrix, verifying the fit and the correct palatal contour; acid etching; application of adhesive system; filling of the silicone matrix with composite resin in order to construct a palatal guide followed by resin polymerization inside the matrix; incremental insertion of composite resin respecting dental shape and contour; occlusal adjustment and finishing and polishing. The presence of teeth with modified anatomy causes esthetic disharmony and promotes a functional bio-psychological-social discomfort to the patient. So, dental transformation restored the harmony of the smile and increased quality of life of the patient.

## 158 Tooth developmental alteration due to trauma to the deciduous dentition

CHADI, S.F. (Sílvia Fernandes Chadi silviafasak@hotmail.com); OLTRAMARI-NAVARRO, P.V.P.; NAVARRO, R.L.; CONTI, A.C.C.F.; ALMEIDA, M.R.; ALMEIDA, R.R.

Dental trauma is defined as a lesion presenting different grades of extension, intensity and severity. As to its origin, it can be accidentally or intentionally caused by forces acting on dental organ derived from traffic collisions, falls, aggressions, contact sports injuries and others. The majority of these lesions are presented in upper arch, and central incisors are most frequently involved. It is important to perform a complete health questionnaire and a proper examination of intra and extra oral structures during the first patient care. When a trauma occurs in the deciduous dentition, dentists must alert parents about a possible morbidity of intrusion movements of deciduous teeth toward the developing permanent teeth. Alterations such as stained surfaces, abnormal shapes and alterations in eruption could follow. This study aims at describing the correct diagnosis and treatment of a deciduous tooth trauma case in which the patient suffered a trauma on her deciduous dentition with a consequent developmental alteration in the permanent upper central incisor. The patient searched for orthodontic treatment complaining of the absence of the upper central incisor, even after an attempt of orthodontic traction. Medical history was related from her mother with special attention to the mechanisms of trauma. With the aid of the radiographic exams, it was diagnosed the presence of the upper central incisor in an unusual shape and position with a severe deformity that excluded any possibility of orthodontic movement. Though, the treatment was planned with the extraction of the tooth and graft positioning for posterior rehabilitation with osseointegrated implants in the same area. After completion of the orthodontic treatment, a retainer with provisional prosthesis was installed to maintain the space for future rehabilitation.

## 159 Evaluation of the biocompatibility of repairing root canal sealers using subcutaneous implants

WATANABE, S. (Simone Watanabe mone_wata@yhaoo.com.br); GOMESFILHO, J.E.; RODRIGUES, G.; BERNABÉ, P.F.E.; LODI, C.S.; GOMES, A.C.

The purpose of this study was to evaluate the subcutaneous response of rat connective tissue to CER (Cimento Endodôntico Rápido or fast endodontic cement) and Ângelus MTA®. These materials were placed in polyethylene tubes and implanted into dorsal connective tissue of Wistar rats for 7, 30 and 60 days. The specimens were prepared to be stained with hematoxylin and eosin or Von Kossa or not stained for polarized light. The presence of inflammation, predominant cell type, calcification, and thickness of fibrous connective tissue were recorded. Scores were defined as follows: 0, none or few inflammatory cells, no reaction; 1, <25 cells, mild reaction; 2, 25 to 125 cells, moderate reaction; 3, >125 cells, severe reaction. Fibrous capsule was categorized as “thin” when thickness was < 150 ìm and “thick” at > 150 ìm. Necrosis and formation of calcification were recorded. Results were analyzed statistically by Kruskal Wallis tests. Both materials Ângelus MTA® and CER caused moderate reactions at 7 days which decreased with time. The response was similar to the control at the 30th and 60th days with Ângelus MTA® and CER characterized by organized connective tissue and presence of some chronic inflammatory cells. Mineralization and granulations birefringent to the polarized light were observed with both materials. It was possible to conclude that CER was biocompatible and stimulated mineralization.

## 160 Oral and general health conditions of female drug users attending a detoxification program

ABATE, S.P.A. (Stefania de Paula Assunção Abate stefaniaabate@hotmail.com); LINS, A.S.; TOLEDO, H.B.; CIESIELSKI, F.I.N.; GAETTI-JARDIM JR, E.

In recent decades there has been a staggering increase in the consumption of drugs with psychotropic effects, such lawful as tobacco or alcohol, or illegal, such as cocaine, heroin, crack, solvents, ecstasy, among others, making it a serious public health problem in Brazil and the rest of the world. The present investigation evaluated the health conditions of 50 female drug-users (age 29.88 ± 9.3 years on average) attending a program for drug detoxification, in the city of Santa Fé do Sul, in comparison to a control group of non-dependent females presenting similar age and socioeconomic conditions. Socioeconomic conditions, history of consumption of licit or illicit drugs and patterns of association of these compounds, as well intra and extra oral examinations were carried out. Clinical parameters were subjected to multiple comparisons and dichotomous variables were analyzed by mean of non-parametric Mann-Whitney test. Of the 19 chemical dependent patients had generalized gingivitis, 12 had chronic periodontitis, 13 were periodontally healthy, while six wore complete dentures. The control group patients, 11 were generalized gingivitis patients, one had periodontitis, 36 were periodontally healthy, while two were complete denture users. Ischemia of palate mucosa was observed in most of drug users. The report of the occurrence of back pain, skin and respiratory infections, headache, fatigue, dyspnea, polyuria, xerostomia, tingling of the extremities, anemia, allergies, arthritis, polydipsia, occurrence of periodontitis, weight loss and impairment of the repair process were statistically more frequent in the group of patients with chemical dependence, compared with the control group. There was no statistically significant difference between groups in the oral hygiene standards.

## 161 Treatment of transverse discrepancies in skeletal class II and class III malocclusion

PEREIRA, S.C.C. (Suelen Cristina da Costa Pereira sucristina@hotmail.com); PIERI, L.V.; HENRIQUES, J.F.C.; PINZAN, A.; JANSON, G.; FREITAS, M.R.

Maxillary and mandibular transverse discrepancy corrections in Class II and Class III malocclusions will be showed with two report cases in growing patients that treated orthopedically and/or orthodontically associated or not with fixed appliances. Rapid maxillary expansion (RME) was associated with facial mask in the dentoskeletal Class III treatment. In the vertical growth pattern should wear a high-median headgear (IHG) for vertical control. The orthodontic mandibular expansion with Schwarz appliance upright the posterior lower teeth in the apical base creating a barrier avoiding RME relapse. Retention with an orthopedic or orthodontic removable appliance, associatted or not with fixed appliance, must be done for time based on the quantity of the obtained expansion in order to achieve treatement stability.

## 162 Facial lesions with dental involvement caused by traffic accidents based on the forensic reports of the Forensic Medicine of Taubaté/SP

NARESSI, S.C.M. (Suely Carvalho Mutti Naressi@fosjc.unesp.br); BARBIERI, A.A.; SCORALICK, R.A.; MAROTE, I.A.A.

Statistical studies made in several areas of human knowledge and the widespread nature of social scientific research have greatly contributed to the knowledge and application of solutions to situations that, somehow, are part of people's daily lives. The reform of the Brazilian Traffic Code minimized the rate of automobile accidents with fatalities and/or serious injuries due to the requirement for some safety items. However, the prevalence of sequelae from this type of accident is still high and the face is one of the most affected regions, with repercussions even for the social life of the traumatized individuals. This analysis was based on the forensic reports of the Forensic Medicine of Taubaté/SP, made by medical forensic experts, about the facial injuries with dental involvement caused by traffic accidents between January 2005 and December 2007, focusing on the region, age and gender and most common type of injury among those with dental involvement. Of the 12,184 (twelve thousand one hundred and eighty-four) reviewed reports, traffic accident was the cause of facial involvement in 4.82% (588), with reports of dental involvement in only 0.63% (77) of these cases. The most affected facial region was the jawbone, the higher occurrence of dental injury was the fracture, accounting 9.01% of cases, and males as the most affected, representing 6.80% of cases. The most affected age group for females was between 16 and 24 years (2.21% of cases) and for males, between 24 and 32 years (1.87%). The proportion of facial injuries in the dental involvement suggests under-reporting, in addition to inappropriate use of nomenclature to describe the lesion, compromising the damage valuation and its consequences.

## 163 The effect of green tea (*Camellia Sinensis*) on the glycemic profile and body mass of normal and streptozotocin-induced diabetic rats

OLIVEIRA, T.V.Z. (Tamiris V. Z. de Oliveira tamirisvallim@hotmail.com); MENEGHETTI, I.C.; GENNARO, G.; RODRIGUES, P.A.L.; MARCHESANO, L.H.; ASSIS, G.F.

Diabetes Mellitus is the most common endocrine disorder. It is the result of a malfunction of insulin-dependent glucose and lipid metabolisms. Diverse diabetic experimental models have been used targeting an effective treatment. Purpose: The objective of this study was to investigate how the Green Tea (*Camellia Sinensis*) acts on the control of the glycemic profile and body mass of normal and streptozotocin-induced diabetic rats. Forty male rats had diabetes induced by streptozotocin injection (50mg/kg body weight) and forty healthy male rats were maintained as control group. Diabetic and control groups received water or green tea treatment and then they had their weight and glycemic level analyzed at 15, 30, 60 and 90 days. The control group increased weight, while the diabetic group lost weight, irrespectively of green tea treatment or water intake. Blood glucose levels decreased in diabetic rats treated with green tea only at 15 days, while, there were not statistic differences in other periods when compared diabetic with control group. In conclusion, the Green tea showed a positive effect on the glycemic level in diabetic rats only at initial period of treatment, and did not confirm effects on the prevention of weight loss in diabetic animals.

## 164 Treatment of amalgam tattoo associated with gummy smile

MISKO, T.P. (Tárcio de Pádua Misko varteahmed@hotmail.com); MAIA, L.P.; SOUZA, S.L.S.; REINO, D.M.

Gummy smile is an esthetic problem that can be caused by vertical maxillary overgrowth. It is characterized for altering teeth-gingival tissue proportion resulting in a discrepant exposure of gingival tissue during the patient smiling. Impairment to smiling is severely associated to amalgam tattoos implying into major esthetic complaints from patients in this condition. Amalgam tattoos are blue, gray, or black colored lesions caused by iatrogenic insertion of amalgam particles into the soft tissues. Several treatment techniques for gummy smile are available, such as gingivectomy and orthognathic surgery. However, gingivectomy procedures increase vertical teeth size, which makes the procedure inadvisable for a great number of cases. Onward, patients frequently abstain from orthognathic surgeries due to more expensive, complex, and invasive procedures. The management of labial frenum technique has been indicated for esthetic improvement of excessive gum tissue smile lines, unadvised for gingivectomy or orthognathic surgery. Therefore, the aim of this study is to demonstrate the use of the esthetic management of upper labial frenum. Female patient with gummy smile and large amalgam tattoo in teeth 12 and 13 reported great esthetic complaint. Supragingival scaling, plaque control and oral hygiene orientation were performed. Surgical treatment was performed after achieving plaque index less than 20%. Incisions were made over the mucogingival junction; a split-thickness flap was elevated, residual amalgam was removed, and the lip was coronally positioned. Evaluations were taken after one week, two weeks, one month, and six months. The amalgam tattoo disappeared and the gummy smile was reduced leading to esthetic improvement. Eventually, the undertaken procedure was effective for the treatment of amalgam tattoo associated to Gummy smile, improved the esthetics of the patient's smile line.

## 165 Patients with eating disorder: tooth wear and possible etiologic factors

SILVA, T.C. (Thais Cristina da Silva thais.cristina.silva@usp.br); MARSICANO, J.A.; JULIANELLI, J.; SALES-PERES, A.; BASTOS, J.R.M.; SALES-PERES, S.H.C.

The eating disorders (periodical compulsive eating disorder., anorexia, bulimia nervosa) is increasing in incidence and prevalence, becoming a real public health problem. This study investigated the prevalence and severity of dental wear and the possible etiologic factors in patients with anorexia and bulimia nervosa treated at the Hospital das Clínicas de Ribeirão Preto – SP. The sample was formed by 60 patients, divided in two groups: experimental group (G-1) – patients with eating disorder (n=30) and control group (G-2) – patients attend in the same hospital, due to other causes (n=30). The occurrence of dental wear was evaluated by IDD index and an exploratory questionnaire about etiologic factors (hygiene and feeding habits, gastroesophageal reflux, clenching and grinding of teeth, bruxism and mastication) was applied. The analysis of the data was descriptive and analytical, using the Mann-Whitney test at a level of significance of 5%. There was a mean number of 19.93 worn teeth in G-1 and 23.4 in G-2. On severity, the involvement of dentin was identified 159 times in G-1 and 134 times in G-2 for anterior teeth, and 39 times in G-1 and 25 times in G-2 for posterior teeth. Among the etiological factors studied and tooth wear there was significant association with tooth clenching and grinding (p<0.05). There was no statistically significant difference between the studied groups. The conclusion is that patients with eating disorders present dental wear, with involvement of dentin that favors dental hypersensitivity.

## 166 Contribution of CBCT in the anatomic identification of the mental region

IMADA, T.S.N. (Thaís Sumie Nozu Imada thaimada@usp.br); RUBIRABULLEN, I.R.F.; DEZOTTI, M.G.; FERREIRA JÚNIOR, O.; CAPELOZZA, A.L.A.; CARVALHO, P.S.P.

Cone beam CT (CBCT) has changed the way we approach diagnosis and treatment particularly when anatomy of the maxillofacial complex is an important features, in particular for implant planning. Viewing the mandible in all three dimensions helps us extract the maximum information needed. The mental region may receive various surgical procedures including grafting procedures and the number of postoperative complaints has been rising, among them hemorrhage. Thus, the presence of neurovascular structures should be carefully assessed prior to surgery. The superior and inferior genial spinal foramina and their bone canal are situated in the midline of the mandible. In dissections, it was identified branches of the lingual artery and vein, lingual nerve, mylohyoid nerve altogether with branches or anastomoses of the sublingual and/or submental artery and vein, inside those foramina. This case discusses the contribution of CBCT images to the identification of the anatomic structures in the mental region. The patient was subjected to CBCT exam for a implant planning, even though the mental region was not the implant target, it was observed a variation in the trajectory of the bone canal inside the genial spinal foramina. The images revealed that the bone canal that accompanies the foramina had a curved trajectory inside the mental region connecting the mandibular lingual cortical to the mandibular base. The images seen in the panoramic radiograph could not suggest such trajectory for the bone canal, otherwise, the panoramic image showed as if there were a bone sclerosis in the mental region, which was proven not to be true in the CBCT images.

## 167 “*In vitro*” evaluation of bacterial leakage in root canals obturated with different techniques

CUNHA, T.V.R.N. (Thaís Vieira Rizzo Nunes da Cunha tvrizzo@gmail.com); BROSCO, V.H.; BRAMANTE, C.M.; MORAES, I.G.; BERNARDINELI, N.; GARCIA, R.B.

The aim of this study was to evaluate the coronal bacterial leakage and penetration of bacteria into the root canals and dentinal tubules in root canals obturated by different techniques. The canals of palatal roots of one hundred and sixty maxillary molars were instrumented and divided into different groups, according to the obturation technique applied -lateral condensation, Microseal system, Touch'n heat + Ultrafil system, and Tagger's hybrid technique- and the extension of the remaining obturation material (5mm and 10mm). Ten additional roots were employed as control. The culture medium BHI was inoculated with the microorganism Enterococcus faecalis and placed in contact with the coronal portion of roots; the same sterile culture medium was placed in contact with the apical portion of roots. Leakage was evaluated daily, for 120 days, and was considered when the culture medium at the apical portion was rendered turbid. The roots were microscopically analyzed for evaluation of bacterial penetration into the root canals and dentinal tubules. In the leakage test, the Tagger's hybrid technique, exhibited the worst sealing ability. Root canals with 10mm of remaining obturation material presented better sealing than canals with 5mm. Microscopic evaluation revealed that the lateral condensation technique allowed lower penetration of bacteria into both root canals and dentinal tubules, followed by the Touch'n heat + Ultrafil, Microseal and the Tagger's hybrid technique. Root canals with 10mm of remaining obturation material presented similar bacterial penetration as root canals with 5mm.

## 168 Chlorhexidine solutions in dental bonding procedures: a recommendation to reach great durability

PINTO, T.A. (Thales Tato de Assis Pinto thalestato@usp.br); SILVA, L.M.; MARTINS, L.M.; HANNAS, A.R.; FRANCISCONI, P.A.S.; WANG, L.

Activated matrix metalloproteinases (MMPs) present the capacity to provoke collagen fibrils degradation, which may compromise the hybrid layer. Recently, it has been shown that chlorhexidine has a potential ability to inhibit MMPs that were activated after dentin conditioning in bonding procedures. This study aims to present some literature reports that attest this positive action. Based on thi evidences, a new clinical bonding protocol has been considered, which includes an aqueous chlorhexidine solution conditioning after phosphoric acid etching and rinsing. In addition, biocompatibility and toxicity of this product will also be discussed. According to this scenario, chlorhexidine seems to offer a great perspective in order to increase the longevity of adhesive restorations. This case report will show a restorative procedure following the protocol of use of chlorhexidine. After tooth preparation, etching was performed using phosphoric acid for 15 seconds in dentin and 30 seconds in enamel. Acid was completely rinsed off and 2% chlorhexidine digluconate was applied for 60 seconds, which was gently dried with absorbent material, followed by application of the adhesive system according to a moist-bonding technique, and restored incrementally with composite resin systems.

## 169 Paracoccidioidomycosis: case involving several regions of the mouth

MACHADO, T.A. (Thiago Alves Machado thiagoameba@hotmail.com); CARDOSO, C.L.; DAMANTE, J.H.; BARRETO, J.A.; TAVEIRA, L.A.A.

A 48-year-old male had main complained of having “ulcers in the mouth” for 20 years. He expressed fatigue and had difficulties to talk and swallow. He was a smoker and consumed alcohol. Mulberry-like ulcerations were observed in the mouth, especially in the buccal mucosa, palate and internal face of the lips. Based on the clinical features, the presumptive diagnosis was of paracoccidioidomycosis. A chest radiograph and a biopsy were performed. The radiograph disclosed moderate alterations in the lungs and normal cardiac area. The microscopic characteristics showed hyperplastic parakeratinized stratified squamous epithelium with areas of exocytosis. A densely collagen-rich connective tissue with intense inflammatory infiltrate was observed underlying this region. Moreover, epithelioid macrophages and multinucleated giant cells with birefringent yeasts were found. The diagnosis of paracoccidioidomycosis was confirmed. The patient was referred to the Hospital Lauro de Souza Lima for systemic treatment. Three months after treatment with Itraconazol the oral lesion had disappeared with improvement of the general oral health condition. The patient is still being followed up.

## 170 Analysis of the fracture strength of external hexagonal implant system: regular and narrow diameter

CARNEIRO, T.A.P.N. (Thiago de Almeida Prado Naves Carneiro thiago_paulista_@hotmail.com); SILVA-NETO, J.P.; PRUDENTE, M.S.; PRADO, C.J.; MUNDIM, A.R.; NEVES, F.D.

The precursor system of the contemporary implantology, called Bränemark System, was based on a configuration with three parts, the implant, the abutment and prosthesis, connected by screwed junctions. In the platform of the implant, it has a external hexagon where the abutment is connected, this implant has a diameter of 3.75mm and is used since the mid 1960's. This implant is considered the most studied of all times and longitudinal monitoring has shown its risk of fracture, estimated between 0.1 and 0.2%. In the mid 1990's, implants with smaller diameter were introduced and have been used since then. The objective of this study was to define proportionally, how much the implant loses in resistance when its diameter is diminishes from 3.75mm to 3.3mm (majority of the national systems). For this, fracture strength testing was applied to 9 samples (n=9) of each group. The maximum force was evaluated by flexion test with a perpendicular force applied to the long axle of the implant on a mechanical testing machine. From the generated graphics, the corresponding force (N) to the fracture of the implant was determined. The data analyzed statistically by one-way ANOVA and and Tukey's test (P< 0.05) showed that the Regular group (3.75mm) presented statistically significant higher fracture strength values than the Narrow group (3,3mm). The mean value for Narrow group was 261.5N, which is equivalent to approximately 70% of the resistance of the Regular group (378.1N). These data suggest that the use of narrow implants in possibly overloaded areas can increase considerably the risk of fracture, thus its clinical indication should be limited to upper lateral incisors and mandibular incisors when the placement of regular implants is not possible.

## 171 Contemporary orthodontic treatment

LIMA, T.F. (Thiago Freire Lima lima-thiaguinho@uol.com.br); OLIVEIRA, R.B.S.; JANSON, G.

The Bioefficient therapy is an orthodontic technique performed with Viazis bracket system in association with state-of-the-art orthodontic wires including contemporary concepts of tooth movement. This bracket system was developed in order to take good advantage of the elastic capacity of thermoactivated superelastic orthodontic wires, reducing even more the force transmitted to the teeth and increasing the amount of wire activation. This technique aims to spend shorter time on the initial stages of the treatment, employing as much as possible the properties of the thermoactivated superelastic “Bioforce Ionguard” NiTi wires. These wires deliver very low force and in association with an innovative bracket design allow that the stages of leveling, aligning, space closure can be accomplished simultaneously because rectangular wires can be inserted into the brackets at the beginning of treatment. Therefore, this therapy enables the professional additional time for the final detailing of the static and functional occlusions. The present study demonstrates the treatment planning and mechanical sequence of two clinical cases in which the treatment plan comprised extractions and was conducted with the Bioefficient therapy.

## 172 Inter-relationship of peridontics and operative dentistry in the esthetic rehabilitation of smile

TOLENTINO, A.B. (Andrea Barros Tolentino paulovsoares@foufu.ufu.br); PEREIRA, F.A.; SVERSUT, R.; PAULILLO, L.A.M.S.; SOARES, C.J.; SOARES, P.V.

Dental esthetics associated to function has probably been one of the factors of greatest emphasis in dentistry today. The esthetic viability of restorative techniques with composites associated to the conservative aspect of procedures in enamel and adhesives in conjunction with periodontal surgery can therefore bring greater satisfaction to the patients regarding their smile and to improve their social skills. Patient A.B.T. 21 years old, female, came to Specialization Clinic in Restorative Dentistry, Dentistry School, Federal University of Uberlandia (FOUFU) complaining about the presence of diastema and great gingival volume. The clinical examination showed that the patient had teeth with altered shape and size. In addition, it was observed that the insertion of the labial frenum was one of the reasons for the presence of diastema. For the implementation of treatment, it was defined the need for use of elastic spacers between teeth 21 and 22, followed by plastic periodontal surgery and correction of the insertion of the labial frenum. After 21 days of healing, impressions were taken for fabrication of a cast model and subsequent waxing and fabrication of a silicone matrix that would serve as a guide for the restoration. Composite resin shade was chosen using the VITAE scale and the restoration was carried out using the technique with layers of different shades in order to reproduce the dental structure. The finalization of the case was the polishing of restoration to obtain a smooth surface, avoiding the accumulation of plaque and facilitating cleaning. A simple, direct, minimally invasive and low-cost restorative technique provided a more harmonious smile with excellent esthetics and function. This allied to the periodontal plastic surgery brought satisfaction for the professional and patient.

## 173 Esthetic rehabilitation of the smile using direct composite resin laminated veneers

UBALDINI, A.L.M. (Adriana Lemos Mori Ubaldini dri_ubaldini@hotmail.com); PINI, N.I.P.; BENETTI, A.R.; PASCOTTO, R.C.; NUNES, M.C.P.; DANIELETTO, C.F.

The constant urge for natural esthetics associated with the development of efficient and reliable restorative materials have guaranteed conservative esthetic techniques in operative dentistry. Indeed, direct composite resin laminated veneers have become a routine treatment alternative in dental offices when it is necessary to restore discolored or misaligned anterior teeth. Successful esthetic results are dependent primarily on understanding the proper indication and performing of this technique. An adult female patient searched for treatment at the dental clinic of Maringá State University (UEM) complaining about the misalignment and severe staining of teeth 11 and 21. Clinical examination and radiographs confirmed the debility of the dental structures due to the presence of several restorations and endodontic treatment. The treatment consisted of cementation of posts and restoration with direct composite resin (Opalis, FGM) laminated veneers associated with opaque resin (Opak, Angelus). The treatment reestablished the harmony of the smile while preserving functional integrity and periodontal health, other than providing psychological comfort to the patient. Therefore, this clinical report intends to present and discuss the indication, advantages, and disadvantages of direct composite resin laminated veneers.

## 174 Dental erosion from different beverages: alteration on dental surface and implications to bonding procedures

DREIBI, V.M. (Vanessa Dreibi vanessa_dreibi@hotmail.com); HIPÓLITO, A.C.; CASAS-APAYCO, L.C.; MAGALHÃES, A.C.; HONÓRIO, H.M.; WANG, L.

Literature has been presented evidence of growing incidence of dental erosion. The main etiologic factors involve intrinsic and extrinsic reasons. It is licit to affirm that beverages are the commonest extrinsic agent provoked due to the modern society' s lifestyle, which are responsible for their high consumption. The aim of this study was to show evidence of structure and composition of enamel previously eroded by different beverages and how it can alter the establishment of the hybrid layer. Enamel blocks obtained from bovine incisors were prepared in dimensions of 4mmx4mm of surface. Only specimens with approximately 350 KHN were selected, as determined with a microhardness tester (25gF for 10s). The specimens received the application of an etch-and-rinse bonding system (Adper Single Bond 2), previously modified by addition of rhodamine (0.16g/mL). Reconstruction was made with a nanotechnology composite resin (Filtek Z350). After 24 hours in water storage at 37°C, they were bisected longitudinally and polished. Laser confocal microscopy was used to analyze the hybrid layer characteristics. Different patterns of hybrid layer formation were observed, which can implicate clinical differences.

## 175 Epstein-Barr virus and cytomegalovirus and radiotherapy: relationship with mucositis

SILVA, V.F. (Vanessa F. da Silva vanefs88@hotmail.com); GALLO, A.J.; GAETTI-JARDIM JR, E.

FAPESP 07/54851-0.

Viruses of the family Herpesviridae have been associated with exacerbation of several oral infectious diseases such as periodontal and periapical diseases. In addition, Epstein-Barr virus (EBV) and Cytomegalovirus (CMV) are able to produce acute, persistent and chronic infections in different cell lines such as epithelial cells, lymphocytes and macrophages, leading to the development of suitable condition for microbial colonization of periodontal tissues. The present investigation evaluated the occurrence of these viruses in saliva, oral mucosa and biofilms from patients undergoing radiotherapy (RT) for treatment of head and neck cancer. Initially, these clinical samples were collected from 50 patients aged 16 to 80 years, while the achievement of clinical specimens. Sample collection was performed at baseline (prior radiotherapy), immediately after radiotherapy, 30, 90 d. after RT and 6 months after radiotherapy. Then total DNA was extracted by using Qlamp DNA Mini Kit and the presence of DNA of these two viruses was evaluated by nested PCR. The results showed the presence of EBV virus and CMV in 10% and 12%, respectively, before radiotherapy, while after radiotherapy, 88% of patients presented oral mucositis, and 44% harbored EBV in oral cavity and the same was detected in 54% for CMV. None of the patients who did not develop mucositis showed infection by CMV and EBV viruses at the time of collection of clinical specimens. The results of this study suggest that the cellular events and tissue involving radiotherapy for patients with head and neck cancer may be closely related to recurrence or reactivation of herpetic infections, which can affect the immune response of the patient and therefore the development of diseases associated with opportunistic pathogens.

## 176 Health integral and integrated actions in schoolchildren of the Rio das Pedras Settlement

MACHADO, V.R. (Vanessa Rodrigues Machado vanessarmm@yahoo.com.br); ARCIERI, R.M. MARRA, E.M.O.; AZEVEDO, M.R.; LOUREIRO, R.M.T.; ONO, R.

It is about a University Extension Project suggested for several graduation course of health area of Federal University of Uberlândia (FUU) that competed and have been select for PROEXT 2006, program that contain programs and projects with emphasis on the social inclusion that strengthen the institutional act of extension in the ambit of the University Institutions. The purpose of this project is to give opportunity to the FUU dental students for extension activities in an integrated manner and with participation of different disciplines searching the social inclusion of 6-14-year-old students living in the rural zone by means of attention to their health and the implantation of public health policies permitting a behavioral and educational change of the future health professional. The UFU, by the Dentistry (5 professors and 47 students); Medicine (2 professors and 18 students) and Nursing (2 professors and 12 students) courses developed educational, preventive and curative actions directed at schoolchildren (6 to 14 years old). These project was developed on two ways: a) educational and preventive/ in the Rio das Pedras Settlement of Uberlândia/MG and Dom Bosco Rural Municipal School; b) Curative/ preventive assistance in the Medical/Dental outpatient service installed by the PACTo Project in the Rio das Pedras Settlement. Eight-seven families of the Settlement were assisted and 66 medical/dental consultations were provided to children aged 6 to 14 years. The actions at school included supervised oral hygiene, health education, chats, workshops, and enrolled 244 students, improving their health quality and rescuing their citizenship. The Project allowed that our students live the university extension, comprehending its challenge, the curricular amplification and the involvement between many disciplines through the relation and orientation at target population.

## 177 Reconstruction with screw and titanium mesh screen in a case of fracture of the left orbital floor

VIADANNA, A.P.O. (Ana Paula de Oliveira Viadanna apaulav31@hotmail.com); OLIVEIRA, N.C.M.; BATISTA, J.D.; SARDINHA, M.C.; ZANETTABARBOSA, D.; SILVA, C.J.

Facial trauma frequently results in damage of soft tissue and bone components of the facial skeleton (mandible, maxilla, and other). Besides, those injuries are usually associated to injuries in other parts of the body. The etiology of the facial fractures includes automobile accidents, physical assaults, falls, sporting and occupational accidents. The treatment and rehabilitation of this patient involves a detailed comprehension of evaluation, diagnosis and surgical technique. The facial fractures can be classified as mandibular fractures and fractures of the middle third of the face that includes maxilla, zygomatic and naso-orbito-ethmoid complex bones. We describe a case of a 20-year-old woman who has presented to Uberlândia Federal University with a history of bicycle accident. After a clinical and radiographic examination she was diagnosised with zygomatic fracture. During the clinical exam, the patient complained about paresthesia, diplopia and difficulty of ocular movement. The patient reported that she visited an eye specialist which associated the case to the imprisonment of the extrinsic eye musculature. Radiografic exams were requested and showed fractures of the floor left orbita. The patient was subjected to the reconstruction of the orbital floor with screw and titanium mesh screen. At 45 days postoperatively, the patient was well without complaints.

## 178 Rapid maxillary expansion anchored by implants – a new proposal to orthopedic expansion in the permanent dentition

PICCOLI, V.D. (Vicente Dias Piccoli vipiccoli@hotmail.com); GARIB, D.G.; NAVARRO, R.L.; FRANCISCHONE, C.E.; OLTRAMARI-NAVARRO, P.V.P.; AYUB, P.

The rapid maxillary expansion through midpalatal suture opening is an efficient method to correct the upper arch morphology. However, this procedure often results in undesirable buccal tipping of the posterior teeth supporting the expansion appliance. This orthodontic effect accounts for about half of the expansion screw opening in deciduous and mixed dentitions and two-thirds of that in the permanent dentition. This study presents a method for maxillary orthopedic expansion, in the permanent dentition, using implants as anchorage. This method is illustrated here by a case report of a 14-year-old female with a Class I malocclusion and unilateral posterior crossbite. The treatment plan consisted of rapid maxillary expansion using a Hirax expander, supported by the permanent first molars and by palatal implants placed bilaterally between the first and second premolars. The rapid maxillary expansion supported by palatal implants presented similar orthopedic effects to that of conventional tooth-supported expanders, but there was significantly less buccal tipping of the maxillary posterior teeth. Thus, the procedure reduced the risk of negative periodontal sequelae.

## 179 Intracanal adhesive luting: achieving a successful treatment

MARQUES, V.R. (Vinícius Rizzo Marques sarna@usp.br); BARBÉRIO, G.S.; SILVA, L.M.; LORENZONI, F.C.; MARTINS, L.M.; BONFANTE, G.

The adequate selection of the luting cement is essential to prevent failures due to dislodgement of posts. Adhesive luting of pre-fabricated posts promotes improvements as color stability, mechanical resistance and low solubility. However, its main contribution would be the achievement of a unique biomechanical complex (monoblock) by the adhesion of all reconstructive constituents (post, luting cement, core material) and dental structure. Thus, tooth and restoration become a single structure and stress would be distributed more evenly to all constituents. However, obtaining this single body becomes more complex when a larger number of materials are incorporated. Besides the limitations of the adhesive luting agent inside root canal space, such as polymerization and moisture control, some incompatibility may occur between chemical or dual cured resin cements and simplified adhesive systems. This chemical incompatibility avoids the adequate polymerization of resin cements and these adhesive systems, naturally permeable to dentin fluids, promote an instable and weak adhesion. This work aimed at discussing the problems related to adhesive luting and their possible solutions, presenting a case report and a brief review of literature.

## 180 Immunolocalization of osteocalcin on healing of autogenous bone block in an ovariectomized aged and young rat model

SILVA, V.C. (Viviane Clicie da Silva leandroataunesp@ig.com.br); BOSCO, A.F.; OKAMOTO, R.; MURAKAWA, A.C.; MORAES, R.O.; FERNANDES, L.A.

The aim of this study was to evaluate the influence of the ovariectomy (estrogen depletion), on healing process of autogenous bone block grafts in young and aged rats, through the immunolocalization of the osteocalcin (OCN). Material and Methods: 96 female rats (Wistar) were used, being 48 rats with 3 months-old, divided in subgroups: Ovx, subjected the ovariectomy surgery and Sham, subjected to the same surgical procedure without the removal of the ovaries; and 48 female rats with 12 months-old, also divided in Ovx and Sham subgroups. After 30 days of Ovx or “sham” operation, all the animals received autogenous bone block graft in the jaw, harvested from the calvaria. The animals were euthanized in 7, 14 and 28 days postoperatively. The specimens were subjected to histometric and immunohistochemistry analysis. This was accomplished in a semi-quantitative way, to analyze the immunolocalizations against osteocalcin, evaluating the interference of the estrogen in this process. Results: In Young group, there was smaller osteocalcin immunolocalization in the Ovx subgroup in all the experimental periods, compared with the Sham subgroup and the intensity increased with elapsing of periods. This also happened in the Aged group, however with less intense immunolocalizations when compared with the Young Group. Conclusion: Within the limits of this study, the estrogen depletion causes a decrease of bone mineralization in young and aged rats and those effects were more negative in aged group.

## 181 Psychology association in the treatment of oral injuries: brief psychotherapy approach

AUGUSTO, V.O. (Viviane Oliveira Augusto vivianeoaugusto@gmail.com); ARAÚJO, K.C.V.; AZEVEDO, R.M.C.; MESSI, A.C.A.; ONOFRE, M.A.; SPOSTO, M.R.

Xerostomia and oral lichen planus (OLP) are diseases that can be associated with emotional disturbances. The aim of this study is to present two cases emphasizing the importance of brief psychotherapy as auxiliary treatment of these lesions. Case 1: A 69-year-old woman presented to Oral Medicine Service (OMS) with a chief complaint “pain in tongue and xerostomia”. When clinically examined, we observed hypossalivation. The patient showed had emotional disorders, then was referred to psychotherapy service. In psychological interview, the patient reported family conflicts and thus existential crisis. It was applied two psychological tests. The Lipp's Symptoms of Stress Inventory for Adults resulted in “Stress in Exhaustion's Phase with Predominance of Physical Symptoms” and the Beck's Depression, “Severe depression”. After several sessions of brief psychotherapy, the patient presented improvement of her emotional condiction and oral symptoms. Case 2: A 32 year-old woman, presented to the OMS complaining of “burning mouth and injury inside the mouth”. It was observed alteration suggestive of OLP. The patient also showed accented stress and anxiety, then was referred to the psychotherapy. In the interview she reported matrimonial conflicts with physical and moral aggression, affecting her emotional, causing oral symptoms. The Lipp's Symptoms of Stress Inventory for Adults resulted in “Stress in Exhaustion's Phase with Predominance of Physical Symptoms” and the Beck's Depression, “Severe depression”. The psychological treatment, although still in initial phase, brought benefits contributing to the improvement of emotional and oral symptoms. Based on these findings, we believe that the brief psychotherapy as support on the treatment of some oral lesions presents satisfactory results in both dental and psychological way.

## 182 Technique of alveolar bone expansion by osteotomy and use of chisels for placement of osseointegrated – case report

GOMES, W.D.S. (Weglis Dyanne de Souza Gomes weglis.daiane@hotmail.com); KAYATT, F.E.; SANTOS, P.L.; SANTIAGO-JR, J.F.; GARCIA-JR, I.R.

The treatment with osseointegrated implants is a technique of great predictability. The complexity of these rehabilitations is dependent mainly on the quality and amount of existing bone volume in the area that will receive the implant. Several advanced surgical techniques exist to establish bone volume of the maxillary and mandible. One of these strategies is the assisted rapid bone expansion by osteotomy and use of chisels. Initially, partial osteotomy of the vertical crestal is done with a saw. After, using a chisel, it is made an expansion of the bone plate. The implants are then inserted in the expanded alveolar bone. The objective of this study is to present through two reports of cases, maxillary alveolar bone expansion and subsequent installation of dental implants. It was observed, after the patients' final rehabilitation, that the technique fulfilled the expectations regarding the esthetic and functional rehabilitation, with a shorter treatment time and smaller risk for the patients. This technique results very satisfactory, allowing an esthetic and functional result and, presenting advantages, when the indication is pertinent, as clinical applicability of the technique (simple method), smaller surgical risk when compared to the grafts and, also smaller time of treatment.

## 183 Fracture resistance of endodontically treated teeth with different heights of crown ferrule restored with prefabricated carbon fiber post and composite resin core by intermittent loading

ROCHA, W.P. (Willian Pereira Rocha williampereirar@hotmail.com); PEREIRA, J.R.; OLIVEIRA, J.A.; ZOGHEIB, L.V.; VALLE, A.L.; GHIZONI, J.S.

This study evaluated the fracture resistance of endodontically treated teeth restored with prefabricated carbon fiber posts and varying quantities of coronal dentin. Sixty freshly extracted upper canines were randomly divided into groups of 10 teeth. The specimens were exposed to 250,000 cycles in a controlled chewing simulator. All intact specimens were subjected to a static load (N) in a universal testing machine at 45 degrees to the long-axis. Data were analyzed by one-way analysis of variance and Tukey test (á=.05). Significant differences (P<.001) were found among the mean fracture forces of the test groups (Positive Control group – 1022.82 N; 0 mm,1 mm, 2 mm, 3 mm groups and Negative Control group – 1008.22 N, 1292.52 N, 1289.19 N, 1255.38 N and 1582.11, respectively). These results suggested that the amount of coronal dentin did not significantly increase the fracture resistance of endodontically treated teeth restored with prefabricated carbon fiber post and composite resin core.

## 184 Methods for desinfection, esterilization and storage of teeth in human teeth Banks

FERNANDES, Y.R. (Ygor Renato Fernandes ygor.fernandes@usp.br); FREITAS, A.R.; SALES-PERES, A.; SALES-PERES, S.H.C.

The human teeth banks (HTB) have great importance in the reuse of dental organs, since they contribute to the obtaining, sterilization, storage and donation to academics and researchers. The aim of this study was to highlight the methods of disinfection, sterilization and storage used in most HTB. There is controversy in the literature about the methodology used in the sterilization and storage of human teeth, as it presents so diverse and without defined protocol and some of these methods may cause structural changes in the dental elements in order to harm its use in scientific researches. The most used agents in laboratory studies for disinfection/sterilization of dental organs at the HTB include formalin, glutaraldehyde, sodium hypochlorite, autoclave, thymol, sodium azide and chloramine. Others studies was reported the freezing of dental elements, Cryopreservation and use of distilled water for storage of teeth. It may be concluded that there is need to establish a protocol to work in the HTB on standardization of methods for disinfection, sterilization and storage of dental organs, thereby ensuring that the management of these teeth be done in a safe way and without compromising the activities to which they are intended.

## 185 Hypermobility as a risk factor for development of temporomandibular disorders

COSTA, Y.M. (Yuri Martins Costa yuri_martinsc@yahoo.com.br); SILVA, R.S.; MELO, M.P.; MIRANDA, J.T.

The excessive range of motion of a joint is referred as hypermobility. Regarding temporomandibular joints (TMJs), it can be considered hypermobile those in which the condyle pass over the crest of the eminence during maximum mouth opening. The hypermobility can be a consequence of systematic disorders like generalized joint hypermobility, but can also be related with local factors like steeps eminences or loose ligaments. It is supposed that hypermobile TMJ may produce intracapsular instability by mechanical overload. This situation, combined with trauma, parafunctions or emotional stress can lead to the development of temporomandibular disorders (TMD). However, the relationship between hypermobility and TMD has not been well established. The aim of this study is to analyze through a literature review if hypermobility of TMJ can be considered a risk factor to the development of TMD. The used articles were selected from PubMed-Medline data base. Most of the clinical studies show that there is a relation between hypermobility and signs and symptoms of TMD. Despite the lack of standardized methodology research and general consensus, this review endorses a cause-effect relationship between hypermobility and TMD.

## 186 Comparative study of the masticatory performance between patients with an without temporomandibular dysfunction

STRINI, P.J.S.A. (Paulinne Strini paulinne@netsite.com.br); MACHADO N.A.G.; GORRERI, M.C. FERNANDES-NETO, A.J.; NEVES, F.D.; MENDES, F.A.; BORGES, T.F.

Temporomandibular disorders (TMD) are a set of signs and symptoms that affect the stomatognathic system and adjacent structures, generating mainly a pain able to radiate to inter-related structures. The purpose of this study was to evaluate the severity of TMD using the Helkimo Clinical Index (HCI) and Masticatory Performance (MP) in patients with TMD (n = 9) and compare it to the control group (CG) (n = 15), both with natural dentition and bilateral posterior occlusion. The MP tests were performed with the food simulator “Optocal” in portions of 17 cubes with 5.6 mm side and the subjects instructed to chew it for 20 (C1) and 40 (C2) masticatory strokes, monitored by a single examiner. The material collected was placed in a set of eight coupled granulometric sieves in a decresing order of opening. Then sieving, material collection, drying and weighing of the contents of each sieve were performed. It was calculated the geometric mean diameter of the chewed particle (MGD), obtaining a percentage of the MP index. The obtained data were subjected to the parametric Student Test t for independent samples (p <0.05). According to CHI, n = 8 patients presented severe TMD and 1 patient showed light TMD. The results indicated statistically significant differences (p = 0000) both for GC (47.76 ± 8.75 in C1, C2 at 70.27 ± 3.37) and for the TMD group (24.06 ± 12.93 in C1, 49.32 ± 10.77 in C2). We conclude that patients with painful symptoms tend to have the chewing function impaired, requiring a treatment able to restore their physiological functions, improving their chewing ability and promoting their life quality.

## 187 Facial and dental injuries due to dog bite in a 15-month-old child with sequelae in permanent teeth: a case report

CORREIA, A.S.C (Adriana de Sales Cunha Correia dricunhacorreia@yahoo.com.br); CUNHA, R.F.; DELBEM, A.C.B.; NOVAIS, R.Z.

This article reports a longitudinal follow-up of a 15-month-old child with dental trauma resulting from an attack by a dog. The injury consisted of laceration of the facial tissues and loss of the upper central deciduous incisors, in addition to loss of bone tissue in the same area. A malformation of the crown of the right central permanent incisor and complete change of the shape of the left central permanent incisor were observed. The etiological factors of childhood injuries as well as the importance of dental emergency care are discussed and the 14-year clinical and radiographic follow up of the case is presented.

## 188 Pathogenic potential of the dental organs used in laboratory and scientific activities at dental schools

FREITAS, A.R. (Adriana Rodrigues de Freitas adrianafreitas@usp.br); FERNANDES, Y.R.; SALES-PERES, A.; SALES-PERES, S.H.C.

The reuse of teeth in academic, clinical and scientific activities contributes to the scientific progress of Dentistry, since it allows the development of new surgical techniques and restorative materials. However, the treatments prior to use of these organs often are not adequate. Important steps are overlooked as the disinfection or sterilization, proper storage, disposal, and where its reuse, the free and informed consent of their donors. The aim of this study was to report the necessary precautions for the use of dental organs from its acquisition until its use in various activities. The scientific evidence suggests that the dental organs can be considered as potential sources of infection when they are not properly undergo the process of sterilization or disinfection prior, and the Human Teeth Banks (HTB) are viable alternatives to secure the acquisition of these items. Among the methods used for the decontamination of dental elements was observed the use of solutions such as formalin, thymol, sodium hypochlorite, glutaraldehyde and sterilization by autoclaving. It is concluded that the creation and standardization of HTB are of great importance to help researchers and academics in the safe acquisition of dental organs and also respecting the ethical and legal aspects to use these elements.

## 189 Occurrence of periodontal pathogen microorganisms of red complex and *Aggregatibacter actinomycetemcomitans* in babies aged 6 and 12 months and their respective mothers

ANDREOTTI, A.M. (Agda Marobo Andreotti agda.ani@hotmail.com); BIANCO, K.G.; GAETTI-JARDIM JR, E.

The purpose of this study was assessed the presence of periodontal pathogen microorganisms in children aged 6 to 12 months, assisted by an educational-preventive program, its correlation with the oral microbiota and periodontal conditions of their mothers, and family socioeconomic and cultural aspects. After the evaluate socio-economic and cultural family through validated form, was held a clinical examination of mothers and children selected and their respective collections of saliva and subgingival biofilm. The saliva was collected at 6 months when edentulism, and after the initial eruption, were subgingival biofilm and saliva in 12-month period. The clinical specimens were transferred to tubes containing transport means and VMGA III cryotubes with Milli Q water. Specimens transported in VMGA III suffered dilutions and were inoculated on agar TSBV for the isolation of *Aggregatibacter (Actinobacillus) actinomycetemcomitans*, whose isolates were subjected to assess the potential of leucotoxigenicity using PCR. The presence and participation of periodontal pathogen microorganisms in the oral microbiota of mothers and children were assessed using conventional PCR and Real-Time PCR. Statistical analysis determined by means of multivariate logistic regression, the existence of correlations between the parameters studied, while, through the statistics and the Cochran Mantel-Haenszel (calculation of odds-ratio) and chi-square of Pearson were performed analysis of risk.

## 190 Analysis of radiographic magnification of osseointegrated implants in periapical radiographs: a preliminary study

AGUILERA, GS (Gustavo Souza Aguilera guto.aguilera@gmail.com); DUARTE, B.G.; GONÇALES, E.S.; CAPELOZZA, A.L.A.; DUARTE, M.A.H.

Osseointegrated implants have been used in the total and partial rehabilitation of edentulous individuals. Modern implants are cylindrical or conical titanium screws that are placed inside the alveolar bone, replacing the root(s) of lost teeth. It is necessary, therefore, appropriate amount of bone tissue and precise surgical planning need to install them. During the planning, measurement of bone availability appears to be fundamental and can be made using radiographs periapical, panoramic or in CT scan. The periapical radiograph is indicated in cases where we want to see details of the local bone anatomy, but does not allow precise measurements, since, even when performed with radiographic advices, magnification of the image cam occur. This fact seems not be consensus in the literature, since there are studies that suggest precise measurements with the periapical radiographic technique. The objective of this study was to verify the occurrence of magnification in images obtained by means of periapical radiographs, which are obtained from an dental implant placed in an artificial jawbone, with standardized time of exposure, processing and positioning. After obtaining the radiographic images of the implant, it was direct measured by millimeter ruler and the data were tabulated and analyzed statistically. The results showed magnification of the dental implant image.

## 191 Analysis of behavioral characteristics associated with dental caries in children of Bauru, São Paulo, Brazil

MELO, A.O. (Alcides Oliveira de Melo aomelo2@hotmail.com); CARVALHO, C.A.P.; MARSICANO, J.A.; CARVALHO, F.S.; SALES-PERES, A.; SALES-PERES, S.H.C.

The declining of dental caries prevalence has aroused interest among researchers to investigate not only clinical, but also socioeconomic and behavioral characteristics associated with the development of disease. The aim of this study was to analyze behavioral variables associated with dental caries in deciduous dentition of children from Bauru, SP. Three hundred and fifteen children with 4 to 6 years old, from 6 schools, were examined by a calibrated dentist. The dmft index was used in the survey, according to WHO criteria. The children's parents answered a questionnaire about the person responsible for brushing, frequency of brushing, ingestion of dentifrice, use of fluoride rinses and type of water ingested by child. The association between dental caries and behavioral factors was made by chi-square test. The mean dmft index was 1.07 and 69.21% of examined children were caries-free. In 73.44% of caries-free children, parents were responsible for brushing and about 70.00% of them brushed their teeth 3 or more times a day. Furthermore, children who had fluoride rinses, 72.00% were caries-free. It was observed higher proportion of children who ingested public water supply with caries in relation to those who ingested mineral water or water from other sources. However, the behavioral variables examined did not show statistically significant association with dental caries (p> 0.05). In the sample studied, the prevalence and severity of dental caries were considered low and behavioral factors were not considered significant to the development of disease. Further studies should be conducted to investigate the possible influence of these factors for the development of dental caries.

## 192 Percentage of sealer adaptation in roots obturated with the Thermafil and RealSeal-1 obturation techniques

DEL CARPIO, A (Aldo del Carpio Perochena aldo_delcp@hotmail.com); BRAMANTE, C.M.; BERNARDELI, N.; MORAES, I.G.; GARCIA, R.B.; ZAPATA, R.O.

The aim of this study was to evaluate the percentage of sealer penetration in root canals filled with the Thermafil or RealSeal1systems analyzed by Confocal Laser Scanning Microscopy (CLSM). Twenty canals in 10 mesial roots of mandibular molars were cleaned and shaped using ProTaper and ProFile instruments to a size 35.04 taper at the apex and filled using RealSeal1 or Thermafil systems on mesial canal of each root. Horizontal sections were made at the 3 and 5 mm level from the apex and the percentage of sealer penetration in the root canal walls was analyzed using a CLSM. Thin layers of sealer (2-30μm) and sealer tags into dentinal tubules were found in the root canal walls in a high percentage using both techniques at both evaluated levels with no statistical differences between the techniques, T-student test (p>0.05). In conclusion, the percentage of sealer penetration in the root canal walls was similar using both thermoplastic carriers based systems.

## 193 Denture-induced stomatitis

CANDIDO, A.G. (Aline Gabriela Candido alinecandido@hotmail.com); GONÇALVES, E.A.L.; SILVA, P.V.R.; CANDIDO, G.C.; COSTA, M.C.M.; COSTA, R.R.

Denture-induced stomatitis refers to injuries commonly observed in oral mucosa under the base of those prosthesis used for long time. Its development occurs in the region of maxilla, mandible and palate, as an edema or erythema and it can be located or widespread. The lesion may be present as a hyperplasia, like inflammatory fibrous hyperplasia and inflammatory papillary hyperplasia. It may also be associated with candidosis, the erythematous candidosis being the most frequent clinical form. This work aims to perform a literature review about denture-induced stomatitis, through the approach of its various forms of occurrence, etiology, clinical and microscopic aspects, and its treatment. It was observed that in every analyzed manifestation of denture-induced stomatitis, the etiologic factor converged to the daily use of the prosthesis for a period longer than 24 hours, which were ill-fitted, and sometimes associated with poor oral hygiene. Therefore, it was not verified disparities in the treatment, which prescribes interruption of denture use, cleansing and administration of antifungal agents when Candida albicans is present. In this context, it can be concluded that this alteration in the mucosa appears as a manifestation of bad habits in association with the ill-fitted dentures, and its treatment is usually surgical.

## 194 Mollicutes in the oral cavity: distribution in eight native ethnic groups from Brazil and from a Nigerian community

GERALDES, A.M. (Aline Martucci Geraldes aline_geraldes@hotmail.com); GAETTI-JARDIM, E.C.; NETO, R.L.; TIMENETSKY, J.; OBIAGERI, N.F. FAPESP 08/53297-2.

The class Mollicutes is associated with infectious and inflammatory diseases in the respiratory, oral, genital, gastrointestinal mucosa, ands they may collaborate to the establishment of arthritis and other chronic diseases. Some studies have shown its participation in the etiology of periodontitis. However, most of studies were performed in North America and Europe, and its distribution in native South Americans and Subsaharian Africans is not known. Then, this study evaluated the occurrence of Mollicutes in the oral cavities of 100 natives of Umutina, Paresi, Bororo, Bakairi, Kayabi, Irantxe, Nambikwara and Terena nations from Central Highlands in Brazil, and 40 Nigerians from Igbo tribe, Lagos, Nigeria. Initially, periodontal and oral health conditions of the patients were examined and recorded. Then, resting saliva, supragingival biofilm, subgingival biofilm and oral mucosa were sampled. The detection of Mollicutes was evaluated by PCR. Forty-eight native Brazilians were gingivitis patients, 38 periodontitis patients and 14 periodontally healthy subjects, while 20 Nigerians were periodontally healthy and 20 periodontitis patients. All samples positive for Mollicutes were also tested for Mycoplasma sp., Acholeplasma sp. and Ureaplasma sp. and for the most relevant species of these genera. Subgingival biofilm was the most important oral habitat of these microorganisms, which were detected from 28.57% healthy native Brazilians, 43.75% native Brazilians with gingivitis and from 50% natives with periodontitis, as well as from 40% healthy and 80% periodontitis Nigerians. The most commonly detected species were M. orale, M. salivarium, M. hominis and U. urealyticum, and 30% Mollicutes positive samples could not be properly identified to the species level. In Nigerian samples, besides these species, M. pneumoniae and Ureaplasma sp. were also frequently detected. It was not observed any statistically significant association between periodontal status of native Brazilians or Nigerians and the presence of these microorganisms in the oral cavity.

## 195 Short and long-term effect of rapid maxillary expansion on nasal patency: a case report

IOST, A.V (Aline Victor Iost aline.iost@usp.br); SAMPAIO-TEIXEIRA, A.C.M.; YAMASHITA, R.P.; FUKUSHIRO, A.P.; TRINDADE, I.E.K.

Rapid maxillary expansion (REM) is an orthodontic procedure designed for the correction of maxillary atresia, which has the potential effect of changing nasal patency, usually impaired in individuals with cleft lip and palate. The purpose of this report is to present a clinical case of REM performed in a patient with cleft lip and palate and analyze its effect on the internal nasal dimensions in the short and long-term, by means of acoustic rhinometry. The technique allows the measurement of sectional areas of different nasal segments and thus the assessment of nasal geometry. In a patient with repaired unilateral cleft lip and palate, 15 years of age, male, minimum cross-sectional area of the nasal cavity (MCSA) was determined before expander installation and 2 months and 10 months after the active phase of expansion, using an Eccovision Acoustic Rhinometer (HOOD Laboratories). Measures were also taken after nasal decongestion in order to eliminate mucosal interference. For analysis, areas of the left and right cavities were added. MCSA corresponded to 0.70cm^2^ before expansion, 1.24cm^2^ after 2 months and 0.71cm^2^ after 10 months of the orthodontic procedure. Areas after nasal decongestion corresponded to 1.06cm^2^, 1.16cm^2^ and 1.13cm^2^, respectively. Results show that REM led to a short and long-term skeletal increase of the nasal airway. However, data observed without nasal decongestion points to an eventual acommodation of the mucosa to the new anatomic conditions causing nasal patency to return to the baseline state in the long-term. A study is currently being conducted at our laboratory to confirm these findings on a larger sample size.

## 196 Effect of raloxifene and estrogen on bone metabolism of rats

OLIVEIRA, C.B. (Claudiel Batista de Oliveira claudieloliveira@gmail.com); ALMEIDA, L.R.B.; NAKAMUNE, A.C.M.S.; DORNELLES, R.C.M.

The total amount of bone mass in women is directly related to plasmatic concentration of estrogen (E2). With increasing age, the production and secretion of less E2 trigger loss of bone associated with menopause (Bone 19:185 S, 1996). The Raloxifene hydrochloride has selective agonist or antagonist activity on the tissues that respond to estrogen. The objective of this study was to analyze the process of alveolar repair and the plasmatic concentrations of phosphorus, calcium and estradiol in rats in different experimental situations. Thirty two Wistar rats, and 8 intact rats (2 m) with regular estrous cycle and 24 rats (18 m) ovariectomized (OVX) one year ago were used. During 60 days, the OVX rats received pellets containing estrogen (400 ug/17b-estradiol) or corn oil, these pellets exchanged every 30 days. Other group of OVX rats received raloxifene (1mg/Kg/d) by gavage. Under general anesthesia, the animals were subjected to extraction (28 days before euthanasia) of the upper right incisors for further analysis of the of alveolar repair process. At the end of the treatment period, animals were anesthetized (xylazine - 4 mg / kg, ketamine - 40 mg / kg) for blood collection and the jaws were removed, decalcified and dried for the preparation of microscopic slides. The histometric analysis revealed bone formation of 51.12% (intact), 30.99% (OVX/oil), 50.28% (OVX/E2) and 36.93% (OVX / RLX) in the middle third of the animals studied. The plasma analysis showed significantly higher concentration of phosphorus in the group of OVX rats pre-treated with raloxifene and the concentration of calcium was lower in animals of group OVX/E2. In ovariectomized animals that received hormone replacement, the plasma concentration of E2 was significantly higher than that of the other groups. The 1-year results suggest potent activity of E2 and subtle of raloxifene on bone formation in OVX animals.

## 197 Papillary squamous cell carcinoma: a case report

PAULA, A.R. (Alyne Reis de Paula alyninha_reis@hotmail.com); ROSA, R.R.; BARBOSA DE PAULO, L.F.; SARGENTI-NETO, S.; CARDOSO, S.V.; LOYOLA, A.M.

Papillary squamous cell carcinoma (PSCC) is a rare variant of squamous cell carcinoma (SSC) that can occur *in situ* or as an invasive tumor. There have been only a few cases of PSCC reported in the oral cavity, and the most important differential diagnosis in this region is with verrucous carcinoma (VC). Most cases of PSCC are solitary lesions or part of a cluster of papillary lesions, some of which have evolved from preexisting papillomas and all of which have a fibrovascular core. These tumors are most commonly found in the oropharynx, hypopharynx, larynx, or sinonasal tract. This subtype of SCC is most commonly found in adults in their 70s and tends to affect men more frequently than women. The differential diagnosis should be made with other papillary neoplasms such as papilloma and VC. Surgery is the treatment of choice and adjunctive therapy may be used. The majority of PSCCs are at a low clinical stage (T1). Some authors report a better prognosis for papillary SCC than for conventional SCC when matched for T stage. We present a case of PSCC arisen in a 32-year-old woman that was referred to the oral diagnosis service with the chief complaint of an asymptomatic growing mass on the lateral border of the tongue. The mass had been there for nearly 1 month and no smoking or drinking vicious habit was reported. The medical history revealed diabetes type 2 and hypertension in treatment for cardiologist. The oral examination evidenced an exophytic mass, 2.5 cm in diameter, with a papillary aspect and white patches on the surface. A clinical diagnose hypothesis was papilloma and a incisional biopsy was performed. The histopathological analysis evidenced a PSCC. In view of this fact, the patient was referred to a head and neck surgeon for treatment.

## 198 Actinic cheilitis with moderate dysplasia

MESSI, A.C.A. (Amanda Cabbau do Amaral Messi amandamessi@yahoo.com.br); AZEVEDO, R.M.C.; CHAVEZ, G.; AUGUSTO, V.O.; ANDRADE,C.R.; MASSUCATO, E.M.S.

The actinic cheilitis (AC) is a potentially malignant epithelial lesion of the oral mucosa that attack the inferior lip. It is characterized by atrophic areas and lost of desing's lip. Can be associated with white plaques that cannot be scraped (leukoplakia), ulceration or crust. Prevalence is higher in white people and the main cause is exposure to the solar radiation without protection. The purpose of this work is to present a clinical case of a white man, 62 years old, carrier of diabetes and hypertension. The patient was smoker and worked in farming during 40 years. When clinically examined, painless inferior lip's atrophy associated with non-scraping white plaque could be observed. Our diagnostic hypothesis was actinic cheilitis with leukopakia and erosive areas. Before the incisional biopsy, a test with toluidine blue was performed, with retention in some areas. The histopathological examination revealed actinic cheilitis with moderate dysplasia. The pacient was referred to surgery to realize vermillectomy. And to the psychotherapy's service to receive emotional support and to be helpeld to stop smooking. We conclude that the early diagnosis is the main factor to the proginosis of potentially malignant epithelial lesion and that the psychologist's perfomance is important to the control of stress and complicantion's prevention.

## 199 Biomechanical explanations for failures in indirect restorative treatment

KASUYA, A.V.B (Amanda Vessoni Barbosa Kasuya amandinha_vk@hotmail.com); FAVARÃO, I.N.; SOARES, C.J.; URSI, W.; FONSECA, R.B.

The choice of post material is important for the preservation of dental structure and reduction of possible future failures. At the same time the biomechanical properties of restorative dental materials and its capacity of interaction with dental structures must be known to prevent damages. The aim of this work is to present errors in indirect restorative treatments and the possible biomechanical factors involved in the selection of materials and post types. Prefabricated and cast metal posts had an ample use during XX century; however their high modulus of elasticity provide high stress concentration, increasing risk for fractures, in addition to the lack of adhesive interaction with the dental structure. Glass fiber posts present lower fracture resistance, modulus of elasticity close to that of dentin and bonding capacity, resulting in stress distribution and allowing for economy of tooth structure. Ceramic restorations tend to fracture without compromising dental structure and the opposite is truth for those made up by Laboratorial Composite Resin; this can be explained by materials properties since both have adhesive capacity. The knowledge of biomechanical materials and dental structure properties is essential to prevent failures during restorative treatment. Full knowledge of the materials, techniques and their interaction with dental structure are needed for rehabilitation of function, esthetics and form of teeth.

## 200 Dentistry as a conclusive science in human identification – a case report

BARBIERI, A.A. (Ana Amélia Barbieri anameliabarbieri@gmail.com); SCORALICK, R.A.; MORAES, Z.M.; NARESSI, S.C.M.; DARUGE JÚNIOR, E.

The oral radiographic exam, besides being an important tool for diagnosis, keeps record of an individual dental characteristic, carrying a number of details that individualize a person, being important to human identification cases. Human identification consists in determining an individual's identity by comparison of his/her sinaletic elements registered in the present with the other ones previously recorded. Several viable methods to human identification have been developed, but this one, used by dentistry, deserves a particular attention for being simple and practical. The objective of this study was to demonstrate that making and keeping dental records correctly are essential pre-requirements not only to the professional defense in court, but also to cooperate with the individual identification if it is necessary. This study reports a carbonized individual identification case in which a comparison between a panoramic oral radiograph previously taken with the periapical radiographs taken after death was performed. This procedure presented twelve common points, leading to the body positive identification. It was concluded that the identification method by radiographic comparison, used for this human identification case, was efficient, presented a large safety margin and a low cost, demanding also a few equipments.

## 201 Biochemical and bone densitometry of ovariectomizeds rats treated with raloxifen and fluoride water

ROSSI, A.C. (Ana Cláudia Rossi anacrossi1@hotmail.com); IGREJA, B.B.; CARVALHO, A.A.F.; DORNELLES, R.C.M.

The reduction of the plasmatic concentration of estrogen modifies the function of systems and results in significant loss of bone mass. The Raloxifen (RLX) mimics the beneficial effect of hormonal replacement compensating for the disadvantages of this therapy, being able to prevent bone resorption and reduce the risk of fractures. Studies point to the importance of the sodium fluoride in the composition and incorporation to present calcium in the bone, conferring to it higher resistance. The purpose of this work was to verify, through bone densitometry and analysis of plasmatic concentrations of calcium and phosphorus, if the association of fluoride and Raloxifen improve the bone quality of ovariectomized rats. Ten days after the ovariectomy, Wistar rats (6 m) received Raloxifen, through gavage, during 6 months. During this period, they received distilled water and distilled water + NaF/20 or 40 ppm. After 180 days, the animals had been anesthetized for blood collection and sacrificed by anesthetic overdose to remove femurs for evaluation of the maximum and minimum bone densities using Digora digital system in three regions (head, throcanteric fossa and throcanter) from proximal epiphysis. The densitometry evaluation not evidenced alteration in the radiopacity/radiolucency in the three regions between the groups. The plasmatic results demonstrated that it does not have significant alteration in the calcium concentration between the groups. The plasmatic concentration of phosphorus was significantly lower in the animals of group RLX/20 ppm of NaF. The results suggest that the combination of anabolic and antiresorptive therapy changes in the metabolism of phosphorus, but did not alter the bone density of the femurs of experimental animals.

## 202 The evolution and applications of xylitol in dentistry

FLORENTINO, A.C.A. (Ana Carolina Andrade Florentino anacarolinaflorentino@gmail.com); AZEVEDO, M.R.

Xylitol is a kind of sugar's substitute, usually obtained from vegetables and biotechnology, and has been the object of studies on its obtaining from Biotechnology engineering, food industries, dentistry and medicine in the treatment and prevention of diseases. It is associated with better health quality because it provides a slightly sweet taste with low-carbohydrate content, with 40% less calories than refined sugar. In addition, its antibacterial properties allow its smart consumption, by normal digestive and parenteral vias, usually in patients with sepsis risk. In dentistry, its cariostatic and anti-cariogenic effects allow the its incorporation to gums, lozenges, dentifrices with or without fluoride, mouthwashes and varnishes, which has recently been investigated for long-term applications. Studies on dentifrices prepared with xylitol show that the periodontal tissues are benefited by plaque reduction in patients with orthodontic appliances acute periodontitis. In the cases reorted in dental, bleeding index regresses. Besides, it stimulates the salivary gland combating the xerostomia and fungi involved in angular cheilitis in debilitated patients. Antimicrobial properties give to this substance a high potential for the management of diseases of the otolaryngologic tract, been recommended in sprays and syrups to the prevention of acute otitis media, allergic rhinitis crises and rhinosinusitis episodes. This study presents a literature review about the evolution in obtaining xylitol, main derived products, the prevention of oral diseases, the medicinal properties, and the indications and contraindications for human use.

## 203 Qualitative and quantitative tools applied to analyze adhesion to dental structure

HIPÓLITO, A.C. (Ana Carolina Hipólito carol_hypolito@hotmail.com); CALABRIA, M.P.; FRANCISCONI, L.F.; PEREIRA, J.C.; RIOS, D.; WANG, L.

Hybrid layer is a mechanical interlocking structure established in dental substrates previously conditioned and treated with a resin adhesive. Besides the great expectative of adhesive treatment, it is not uncommon to observe clinical failures that occur, mainly in this layer. In attempt to a better comprehension of the mechanism involved in its establishment and in its performance under service, adequate methods should be applied to study the hybrid layer. This presentation aims to explore qualitative and quantitative laboratory methods, highlighting their advantageous and limitations features. Qualitative resources include scanning electron microscopy, transmission electron microscopy and microscopy of confocal laser. Regarding the quantitative methods, mechanical tests as shear and microtensile resistances are the commonest tools. In final instance, clinical assessment thought time attest the behavior of the bonded restorations. All of the presented methods are far from ideal to the total understanding of hybrid layer' characteristics, however they can be associated to reach relevant results that are helpful to propose new directions.

## 204 Perymolisis provoked by hiatus hernia: a case report

ROBERTO, A.F.B. (Ana Flávia Betoni Roberto anaf_betoni@yahoo.com.br); OLIVEIRA, G.C.; PEGORARO, C.N.

The aim of this paper is to report the clinical case of a 52 year-old male patient, who sought for dental care at the Restorative Dentistry Clinic of Sacred Heart University, in Bauru, SP, complaining about extreme and generalized tooth sensitivity, difficulties in biting and feeding and dissatisfaction with the appearance of his teeth. By performing the clinical exam it was verified the existence of typical dental erosion lesions from intrinsic causes, the perymolisis, which are an extensive loss of enamel and dentin in various teeth, in special in the palatal face of anterior teeth and palatal and occlusal faces in the posterior teeth. The lesions cause chamfered appearance of the incisal board in anterior teeth, loss of occlusal outline of posterior teeth and raised amalgam restorations. During the anamnesis, the patient told that he had a hiatus hernia for many years. This gastroesophageal disturbance provoked nausea and daily reflux which, for many times, reached his throat and caused cough and vomiting sensations. This is caused mainly due to an alteration in the functioning of the inferior esophagus sphincter muscle which controls the passage of food from the esophagus to the stomach and hinders the return of acids and the stomach content to the mouth cavity. In his initial appointment the patient was warned about the etiology of dental lesions, which provoked extensive dentin sensitivity and discomfort on chewing. He was orientated to maintain the medical treatment for his hernia and the restorative treatment started in the areas affected by the erosions.

## 205 The golden proportion in dentistry: a clinical case report

GARRIDO, A.M.R (Ana Miriam Garrido anamiriam@usp.br); BRAVO, D.M.C.; MONDELLI, R.F.L.; ISHIKIRIAMA, S.K.

The golden proportion is defined as a special relationship observed in nature and instinctively prized like beautiful. It is mathematically represents for the Greek word (phi), and it is rounded value is 1.618. In dentistry, it is uses in lopsided initial evaluation and in planning esthetics treatments, obtaining as results both natural and artistic beauty. The aim of this work was to demonstrate how the golden proportion can be applied in dentistry to reestablish harmonics, functional and esthetic dimensions. The patient, a 45 year-old male, C.N.Q, came to the Bauru School of Dentistry unhappy with the appearance of his teeth. After clinical and radiographic examination defective restorations in the teeth 21 and 22 were diagnosed, presenting multiple diastemas and dental attrition causing alterations in anterior and canine guides. Plaster models were obtained and a diagnostic waxing was performed, based on the golden proportion theory, using a caliper, golden mean gauge and grids. The treatment plan included: oclusal adjustment, distalization of teeth 12 and 22 with a rubber band, gingival conditioning with composite increments, obtaining a silicone matrix to be used as a guide to facilitate the restorations technique, closure of the diastemas and increasing of the incisal borders of tooth 13 to 24 thus reestablishing the canine guide and the anterior guide. Finally, finishing and polishing were done. It was proven that golden proportion can be used to obtain esthetic improvements, reaching the expectations of both patient and dentist.

## 206 Temporomandibular disorders treatment based on scientific evidence

CORRÊA, A.S.M (Ana Sílvia Da Mota Corrêa asmcorrea@yahoo.com.br); ALENCAR, E.N.; CUNHA, C.O.; PINTO, L.M.S.; CONTI, P.C.R.

Recently, the incorporation of evidence-based dental concepts has been defined by the ADA (American Dental Association) as an approach to oral health care that requires the judicious integration of systematic assessments of clinically relevant scientific evidence, relating to the patient's oral and medical conditions and history, with the dentist's clinical expertise and the patient's treatment needs and preferences. This concept requires that to be accepted as a study of great value to clinical practice a research needs to follow specific criteria and rigid methodology. Therefore, this literature review was based on studies with the highest level of scientific evidence as randomized controlled trials (RCTS) and systematic reviews including meta-analysis. A total of thirty-seven papers were held to assess the presence of evidence in manual therapy, pharmacotherapy, occlusal therapy, needling of trigger points, orthodontics, surgery and cognitive therapy used to control temporomandibular disorders (TMD). According to this review, evidence was found only for therapies as low level laser therapy and mid-laser, and for the analgesic effect of tricyclic antidepressants. The use of stabilization splints showed no efficacy in the treatment of TMD, but seems to be benefic in clinical practice. The occlusal adjustment is not supported by evidence and should be contraindicated. It is suggested that surgical therapy should only be indicated for refractory patients to reversible therapy. Cognitive therapy appears to be effective in the treatment of TMD. In general, more high-quality studies are needed to evaluate the effectiveness of these therapies in the treatment of TMD.

## 207 Dental care to autistic patients

DORETTO, A. (Anaita Doretto Eugênio anaitadoretto@hotmail.com); SANTOS, M.J.P.; MENEZES, T.E.C.; AGUIAR, S.M.H.C.A.

Autism is a development disorder with brain alteration that in certain cases affects the individual's capacity of establishing relationships. This presentation has the goal to evaluate the incidence of autism among the patients assisted at CAOE (Center of Dental care to Handicapped, an Auxiliary Unit of the Dental School of Araçatuba, SP), presenting the methods used in dental care performed to these patients, their dental characteristics, needs and the reactions during the treatment. The records of 78 patients diagnosed with autism and assisted at CAOE were evaluated. These were selected through a survey carried out based on data, files of neurological diagnosis of the Center, using database (file DBF), considering the period from 1985 to 2004. From the 4 situations of dental care, it was observed that 24 patients went assisted through adaptation, 47 through physical restraint, 32 with sedation and 29 with general anesthesia. It was noticed the lack of oral hygiene raised upon the difficulty of dealing with autistic patients, classified as severe and aggressive, consequently presenting, in certain cases, high index of poor oral health and periodontal problems. In conclusion, this information is very important to health professionals, especially to those who work with Dental Care for Patients with Special Needs and, the physical restraint was the most used resource in assistance to these patients during their dental treatment.

## 208 Fracture resistance of weakened roots restored with composite esin and glass fiber post

FELIPE, A.F. (Anderson Fernandes Felipe andsofelipe@hotmail.com); PEREIRA, J.R.; ZOGHEIB, L.V.; OLIVEIRA, J.A.; VALLE, A.L.; GHIZONI, J.S.

This study evaluated the fracture resistance of weakened roots restored with glass fiber posts, composite resin cores and complete metal crowns. Thirty maxillary canines were randomly divided into 3 groups of 10 teeth each: teeth without weakened roots (control); teeth with partially weakened roots (PWR) and teeth with and largely weakened roots (LWR). The control group was restored with glass fiber posts and a composite resin core. Teeth in the PWR and LWR groups were flared internally to standardized dimensions in order to simulate root weakness. Thereafter, the roots were partially filled with composite resin and restored in the same way as in the control group. The specimens were exposed to 250,000 cycles in a controlled chewing simulator. All intact specimens were subjected to a static load (N) in a universal testing machine at 45 degrees to the long axis of the tooth until failure. Data were analyzed by one-way ANOVA and Dunnett's test for multiple comparisons (p=0.05). There were statistically significant difference differences (p<0.01) among the groups (control group = 566.73 N; PWR = 409.64 N; and LWR = 410.91 N), with significantly higher fracture strength for the control group. There was no statistically significant difference (p>0.05) between the weakened groups. The results of this study showed that thicker root dentin walls significantly increase the fracture resistance of endodontically treated teeth.

## 209 Contribution of the fluoridation of public water supply to the decline of dental caries

SALES-PERES, A.C. (André de Carvalho Sales Peres andre.carvalho.peres@usp.br); TAGLIAFERRO, E.P.; SALES-PERES, S.H.C.; AMBROSANO, G.M.; PEREIRA, A.C.; BASTOS, J.R.M.

The aim of this study was to compare a period of fluoridation of public water supply and identify the decline in the prevalence of dental caries in adolescents in the last 30 years. Probabilistic samples were obtained from adolescents with 12 years old, in Bauru-SP, which were evaluated by calibrated examiners, in 1976 (n=261) and 2006 (n=334). For data analysis, the DMFT, SiC Index and percentage of caries-free children were determined. The means (SD) for DMFT were 9.89(3.96) and 0.90(1.53), which the SiC Index were 14.34 to 2.63, in 1976 and 2006, respectively. The number of caries-free children (DMFT=0) ranged from 0.4% in 1976 to 63.8% in 2006. The fluoridation of water supply with other preventive methods available in Bauru city contributed to the occurrence of this great decline.

## 210 Orthodontic traction of impacted tooth associated of odontogenic tumor

SCATOLON, A.L. (André Luis Scatolon andre.scatolon@usp.br); CARDOSO, C.L.; CAPELOZZA, A.L.A.; FERREIRA JÚNIOR, O.; CONSOLARO, A.

A 15-year-old male was directed for surgical evaluation of an impacted upper right second molar. Clinically, it presented a discrete swelling in the posterior region of right maxilla. Radiographically the presence of a well circumscribed radiopaque lesion, with approximately 8mm of diameter was observed, right coronally the impacted tooth. A cone beam CT scan was performed to better evaluate the relation of the lesion with the involved tooth. The presumptive diagnosis was of odontoma compound or complex type. The lesion was removed, the impacted tooth was exposed, and orthodontic traction was done. The microscopic characteristics revealed disclosed disorganized proliferation of odontogenic cells with enamel synthesis, dentin and pulpal tissue in the most internal portions of dentin. The odontogenic tissues presented disorganized complex soft and hard odontogenic tissue mass. The final diagnosis was complex odontoma. Six months later, the tooth is correctly positioned with no lesion recurrence. This case emphasizes the importance of maintenance the impacted teeth associate the benign odontogenic tumors.

## 211 The early treatment of maxillomandibular dimensional changes in the anterior-posterior direction

KIMURA, A.Y. (André Yukio Kimura ayykimura@hotmail.com); ALMEIDA, M.R.; ALMEIDA, R.R.; OLTRAMARI-NAVARRO, P.V.P.; NAVARRO, R.L.; CONTI, A.C.C.F.

The anterior crossbite is defined as an incorrect relationship between the incisors, clinically observed as a negative overjet when the subject is in a maximum intercuspation relationship. This problem is normally related to a prognathism of the mandible, which is a characteristic of Angle Class III malocclusion. It can also be caused by: a deciduous dentition trauma, premature loss of deciduous teeth, inadequate arch length, maxillary hypodevelopment, mandibular hyperdevelopment or a combination of both. The differential diagnostic is always indispensable to detect a skeletal or a dental involvement. The prevalence of anterior crossbite is a health concern because it occurs in 7.6% of mixed dentition population once this malocclusion is not self-correcting. The Angle Class III malocclusion can be observed in many cases with a skeletal severity which does not exclude the possibility of surgical treatment. However, it can also be treated as early as possible. The advantage of this protocol includes a decrease in the number of cases that should be treated with a surgical approach. The present case has as an objective to show the early orthodontic treatment of the anterior crossbite promoting a normal maxilla's growth.

## 212 Bone expansion: an alternative to atrophic alveolar bone reabilitation

SOUZA, A.P. (Andréia Pereira de Souza andreia.xlvi@usp.br); GREGHI, S.L.A.; REZENDE, M.L.R.; SANT'ANA, A.C.P.; FIGUEIRA, E.A.; RESENDE, D.R.B.

Currently, the osseointegration advance has contributed to the increase of implant use in the oral rehabilitation of partially or completely edentulous patients. However, some patients have anatomic defects in alveolar bone that prevent or difficult implant installation in a favorable position. The etiology of these anatomic defects is related, mainly, with tumor resetion, congenital deformities or tooth losses by trauma, periodontal disease, periapical disease, root fracture and iatrogenic factors, which, invariably result in 60% of bone volume resorption in the first three years. Aiming to return to the patient to an anatomic condition that enables an oral rehabilitation, several surgical procedures have been developed and studied, from extraction socket preservation up to alveolar bone reconstruction, by surgical techniques to correct alveolar bone thickness and height. However, there are several techniques to restore or increase bone structure, such as particulate or onlay bone graft techniques, guided bone regeneration, distraction osteogenesis, sinus lifting, among others. Most of techniques results in implants installation in a second surgical intervention, increasing the number of surgeries and, consequently, the postoperative risks and morbidity. In some cases, the bone expander utilization can be an alternative to implant installation in atrophic alveolar bones. The implant installation technique with expanders benefits from the bone elasticity modulus, especially in the maxilla, to allow the bone expansion, creating a fast alternative and with low morbidity to implants placement. This study discusses the indications, technique and different types of expanders, with the illustration of clinical cases.

## 213 A protocol for the treatment of the maxillary central incisor impaction

ROSSETTI, A.P. (Andressa Pablos Rossetti andressa_pablos@hotmail.com); CONTI, A.C.C.F.; ALMEIDA, M.R.; OLTRAMARI-NAVARRO, P.V.P.; ALMEIDA, R.R.; NAVARRO, R.L.

During the development of the occlusion, some anomalies can be expected. Dental impaction and ectopic irruption are the most common problems in this regard, sometimes caused by the presence of a supernumerary tooth. The prevalence of the impaction of the maxillary central incisor affects 1% of the general population. The presence of mesiodens, and subsequent alteration in the normal path of irruption of the tooth is considered one of the reasons for such abnormality. Based on that, it will be discussed a protocol for the treatment of the impaction of a maxillary central incisor, in a female patient with permanent dentition. Clinical and radiographic evaluations were performed and the presence of a supernumerary tooth was detected, blocking the maxillary incisor normal irruption. A rapid maxillary expansion was indicated to provide arch space and to correct the posterior crossbite. After that, a surgical procedure was performed to remove the supernumerary teeth and to prepare the maxillary incisor for traction. Orthodontic mechanics were applied to correct the tooth alignment and, for esthetic reasons, a dental cosmetic procedure was also executed. An early diagnosis, done in the mixed dentition, should be emphasized in order to allow a spontaneous irruption of the impacted teeth, which could improve the treatment prognosis.

## 214 Dental caries profile in Monte Negro, State of Rondonia, Brazil, 2008

XAVIER, A. (Angela Xavier angelax@usp.br); BASTOS, R.S.; BASTOS, J.R.M.; SILVA, R.P.R.; CALDANA, M.L.; LAURIS, J.R.P.

This epidemiological survey has been done to assess the dental caries profile in the town of Monte Negro and its relation to the north region and all Brazil. The groups examined randomly were 5, 12, 15 to 19, 35 to 44 and 65 to 74 years old in both rural and urban areas. The dft (sd) and DMFT (sd) were, respectively, 3.15 (3.12), 3.41 (2.69), 5.96 (4.19), 16.00 (7.30), 25.96 (9.82) and the percentages of caries free of 34.42, 14.81, 8.16 were credited for preschool children, schoolchildren and adolescents, respectively. The SiC index applied to the two younger groups were 6.65 and 6.70 increasing to 32.00 in 65 to 74 years old. At the teenager group the DMFT were 5.96 (4.19) and the Care Index still shows the low percentage of 29.40. In the elderly group, represented by 65 to 74 year olds, the data show the strongly neglected situation of oral health related with quality of life, it means almost the entire sample needed to use artificial dentures. Oral health in Monte Negro is synthesized by one word: “necessities”. Oral health promotion and the prevention of oral disease policies are urgent needs.

## 215 Antimicrobial activity of six plant extracts from the Brazilian savanna on microbial biofilms

FARDIN, A.C. (Angélica Cristiane Fardin angelicafardin@yahoo.com.br); SANGALLI, J.; GAETTI-JARDIM, E.C.; GAETTI-JARDIM JR, E.

The extracts of Ficus enormis, Maytenus ilicifolia, Myracrodruon urundeuva, Patagonula americana, Piptocarpha rotundifolia, and Psidium cattleianum are currently used in popular medicine of the Central, Northern and Northeastern Brazilian regions and they represent significant part of the biomass of Brazilian savanna. The aim of this study was to evaluate the inhibitory activity of plant extracts from these plants on periodontal bacteria and oral superinfecting bacteria and fungi. Alcoholic and aqueous extracts were prepared from leaves and stems. The minimal inhibitory concentrations were evaluated by the agar dilution method, using Wilkins-Chalgren agar supplemented with horse blood (5%), hemin (5μg/ml) and menadione (1μg/ml) for the anaerobes (P. gingivalis, P. intermedia and F. nucleatum) and microaerophiles (A. actinomycetemcomitans), and Mueller-Hinton agar for E. faecalis. Antimicrobial activity of plants extracts on microbial biofilms was determined in microplates. Psidium cattleianum and Myracrodruon urundeuva extracts demonstrated significant inhibitory activity on all bacterial strains tested; alcoholic and aqueous extracts showed similar results. Psidium cattleianum and Myracrodruon urundeuva extracts were able to inhibit microbial biofilm while other extracts that produced inhibitory effects on planktonic cells did not show a significant inhibition of microbial biofilms. The aqueous extracts of P. cattleianum was the most effective on E. faecalis, producing a Log reduction of cellular viability of E. faecalis & C. albicans biofilm after 5 min. of direct contact, while the other extracts spent more than 2 h. to produce this effect.

## 216 Squamous cell carcinoma in soft palate

MARQUETI, A.C. (Antonio Carlos Marqueti acmjab@hotmail.com); CASTRO, A.L.; CRIVELINI, M.M.; GAETTI-JARDIM JR, E.

Epidermoid carcinoma is the most common of malignant neoplasm in the oral cavity and adjacent structures. It has a higher incidence in men, particularly at the fourth decade of life and from an epidemiological point of view, it is associated with chronic use of tobacco and alcohol. This work reports a case of squamous cell carcinoma in the soft palate, emphasizing the importance of the dentist in the team of professionals treating these patients, both in the early diagnosis and in the management of the stomatological alterations resulting from the anti-neoplastic therapy. The clinical case refers to an alteration presented by a Caucasian male patient aged 47 years, who was forwarded to dental treatment due to presence of an exophytic nodular lesion on right side of the soft palate, with round shape, sessile base, rough and whitish surface, with approximately 1.5 cm in diameter, single, ulcerated, normal fibrous with adjacent mucosa. The medical history revealed allergy, hypertension and cardiopathy, being forwarded to medical evaluation for diagnosis of the lesion. After detailed extraoral and intraoral clinical examination, it was considered as differential diagnosis: capillary hemangioma, ulcerated papilloma, Ackermann verrucous carcinoma, atypical fibrous hyperplasia and inflammatory pyogenic granuloma, which was the clinical diagnosis. Tissue biopsy confirmed the diagnosis of squamous cell carcinoma degree I, and the patient was instructed to see an oncologist for treatment.

## 217 Resistance to antimicrobial agents in microorganisms isolated from oral cavity of patients with poor oral hygiene

GALLO, A.J. (Ariane Jamile Gallo arianejgallo@hotmail.com); SILVA, V.F.; GAETTI-JARDIM JR, E.C.; GAETTI-JARDIM, E.C.

Head and neck infections are usually mixed in most cases and most of microorganisms associated to these diseases are strict anaerobes, even though facultative anaerobes may be isolated in some circumstances, particularly from immune depressed patients and from infections refractory to antimicrobial treatment. In addition, just few clinical laboratories perform culture techniques or antimicrobial susceptibility tests with clinical samples obtained from these infections, not only due to technical restrictions, but also due to the misconception that oral microbiota is universally sensitive to the antimicrobial drugs most commonly used in medicine and dentistry. Then, this study evaluated the resistance to antimicrobials of microorganisms isolated from patients wearing complete dentures, gingivitis patients, periodontitis patients, and periodontally health subjects presenting poor hygiene standards. Three hundred and four aerobes or facultative anaerobes, as well as 270 strict anaerobes were tested. The minimal inhibitory concentrations of the drugs were evaluated through the agar dilution method. The Mueller-Hinton agar was used for facultative anaerobes and aerobes, while Wilkins-Chalgren agar supplemented with hemin, menadione and horse blood was employed for the strict anaerobes. In the tests, the bacterial inoculum corresponded to 105 CFU/spot, which were transferred to agar surfaces by a Steers' replicator. A significant level of resistance to most antimicrobial agents was observed for aerobes and facultative anaerobes, excepting carbapenems, rifampicin and ciprofloxacin, while strict anaerobes evidenced resistance especially to â-lactams, erythromycin, and tetracycline. Most of isolates resistant to â-lactams were â-lactamase producers and these enzymes were active against a wide range of drugs. Metronidazole was highly effective against Gram-negative anaerobes and clindamycin, imepenem, meropenem and rifampicin were highly effective against all strict anaerobes and most aerobes and facultative anaerobes.

## 218 Autogenous gingival-bone graft

BARBOSA DE PAULO, L.F. (Luiz Fernando Barbosa de Paulo nandim3@hotmail.com); SILVA, C.J.; MARQUEZ, I.M.; FURTADO, L.M.; BATISTA, J.D.; ZANETTA-BARBOSA, D.

Bone resorption after tooth extraction is a common feature that can complicate future implant placement. The placement of immediate implants into extraction sockets has been advocate to reduce this kind of bone loss but it is not always possible to place implants in a socket that has lost part or all buccal bone plate. Autogenous bone grafting is the gold standard for reconstructive procedures in this clinical situation, but is a time-consuming procedure and increases morbidity of treatment. The autogenous gingival-bone graft technique is a simple, low-morbidity procedure that can be used immediately after tooth extraction to avoid a more extensive treatment. In this case, bone reconstruction was performed using a gingival-bone graft removed from maxillary tuberosity, restoring bone volume, preserving attached soft tissue in receptor area and allowing the posterior installation of an implant in the ideal position. A 46-year-old woman with previous diagnosis of root fracture whose clinical examination showed signs of periradicular bone loss, fistulae, and 9mm periodontal probed depth in the buccal side was subjected to the treatment. Tooth extraction was performed with a periotome, preserving gingival attached tissue and remaining bone architecture and a trephine bur with similar diameter of the socket was selected and used to obtain a bone-gingival graft from the maxillary tuber. This bone-gingival graft was adapted by compression and fixed by sutures in the socket. After six months, the implant was installed, left to osseointegration and finally restored with a crown.

## 219 Clinical management of osteoradionecrosis

NAPOLES, B.B. (Bianca Barchetta de Nápoles biancanapoles@hotmail.com); SPAGNOL, G.; SANTOS, P.S.S.; FREITAS, R.R.

Osteoradionecrosis is defined as a sequela of radiotherapy. It is characterized by a loss of the mucosal lining and the consequent exposure of necrotic bone tissue for a certain period of time. This bone exposure is generally accompanied by other clinical signs and symptoms, such as oral and/or skin fistulas, muscle trismus, drainage of purulent secretion, pain, discomfort and difficulty in chewing. (Cury and Kowalski, 2003). The objective of this work is a retrospective study of the clinical management of osteoradionecrosis. Were evaluated 6 patients who were subjected to surgery and conventional radiotherapy for malignant neoplasias of the head and neck. The selected patients had to present clinical and radiographic signs of osteoradionecrosis. Treatment of the lesions was established as curettage to stimulate bleeding, irrigation with 0.12% chlorhexidine and removal of bone sequestration. Of the 6 cases assessed, 3 (50%) had purulent drainage, from which microbiological culture was carried out and antibiotic therapy was administered for 14 days (combination of amoxicillin and metronidazole) with complete regression of signs of infection. Bone exposure persisted in only 1 (16.66%) of the patients, although it was asymptomatic. In 5 (83.3%) patients there was epithelialization of the area of bone exposure, which was also asymptomatic.

## 220 Profile and professional relationship in family health program teams

MORAIS, B.G. (Bruna Guimarães Morais brubaguimaraes@yahoo.com.br); CARVALHO, M.L.; LIMA, D.C.; ARCIERI, R.M.

The work in health is essential for the human being life and has deserved bigger attention of the society in the last few decades, in virtue of its importance for the quality of life of the individuals. It was verified the profile and the interprofessional relation of the teams of the Family Health Program – FHP. Participated of this research, eight teams of the FHP and five of oral health teams (OHT) of five cities of small port of the Northwest of the State of São Paulo, Brazil. The study was quanti-qualitative and the data had been gotten by interviews with the aid of a script half-structuralized for registers. It was verified that the members of the studied teams were young (of 18 to 29 years-old), with less of one year of work in the FHP and the majority lived in the city where they worked; the doctors and oral health team did not relate with other members of the team of the FHP, as well as the easiness of the nurses to relate with them. It was evidenced in the results, the difficulty of the professionals in becoming related in the Team of the FHP. There is a necessity to enable the teams to become, in fact, multiprofessional.

## 221 Comparison of cross-sectional hardness and transversal microradiography of artificial carious enamel lesions induced by different demineralizing solutions/gels

MORON, B.M. (Bruna Mangialardo Moron brumoron@usp.br); MAGALHÃES, A.C.; COMAR, L.P. WIEGAND, A.; BUCHALLA, W.; BUZALAF, M.A.R.

The aim of this study was to compare the mineral content and cross-sectional hardness of artificial enamel caries lesions produced by different demineralizing solutions/gels. Fifty bovine enamel samples (4x4 mm) were allocated by stratified randomization according to their surface hardness (SH) values into five groups: gel I – 8% methylcellulose gel + 0.1 M lactic acid (pH 4.6, 37°C, 14 days); gel II – 20 g/L polyacrylic acid + 0.1 M lactic acid, with 500 mg/L hydroxyapatite (pH 4.8, 37°C, 16 h); buffer I – 50 mM lactic acid solution with calcium, phosphate and methyl diphosphonate (pH 5.0, 37°C, 6 days); buffer II – 50 mM acetic acid solution with calcium, phosphate and fluoride (pH 5.0, 37°C, 16 h); cycl – pH-cycling. The final surface hardness (SH1) was performed to calculate the percentage of surface hardness change (%SHC). The samples were then longitudinally sectioned and sections (138±7.6 μm) were subjected to transversal microradiography and cross-sectional hardness at 10 to 220 μm depth from the surface. There was a low linear relationship between mineral content and hardness (r2=0.43, considering all groups). Generally, all groups presented similar hardness values except between some groups at specific depths. Regarding the mineral volume, buffer I produced higher demineralization than the other groups, followed by cycling, gel I, gel II and buffer II. The mean lesion depth was significantly different between gel I (35.79 μm) and buffer I (85.55 μm). Thus, the protocols for producing artificial caries lesion differ especially when considering the method of analysis.

## 222 Oroantral communication: two case reports

TEIXEIRA, B.M. (Bruna Maggi Teixeira bruninhamaggi@hotmail.com); BASSO, L.; CAMARINI, E.T.; FARAH, G.J.; IWAKI FILHO, L.; PAVAN, A.J.

The maxillary sinus is a pneumatic space contained inside of the maxillary bone bilaterally. This cavity is associated with bone fragility and the proximity with the apex of some posterior superior teeth and allows the formation of a direct access between the sinus and oral cavity called oroantral communication, common in cases of dental extractions, cystic lesions, etc. When this access canal between these cavities is coated with epithelial tissue, it is called oroantral fistula, which generally will form within three weeks. This communication needs a surgical intervention that can be realized with displacement of retail. The purpose of this work is to present two distinct techniques for closing the oroantral communication performed in dentistry clinics of State University of Maringá. In the first case, patient P.B. 52 years old, male, was sent with suspected oroantral communication in the region of tooth 27, and complaining that after intake of liquids they came out through the nose. After the clinical and radiographic exams, the communication was recognized. The treatment of the communication was done by Caldwell-Luc technique for curettage of the oroantral membrane and surgical closure with the displacement of the body fat of the cheek. In the the second case, patient K.H., 73 years old, male, reported that an oroantral communication occurred after an extraction. During clinical examination, the patient complained about pain on palpation on the lateral wall of the maxillary sinus and the radiographic exam revealed the communication. The adopted protocol included irrigation of the maxillary sinus with saline during two weeks, with further closure of the communication by buccal flap displacement. In both cases, there was complete closure of the communications, the techniques being considered of excellent prognosis.

## 223 Efficiency and heat release of Mtwo and ProTaper Universal systems in endodontic retreatment

CAVENAGO, B.C. (Bruno Cavalini Cavenago brunocavenago@usp.br); ASSUMPÇÃO, T.S.; FIDELIS, N.S.; BERNARDINELI, N.; BRAMANTE, C.M.; GARCIA, R.B.

Recently, new rotary systems have been specifically developed for retreatment due to their safety, efficiency and speed in gutta-percha removal. The aim of this study was to investigate the cleaning efficacy, rate of extrusion, temperature variation and time during retreatment procedures using ProTaper Universal Retreatment, Mtwo Retreatmnet and manual technique. Sixty filled single rooted teeth were randomly divided into 3 groups (n = 20). The filling materials were removed with: ProTaper Universal retreatment system; Mtwo R and hand instruments. Thermoelectric pair was kept in position in order to register the temperature on the cervical and apical thirds. Roots canals walls were analyzed at 12,5 magnification. The time, temperature and cleanliness rate were subjected to Tukey's test, and extrusion to Kruskal-Wallis and Dunn's test, (P<0.05). None of the techniques used was able to completely remove all the residues from the root canal. ProTaper showed the greatest extrusion of debris, shorter time and the highest temperature variation. The Mtwo R group was significantly less able to clean.

## 224 Root reinforcement with resin materials and use of fiber posts in direct composite resin restorations

BUENO, T.L. (Tamires de Luccas Bueno tamyluc@hotmai.com); RAHAL, S.; TESTA, R.; SANTOS, M.R.; BRISO, A.L.F.

The posts are being used widely in clinical endodontically treated teeth, with the coronal tooth structure is insufficient to retain the final restoration, and are used as a central support element. However, the destruction of dental tissue can reach much of the root dentin, which undermines the ideal functioning of restraint systems. To solve this problem, adhesive restorative materials have been employed in order to strengthen the root canal and enable the use of posts. This study presents cases in which the root canal was reinforced by the adhesive technique with glass ionomer cement or composite resin, the tooth was restored with prefabricated fiber posts and restorative materials in the same session. Case follow up showed that the adhesive materials and restraint systems were effective in restoring the function and esthetics.

## 225 Influence of optical characteristics of dental substrates and resin-based materials in the success of esthetic restorations

LIBARDI, C.C. (Camila Cruz Libardi camilalibardi@hotmail.com); SVIZERO, N.R.; D'ALPINO, P.H.P.; PALMA–DIBB, R.G.; WANG, L.; ATTA, M.T.

In esthetic restorations, it is common to expect for an ideal clinical result based solely on the properties of composite resins. Besides, the selection of a correct shade of a material is a hard clinical task. Whether the clinical factors to obtain a well-polymerized restorative materials are not respected, poor optical properties would lead to restoration failure with time. Thus, the purpose of this presentation is to explore some relevant biological factors of dental substrates and the resin-based materials commonly indicated to restore them as the ultimate goal is to reach successful esthetic and functional restorations. To begin, the composition of dental tissues as well as their structural organization will be presented. Later on, composite resins in association to other restorative materials will be presented, regarding their composition, distribution and characteristics, with special attention to the optical properties and relation to light that contributes to restoration perception. Then, some laboratory and clinical experimental designs will be presented in order to demonstrate the correct restorative material application. The different outlines to be presented will reveal important sources of knowledge and information in relation to each of these clinical themes with a perspective to obtain long-lasting restorations.

## 226 Rehabilitating alternative in an edentulous patient with cleft lip and palate and severe maxillary atrophy

CIRINO, C.C.S. (Camila Camarinha da Silva Cirino camilacamarinha@yahoo.com); FIAMENGUI FILHO, J.F.; DIAS, R.P.; AMADO, F.M.; ALMEIDA, A.L.P.F.; FIGUEIREDO, C.M.

Edentulous patients with severe maxillary atrophy and considerable maxillomandibular discrepancy bring out important challenges in their oral rehabilitations. The use of osseointegrated implants are been shown to be viable in some treatments for preservation of the remaining bone ridge and great increase in retention and stability of total prosthesis. The aim of this work is to present a clinical case of a totally edentulous individual with cleft lip and palate, maxillary atrophy and great maxillomandibular discrepancy, who was rehabilitated without orthognathic surgery. This patient received maxillary bone grafts removed from the iliac crest. Next, 6 implants were installed on the maxilla and 5 on the mandible. The prosthetic planning included maxillary overdenture and mandibular fixed prosthesis. After 2 years of follow up, it was observed good stability, bone preservation and periimplant health. The obtained results demonstrate success in the rehabilitating treatment, despite difficulties of the case, with important esthetic and functional improvement.

## 227 Periodontitis: a contributing factor for the development of stroke

ROSA, C.C. (Camila Costa da Rosa camiodt@hotmail.com); GHIZONI, J.S.; TAVEIRA, L.A.A.; SANT'ANA, A.C.P.; PEREIRA, J.R.

The aim of this study is to evaluate the periodontal conditions of patients suffering from stroke compared to systemically healthy patients. Test group was comprised by 20 hospitalized patients that have developed stroke. Control group was comprised by 60 systemically healthy age-matched patients without previous history nor clinical signs and symptoms of stroke or cardiovascular events. Periodontal examination was performed by a single trained examiner and consisted of probing depth, clinical attachment level, bleeding on probing and plaque index. Periodontal disease was defined by the presence of at least one site with probing depth >4mm. The risk of developing AVC influenced by periodontal disease was very high (ORunadjusted=48.06; 95% CI: 5.96-387.72; p<0.001). Periodontal disease was more prevalent and severe in ischemic and hemorrhagic stroke than is systemically healthy patients, as observed by increased probing depth, attachment loss, bleeding on probing and plaque index (unpaired t test; p<0.05). These findings suggest that periodontal disease can be considered as a risk factor for the development of stroke.

## 228 Color change of a resilient relining material after immersion in dye solutions and brushing

DAHER, C. (Camila Daher cazinha.daher@hotmail.com); PECCINATTI, C.B.; PISANI, M.X.; MALHEIROS-SEGUNDO, A.L.; PARANHOS, H.F.O.; SILVA-LOVATO, C.H.

This study evaluated the alteration of color of a resilient relining material after immersion at food colorants and after brushing. Seventy two Mucopren Soft specimens (18mm x 3mm) were randomly distributed and immersed in coffee, coke and wine for 36 days (3 years). Next, the specimens were brushed with a bristle brush (Colgate Professional) and water alone or associated with the dentifrices Corega Brite, Experimental 1 (Zonyl) and Experimental 2 (Chloramine T) in a brushing machine for 150 minutes (3 years), with the brushes and the dentifrice suspensions were replaced every 50 minutes. Six brushes were used being one for each specimen. The color was valued by spectrocolorimetry and the ÄE*ab was calculated after immersion in the solutions and after brushing regarding the initial color. The data were analyzed by ANOVA (P <0.05) and the color alteration was quantified by the National Bureau of Standards. After 36 days, there was statistically significant difference in DE*ab between three solutions, with coffee (12.28), coke (25.25), and wine (4.32) producing high, very high and acceptable color change. After the immersion in coffee, the difference of color lessened after brushing with water (18.48) and with chloramine (14.83), but remained very high. With the wine, the difference of color lessened after brushing with all products ΔE*abT36=12.28; ΔE*abT150=10.18. After the immersion in coke, there was no difference between products of hygiene, not even between the periods.

## 229 Esthetic and functional rehabilitation with fiber-reinforced composite post and metal-free ceramic: case report

ROSATTO, C.M.P. (Camila Maria Peres de Rosatto camilamaria_pr@hotmail.com); VALDIVIA, A.D.C.M.; ROSCOE, M.G.; NOVAIS, V.R.; CAMPOS, R.E.; SOARES, C.J.

The aim of this study was to report a case of esthetic rehabilitation of maxillary anterior teeth with feldspathic veneering and crown, associating, if necessary, glass fiber-reinforced composite (FRC) post to provide optimal esthetic smile and a quality life for patient. Patient came to the Dental School of the University of Uberlândia in order to reestablish the esthetic of his maxillary anterior teeth, which had poor appearance, unsatisfactory endodontic treatments (teeth 11 and 21), extensive composite resin restorations (teeth 13, 21, 22 and 23) and metalloplastic crown (12). First it was done internal bleaching of teeth 11 and 21 using sodium perborate mixed with 30% hydrogen peroxide. Then, FRC post was cemented in the same teeth (11 and 21); this post system present similar mechanical properties to dentin, resulting in similar stress patterns. After this procedure, teeth 13, 11, 21, 22 and 23 were prepared for indirect veneering and tooth 12 was prepared for total crown. The provisional restorations had been confectioned from a diagnostic waxing, using the silicone matrix shell and composite resin. Impressions were made using a condensation silicone material and retractor wire. The definitive restorations were fabricated in feldspathic ceramic because this material warrants esthetic success, in addition to having biomechanical characteristics close to that of the enamel. The restorations were cemented with adhesive luting cement. Esthetic and functional adjustment of the restorations was done. Complete functional and esthetic rehabilitation was achieved because of the biomechanical characteristics of all used materials, confirming this as a good alternative of rehabilitation.

## 230 External control of the fluoridation of public water supply in Bauru, SP, Brazil

MORAES, C.M. (Camila Mascarenhas Moraes mila_mmoraes@hotmail.com); RAMIRES, I.; GRIZZO, L.; OLIVEIRA, R.C.; MAGALHÃES, A.C.; BUZALAF, M.A.R.

The controlled fluoridation of public water supply for caries prevention is regarded among the top ten most effective measures in public health in the world. The aim of this study was to monitor the fluoridation of the public water supply in Bauru, state of São Paulo, Brazil. The study was carried out from August 2007 to July 2008. Every month, on dates randomly established, 58 water samples were collected from the 19 supply sectors of the city, totaling 691 samples. The fluoride concentration in the water samples was determined in duplicate, using an ion-specific electrode (Orion 9609) coupled to a potentiometer. Following analysis, the samples were classified as acceptable or unacceptable according to their fluoride concentration. Descriptive statistical analysis was utilized. The mean fluoride concentration observed in the different collection months ranged from 0.10 to 2.63 mg/L. Around 86.6% of the samples were classified as acceptable and among them, 36% were regarded as optimum. Additionally, 12.2% of the samples were considered inadequate (more than 0.84 mg fluoride/L). The results indicate a tendency from optimum fluoridation to overfluoridation. When the present data are compared to previous data reported for the city, it can be seen that the conditions of public water fluoridation have been improved after the implementation of the external monitoring. Thus, the implementation of the monitoring of water supply fluoridation by means of surveillance systems must be stimulated, since this is fundamental for controlling dental caries.

## 231 Initial approach to the polytraumatized patient: need for multidisciplinary intervention

CANDIDO, G.C. (Graciela Cristina Candido gracicristina@hotmail.com); COSTA, F.; FARAH, G.J.; IWAKI FILHO, L.; TRINDADE, J.P.; CANDIDO, A.G.; SILVA, P.V.R.

Facial trauma is a reality in emergency services, mainly due to the increase of automobile accidents, which usually cause systemic lesions concomitant with lesions on the face of patients. Therefore, the presence of a multidisciplinary team in the initial care is of fundamental importance because of the impact of injuries on various organ systems, aiming at reducing the morbidity and mortality. The treatment of victims of severe trauma requires rapid assessment of injuries and establishment of therapeutic measures to preserve the patient's life, leading him/her to a homeostasis status, normal pressure and volemia. Our objectives are to emphasize the importance of multidisciplinary management at the emergency care, particularly in this type of patient, and to report the case of a male patient, 29 year old, victim of a motorcycle accident who presented with extensive lacerations in the face, and crepitus in middle third of the face and total disarticulation of the zygomatic bone, jaw, and dental avulsions. He was diagnosed with Le Fort III fracture on the left side, Le Fort II on the right side, frontal fracture, nose bone fracture, bilateral clavicle fracture, and need for enucleation of the right eyeball. We conclude that the multidisciplinary care to polytraumatized patients, offers them a greater survival chance, so they can withstand the consequences inherent to this type of trauma, as well as prevent the occurrence of sequelae of greater magnitude.

## 232 Foramen of vesalius: complementary study for a safer planning and execution of the trigeminal rhizotomy technique

MELO, C.G.S. (Carina Guimarães de Souza Melo carinamgs@yahoo.com.br); SHINOHARA, A.L.; SILVEIRA, E.M.V.; LAURIS, J.R.P.; ANDREO, J.C.; RODRIGUES, A.C.

The sphenoid bone stands out as a complex structure centrally located in the skull base which articulates with many facial bones. This bone presents several foramina which allow the extracranial passage of nerves and veins. Among these foramina is the foramen of Vesalius (FV) which allows the passage of emissary veins, communicating the cavernous sinus to the pterygoid venous plexus. This fact highlights its importance because clinically it can transmit serious infections to the middle cranial fossa. The FV is located in the greater wing of sphenoid bone between the foramen ovale (FO) and the foramen rotundum (FR), and anteriorly and medially to the FO. The FO allows the passage of the mandibular branch of trigeminal nerve, which is the target of the trigeminal radiofrequency rhizotomy (TR). The TR is a surgical technique used in the treatment of the trigeminal neuralgia (TN). This technique promotes a great pain relief in the immediate postoperative. In this procedure a puncture of the medial margin of the FO is performed to reach the trigeminal ganglion. During this procedure many attempts are made before the right puncture, and the FV can be accidentally reached. A needle insertion through the FV can cause serious complications. Actually in the TR, the professional is guided by high-tech equipment, but the knowledge of the relevant anatomy makes this technique safer and precise. Therefore, this study intend to offer specific anatomical data with morphological patterns (macroscopic and mesoscopic), to increase the understanding of the FV features as frequency, incidence and important distances with adjacent foramina. This information can help the surgeon to improve the planning, and to have a safer execution of the TR by the fact of avoiding the accidental puncture of the FV.

## 233 Mini-implant and lingual button as an anchorage to initial retraction of upper canines: a prospective study in casts

MENDES, C.C. (Carla Corrêa Mendes carla_cmendes@hotmail.com); ARANTES, F.M.; GONÇALVES, M.J.B.; KINA, J.; GURGEL, J.A.; SANTOS, E.C.A.

Osseous anchorage replaces the traditional procedures such as extrabucal appliance, allowing the use of continuous forces and reducing the time of treatment. For these reasons and the indiscriminate use of mini implants in the clinical practice, the interest about mini implants in relation to the loss of anchorage was increasing. The aim of this research was to compare the loss of anchorage in study casts after initial retraction of upper canines between two groups. The patients were assessed in the Department of Orthodontics of Araçatuba School of Dentistry – UNESP. Eighteen patients were randomly chosen and divided in two groups (A and B) of 9 subjects each. Mini implants were placed in group A and lingual button were placed in group B. Two study casts were obtained for each patient, one at the beginning of the treatment (M1) and the other at the end of canine retraction (M2). The measurement of study casts were performed using the Autodesk Autocad 2007 program. All measurements were tabulated and subjected to analysis by paired “t” test to verify the intra-examiner method of error. To determine the casual error, was used the formula proposed by Dahlberg (Houston, 1983). To compare the initial and the final phase was used the paired “t” test. Student's “t” test was applied to compare between the mini implant and lingual button groups. A significance level of 5% was used for all of tests. The statistical analysis was performed in the Statistica for Windows version 5.1 software (StatSoft Inc, Tulsa, USA). There was no statistically significant difference between the two groups comparing in the study casts the loss of anchorage of molars after the initial retraction of the upper canines using two different systems of anchorage (mini implants and lingual button).

## 234 Use of direct esthetic restorations in the esthetic rehabilitation of smile

GUIMARÃES, C.M. (Carlla Martins Guimarães carlla.martins@gmail.com); PEREIRA, F.A.; REIS, B.R.; SILVA, G.R.; SOARES, C.J.; SOARES, P.V.

One of the simple techniques to resolve the presence or absence of diastema of contact between two or more consecutive teeth is esthetic recontouring using composite resin restorations to change the characteristics of shape, contour and position of teeth. Patient S.J.B, 20 years old, presented to the Integrated Dental Clinical, Federal University of Uberlandia, with the main complaint the esthetic appearance of her smile, showing diastema in upper anterior teeth. It was the planned closure of the diastema and processing with direct dental restorations with composite resins. Impressions were taken followed by waxing and fabrication of a silicone matrix. Next, the following procedures were done: selection of resin shade; rubber dam placement; reduction of the mesial and incisal faces of the upper right canine; checking of the silicone matrix, verifying the fit and the correct palatal contour; acid etching; application of adhesive system; filling of the silicone matrix with composite resin in order to construct a palatal guide followed by resin polymerization inside the matrix; incremental insertion of composite resin respecting dental shape and contour; occlusal adjustment and finishing and polishing. The nano-composite resin used has good polishing surface with biomimetic optical properties. The use of direct composite resin restorations restored esthetics and function, increasing the patient's quality of life in a simple and conservative manner.

## 235 Accuracy of the guided-surgery technique in edentulous maxillas (NEOGUIDE)

BARROS, C.A.V.S. (Carlos Alberto Villaça de Souza Barros -carlosvillaca@intitutodoimplante.com.br); VIEIRA, D.M.; REIS, E.S.; ROSSETTI, P.H.O.; PADOVAN, L.E.M.; FRANCISCHONE, C.E.

The aim of this study was to evaluate the precision of flapless, guided surgery comparing angular and linear deviations of cone-morse dental implants before and after surgical procedures through computed tomography (CT) slices generated on a computerized three-dimensional virtual environment. Five completely edentulous patients were enrolled in this pilot study. After reverse planning for tooth set-up and tomographic guide fabrication, CTs were made 0.2mm thick with images generated on Dental Slice (Bioparts) for placement of Titamax EX cone-morse implants (Neoguide, Neodent; 3.5-3.75mm in diameter). After implant placement, new CTs were made following the same protocol and transferred to Dental Slice software. Mean angular deviation values were of2.033 ± 0.782°; registered linear differences at implant platform, middle and apical thirds were 1.082 ± 0.907mm, 1.599 ± 1.22mm, and 2.542 ± 2.047mm, respectively. All acrylic-veneered, metal-reinforced definitive prostheses were installed after 48 hours and occlusal adjustments provided after 72 hours. No clinical complications were seen, with all implants and prostheses remaining into function. Within the limits of this study, the results showed that this technique is safe and accurate for routinely dental practice. However, these values must be confirmed in further studies.

## 236 Conservative treatment by pulp curettage: a case report

FRANCISCONI, C.F. (Carolina Fávaro Francisconi carolff@usp.br); FRANCISCONI, L.F.; SCAFFA, P.M.C.; SILVA, R.V.C.; CASAS-APAYCO, L.C.; FRANCISCONI, P.A.S.

The diagnostic concepts and techniques and the conservation of the vitality of the dentin-pulp complex have received special attention from Dentistry and are essential for the success of the restorations from the biological point of view. The pulp tissue keeps the vitality of the dentin and the needed physiology to neutralize tensions resulting from mastication, being responsible for all the answering mechanisms against stimuli that, together, configure its defensive action while the pulp is biologically active. This case report intends to elucidate obtained results on the restoration of tooth #36, after pulp curettage, following-up, mineralized barrier formation and application of a composite resin, according to the concepts of dentin-pulp complex protection. Treatment began with anamnesis and a detailed clinical examination, considering pain resulting from thermal stimuli and presence of temporary material in an occluso-mesial extensive Class II cavity on tooth #36 as the main patient's complaints. Under rubber dam, after removal of remnant restorative material and decayed tissue, it was observed pulp exposure due to the evolution of the caries process. This way, pulp curettage was accomplished, extending the exposed area, for application of calcium hydroxide powder and cement. Subsequently, after rebuilding of the mesial wall with composite resin to warrant the local resistance to masticatory efforts, the cavity was filled with a glass ionomer cement. After 90 days, the ionomer was replaced by composite resin, following a new protection of the dentin-pulp complex, as vitality tests were positive and there was formation of a mineralized barrier both radiographically and clinically. Immediate evaluation showed patient satisfaction and constant evaluation shows treatment effectiveness until the present moment. Thus, by employing a conservative treatment such as curettage it was possible to preserve pulp vitality, avoiding an endodontic treatment or extraction in a worst case scenario.

## 237 Alveolar ridge reconstruction with autogenous bone graft combined with platelet-rich plasma: a case report

SANTINONI, C.S. (Carolina Santinoni natidecampos@gmail.com); MESSORA, M.R.; NAGATA, M.J.H.; BOSCO, A.F.; FURLANETO, F.A.C.; GUSKUMA, M.H.

Alveolar ridge deformities are one of the greatest problems in oral rehabilitation and may impair patients' function and esthetics. A sufficient amount of bone volume is required for the placement of osseointegrated implants. Among the techniques described for alveolar ridge reconstruction, particulate and block form of autogenous bone graft can be used to correct horizontal and vertical alveolar ridge deformities. Many factors, such as the local availability of growth factors, have been considered important to the incorporation of bone grafts. The use of platelet-rich plasma (PRP) is based on the premise that platelets constitute a reservoir of critical growth factors, which once released, may positively regulate the wound healing process. Currently, studies have been reported on the regenerative capacity of PRP, which has been successfully combined with bone grafts in oral reconstructions and in Implantology. The purpose of this study was to report on a case with a horizontal alveolar ridge deficiency (Class I by Seibert 1983) in the anterior area of the maxilla, treated by PRP combined with autogenous bone block graft. After 6 months, the thickness of the alveolar ridge was enhanced and the placement of an osseointegrated implant in the area was possible. The use of PRP combined with bone graft can be successfully used in the reconstruction of alveolar ridge deficiencies.

## 238 *In situ* evaluation of the effect of sucrose on the biofilm in a short-term experimental period

LODI, C.S. (Carolina Simonetti Lodi carol_lodi@yahoo.com.br); AMARAL, J.G.; DELBEM, A.C.B.; SASSAKI, K.T.; MARTINHON, C.C.R.

Sucrose is considered the most cariogenic carbohydrate since it is fermented to acids and also metabolized to intracellular and extracellular polysaccharides by microorganisms from biofilm. The aim of this study was to evaluate the dental demineralization and the inorganic composition of the biofilm using 30% sucrose solution in a short-term experimental period. Ten volunteers wore palatine appliances containing 4 blocks of bovine dental enamel during 3 phases of 7 days each. The blocks were exposed to 30% sucrose solution 6 times/day and volunteers used placebo, fluoride dentifrice of 500 μg F/g or 1100 μg F/g. The concentrations of fluoride (F), calcium (Ca), phosphate (P) and alkali-soluble carbohydrates on the biofilm were analyzed. The analysis of F was accomplished with ion specific electrode and the Ca, P and carbohydrates with the spectrophotometer. The initial and final microhardness were determined for evaluation of the mineral loss. The data were subjected to the Kruskal Wallis and Miller tests (p<0.05). The results showed that the 1100 μg F/g dentifrice presented the highest concentration of F, Ca, and P ions in the biofilm followed by 500 μg F/g dentifrice and placebo. Carbohydrate concentration and the percentage of mineral loss were the highest in the placebo and the lowest in the fluoride dentifrices. According to the results, it was observed that 7-day experimental period and 30% sucrose solution were sufficient to the cariogenic biofilm formation with demineralization of the enamel when exposed to the challenge 6 times/day, even with the use of fluoride dentifrice.

## 239 Anterior open bite: fixed or removable appliance?

KNOB, C.D. (Carollinie Dias Knob carolknob@usp.br); LANCIA, M.; SATHLER, R.; FERNANDES, T.M.F.; HENRIQUES, J.F.C.

Open bite can be defined as a negative overbite measured on the incisal edges of the upper and lower teeth and can occur in the anterior and posterior region. The anterior open bite deserves emphasis, because its prevalence is higher than the posterior open bite. It is known that the etiology of this malocclusion is multifactorial and among several factors, the most common are: deleterious oral habits, hereditary, tongue interposition, mouth breathing and trauma. After five years of age, this malocclusion requires an immediate intervention because the earlier diagnosis and treatment is chosen, the better, faster and the more stable results are. Interception of the anterior open bite can be achieved with various types of appliances and techniques. Treatment usually consists of interrupting the deleterious habits such as tongue interposition and digital or pacifier sucking, allowing dentoalveolar development without interference. The aim of this study is to present an updated review on the etiology, diagnosis and treatment of anterior open bite, demonstrating that treatment success is achieved more quickly when the dentist does not need patient cooperation with the use of removable appliances.

## 240 Analysis of variation at artificial tooth position according to the processing of mandibular complete dentures measured by a graphic computer system

ZAZE, C.A. (Cesar Aurélio Zaze cesarzaze@terra.com.br); GENNARI FILHO, H.

The use of specific techniques and materials in complete denture processing results in prosthetic rehabilitation with less adjustment needs at the time of installation, allowing minor changes in artificial tooth anatomy in order to significantly improve its function. Forty dentures were waxed and divided into four groups of 10 specimens each. The groups were fabricated in the following manner: Group 1 - Processed in metallic flask, stone plaster type III and polymerized in hot water; Group 2 - Processed in fiberglass flask, stone plaster type III and polymerized in microwave; Group 3 - Processed in metallic flask, silicon and stone plaster type III and polymerized in hot water; Group 4 - Processed in fiberglass flask, silicon and stone plaster type III and polymerized in microwave. Five pre-established points on the teeth and three pre-established points on denture base were used to draw straight lines and than measure these lines comparing teeth position before and after denture processing. As a way of obtaining the measures, we used the Auto Cad. The figures were subjected to ANOVA and Tukey's test and showed that there was change in tooth position in all processing techniques. Group 3 showed the lowest values of changes in teeth position, followed by groups 4, 2 and 1. The fact that there are changes in all processing techniques is consistent with findings in scientific literature as well as the fact of group 1 have shown the highest values of change. Regarding the lower values of change, there is divergence of our results from the findings in the literature and this is possibly due to the fact that we used mandibular prosthesis and all studies that we can compare results to used maxillary dentures. Thus, all processing techniques change in some degree artificial tooth position, what indicates a new occlusal adjustment during installation.

## 241 Effect of Ricinus communis solution on color alteration and roughness of soft relining material

PECCINATTI, C.B. (Christiane Baghin Peccinatti chris.peccinatti@gmail.com); DAHER, C.; PISANI, M.X.; PARANHOS, H.F.O.; SOUZA, R.F.; SILVALOVATO, C.H. FAPESP IC n° 08/52133-6

The aim of this study was to evaluate the influence of an experimental Ricinus communis solution for denture cleansing on color alteration and roughness of a soft relining material after immersion in beverages. Fifty-four specimens (18mm x 3mm) of a relining material (Mucopren Soft, Kettenbach, Germany) were made and randomly divided into three groups (n = 18): negative control (distilled water, 37oC), positive Control (1% hypochlorite) and experimental (Ricinus communis). The immersion in beverages (coffee, Coke, wine) was simulated for 36 days, representing 3 years. After that, the specimens were immersed in cleansers for 15 days, simulating 20-minute daily immersions for 3 years. The color and surface roughness were assessed after finishing, after staining and after immersion in cleansers. Data from each group of beverages were analyzed independently with the Kruskal-Wallis test (P < 0.05). The color analysis obtained after immersion in coffee and Coke and after immersion in the cleansers indicated that the hypochlorite caused a significant color alteration and Ricinus communis was statistically similar to water. After immersion in wine, there was a difference between the solutions and hypochlorite caused the highest color change, followed by Ricinus communis and water. Regarding the surface roughness, there was no statistically significant alteration in the groups of coffee and Coke. After immersion in wine, there was an increase in roughness compared to the baseline values, hypochlorite caused a reduction in this property, while the other cleansers did not cause roughness alteration. All tested cleansers produced significant color alterations. Sodium hypochlorite was the main responsible for color alteration and roughness reduction in the wine group.

## 242 Viability of human peripheral mononuclear blood cells maintained in the Euro-Collins® solution

MARTINS, C.M. (Christine Men Martins christinemen@hotmail.com); SCHUTZ, C.Y.K.; SELL, A.M.; GARCIA, J.N.B.; CASAROTTO, A.R.; HIDALGO, M.M.

The Euro-Collins® is a solution for the gravitational perfusion and hypothermic storage of kidneys for donation. Its features attracted the interest for possible use as a storage medium for avulsed teeth. In this study, we compared the viability of human mononuclear cells maintained for 24 hours in Euro-Collins® in relation to milk, a traditionally indicated storage medium, and the controls: Hanks balanced salt solution (HBSS - positive) and distilled water (negative). The study evaluated freshly opened Euro-Collins® and the solution open for 30 and 120 days and kept at room temperature of 25°C. The cells were isolated from peripheral blood of healthy donors (n = 4), stored in the test media and samples were collected at 0, 1 h, 3 h, 6 h and 24 h to be analyzed by the Trypan blue dye exclusion method for assessing cell viability.. The results were subjected to ANOVA and Contrast statistical tests. Analyzed over the time, Euro-Collins® opened 120 days had worse performance (p <0.05) from the first hour than milk and HBSS, and better than distilled water. The Euro-Collins® opened 30 days and freshly open differed from the control HBSS (p <0.05) at 3 and 6h, respectively. Also it was measured the pH of all solutions and only distilled water showed pH incompatible with cell proliferation. The results suggest that under the tested experimental conditions, the Euro-Collins® may be indicated for the maintenance of cell viability for less than 3h, using freshly opened solutions or opened for a maximum period of 30 days. Studies should be performed with other cell types.

## 243 Evaluate of setting time and solubility of five root-end filling materials

GOMES, C.C. (Cibele Cristina Gomes cibelegomes@lpnet.com.br); DUARTE, M.A.H.; BONELLI, A.C.C.; GAMBARINI, C.T.A.G.; MORAES, I.G.; VIVAN, R.R.

The purpose of the this study was to analyze the setting time and solubility of 5 root-end filling materials (white MTA Angelus, MTA Bio, light-curing MTA, Sealepox RP and Portland cement clinker associated with bismuth oxide and calcium sulfate). The tests were carried out in accordance with ADA's #57 specification with the addition of the needle of 456,6 g for the setting time, in accordance with the ASTM's #C266-03 specification. In relation to the setting time, the results showed that MTA Angelus presented the shortest initial setting time together with MTA Bio, followed by Portland cement clinker associated with bismuth oxide and calcium sulfate. The logest initial setting time was of Sealepox RP. Portland cement clinker associated with bismuth oxide and calcium sulfate was the material that presented the longest final setting time, followed by Sealepox RP and MTA Angelus and Bio (thre two one with the same times). Regarding the solubility, the lowest values were found for light-curing MTA, followed by Sealepox RP. MTA Angelus and MTA Bio presented the highest values, followed by Portland cement clinker associated with bismuth oxide and calcium sulfate. It may be concluded that the setting time vary a lot among the evaluated materials. Regarding the solubility, light-curing MTA and Sealepox RP presented values within the limits established I the ISO 6876 (2001) standard.

## 244 *Aggregatibacter actinomycetemcomitans* and bacteria of red complex in subgingival biofilm of native Brazilians from Umutina Indian Reservation

CIESIELSKI, F.I.N. (Francisco Isaak Nícolas Ciesielski franisaak@uol.com.br);VIEIRA, E.M.M.; ÁVILA-CAMPOS, M.J.; GAETTI-JARDIM JR, E.

By the last three decades, specific microorganisms have been implicated as etiologic agents of periodontal diseases, particularly the members of red complex and Aggregatibacter actinomycetemcomitans, which has also evidenced a remarkable association with ethnicity and human migrations. Thus, the aim of this investigation was to evaluate the occurrence of such microbial groups in Indians from Umutina Indian Reservation, Mato Grosso. One hundred adults of Umutina, Paresi, Bororo, Bakairi, Kayabi, Irantxe, Nambikwara and Terena ethnicities were examined and samples of saliva, supragingival, and subgingival biofilms were collected. The presence of *A. actinomycetemcomitans, T. forsythia, P. gingivalis and T. denticola* was evaluated by PCR and real-time PCR using specific primers and probes. The comparisons of plaque index and other clinical parameters were performed by mean of Kruskall-Wallis test. Pairwise comparisons were performed by mean of Bonferroni adjusted nonparametric Mann-Whitney test, while differences in the occurrence of selected microorganisms were evaluated by weighted least square method. By PCR, *A. actinomycetemcomitans* was detected from 8.33% gingivitis patients and 34.21% periodontitis patients, while by real-time PCR this microaerophillic bacterium was detected from 8.33% healthy subjects, 16.67% gingivitis patients and from 42.10% periodontitis patients. By PCR, *P. gingivalis* was detected from 21.43% healthy subjects, 47.92% gingivitis natives and 78.92% periodontitis patients, similar values were obtained by real-time PCR. *T. forsythia* was rarely detected from healthy subjects and by real-time PCR 39.58% and 65.79% of gingivitis and periodontitis patients were colonized by this microorganism, respectively. In addition, *T. denticola* was rarely detected and whenever present its population was significantly reduced in comparison to the other anaerobes. The occurrence of *A. actinomycetemcomitans* and *P. gingivalis* was higher from subjects with poor oral hygiene, while *A. actinomycetemcomitans, P. gingiva*lis and *T. forsythia* were associated to bleeding on probing and periodontitis.

## 245 Removal of fractured instrument and gutta-percha extruding the apex through the canal and placement of a MTA plug: case report

RODRIGUES, C.T. (Clarissa Teles Rodrigues clarit@uol.com.br); BRAMANTE, C.V.; BRAMANTE, A.S.; GARCIA, R.B.; MORAES, I.G.; BERNARDINELI, N.

J.A.S., a male patient, presented to the clinic of endodontics of Bauru School Dentistry complaining of painful sensation on chewing and on touching the right maxillary lateral incisor. The clinical examination showed coronal access and fistula. The radiographic exam showed the presence of a fractured instrument and gutta-percha, both beyond the apex and the presence of periapical lesion. The operative field was isolated and the coronal access was corrected and an attempt of removal of the fractured instrument was performed. Due to the impossibility of removal through conventional methods, the Masseram Kit System was used and then the removal was achieved. Subsequently, with the aid of a Hedstroen file, an attempt to remove the gutta-percha was done, but it was only partly removed and part of this material extruded to the periapical area. Intracanal dressing was placed and the patient was dismissed. In the following session, after a new radiograph, it was observed that the gutta-percha had been displaced, apparently to the within the root canal. A Hedstroen file was then used in order to complement its removal. Iodoform-calcium hydroxide dressing was then applied and the presence of apical resorption was noted. In the next session, a MTA plug was applied and the canal was obturated. Closure of the fistula was achieved and the control radiograph showed remission of the periapical lesion.

## 246 Effect of raloxifene and estrogen on bone metabolism of rats

OLIVEIRA, C.B. (Claudiel Batista de Oliveira claudieloliveira@gmail.com); ALMEIDA, L.R.B.; NAKAMUNE, A.C.M.S.; DORNELLES, R.C.M.

Financial Support: FAPESP

The total amount of bone mass in women is directly related to plasmatic concentration of estrogen (E2). With increasing age, the production and secretion of less E2 trigger loss of bone associated with menopause (Bone 19:185 S, 1996). The Raloxifene hydrochloride has selective agonist or antagonist activity on the tissues that respond to estrogen. The objective of this study was to analyze the process of alveolar repair and the plasmatic concentrations of phosphorus, calcium and estradiol in rats in different experimental situations. Were used 32 Wistar rats, and 8 intact rats (2 m) with regular estrous cycle and 24 rats (18 m) ovariectomized (OVX) one year ago. During 60 days, the OVX rats received pellets containing estrogen (400 ug/17b-estradiol) or corn oil, these pellets exchanged every 30 days. Other group of OVX rats received raloxifene (1mg/Kg/d) by gavage. Under general anesthesia, the animals were subjected to extraction (28 days before euthanasia) of the upper right incisors for further analysis of the of alveolar repair process. At the end of the treatment period, animals were anesthetized (xylazine - 4 mg / kg, ketamine - 40 mg / kg) for blood collection and the jaws were removed, decalcified and dried for the preparation of microscopic slides. The histometric analysis revealed bone formation of 51.12% (intact), 30.99% (OVX/oil), 50.28% (OVX/E2) and 36.93% (OVX / RLX) in the middle third of the animals studied. The plasma analysis showed significantly higher concentration of phosphorus in the group of OVX rats pre-treated with raloxifene and the concentration of calcium was lower in animals of group OVX/E2. In ovariectomized animals that received hormone replacement, the plasma concentration of E2 was significantly higher than that of the other groups. The 1-year results suggest potent activity of E2 and subtle of raloxifene on bone formation in OVX animals.

## 247 Occurrence of *Helicobacter pylori* in the oral cavity of native Brazilians

RIBEIRO, C.P. (Clícia Pereira Ribeiro cliciaribeiro@foa.unesp.br); HILDEBRAND, M.C.; CIESIELSKI, F.I.N.; VIEIRA, E.M.M.; GAETTI-JARDIM JR, E.

FAPESP 2007/51016-3

The periodontium has been suspected as an extra-gastroduodenal reservoir for *H. pylori* infection and transmission, but conflicting evidence exists about the occurrence of this microorganism in pre-Colombian societies that maintain strong relationship with the ancestors' traditions. Thus, the aim of this study was to determine the occurrence of *H. pylori* in the subgingival biofilm of 100 native Brazilians from Umutina Indian Reservation, Mato Grosso State, with different conditions of periodontal health, in comparison to a group of non-Indians presenting similar periodontal and socioeconomic status. Relevant dental and periodontal parameters and general health parameters, were recorded. Samples of subgingival biofilm were collected and the presence of *H. pylori* DNA was evaluated through nested PCR. The second PCR was performed using 5 ìL of the first PCR reaction. Primers for both rounds of PCR were designed to amplify *H. pylori*-specific segments within the bacterial 16S rDNA. The primers used in first-round PCR were EHC-U and EHC-L, while in the second round of amplification, primers ET-5U and ET-5L were used, producing a 230-bp fragment within the amplicon of the first-round PCR. In Indians, this bacterium was detected in subgingival biofilm from 21.43% healthy subjects, 33.33% gingivitis patients and 34.21% periodontitis patients. In periodontal specimens from non-Indians, the presence of *H. pylori* was observed from 32.65% healthy subjects, 26.67% gingivitis patients and 38.46% periodontitis patients. This confirms the role of oral cavity as a reservoir of the microorganism and it was not found any significant difference between the occurrences of this rod from Indians and non-Indians, despite of deep differences observed in the diet and personal habits of these communities, and this microorganism did not evidence any association with periodontal status of the patients.

## 248 Use of rapid-prototyping models in oral and maxillofacial surgery: cases report

COELHO, D. (Diogo Coelho coelhobmf@hotmail.com); MOTTA-JÚNIOR, J.; FERNANDES, S.K.; MATHEUS, A.R.; STABILE, V.A.G.; PINTO, R.J.

Rapid-prototyping models are prototypes obtained from computed tomography images. The solid replica of the anatomy of the patient allows the measure of structures, simulation of osteotomies, resection techniques, previous adaptation of internal fixation system and alloplastic implants, minimizing the possibility of errors in planning and leading to more predictable outcomes. This tends to reduce the time of the surgical procedure, and therefore hospital costs, there is still improvement in outcome and lowering the overall cost of treatment. The limitations of the technique are the accessibility of technology, the time taken for confection and the cost of biomodelling. The aim of this study is to provide rapid-prototyping as an auxiliary resource in Oral and Maxillofacial Surgery by the presentation of a case report.

## 249 Clinical analysis of the treatment of multiple gingival recessions through coronally advanced flap with or without subepithelial connective tissue graft

INGRACI - DE LUCIA, C. (Conrado Ingraci De Lucia conrado.ingraci@yahoo.com.br); MAIA, L.P.; SILVA, E.R.; TABA JÚNIOR, M.; GRISI, M.F.M.; REINO, D.M.

Gingival recession has as etiology the inflammation caused by plaque or mechanical trauma, and affects great part of people around the world, damaging esthetics, making oral hygiene difficult and facilitating the occurrence of root caries. For the treatment of multiple gingival recessions, Zucchelli & De Sanctis in 2000 described a technique that involves slidig of a buccal flap to a more coronal position to treat several gingival recessions simultaneously. However, this technique does not increase the thickness of the attached gingiva. To increase the amount of attached gingiva, a modification of this technique has been done by partial sliding of the buccal flap to a coronal position associated with the use of subepithelial connective tissue graft. This case report compares the results of the implementation of both techniques for the treatment of multiple gingival recessions. A male patient, with multiple gingival recessions and dental abrasions in the first and second quadrants received treatment of supragingival scaling, plaque control and oral hygiene instructions. When the plaque index was less than 20%, the surgical procedures were performed, a different technique being made for each affected quadrant. Evaluations were made at one week, two weeks, one month and after six months. Unsatisfactory root coverage was obtained by use of the original technique, but there was great root coverage and gain in gingival thickness in the quadrant that received the modified technique with the use of the subepithelial connective tissue graft, wich was donated by the palate. This case report demonstrates the superiority of the modified technique for the multiple root coverage of teeth with dental abrasion.

## 250 Multidisciplinary treatment in a case of epidermoid carcinoma in the retromolar region

COSTA, R.R. (Renan Roberto da Costa rena_n@hotmail.com); KAMEI, N.C.

Epidermoid carcinoma is the malignant neoplasia with the higest incidence in the oral cavity. Its etiology is related to extrinsic (patients's habits) and intrinsic factors (systemic state). The lesion is often found in adult Caucasian males. The occurrence of metastasis is due the delay in the diagnosis of the lesion. The treatment is surgical and the risk of recurrence depends on the tumor's stage. This study intended to demonstrate the multidisciplinary performance of the oral and maxillofacial surgeon with head and neck surgeon by the presentation of a case of epidermoid carcinoma in the retromolar region that needed cervical emptying. The lesion of interest in both areas leads to the formulation of a multidisciplinary team still poorly reported. However, this type of performance provides an effective and qualified attendance giving satisfactory outcomes.

## 251 Paracoccidioidomycosis: oral manifestations: a case report

NASCIMENTO, D.O.R. (Dabiliane Oliveira Rodrigues Nascimento dabi_musa@hotmail.com); ALEIXO, R.Q.; RODRIGUES, M.T.V.; COSTA, M.R.S.N.; GALVÃO, N.S.; ALMEIDA, D.L.

Paracoccidioidomycosis (PCM) is a systemic and endemic fungal infection caused by Paracoccidioides brasilienses, dimorphic and native from South America, found in soil, plant and water of rural areas. The human contamination occurs by inhalation and primary infection is pulmonary. It is reported chronic, acute/subacute and sequelae or residual clinical forms. The purpose of this study is report a case of oral lesions of PCM and the dentist's role in signs and symptoms identifications. A 49-year-old black male, smoker, farmer, complained of “pain in the lips and cheek, the inside”, 4 months ago. After intraoral examination, it was observed ulcerative “mulberry-like” lesions in the inferior and superior buccal sulcus, cheeks bilaterally, attached gingiva, alveolar ridge and oral floor, lower macroqueilia and regional limphadenopathy, typical secundary features of PCM. Incisional biopsy was performed in lower lip and attached gingival. Histopathological analysis confirmed clinical presumptive diagnosis: PCM. Patient was referred to the center of reference in systemic mycosis (CEMETRON) for systemic therapy. PCM patients are usually male, adults, farmers, smokers and expressing chronic PCM. They may complain of sleepness, weakness, inappetence, dysphagia, dyspnea, cough, hemoptysis, fever, weight loss, pruritus and burning. On extraoral examination there is macroqueilia, pallor, swelling and cervical lymphadenopathy. There may be relapse in wound healing after tooth extractions. In the mouth, the places most affected are lips, cheeks, floor, tongue and pharynx. The periodontum can be compromised, resulting in tooth mobility. Microscopically, the lesions show multibudding yeast issue with “Mickey Mouse” aspect. The findings observed in this patient coincided with the literature, emphasizing the dentist importance in using routinely diagnostic methods, biopsy when necessary for correct analysis of diseases that affect the patient systemically, and for the differential diagnosis: squamous cell carcinoma, hystoplasmosis, tuberculosis, coccidioidomycosis, syphilis, Weneger's granulomatosis, sarcoidosis and leishmaniasis.

## 252 Cleidocranial dysplasia: orthodontic-surgery approach

RESENDE, D.A. (Daniel Araújo Resende danielodonto63@hotmail.com); DUARTE, D.M.; FURTADO, L.M.; BARBOSA, D.Z.; MARQUEZ, I.M.

Cleidocranial dysplasia is characterised by bone imperfections concerning the cranium and clavicle, and it demonstrates dominant autosonomal hereditary patterns. Patients' appearance is peculiar for their low height and big head with frontal and parietal protuberances. Ocular hypertelorism and the widening of nose basis accompanied by the lowering of nose's back are also checked characteristics. At radiographs sutures and fontanels demonstrate late closing or can continue open during the patients' life. There are single manifestations in the face bones. Patients' usually demonstrate high narrow arched palate and prolonged deciduous teeth retention or complete lack of permanent teeth eruption. These factors contribute to inadequate development of the maxilla and mandible causing teeth-skeletal deformity. This wor describes the case of a 17-year-old female cleidocranial dysplasia carrier patient with teeth-skeletal alterations characterised by deciduous teeth presence, not disrupt supernumerary and many permanent teeth and anteroposterior maxilla deficiency. Orthodontic-surgery treatment is proposed to establish a functional occlusion combined with harmonious face shape.

## 253 A comparative study of caries preventive effects of a new glass ionomer sealant and fluoride varnish in newly erupted permanent first molars

OLIVEIRA, D.C. (Daniela Cristina de Oliveira danielaoliveira975@hotmail.com); CUNHA, R.F.

It is in childhood that posterior permanent teeth are more likely to be affected by dental caries. In that period, during eruption, the enamel is not fully matured, the child and parents often do not know that a new tooth is emerging and it is usually difficult for the child to clean the erupting tooth surfaces. Application of pit-and-fissure sealants and topical fluoride are widely used preventive measures, and their effectiveness in caries prevention has been proved by systematic reviews. The aim of this study was to compare the effectiveness of a glass ionomer sealant (GIS) with fluoride varnish (FV) in the prevention of dental caries on permanent first molars. The trial targeted eighty children aged 6 to 8 years, with all four newly erupted permanent first molars present. Two permanent first molars on one side of the mouth were sealed with Fuji Triage, a glass ionomer sealant; fluoride varnish (DURAFLUOR) was applied on the other two permanent first molars. Evaluation of sealant retention was performed after 6 months, using Simonsen's criteria. The effectiveness of both materials in the prevention of dental caries was also evaluated. Out of the 160 sealed teeth, 13 (8%) presented full sealant retention, 127 (79%) presented partial sealant retention, and the sealant was missing in 20 teeth (13%). Regarding the preventive effect on the sample of 320 teeth, only 12 (4%) treated teeth became carious. Of those, six were sealed with GIS and six treated with FV. It was concluded that complete retention of the GIS was very low; however, no difference in caries increment between both treatments was found.

## 254 Orthopedic and orthodontic treatment of Angle Class II Division 1 malocclusion with Interlandi Headgear: a clinical case report

PUPULIM, D.C. (Daniela Cubas Pupulim danielapupulim@hotmail.com); PIERI, L.V.; HENRIQUES, J.F.C.; JANSON, G.; FREITAS, M.R.; PINZAN, A.

The interlandi headgear (IHG) is indicated mainly in the mixed dentition for upper molar distalization in the Angle Class II Division 1 malocclusion. A 9-year old girl with severe convex profile and severe horizontal trespass, without any space for the eruption of the permanent upper lateral incisors, was treated with a IHG appliance 24 hours/day with 350g each side for seven months. In the lower arch, a Schwarz appliance was applied in order to uprigth the lower teeth. Then, the upper and lower fixed appliances were placed to align and level the teeth. The IHG was efficient in moving upper molars distally with a body movement once the forces passed through the center of resistance bringing about sufficient space for eruption of the permanent teeth and a harmonic facial profile.

## 255 Surgical planning for extraction of non-erupted teeth using computed tomography

DANIELETTO, C.F. (Carolina Ferrairo Danieletto carol_danieletto@hotmail.com); COSTA, F.S.; CAMARINI, E.T.; IWAKI FILHO, L.; PAVAN, A.J.; FARAH, G.J.

Surgical extraction of impacted supernumerary or ectopic teeth is a relatively common procedure to the dentist. As every surgical intervention, there are anatomical considerations that predispose the patient to risks of intercurrences or complications, so it is indispensable to accurately determine the localization of the non-erupted teeth to improve surgical planning. Although conventional radiographic techniques are commonly used to detect the presence and location of the teeth, radiography can present some deficiencies. In this situation, additional exams such as computed tomography could be requested. We report the case of a 12-year-old patient that showed an upper third molar in an atypical position. Computer tomography was required as well as manipulation of three-dimensional images. The purpose of this study is to highlight the importance of computed tomography and three-dimensional reconstruction to accurately determine the localization of non-erupted teeth, enabling a better surgical planning and a safer intervention.

## 256 Prevalence of the oral alterations in 0-36-month-old babies attending an educative and preventive program

CAMARA, D.M. (Danielle Mendes da Camara camara_danielle@hotmail.com); PEREIRA, T.S.; SILVA, J.Z.; CUNHA, R.F.; NERY, R.S.; AGUIAR, S.M.H.C.A.

Oral alterations may be present at birth and others can manifest months or years later, reflecting changes in the growth and/or development of the of oral and maxillomandibular structures. The purpose of this study was to evaluate, by the analysis of medical records of babies aged 0 to 36 months enrolled in the Baby Clinic of the School of Dentistry, Aracatuba - Unesp, from January 2002 to December 2007, the prevalence of oral alterations and their distribution according to the type, age, gender and whether or not there was a need for treatment. 1713 records were analyzed, of which 158 (9.2%) had a record of oral alterations. The 0-12-month-old age group (53.2%) and females (50.6%) presented most oral alterations. Primary herpetic gingivostomatitis was the most prevalent oral alteration (25.3%). In 53.2% of the records, some kind of treatment for the diagnosed oral alteration was necessary. Thus, based on the methodology used and the obtained results, it may be concluded that oral alterations are not associated with gender and a treatment is needed in most cases. Moreover, the early dental care to the baby becomes increasingly close to reality and allows the diagnosis of several types of oral amendments, as well the clarification to parents and/or guardians about proper behavior.

## 257 Evaluation of the patient profile related to the type of trauma presented in FOUFU's dentoalveolar trauma clinic

FERREIRA, D.P. (Débora Pelegrino Ferreira deborapelegrino@yahoo.com.br); ALVARENGA, A.C.F.; ROSCOE, M.G.; SOARES, P.V.; SANTOS-FILHO, P.C.F.; SOARES, C.J.

The maintenance of the integrity of the dental structure and periodontium constitutes great challenge when dentoalveolar trauma occurs, and frequently results in loss of healthy dental structure. The trauma is almost always an emergency situation, demanding immediate prompt care with immediate diagnosis based in clinical and radiographic examination. This aim of this study was to evaluate the profile of the patients and type of trauma based on data from the dental charts of 98 patients treated at FOUFU's dentoalveolar trauma clinic, according to the type of trauma; involved tooth; gender and age of the patients. The results were analyzed by frequency. The frequency for age group distribution was: up to 5 years (1%); between 6 and 12 years (46,9%); between 13 and 19 years (19.3%); between 20 and 30 years (17.3%) and above 30 years (15.3%). The relation of the factors in study according to the ages was: between 6 and 12 years, the occurrence of dentoalveolar trauma was higher in men (48%), tooth #11 (44.3%) being the most often affected and crown fracture (56.1%) most frequent type of trauma. In general, the most frequent trauma was crown fracture (59.4%), which occurred predominantly in patients aged 6 to 12 years (56.1%). The teeth most involved, had been uppers central incisors (71.8%) for the position most vulnerable in the dental arch. Based on the knowledge of the type of trauma versus the patient profile, preventive programs must be developed, emphasizing the importance of the use of mouthguards for children in daily activities and mainly when engaged in sports or risky activities.

## 258 Surgically assisted rapid maxillary expansion

SOUZA, D.D. (Dennis Dinelly de Souza dennisouza@gmail.com); MORANDO, F.S.; NOSE, A.R.

Transverse maxillomandibular discrepancies are a major component of several malocclusions. A normal dental occlusion can be characterized by the perimeter of the upper jaw having dimensions slightly larger than the lower jaw. When these dimensions are smaller, there are transverse maxillary discrepancies in the lateral dimension. It is characterized by an atresic palate, which can cause diverse modifications in the occlusion of the patients, such as the posterior crossbite (unilateral or bilateral). For correction of this deficiency, patients who have mature bone may undergo surgically assisted rapid maxillary expansion, whose principles have been proven a great choice for the treatment of this deformity. In other cases, such as discrepancies greater than 4 mm, failure of previous orthodontic treatment and transverse discrepancy greater than 7mm, this technique also can be used. The present study has the objective to show all of the steps of a surgically assisted rapid maxillary expansion by reporting of the case of a 28-year-old female patient under general anesthesia, treated at Southern Regional Hospital, São Paulo, Brazil.

## 259 Why is it important to treat anterior crossbite early?

RODIO, D.C. (Diego Civiero Rodio rodio87@hotmail.com); ALMEIDA, M.R.; ALMEIDA, R.R.; OLTRAMARI-NAVARRO, P.V.P.; NAVARRO, R.L.; CONTI, A.C.C.F.

The current tendency of dentists, pediatric dentists and mainly orthodontists is to give a special emphasis on the problems related to the prevention and interception of malocclusions. Among them, anterior crossbite is one of the most frequently reported in the literature. The presence of this malocclusion can be related to a mandibular prognathism (Class III) or can appear associated to a Class I. Etiologically, it is provided by a trauma, lost or retention of the deciduous teeth, inadequate perimeter of the dental arch, interposition of the upper lip, ectopic eruption of permanent teeth. There is also the possibility to occur associated with the functional protrusive mandibular shift or because of a maxillary hypodevelopment. A differential diagnosis for the situations mentioned above is very important to make sure if there is a bone involvement (Class III) or only a dental involvement (Class I). The prevalence of the anterior crossbite reaches 7.6% among the malocclusions. The therapy depends on a differential diagnosis and is based on the correction of the buccolingual relationship of the anterior teeth, providing a normal growth and developing of the jaws. Among many treatment options, this work aims to emphasize the importance of the interceptive treatment in two clinical cases. On the first case, the anterior crossbite has as an etiologic factor the long retention of the deciduous central incisor, causing a deviation of the permanent right central incisor eruption. On the second case, the anterior crossbite was originated from a trauma on the deciduous incisors with displacement to the lingual region of the permanent incisor. This ectopic eruption can be associated with an occlusal trauma with the lower incisors causing a gingival recession. The treatment protocol was based on the use of a removable orthodontic appliance with double loops for the first case and a removable orthodontic appliance with expansive screws and a simple loop for the second case.

## 260 Epidemiological profile of caries and periodontal disease among schoolchildren in Porto Velho – Rondônia, Brazil

ALMEIDA, D.L. (Dino Lopes de Almeida almeida.dino@hotmail.com); GALVÃO, N.S.; NASCIMENTO, D.O.R.; MENEZES, K.E.; BATISTA, K.M.; DIAS, A.G.A.

The aim of this study was to make an epidemiological survey of caries and periodontal disease in 12-year-old schoolchildren of a municipal public school in a district of Porto Velho – RO, Brazil, in 2008. The indicators of the World Health Organization (WHO) were used to obtain data, DMTF to indicate the rates of caries, and the Community Periodontal Index (CPI) for periodontal disease. The examination was performed by 2 examiners and 2 registers, who were calibrated in advance according to Kappa index (0.84 and 0.81, intra- and inter-examiner, respectively). The participants in this study were 102 children of both genders. The results showed that 37.25% of the schoolchildren were caries-free (n=38). As for the mean DMTF, a result of 1.29 (±1.55) was obtained. The CPI of the examined adolescents showed that 48.2% of the teeth did not present gingival bleeding, 33.2% had bleeding, 17.3% had calculus, 0.2% sextants were excluded and 1.1 sextants were not examined. At the end of the stidy, it was observed that the schoolchildren presented a high prevalence of gingivitis compared to caries disease. Such results allow us to conclude that the need to implement educational measures of oral hygiene orientation, directed to the adolescent public of the schools.

## 261 Oral squamous cell carcinoma: retrospective analysis of 185 cases

FRANÇA, D.C.C. (Diurianne Caroline Campos França diurianne@terra.com.br); MONTI, L.M.; CASTRO, A.L.; SOUBHIA, A.M.P.; AGUIAR, S.M.H.C.A.

The squamous cell carcinoma is the most common malignancy in the oral cavity, representing over 90% of cancers in this region. This study aimed to conduct an epidemiological study of oral squamous carcinoma cases diagnosed by the Department of Pathology, Department of Pathology and Clinical Propaedeutics of the Dentistry Faculty of Araçatuba - UNESP, in the period between 1995 and 2005. 185 cases were studied, it was observed that the oral floor and tongue were the sites most affected, with predominance in males, Caucasians and aged between 41 and 60 years, noting that the majority of patients were smokers. Knowledge of these data is important for the dental surgeon to act preventively, contributing to early diagnosis of cancer.

## 262 Adenomatoid odontogenic tumor: case report

DUARTE, B.G. (Bruno Gomes Duarte duarte.ctbmf@gmail.com); TJIOE, K.C.; GONÇALES, E.S.; SANT'ANA, E.; FERREIRA JÚNIOR, O.; OLIVEIRA, D.T.

Adenomatoid odontogenic tumor (AOT) is a rare odontogenic lesion that accounts for 3 to 7% of all odontogenic tumors. It presents as a slow growing symptom-free lesion and shows predilection for the anterior region of the maxilla and young females. Radiographically, AOT presents as a radiolucent lesion, with regular margins, associated with an enclosed tooth – most commonly canine. On the histological exam of a classic AOT lesion, it is possible to see numerous spindle-shaped cells forming rosette as well a the characteristic duct. Calcified areas are present in most AOT. The diagnoses has being based in the clinically, radiographic and histopathological reports. Surgical enucleation is the treatment of choice, because AOT have a benign behavior with rare recurrence. The aim of this study is presents a case of adenomatoid odontogenic tumor with 4 years of follow-up. The patient, W.H, male, 12 years old, presented with main complain of delay in the exfoliation tooth 53. In the intraoral examination revealed painless swelling in the right region of maxillary. A rigid mass and mucosa integrity was observed. Radiographic examination revealed an extensive radiolucent area, with well-defined margins. Tooth 13 was involved by the lesion and was displaced by the mass. The first diagnostic was dentigerous cyst. After incisional biopsy and microscopic analysis we could detect a virtual cavity coated by a delicate cuboidal epithelium. It could be observed proliferation of odontogenic epithelial cells and duct-like formation. As the final diagnosis of AOT, the lesion was removed by enucleation together with the tooth, and the patient was followed after 8, 14, 60 and 180 days, and after 4 years, not showing clinical or radiographic signs of tumor recurrence.

## 263 Clinical evaluation and subgingival recolonization after SRP in smokers and non-smokers with chronic periodontitis

MUNCINELLI, E.A.G. (Eduardo Augusto Galbiatti Muncinelli eduardo_muncinelli@hotmail.com); BERNAL, M.A.C.; MATARAZZO, F.; FAVERI, M.; FERES, M.; FIGUEIREDO, L.C.

Smokers normally respond less favorably to periodontal treatment. The objective of the study was to evaluate the clinical response and the subgingival bacterial recolonization after scaling and root planning (SRP) in smoking (S Group, n=15) and non-smoking individuals (NS Group, n=15) with chronic periodontitis. Clinical and microbiological evaluations were performed. Subgingival samples were analyzed by Checkerboard DNA-DNA hybridization. SRP did not exceed 21 days. The clinical examination was performed in the beginning, 90 and 180 days post-therapy, and microbiological in the beginning, immediately after SRP, 42, 90 and 180 days. The SRP promoted clinical significant improvements in all the parameters in the group NS (p<0.05), however, for the S group differences were not observed in the gingival bleeding and bleeding on probing (p>0.05, Wilcoxon Test). The greatest microbiological benefits was noticed in NS group, where P. gingivalis was maintained in inferior levels (p<0.05) up to 180 days; and the beneficial species increased from 20.1% for 50.8% (p<0.05), and the pathogenic increased from 72.6% to 27.3% (p<0.05). The S group beneficial species passed from 24.1% to 35.3% (p<0.05), however the pathogenic species passed of 69% for 55.3% (p>0.05) to 180 days. In conclusion, the therapy of SRP promoted clinical improvements in the smokers and non-smokers. However, the recolonization of the subgingival environment by pathogenic microorganisms was more obvious in the smokers.

## 264 Analysis of marginal adaptation of three endodontic sealers used as apical plug

CONSOLMAGNO, E.C.; OROSCO, F.A; BRAMANTE, C.M.; BERNARDINELI, N.; GARCIA, R.B.; MORAES, I.G.

This study evaluated the marginal adaptation of apical plug fabricated with three types of endodontic sealers in roots with apical foramen enlarged. Ninety single-rooted teeth with a single canal were used. The root canals were prepared by crown-down technique with Gates Glidden burs in decreasing order, from bur #5 to #1. After this procedure, the root canals were enlarged with K files #50 to #90. Following, the teeth were divided into 3 groups of 30 specimens each, according to the materials employed to fabricate the 5-mm-thick apical plugs: group 1 – Gray MTA-Angelus®; group 2 – CPM® and grupo 3 – MBPc sealer. Lateral condensation technique was used for obturation and each properly labeled, subgroup was placed in an oven at 37 °C for 48 hours. After, the teeth were longitudinally worn with carborundum discs, gold-sputtered and analyzed by scanning electron microscopy, recording SEM micrographs at 35X and 150X magnifications. For analysis of the marginal adaptation of sealers, the SEM micrographs at 35X magnification were analyzed by the Image Tool 3.0 software and the extension of misfit was measured linearly, in micrometers. The results were tabulated and analyzed statistically by Kruskal-Wallis and Dunn's tests with a significant level of 5%. CPM® sealer had better results though without statistically significant difference.

## 265 Differential association of genetic, microbial and inflammatory factors with MMP-1 levels in healthy and diseased periodontal tissues

SILVEIRA, E.M.V. (Elcia Maria Varize Silveira elcia_mvs@hotmail.com); REPEKE, C.E.; TROMBONE, A.P.F.; FERREIRA-JR, S.B.; MARTINS JR, W.; GARLET, G.P.

The basis of individual susceptibility to periodontitis, an infectious inflammatory disease, involves a high individual variation in the matrix metalloproteinase-1 (MMP-1) levels, which can be due genetic polymorphisms and/or specific periodontopathogens. This study investigated the influence of genetic (MMP1-1607 SNP) and microbial (P. gingivalis, T. denticola, T. forsythia - red complex and A. actinomycetemcomitans) factors in the determination of MMP-1 mRNA levels in periodontal tissues of chronic periodontitis CP (N=178) and control C (N=190) groups. MMP1-1607 SNP was investigated by PCR-RFLP, while the MMP-1 mRNA levels and the periodontopathogens presence and load were determined by RealTime-PCR. No significant differences were found in the frequency of MMP1-1607 genotypes in C and CP groups. In healthy tissues MMP1-1607 2G allele was associated with higher MMP-1 levels while in CP MMP-1 levels were associated with the presence and load of periodontopathogens, and also with TNF-á and IL-1â expression irrespective of MMP1-1607 genotype. In vitro data demonstrate that 2G macrophages low and intermediate dose LPS and TNF-á+IL-1â stimulation was associated with increased MMP-1 expression, while strong and repeated stimulation resulted in higher MMP-1 levels irrespective of MMP1-1607 genotype. Our data demonstrate a limited role for MMP1-1607 SNP in periodontitis, where the extensive chronic antigenic challenge exposure overcome the genetic control and plays a major role in determination of MMP-1 expression.

## 266 Free gingival graft healing: effects of smoking

CAMARGO, E.C.V. (Ellen Cristina Violi Camargo lencristina.v.c@hotmail.com); SALLUM, A.W.; SILVA, C.O.

The aim of this research was to evaluate the influence of tobacco smoking on the levels of free gingival graft (FGG) shrinkage and on the repair of the graft donor area. Ten smokers and twelve non-smokers patients, with <1mm of attached gingiva associated with oral hygiene deficiency, high frenulum attachment or gingival recession were selected to receive FGG for keratinize tissue augmentation. The clinical parameters of probing depth (PD), gingival margin level (GML), clinical attachment level (CAL), keratinized tissue (KT), gingival thickness (Tkn), and FGG high, length, and area were evaluated. The palate donor area was evaluated by immediate bleeding (IB) and complete wound epithelialization (CE). The parameters were evaluated before surgery, 7, 15, 30, 60, and 90 days postsurgery. The outcomes of the present study show that, instead of the absence of statistically significant difference between smokers and non-smokers, there is a tendency for a negative impact of tobacco smoking. At 3 months postsurgery, the percentage of shrinkage of FGG area, high, and length in smokers (58%, 44%, and 25%, respectively) was greater than in non-smokers (44%, 31%, and 22%, respectively), but there was no statistically significant difference. The final amount of KT increased in both groups, but was slightly smaller in smokers (4.91mm) than in non-smokers (5.49mm). A smaller percentage of smoker patients presented immediate bleeding than non-smoker patients (30% and 75%, respectively), the same occurred with complete wound epithelialization (20% and 91.67%, respectively) at 15 days postsurgery. Within the limits of the present study, it may be concluded that FGG is an adequate technique for keratinized tissue augmentation in both smokers and non-smokers. Cigarette smoking interferes with palate epithelialization and bleeding, however, it does not have a negative impact on the clinical outcomes of FGG shrinkage.

## 267 Effects of ions released from dental materials on enamel demineralization

MISSEL, E.M.C. (Emilene Macario Coimbra Missel emilene.mc@bol.com.br); VIEIRA, A.E.M.; DELBEM, A.C.B.; DANELON, M.; CANNON, M.; STOCK, S.R.

The aim of this study was to evaluate the effect of calcium, phosphate and fluoride ions released from dental materials when interfering with caries dynamics, using bovine enamel, pH-cycling model and synchrotron XMT. Fifty enamel blocks (4x3mm) were selected through surface hardness analysis. Five impressions spaced 100 μm from each other were made at 300 and 600 μm from the edge. Specimens (n=10) were prepared from 5 materials: sealant with ACP, sealant with ACP and F (experimental), sealant with F, sealant without F, and a resin-modified glass-ionomer. Next, the specimens attached to the enamel blocks were individually subjected to a pH-cycling model to promote demineralization [Vieira et al.: Caries Res 2005;38:514-520]. Surface hardness was measured again at 300 and 600 μm from the enamel/sealant interface. Synchrotron XMT was performed to analyze the lesion profile and to calculate the mineral concentration (gHAp cm-3) at each 2.8 μm, starting at the surface up to 221.2 μm. The highest mineral content in the first layer of enamel was found in the group of products with anti-caries ions. Specimens of sealant with no F and sealant with ACP led to the lowest mineral content when analysis was performed at the bottom of the caries lesion. The results of this *in vitro* model show that the presence of fluoride in a sealant material can help reduce enamel susceptibility to caries formation. Therapeutic sealants should be further investigated and their use encouraged in situations where enamel demineralization is suspected.

## 268 Evaluation of the alteration of the brush bristles after brushing with dentifrices for complete denture

PEREIRA, E.E.S.S. (Eric Evander Samuel da Silva Pereira eriverp@yahoo.com.br); MACEDO, A.P.; PISANI, M.X.; PARANHOS, H.F.O.; SILVA-LOVATO, C.H.

This study evaluated the change in the of brush bristles after brushing with water and with 4 dentifrices, one for natural teeth (Sorriso®) and three for complete dentures: Corega®, Experimental 1 (Zonil), Experimental 2 (Chloramine T). Brushes with 26 soft tufts of bristles of 0.25 mm in diameter and 10mm in height were used. The assay was performed brushing with the aid of a machine of type Pepsodent, in which the dental brushes aid to suspension of dentifrices brushing the specimens of acrylic resin (Plex-glass). For each test were used 06 brushes for each specimen. The time used for brushing was 50 minutes (one year/17,800 cycles). After the assay, the brushes were removed, washed and dried with absorbent paper sheets. For analysis of bristles, 10 brushes were separated from each group: water, Experimental 1, Experimental 2, Corega® and Sorriso®. As a control, were used bristles of brush not tested. Of each bristle of brush was withdrawn a total of 10 bristles per group. Then, the bristles were placed in a Plex-glass plate so that all of them were in the same plane. Measurement of the diameter was performed in a profilometer accurate to tenths of millimeters (0.01 mm) at 0.02 mm from the tip of the bristle. Ten values for each combination (toothbrush and dentrifices) tested were obtained. Data analysis was performed using the ANOVA (P <0.05). The results indicated that only the group Sorriso® (0.15 ± 0.02) when compared with the control group (0.2 ± 0.02) showed statistical significance (P = 0.0117), while the other examined groups (water: 0.18 ± 0.02; Corega®: 0.17 ± 0.2; Experimental 1: 0.16 ± 0.02; Experimental 2: 0.16 ± 0.02) showed no statistically significant values. All dentifrices promoted changes on the end of bristles, but Sorriso® caused, statistically, the greatest changes.

## 269 Infection of the facial and cervical spaces in a patient with systemic disease

AVILA, E.D. (Erica Dorigatti de Avila erica.fobusp@yahoo.com.br); MOLON, R.S.; GABRIELLI, M.F.R.; GABRIELLI, M.A.C.; HOCHULI-VIEIRA, E.

The anamnesis is essential to detect possible preoperative systemic alterations and, consequently, to direct the professional's behavior. Diabetes mellitus results from an absolute or relative insufficiency of insulin because the pancreas does not make sufficient hormone or cannot use its own insulin as well as it should, or both. When the disease is not adequately controlled some complications may arise. Therefore, it is necessary to run tests disclose these alterations. In the case at issue, the patient came to the Department of Oral Diagnosis and Surgery of the Araraquara Dental School, UNESP, presenting pain and swelling in the mandibular and cervical region, and areas of erythema in the neck. The patient was admitted with deep neck space infection after installation of a healing dental implant. Initially the patient presented discrete edema of submandibular spaces. However, the infection had fast evolution because the patient had uncontrolled diabetes, developing serious complications and needing surgical treatment. Urgency tracheostomy was performed due to the patient impossibility of receiving the endotracheal intubation for general anesthesia, draining of deep neck submandibular, sublingual, lateral pharyngeal spaces and the subcutaneous cervical tissue, as well as draining of the thorax for evacuation of pleural spill. Therefore, it is important to emphasize that the simplicity of the procedure can be aggravated and, many times, taking to death when some information of the anamnesis is overlooked.

## 270 The contribution of speech therapy to facial esthetics

SITTA, E.I. (Érica Ibelli Sitta kek32@hotmail.com); SALLES, M.A.; ARAKAWA, A.M.; BASTOS, R.S.; CALDANA, M.L.; BASTOS, J.R.M.

The demand for treatment in facial esthetics has become a constant in practices. Patients of both sexes concerned about a harmonious and rejuvenated facial appearance go to speech therapy clinics in the search for this type of treatment. This study aimed to highlight the benefits of speech therapy in a case study to provide esthetic improvement in the patient's face with the performance of orofacial movement. The study was held in a private speech therapy clinic in the city of Araraquara after orthodontic intervention and describes the analysis of records of a female patient, 56 years old, by means of history, initial assessment with photographs of the face, treatment, guidelines and final evaluation with new photographs for comparison. The data dashed changes in orofacial muscles as hypotonia, and inadequate function of mastication by chewing preference and presence of left sided pain in the temporomandibular join (TMJ) with pressing dental. The patient attended 15 sessions speech therapy for, including specific approaches of orofacial movement and mobility, guidelines for care in cleaning and protecting the skin, quality of daily diet and the importance of sleep and well being. Having knowledge of the patient's inadequate facial expressions habits, therapies aimed at the muscle harmonization as well as massages for relaxation and wrinkle smoothing were performed. The facial discomfort was resolved by removal of the tooth clenching habit with the help of the minimization of the points of tension and both articular and muscle pain, as well as changes in the masticatory pattern and quality of life, improving the harmony of both hemi-faces. It is concluded that the benefits of the adequation of the orofacial muscles and stomatognathic functions provided together with the balance of form and function a sensitive rejuvenation.

## 271 Labor Dentistry as a tool of support to Forensic Dentistry: a proposal of inclusion and maintenance of workers' dental charts

CARVALHO, E.S. (Érica Silva Carvalho carvalho.odonto@gmail.com); RODRIGUES, L.M.V.; SALES-PERES, S.H.C.; HORTENSE, S.R.; SALES-PERES, A.C.; SALES-PERES, A.

Forensic Dentistry has a role of extreme importance in identifying the victims of airspace and industrial accidents, natural disasters and terrorist attacks. The identification of a corpse is usually made by pictures, comparing the radiographic images, fingerprints and DNA. However there are some limitations in these techniques that are ineffective when the body is found in decomposition or putrefaction process which makes it impossible to recognize the body. Especially in these cases, the identification by the dental arch is essential, because the teeth can withstand extreme degradation conditions, such as exposure to high temperatures, humidity and high pressure. The use of dental charts in the process of corpse's identification is essential in these cases to obtain a correct result being, however, necessary the cooperation of dental professionals by having a vast and organized documentation in their offices, which is not common in the current Dentistry practice. Therefore, it is really important for each worker to have his/her own documentation registered in one's workplace, making the coroner's job easier. This study aimed at demonstrating that it is the Labor Dentistry's role keeping and protecting the workers' dental charts in their workplace, since there are valuable and individual information in such documentation ranging from a possible dentist's legal defense process to the cases of body identification.

## 272 Evaluation of clinical and esthetic results obtained with free gingival graft or free connective tissue graft

SILVA, E.R. (Erick Ricardo Silva erick.silva@usp.br); MAIA, L.P.; LUCIA, C.I.; SOUSA, S.L.S.; REINO, D.M.

Free gingival graft and free connective tissue graft are periodontal surgical procedures used to increase the amount of attached gingiva, by increasing the amount of keratinized tissue in attempt to cover root surface in areas of gingival retraction. Free gingival graft was the first technique described, and shows satisfactory results, but has as as disadvantage the formation of attached gingiva with a different color when compared to the adjacent area. This disadvantage has been attributed to the transplanted palatal epithelium. Thus, the use of connective tissue without epithelium has been suggested to avoid this problem and to obtain better esthetic results. However, it is believed that the connective tissue graft has an increased contraction and less formation of keratinized tissue. The objective of this report is to compare the procedures of free connective graft and free gingival graft. A male patient with lack of keratinized tissue in both mandibular canines, received supragingival scaling, plaque control and oral hygiene instructions. When the plaque index was less than 20%, the patient received in each lower canine, a different type of graft, the palate being the donor area. After evaluation at one week, two weeks, one month and six months, it could be noted the formation of wide gingiva attached, keratinized tissue with good thickness and maintenance of periodontal health in both lower canines. Thus, both procedures were effective to create keratinized tissue in the patient treated, with an excellent functional and esthetic result.

## 273 Periodontal esthetics in Miller's Class III gingival retraction and Tarnow's Class III papilla defect - case report

GUALBERTO JÚNIOR, E.C. (Erivan Clementino Gualberto Júnior erivangualberto@hotmail.com); FERNANDES, L.A.; MURAKAWA, A.C.; BOSCO, A.F.; GOUVEIA, V.G.

The esthetic concept is quite wide and variable judging recognizably subjective values. The idea that the periodontal treatment increases the proximity spaces harming the esthetic result it is an incorrect interpretation of the available resources in the periodontology. The repair of inflamed areas will result in tissue contraction when reducing the edema, but it is possible to minimize undesirable results, or even correct existing defects, improving the esthetics. The surgical procedures used in the treatment of retraction defects can be classified as (1) Soft tissue pedicle graft (2) Soft tissue free graft. Following Miller's classification, total coverage of Class I and Class II defects is perfectly predictable, Class III defects will result in a partial coverage and Class IV defects will not be covered due to advanced interdental loss of bone and soft tissue proximity. We will tell a clinical case of a patient of feminine gender, 28 years old, that presented loss of the interdental papilla among the teeth 21 and 22 with Miller's Class III gingival retraction caused by localized periodontitis. The radiographic exam suggested vertical bone loss from middle to the apical root third. After basic periodontal treatment, laterally positioned flap was elevated with connective tissue graft in order to increase root coverage and reconstitute the interdental papila. The postoperative follow up showed partial root coverage and increase of the soft tissue thickness in the interdental papilla. Six months after this intervention, a second surgical procedure was accomplished using the tunnelization technique and subepitelial connective tissue graft aiming to improve even more the esthetic results obtained. The 1-month postoperative evaluation showed increase of root coverage and interdental papilla reconstitution. The proximal faces of teeth #21 and #22 were restored to mask the “black space”.

## 274 Main pharmacological interactions with antimicrobials of interest in dentistry

SILVA, E.J. (Everton José da Silva evertonjs2@hotmail.com); MUNCINELLI, E.A.G.; OLIVEIRA, R.M.W.

Antimicrobials are medicines widely used in dentistry practice because they are suitable as coadjuvant in several procedures focusing patient\'s recovery. Among the antimicrobials more prescribed by the dentists are the beta-lactams, the lyincosamides, the macrolides, metronidazole and the tetracyclines. For the success of the treatment and the patient\'s safety, it is important that to recognize and understand the main pharmacological interactions of the antimicrobials and their current complications. In addition, the dentist must be able to identify the possible pharmacological interactions as well as to propose appropriate therapeutic alternatives to the proper treatment. The aim of this work was to elucidate the main pharmacological interactions related to the most commonly use antimicrobials in the Dentistry. By means of a literature review we presented the pharmacological interactions among antimicrobials and drugs anesthetic, anti-inflammatory and anxiolytic drugs, as well as the measures that might be taken with the intention of preventing possible complications. Pharmacological interactions happen when the action of a medicine is altered by the presence of other. This alteration can lead to a decrease of the effectiveness or increase of the pharmacological activity, which can produce adverse and unexpected pharmacological events. The pharmacological interactions can be classified in pharmacodynamic interactions, which result from an alteration of the capacity of a medicine to interact with its target or action site, and pharmacokinetic interactions, which result from an alteration of the concentration of the medicine secondary to absorption, distribution, metabolism or excretion processes. Knowing pharmacological interactions caused by drug associations is important in educating dentists. The most efficient way of prevent pharmacological interactions is to guarantee that dentists get knowledge about the mechanisms and consequent complications involved in such interactions, and thus be able to propose proper therapeutic strategies to an efficacious treatment.

## 275 Influence of the sweetener type of cola drinks on erosion subjected or not to subsequent abrasion: an *in situ*/ex *vivo* study

SANTOS, F.Z. (Fabiana Zaidan Cardoso dos Santos - fahzai_dan@hotmail.com); SANTOS, F.Z.C.; HONÓRIO, H.M.; MAGALHÃES, A.C.; BUZALAF, M.A.R.; RIOS, D.

FAPESP (Proc. 2007/07296-1).

This *in situ*/ex *vivo* study evaluated whether the type of cola drink (regular or light) could influence the wear of bovine enamel subjected to erosion followed by brushing abrasion. During an experimental 7-day phase, 10 previously selected volunteers wore intraoral palatal devices, with 8 bovine blocks which were subjected to four groups: (ER) erosion with regular cola, (EAR) erosion + abrasion with regular cola, (EL) erosion with light cola and (EAL) erosion + abrasion with light cola. During 7 days, half of the palatal device was immersed in light cola drink (4 blocks) for 5 min and 2 blocks were brushed using a fluoride dentifrice. Immediately after, the other half of the appliance was immersed in regular cola drink and the brushing procedures were repeated. The pH and phosphorus, calcium and fluoride concentrations were analyzed using standard procedures (Light cola- pH: 3.0; 13.7 mg Ca/L; 15.5 mg P/L and 0.31 mg F/L; Regular cola- pH: 2.6; 32.1 mg Ca/L; 18.1 mg P/L and 0.26 mg F/L). The amount of base (NaOH 0.2 M) added to raise the pH to 7.0 was 0.125 mL for both colas. Enamel alterations were measured using wear profile tests (mm). The data were analyzed by ANOVA and Tukey's test (p<0.05). The light cola drink (EL-0.36/EAL-0.39) promoted less wear than the regular one (ER-0.72/EAR-0.95) for both conditions (erosion and erosion + abrasion). There was no difference between erosion and erosion + abrasion for the light cola drink; however for the regular cola drink the erosion resulted in less wear when compared to erosion + abrasion. The data suggest that the light cola drink promoted less enamel wear even when erosion was followed by brushing abrasion.

## 276 Stafne bone cyst – radiolucent area in the mandible versus bone pathologies: case report and literature review

RIZZANTE, F.A.P. (Fabio Antonio Piola Rizzante fabioantonio_7@hotmail.com); NERI, N.B.D.; SAMPIERI, M.B.S.; DIAS-RIBEIRO, E.; SANT'ANA, E.; FERREIRA JÚNIOR, O.

Stafne bone cyst is a bone cavity in the mandible containing submandibular gland. It was described for the first time in 1942 by Edward Stafne, who reported a well-defined, unilocular radiolucency located in the posterior mandible below the mandibular canal. The Stafne bone cyst is usually an asymptomatic incidental radiologic finding and is much more frequent in males, with a male/female ratio of 6:1. This is a rare defect with prevalence ranging from 0.10 to 0.48%. The purpose of this study was to report a clinical case and present a literature review with the aim of increasing the knowledge of the etiology, epidemiological findings and differential diagnosis of this cyst. Cone beam computed tomography has been used as one of the most important techniques to confirm the diagnosis of Stafne bone cyst. Although panoramic radiographs may be sufficient for the diagnosis in some cases, they may not be enough for a definitive diagnosis in many other cases, especially, when the lesion is atypical. In conclusion, complementary techniques such as computed tomography are sufficient to establish an accurate diagnosis, avoiding invasive procedures, such as exploratory surgery and sialography.

## 277 The importance of the interdisciplinary approach in the treatment of patients subjected to orthognathic surgery in the correction of dentofacial deformities

YOSHIDA, F.S. (Fabio Shigueru Yoshida fabio.yoshida@usp.br); SILVA, A.S.C.; BERRETIN-FELIX, G.

The orthognathic surgery aims to correct cases of dentofacial deformities and this procedure usually results in structural and functional variations of the stomatognathic system. Therefore, the adaptation of the tissues surrounding these structures becomes necessary in order to have an adequate execution of the aimed functions. The objective of the present study is to present the necessities of an interdisciplinary work in the treatment of individuals with dentofacial deformities, concerning to involvement of Speech Therapy and Dentistry specialties. For this, a literature review was carried out from the Lilacs, Medline and Pubmed database, in the last 13 years, aiming to identify studies on diverse variables that approached the impact of the orthognathic surgery in the stomatognathic structures and functions. As result, we retrieved 10 international papers that met these requirements. Among the found implications, the published papers pointed out that the orthognathic surgery results in modifications of the masticatory function, such as electromyographic activity, bite force and functional pattern, however, without reaching normal values. Due to these surgeries, we also can find variations of the breathing flow and nasal area, variations of the resonance, of the face mimics the muscle activity, variations in mandibular mobility, in the speech articulation aspects, and also of creating sensitivity alteration and deglutition function. These findings come to prove that the correction of the structures, carried through for the orthognathic surgery, seems not to be enough for the effective modification of the presented functional alterations, once that the orofaciais functions are founded modified in the postoperative time. Thus, the speech therapy becomes heavily important in the adequacy of functions in order to lead the muscular functioning for balanced standards, becoming possible the harmonious interaction in the stomatognathic system components relations, preventing orthodontic relapse and guaranteeing the treatment stability. Finally, the interdisciplinary performance must include dentistry and speech therapy cooperative actions in the different phases of this treatment, guaranteeing integral rehabilitation and adequacy of the individuals esthetic and functional conditions.

## 278 Early stimulation for babies with Down syndrome

DIOGO, F.S.F. (Fabíola Diogo de Siqueira Frota fabdiogo@hotmail.com); AGUIAR, S.M.H.C.A.

Down's syndrome is a chromosomal disorder caused by trisomy of chromosome 21. Its incidence is approximately 1: 800 births. Presented as general characteristics of delayed development, congenital heart disease, generalized muscle hypotonia, facial development changes, almond eyes, hypotelorism, increased susceptibility to infections and oblique palpebral fissures. The early stimulation is a key resource to mitigate risk or delay the development of children with Down's syndrome, which have difficulties in learning and associated clinical complications. Early stimulation is the term used to mean the multidisciplinary work characterized by a dynamic set of human and environmental, which aim to provide meaningful experiences in the first 3 years of life of children with special educational needs, promoting their development. The participation of parents is fundamental and through the guidance of the professional they feel more emotionally prepared to deal with the difficulties and encourage the potential of their children. The preventive dental care gives favorable results, especially when started at an early age, so the baby can acquire oral health habits. The timeliness is a key factor not only to prevent, but also to eliminate the negative stigma about the dental care. This purpose of this work was to demonstrate the importance of early stimulation programs and present the clinical and oral characteristics of babies with Down syndrome, emphasizing the importance of prevention of dental caries since the first year of life in order to improve the quality of life of these patients.

## 279 Knowledge of dental prosthesis technicians and students about cross infection (prevention and control)

CUNHA, F.G. (Fabrícia Gonçalves Cunha fabricia_dcunha@hotmail.com); PAIVA, R.C.; BATISTA D.E.; CARVALHO, M.L.; ARCIERI, R.M.

The objective of this study was to evaluate the perception and characterize the knowledge and laboratory practices of students of the UFU' technical course in the dental prosthesis and dental prosthesis technicians (DPTs) registered at CROMG in the city of Uberlândia. Questionnaires were applied in the form of interviews to 69 individuals, randomly selected 43 professionals and 26 students from 1st and 2nd year, who agreed to participate in this study. The results showed that age ranged from 19 to 42 years for students and 23 to 63 years for the professionals. In relation to the work received, it was observed that both groups were evaluated molds and models kindness 42.30% (students) and 37.20% (DPT). The question “The profession of DPT offers risk of diseases that can be acquired at work?” The majority of respondents answered that yes. Regarding the question: which diseases offer risk of infection? For students and DPTs, the main infectious diseases were AIDS (18.60% and 16.07% respectively) and hepatitis (48.83% and 58.92% respectively) and the form of acquiring these diseases was consensual for both groups: “contaminated impressions”. Personal protective equipments were used by 96.15% of students and 93.02% of DPTs. Of the respondents, 79.71% were vaccinated to avoid contracting diseases. The occurrence of accidents at work was evident in 47.82% of participants, cutting and drilling being the most frequent causes. Based on these results, we can conclude that although both groups of respondents were aware of the risk that the profession of DPT offers, students have more information about it.

